# Tensor GSVD of Patient- and Platform-Matched Tumor and Normal DNA Copy-Number Profiles Uncovers Chromosome Arm-Wide Patterns of Tumor-Exclusive Platform-Consistent Alterations Encoding for Cell Transformation and Predicting Ovarian Cancer Survival

**DOI:** 10.1371/journal.pone.0121396

**Published:** 2015-04-15

**Authors:** Preethi Sankaranarayanan, Theodore E. Schomay, Katherine A. Aiello, Orly Alter

**Affiliations:** 1 Scientific Computing and Imaging (SCI) Institute, University of Utah, Salt Lake City, Utah, United States of America; 2 Department of Bioengineering, University of Utah, Salt Lake City, Utah, United States of America; 3 Department of Human Genetics, University of Utah, Salt Lake City, Utah, United States of America; Deutsches Krebsforschungszentrum, GERMANY

## Abstract

The number of large-scale high-dimensional datasets recording different aspects of a single disease is growing, accompanied by a need for frameworks that can create one coherent model from multiple tensors of matched columns, e.g., patients and platforms, but independent rows, e.g., probes. We define and prove the mathematical properties of a novel tensor generalized singular value decomposition (GSVD), which can simultaneously find the similarities and dissimilarities, i.e., patterns of varying relative significance, between any two such tensors. We demonstrate the tensor GSVD in comparative modeling of patient- and platform-matched but probe-independent ovarian serous cystadenocarcinoma (OV) tumor, mostly high-grade, and normal DNA copy-number profiles, across each chromosome arm, and combination of two arms, separately. The modeling uncovers previously unrecognized patterns of tumor-exclusive platform-consistent co-occurring copy-number alterations (CNAs). We find, first, and validate that each of the patterns across only 7p and Xq, and the combination of 6p+12p, is correlated with a patient’s prognosis, is independent of the tumor’s stage, the best predictor of OV survival to date, and together with stage makes a better predictor than stage alone. Second, these patterns include most known OV-associated CNAs that map to these chromosome arms, as well as several previously unreported, yet frequent focal CNAs. Third, differential mRNA, microRNA, and protein expression consistently map to the DNA CNAs. A coherent picture emerges for each pattern, suggesting roles for the CNAs in OV pathogenesis and personalized therapy. In 6p+12p, deletion of the p21-encoding *CDKN1A* and p38-encoding *MAPK14* and amplification of *RAD51AP1* and *KRAS* encode for human cell transformation, and are correlated with a cell’s immortality, and a patient’s shorter survival time. In 7p, *RPA3* deletion and *POLD2* amplification are correlated with DNA stability, and a longer survival. In Xq, *PABPC5* deletion and *BCAP31* amplification are correlated with a cellular immune response, and a longer survival.

## Introduction

The growing number of large-scale high-dimensional datasets recording different aspects of a single disease promise to enhance basic understanding of life on the molecular level as well as medical diagnosis, prognosis, and treatment. This is accompanied by a fundamental need for mathematical frameworks that can create one coherent model from multiple datasets arranged in multiple order-matched, column-matched, and row-independent tensors, i.e., tensors of the same number of dimensions each, with one-to-one mappings among the columns across all but one of the corresponding dimensions among the tensors, but not necessarily among the rows across the one remaining dimension in each tensor. Consider, e.g., the structure of the DNA copy-number datasets in the Cancer Genome Atlas (TCGA) [1, 2]. Profiles of tumor and normal tissues from the same set of patients have the structure of two matrices, i.e., second-order tensors, with a one-to-one mapping between the columns that correspond to the same set of patients, but not necessarily between the rows that correspond to the DNA copy-number probes with valid data in either the tumor or the normal dataset, and may be different. When the tumor and normal profiles are measured in replicates, e.g., by the same set of profiling platforms, then the structure of the tumor and normal datasets is that of two third-order tensors, of matched columns that correspond to the same sets of patients and platforms, and independent rows that correspond to the probes in either the tumor or the normal dataset.

The higher-order generalized singular value decomposition (HO GSVD) is the only simultaneous decomposition to date of more than two such column-matched but row-independent datasets, which is by definition exact, and which mathematical properties allow interpreting its variables and operations in terms of the similar as well as dissimilar, e.g., biomedical reality among the datasets [3, 4]. The HO GSVD generalizes the GSVD [5–12], which was demonstrated in comparative modeling of, e.g., patient-matched but probe-independent glioblastoma (GBM) brain tumor and normal DNA copy-number profiles from TCGA [13]. The modeling uncovered a previously unrecognized genome-wide pattern of tumor-exclusive copy-number alterations (CNAs). Prior to the modeling, DNA copy-number subtypes of GBM predictive of survival and response to chemotherapy were not conclusively identified [14, 15], and the best predictor of GBM survival was the patient’s age at diagnosis [16, 17]. Survival analyses [18, 19] showed and validated that the pattern is correlated with a GBM patient’s prognosis and response to chemotherapy, is independent of age, and together with age makes a better predictor than age alone. Segmentation [20, 21] of the pattern showed that it includes most known GBM-associated changes in chromosome numbers and focal CNAs, as well as several previously unreported, yet frequent CNAs. This suggested that the pattern is not only correlated, but also possibly causally coordinated with the GBM tumor’s pathogenesis. Previously unrecognized targets for personalized GBM drug therapy were also suggested, the tousled-like kinase 2 *TLK2* and the methyltransferase-like 2A *METTL2A* [22–24]. The GSVD comparative modeling, therefore, resulted in new insights into the poorly understood relations between a GBM tumor’s genome and a patient’s survival phenotype.

The GSVD and HO GSVD, however, are limited to datasets arranged in second-order tensors, i.e., matrices. We define, therefore, a novel tensor GSVD, i.e., an exact simultaneous decomposition of two datasets, arranged in two higher-than-second-order tensors of matched column dimensions but independent row dimensions. The tensor GSVD factors or separates the pair of tensors into corresponding pairs of “subtensors”, i.e., pairs of outer products or combinations of a paired set of patterns each: patterns, one across each of the matched column dimensions, which are identical for both tensors, combined with one pattern across the independent row dimension of either one of the two tensors. The pairs of subtensors are of varying relative mathematical significance, i.e., the significance of one subtensor in a pair in the corresponding tensor relative to the significance of the second subtensor in the second tensor varies among the pairs of subtensors. We prove that the tensor GSVD extends the GSVD and the tensor higher-order singular value decomposition (HOSVD) [25–28] from a decomposition of either two column-matched matrices or one tensor, respectively, to a decomposition of two order-matched, column-matched, and row-independent tensors [29]. We also show that the mathematical properties of the tensor GSVD allow interpreting the subtensors in terms of the biomedical similarities and dissimilarities between the two corresponding high-dimensional datasets.

We demonstrate the tensor GSVD in comparative modeling of patient- and platform-matched but probe-independent ovarian serous cystadenocarcinoma (OV) tumor and normal DNA copy-number profiles from TCGA. Most of the tumors, i.e., >95%, are high-grade tumors [30]. OV accounts for about 90% of all ovarian cancers. Despite recent large-scale profiling efforts, the best predictor of OV survival to date has remained the tumor’s stage at diagnosis, a pathological assessment of the spread of the cancer numbering I to IV [31]. About 25% of primary OV tumors are resistant, and most recurrent OV tumors develop resistance to platinum-based chemotherapy, the first-line treatment for more than 30 years now [32]. Even though there exist drugs for platinum-based chemotherapy-resistant OV tumors, no pathology laboratory diagnostic exists that distinguishes between resistant and sensitive tumors before the treatment [33]. OV tumors exhibit significant CNA variation among them, much more so than, e.g., GBM tumors, and very few frequent CNAs typical of OV have been identified so far. We, therefore, model the profiles across each chromosome arm, and each combination of two chromosome arms, separately. The modeling uncovers previously unrecognized chromosome arm-wide patterns of tumor-exclusive and platform-consistent co-occurring CNAs.

By using survival analyses of the discovery and, separately, validation set of patients, as well as only the platinum-based chemotherapy patients in the discovery and validation sets, we find, first, and validate that each of the patterns across only the chromosome arms 7p and Xq, and across only the combination of the two chromosome arms 6p+12p (but not 6p nor 12p separately), is correlated with an OV patient’s prognosis and response to platinum-based chemotherapy, is independent of stage, and together with stage makes a better predictor than stage alone. By using survival analyses of only the > 95% patients with high-grade tumors, we find and validate that these patterns are also independent of the OV tumor’s grade. We observe three groups of significantly different prognoses among the patients classified by a combination of the 6p+12p, 7p, and Xq tensor GSVD classifications, suggesting a possible implementation of the patterns in a pathology laboratory test. Second, by using segmentation of the 6p+12p, 7p, and Xq patterns, we find that the amplifications and deletions identified by these patterns include most known OV-associated CNAs that map to these chromosome arms [34], as well as several previously unreported, yet frequent focal CNAs [35–38]. Third, by using gene ontology enrichment analyses of the OV tumor mRNA expression profiles of the patients [39, 40], we find that differential mRNA expression between the patients, classified by any one of the three tensor GSVDs, is enriched in ontologies corresponding to one of three hallmarks of cancer [41]: a cell’s immortality in 6p+12p, DNA instability in 7p, and cellular immune response suppression in Xq. The differential mRNA expression of genes from these enriched ontologies that are located on any one of the chromosome arms is consistent with the CNAs across that arm. Genes that map to amplifications or deletions on any one pattern, are overexpressed or underexpressed, respectively, in the patients which tumor profiles are classified as highly similar to that pattern. The differential expression of all microRNAs and proteins that map to any one of the chromosome arms is also consistent with the CNAs across that arm.

Taken together, a coherent picture emerges for each of these previously unrecognized chromosome arm-wide patterns of tumor-exclusive and platform-consistent co-occurring alterations, suggesting roles for the DNA CNAs in OV pathogenesis in addition to personalized diagnosis, prognosis, and treatment. In 6p+12p, loss of the p21-encoding *CDKN1A* and the p38-encoding *MAPK14* on 6p, and gain of *KRAS* on 12p, combined but not separately, can lead to transformation of human normal to tumor cells [42, 43]. These transformation-encoding CNAs, together with deletion of *TNF* on 6p, and amplification of *RAD51AP1* and *ITPR2* on 12p, are correlated with a suppression of cell cycle arrest, senescence, and apoptosis, i.e., a tumor cell’s immortality, and a patient’s shorter survival time [44–55]. Note that there already exist drugs that interact with *CDKN1A*, *MAPK14*, and *RAD51AP1*, even though these genes were not recognized previously as targets for OV drug therapy [56]. In 7p, *RPA3* deletion and *POLD2* amplification are correlated with DNA repair during replication, i.e., DNA stability, and a longer survival time [57, 58]. In Xq, *PABPC5* deletion and *BCAP31* amplification are correlated with a cellular immune response, and a longer survival time [59].

## Mathematical Method: Tensor GSVD

### Discovery Datasets are Pairs of Column-Matched but Row-Independent Tensors

We selected primary OV tumor and normal DNA copy-number profiles of a set of 249 TCGA patients [2] (Sec. 1.1 in S1 Appendix, and S1 Dataset). Each profile was measured in two replicates by the same set of two DNA microarray platforms. For each chromosome arm or combination of two chromosome arms, the structure of these tumor and normal discovery datasets 𝒟_1_ and 𝒟_2_, of *K*_1_-tumor and *K*_2_-normal probes × *L*-patients, i.e., arrays × *M*-platforms, is that of two third-order tensors with one-to-one mappings between the column dimensions *L* and *M*, but different row dimensions *K*_1_ and *K*_2_, where *K*_1_, *K*_2_ ≥ *LM*.

### The Tensor GSVD

**Fig 1:**
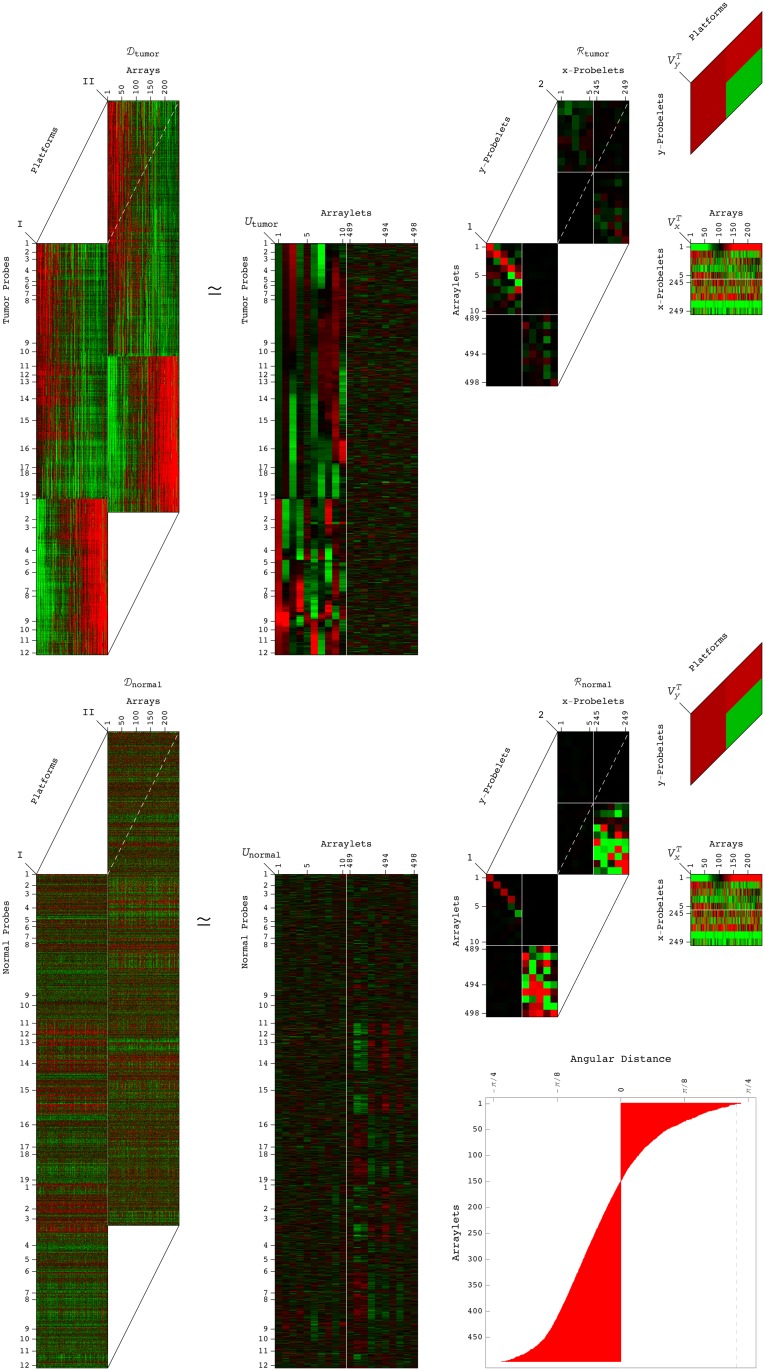
Tensor generalized singular value decomposition (GSVD) of the patient- and platform-matched DNA copy-number profiles of the 6p+12p chromosome arms. For each chromosome arm or combination of two chromosome arms, the structure of the tumor and normal discovery datasets (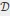
_1_ and 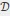
_2_) is that of two third-order tensors with one-to-one mappings between the column dimensions but different row dimensions. The patients, platforms, probes, and tissue types, each represent a degree of freedom. Unfolded into a single matrix, some of the degrees of freedom are lost and much of the information in the datasets might also be lost. We define a tensor GSVD that simultaneously separates the paired datasets into weighted sums of paired subtensors, i.e., combinations or outer products of three patterns each: Either one tumor-specific pattern of copy-number variation across the tumor probes, i.e., a tumor arraylet (a column basis vector of *U*_1_), or the corresponding normal-specific arraylet (a column basis vector of *U*_2_), combined with one pattern of variation across the patients, i.e., an *x*-probelet (a row basis vector of $V_{x}^{T}$), and one pattern across the platforms, i.e., a *y*-probelet (a row basis vector of $V_{y}^{T}$), which are identical for both the tumor and normal datasets (Equation 1). The tensor GSVD is depicted in a raster display, with relative copy-number gain (red), no change (black), and loss (green), explicitly showing the first through the 5th, and the 245th through the 249th 6p+12p *x*-probelets, both 6p+12p *y*-probelets, and the first through the 10th, and the 489th through the 498th 6p+12p tumor and normal arraylets. We prove that the significance of a subtensor in the tumor dataset relative to that of the corresponding subtensor in the normal dataset, i.e., the tensor GSVD angular distance, equals the row mode GSVD angular distance, i.e., the significance of the corresponding tumor arraylet in the tumor dataset relative to that of the normal arraylet in the normal dataset. The tensor GSVD angular distances for the 498 pairs of 6p+12p arraylets are depicted in a bar chart display, where the angular distance corresponding to the first pair of arraylets is ∼ *π*/4. For the 6p+12p combination of two chromosome arms, we find that the most significant subtensor in the tumor dataset (which corresponds to the coefficient of largest magnitude in ℛ_1_) is a combination of (*i*) the first *y*-probelet, which is approximately invariant across the platforms, (*ii*) the first *x*-probelet, which classifies the discovery set of patients into two groups of high and low coefficients, of significantly and robustly different prognoses, and (*iii*) the first, most tumor-exclusive tumor arraylet, which classifies the validation set of patients into two groups of high and low correlations of significantly different prognoses consistent with the *x*-probelet’s classification of the discovery set.

We define, therefore, a novel tensor GSVD that simultaneously separates the paired datasets into weighted sums of *LM* paired “subtensors”, i.e., combinations or outer products of three patterns each: Either one tumor-specific pattern of copy-number variation across the tumor probes, i.e., a “tumor arraylet” *u*_1,*a*_, or the corresponding normal-specific pattern across the normal probes, i.e., the “normal arraylet” *u*_2,*a*_, combined with one pattern of copy-number variation across the patients, i.e., an “*x*-probelet” $v_{x,b}^{T}$ and one pattern across the platforms, i.e., a “*y*-probelet” $v_{y,c}^{T}$, which are identical for both the tumor and normal datasets (Fig. 1, and Figs. A and B in S1 Appendix),

$$\begin{array}{ccc} {𝒟}_{i} & = & {ℛ}_{i}\times_{a}U_{i}\times_{b}V_{x}\times_{c}V_{y}\\ & = & \sum_{a=1}^{LM}\sum_{b=1}^{L}\sum_{c=1}^{M}{ℛ}_{i,abc}{𝒮}_{i}\left(a,b,c\right),\\ {𝒮}_{i}\left(a,b,c\right) & = & u_{i,a}⊗v_{x,b}^{T}⊗v_{y,c}^{T},\;i=1,2,\; \end{array}$$ (1)

where ×_*a*_ *U*_*i*_, ×_*b*_ *V*_*x*_ and ×_*c*_ *V*_*y*_ denote tensor-matrix multiplications, which contract the *LM*-arraylet, *L*-*x*-probelet, and *M*-*y*-probelet dimensions of the “core tensor” ℛ_*i*_ with those of *U*_*i*_, *V*_*x*_, and *V*_*y*_, respectively, and where ⊗ denotes an outer product.

#### Construction

Suppose that unfolding (or matricizing) both tensors 𝒟_*i*_ into matrices, each preserving the *K*_*i*_-row dimension, e.g., by appending the *LM* columns 𝒟_*i*,:*lm*_ of the corresponding tensor, gives two full column-rank matrices *D*_*i*_ ∈ ℝ^*K*_*i*_×*LM*^. We obtain the column bases vectors *U*_*i*_ from the GSVD of *D*_*i*_ [5–13], i.e., the “row mode GSVD”

$$\begin{array}{c} D_{i}=\left(\ldots ,{𝒟}_{i,:lm},\ldots \right)=U_{i}\Sigma_{i}V^{T},\;i=1,2.\; \end{array}$$ (2)

Suppose, similarly, that unfolding both tensors 𝒟_*i*_ into matrices, each preserving the *L*-*x*- (or *M*-*y*-) column dimension, e.g., by appending the *K*_*i*_ *M* rows ${𝒟}_{i,k_{i}:m}^{T}$ (or the *K*_*i*_ *L* rows ${𝒟}_{i,k_{i}l:}^{T}$) of the corresponding tensor, gives two full column-rank matrices *D*_*ix*_ ∈ ℝ^*K*_*i*_*M*×*L*^ (or *D*_*iy*_ ∈ ℝ^*K*_*i*_*L*×*M*^). We obtain the *x*- (or *y*-) row basis vectors $V_{x}^{T}$ (or $V_{y}^{T}$), from the GSVD of *D*_*ix*_ (or *D*_*iy*_), i.e., the *x*- (or *y*-) column mode GSVD,

$$\begin{array}{cccc} D_{ix} & = & \left(\ldots ,{𝒟}_{i,k:m}^{T},\ldots \right) & =U_{ix}\Sigma_{ix}V_{x}^{T},\\ D_{iy} & = & \left(\ldots ,{𝒟}_{i,kl:}^{T},\ldots \right) & =U_{iy}\Sigma_{iy}V_{y}^{T},\;i=1,2.\; \end{array}$$ (3)

Note that the *x*- and *y*-row bases vectors are, in general, non-orthogonal but normalized, and *V*_*x*_ and *V*_*y*_ are invertible. The column bases vectors are normalized and orthogonal, i.e., uncorrelated, such that $U_{i}^{T}U_{i}=I$.

The generalized singular values are positive, and are arranged in Σ_*i*_, Σ_*ix*_, and Σ_*iy*_ in decreasing orders of the corresponding “GSVD angular distances”, i.e., decreasing orders of the ratios *σ*_1,*a*_/*σ*_2,*a*_, *σ*_1*x*,*b*_/*σ*_2*x*,*b*_, and *σ*_1*y*,*c*_/*σ*_2*y*,*c*_, respectively. We then compute the core tensors ℛ_*i*_ by contracting the row-, *x*-, and *y*-column dimensions of the tensors 𝒟_*i*_ with those of the matrices *U*_*i*_, $V_{x}^{-1}$, and $V_{y}^{-1}$, respectively. For real tensors, the “tensor generalized singular values” ℛ_*i*,*abc*_ tabulated in the core tensors are real but not necessarily positive. Our tensor GSVD construction generalizes the GSVD to higher orders in analogy with the generalization of the singular value decomposition (SVD) by the HOSVD [25–28], and is different from other approaches to the decomposition of two tensors [29].

#### Existence, uniqueness and special cases

We prove that our tensor GSVD exists for two tensors of any order because it is constructed from the GSVDs of the tensors unfolded into full column-rank matrices (Lemma A in S1 Appendix). The tensor GSVD has the same uniqueness properties as the GSVD, where the column bases vectors *u*_*i*,*a*_ and the row bases vectors $v_{x,b}^{T}$ and $v_{y,c}^{T}$ are unique, except in degenerate subspaces, defined by subsets of equal generalized singular values *σ*_*i*,*a*_, *σ*_*ix*,*b*_, and *σ*_*iy*,*c*_, respectively, and up to phase factors of ±1, such that each vector captures both parallel and antiparallel patterns (Lemma B in S1 Appendix). The tensor GSVD of two second-order tensors reduces to the GSVD of the corresponding matrices (Corollary A in S1 Appendix). The tensor GSVD of the tensor 𝒟_1_ ∈ ℝ^*LM*×*L*×*M*^, which row mode unfolding gives the identity matrix *D*_1_ = *I* ∈ ℝ^*LM*×*LM*^, and a tensor 𝒟_2_ of the same column dimensions reduces to the HOSVD of 𝒟_2_ (Theorem A in S1 Appendix).

#### Interpretation

The significance of the subtensor 𝒮_*i*_(*a*,*b*,*c*) in the tensor 𝒟_*i*_ is defined proportional to the magnitude of the corresponding tensor generalized singular values ℛ_*i*,*abc*_ (Fig. C in S1 Appendix), in analogy with the HOSVD,

$$\begin{array}{c} {𝒫}_{i,abc}={ℛ}_{i,abc}^{2}/\sum_{a=1}^{LM}\sum_{b=1}^{L}\sum_{c=1}^{M}{ℛ}_{i,abc}^{2},\;i=1,2.\; \end{array}$$ (4)

The significance of 𝒮_1_(*a*,*b*,*c*) in 𝒟_1_ relative to that of 𝒮_2_(*a*,*b*,*c*) in 𝒟_2_ is defined by the “tensor GSVD angular distance” Θ_*abc*_ as a function of the ratio ℛ_1,*abc*_/ℛ_2,*abc*_. This is in analogy with, e.g., the row mode GSVD angular distance *θ*_*a*_, which defines the significance of the column basis vector *u*_1,*a*_ in the matrix *D*_1_ of Equation (2) relative to that of *u*_2,*a*_ in *D*_2_ as a function of the ratio *σ*_1,*a*_/*σ*_2,*a*_,

$$\begin{array}{ccc} \Theta_{abc} & = & \arctan({ℛ}_{1,abc}/{ℛ}_{2,abc})-\pi /4,\\ \theta_{a} & = & \arctan(\sigma_{1,a}/\sigma_{2,a})-\pi /4. \end{array}$$ (5)

Because the ratios of the positive generalized singular values satisfy *σ*_1,*a*_/*σ*_2,*a*_ ∈ [0, ∞), the row mode GSVD angular distances satisfy *θ*_*a*_ ∈ [−*π*/4, *π*/4]. The maximum (or minimum) angular distance, i.e., *θ*_*a*_ = *π*/4, which corresponds to *σ*_1,*a*_/*σ*_2,*a*_ > > 1 (or −*π*/4, which corresponds to *σ*_1,*a*_/*σ*_2,*a*_ < < 1), indicates that the row basis vector $v_{a}^{T}$ of Equation (2), which corresponds to the column basis vectors *u*_1,*a*_ in *D*_1_ and *u*_2,*a*_ in *D*_2_, is exclusive to *D*_1_ (or *D*_2_). An angular distance of *θ*_*a*_ = 0, which corresponds to *σ*_1,*a*_/*σ*_2,*a*_ = 1, indicates a row basis vector $v_{a}^{T}$ which is of equal significance in, i.e., common to both *D*_1_ and *D*_2_.

Thus, while the ratio *σ*_1,*a*_/*σ*_2,*a*_ indicates the significance of *u*_1,*a*_ in *D*_1_ relative to the significance of *u*_2,*a*_ in *D*_2_, this relative significance is defined, as previously described [12, 13], by the angular distance *θ*_*a*_, a function of the ratio *σ*_1,*a*_/*σ*_2,*a*_, which is antisymmetric in *D*_1_ and *D*_2_. Note also that while other functions of the ratio *σ*_1,*a*_/*σ*_2,*a*_ exist that are antisymmetric in *D*_1_ and *D*_2_, the angular distance *θ*_*a*_, which is a function of the arctangent of the ratio, i.e., arctan(*σ*_1,*a*_/*σ*_2,*a*_), is the natural function to use, because the GSVD is related to the cosine-sine (CS) decomposition, as previously described [9], and, thus, *σ*_1,*a*_ and *σ*_2,*a*_ are related to the sine and the cosine functions of the angle *θ*_*a*_, respectively.

*Theorem 1. The tensor GSVD angular distance equals the row mode GSVD angular distance, i.e., Θ_*abc*_ = θ_*a*_*.

*Proof*. The unfolding of 𝒟_*i*_ of Equation (1) into *D*_*i*_ of Equation (2) unfolds the core tensors ℛ_*i*_ of Equation (1) into matrices *R*_*i*_, which preserve the row dimensions, i.e., the *LM*-column bases dimensions of ℛ_*i*_, and gives

$$\begin{array}{ccc} D_{i} & = & U_{i}R_{i}\left(V_{x}^{T}⊗V_{y}^{T}\right),\\ R_{i} & = & \Sigma_{i}V^{T}\left(V_{x}^{-T}⊗V_{y}^{-T}\right),\;i=1,2,\; \end{array}$$ (6)

where ⊗ denotes a Kronecker product. Because Σ_*i*_ are positive diagonal matrices, it follows that ℛ_1,*abc*_/ℛ_2,*abc*_ = *R*_1,*a*_/*R*_2,*a*_ = *σ*_1,*a*_/*σ*_2,*a*_. Substituting this in Equation (5) gives Θ_*abc*_ = *θ*_*a*_. Note that the proof holds for tensors of higher-than-third order.

From this it follows that the tensor GSVD angular distance ∣Θ_*abc*_∣ ≤ *π*/4, and that, therefore, the ratio of the tensor generalized singular values ℛ_1,*abc*_/ℛ_2,*abc*_ > 0, even though ℛ_1,*abc*_ and ℛ_2,*abc*_ are not necessarily positive. It also follows that Θ_*abc*_ = ±*π*/4 indicate a subtensor exclusive to either 𝒟_1_ or 𝒟_2_, respectively, and that Θ_*abc*_ = 0 indicates a subtensor common to both.

Note that since the generalized singular values are arranged in Σ_*i*_ of Equation (2) in a decreasing order of the row mode GSVD angular distances *θ*_*a*_, the most tumor-exclusive tumor subtensors, i.e., 𝒮_1_(*a*,*b*,*c*) where *a* maximizes *θ*_*a*_ of Equation (5), correspond to *a* = 1, whereas the most normal-exclusive normal subtensors, i.e., 𝒮_2_(*a*,*b*,*c*) where *a* minimizes *θ*_*a*_, correspond to *a* = *LM*.

### Discovery and Validation of CNAs Predicting OV Survival

We compute the tensor GSVD of the tumor and normal discovery datasets for each chromosome arm and each combination of two chromosome arms, separately (S1 Mathematica Notebook). For each arm or arms we examine the most significant subtensor in the tumor dataset, i.e., 𝒮_1_(*a*,*b*,*c*), where *a*, *b*, and *c* maximize 𝒫_1,*abc*_ of Equation (4).

**Fig 2:**
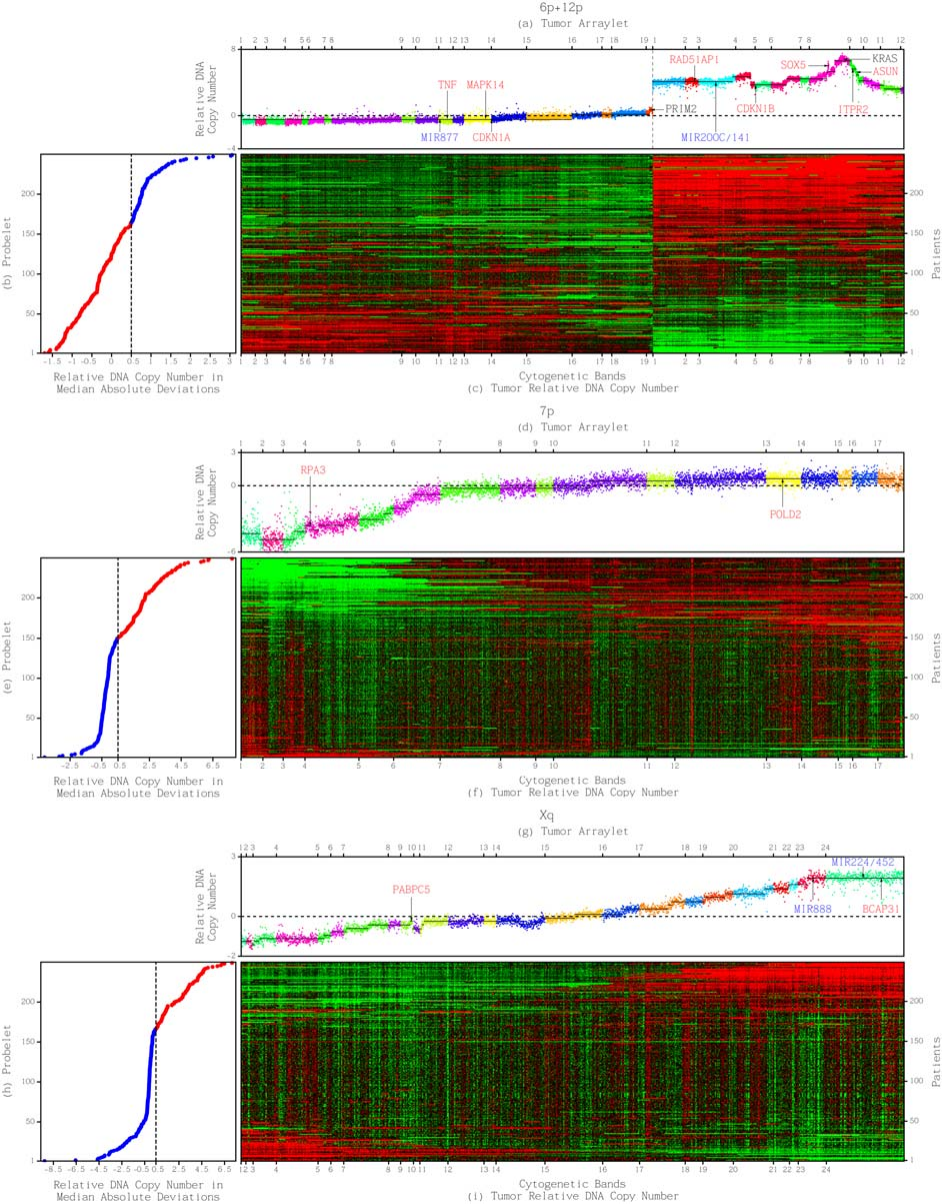
Tumor-exclusive and platform-consistent DNA copy-number alterations (CNAs) correlated with ovarian serous cystadenocarcinoma (OV) patients’ survival. (*a*) Plot of the first 6p+12p tumor arraylet describes a pattern of tumor-exclusive and platform-consistent co-occurring CNAs across the combination of the two chromosome arms 6p+12p. The probes are ordered, and their copy numbers are colored according to each probe’s chromosomal band location. Segments (black lines) amplified and deleted include most known OV-associated CNAs that map to 6p+12p (black), including an amplification of *KRAS* and a deletion of *PRIM2*. CNAs previously unrecognized in OV (red) include a deletion of the p38-encoding *MAPK14*, and p21-encoding *CDKN1A*, and an amplification of *RAD51AP1*, a deletion of *TNF*, and focal amplifications of *ASUN*, *ITPR2*, and the 5’ ends of isoforms a and e, and exons 5 and 6 of *SOX5*. A high 6p+12p arraylet correlation is significantly correlated with a patient’s shorter survival time. (*b*) Plot of the first 6p+12p *x*-probelet describes the classification of the discovery set of patients into two groups of high (blue) and low (red) coefficients. A high 6p+12p *x*-probelet coefficient is significantly and robustly correlated with a patient’s shorter survival time. (*c*) Raster display of the 6p+12p tumor profiles, where medians of the profiles of the same patient measured by the two platforms were taken, with relative gain (red), no change (black), and loss (green) of DNA copy numbers. (*d*) Plot of the first 7p tumor arraylet describes a pattern of CNAs across the chromosome arm 7p. CNAs previously unrecognized in OV (red) include a focal deletion of *RPA3* and an amplification of *POLD2*. A high 7p arraylet correlation is significantly correlated with a patient’s longer survival time. (*e*) Plot of the first 7p *x*-probelet describes the classification of the discovery set of patients into two groups of high (red) and low (blue) coefficients. A high 7p *x*-probelet coefficient is significantly and robustly correlated with a patient’s longer survival time. (*f*) Raster display of the 7p tumor profiles. (*g*) Plot of the first Xq tumor arraylet. CNAs previously unrecognized in OV (red) include a focal deletion of *PABPC5* and an amplification of *BCAP31*. A high Xq arraylet correlation is significantly correlated with a patient’s longer survival time. (*h*) Plot of the first Xq *x*-probelet describes the classification of the discovery set of patients into two groups of high (red) and low (blue) coefficients. A high Xq *x*-probelet coefficient is significantly and robustly correlated with a patient’s longer survival time. (*i*) Raster display of the Xq tumor profiles.

**Fig 3:**
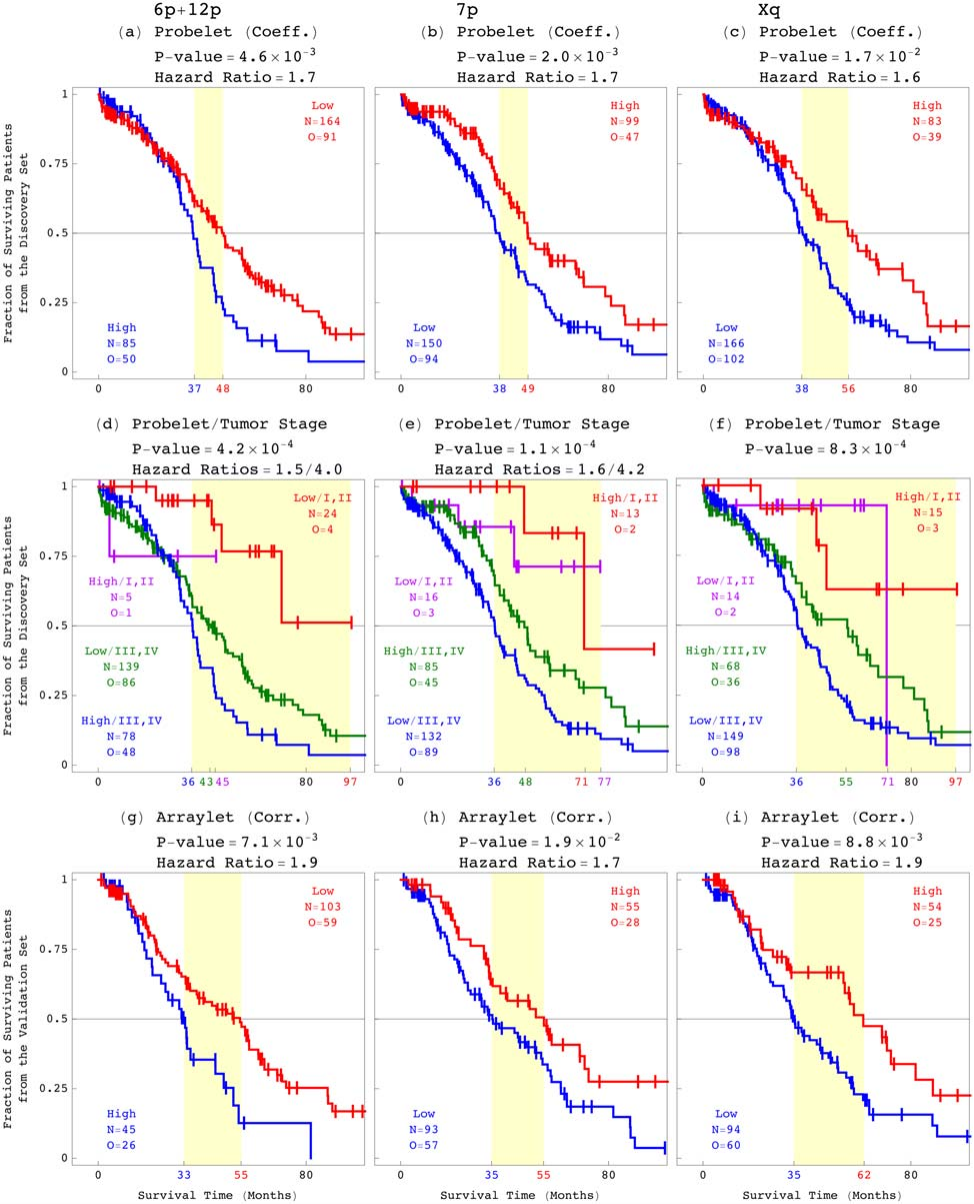
Survival analyses of the discovery and validation sets of patients classified by tensor GSVD, or tensor GSVD and tumor stage at diagnosis. (*a*) Kaplan-Meier (KM) curves of the discovery set of 249 patients classified by the 6p+12p *x*-probelet coefficient, show a median survival time difference of 11 months, with the corresponding log-rank test *P*-value < 10^−2^. The univariate Cox proportional hazard ratio is 1.7. (*b*) Survival analyses of the 249 patients classified by the 7p *x*-probelet coefficient. (*c*) The 249 patients classified by the Xq *x*-probelet coefficient. (*d*) The 249 patients classified by both the 6p+12p tensor GSVD and tumor stage at diagnosis, show the bivariate Cox hazard ratios of 1.5 and 4.0, which do not differ significantly from the corresponding univariate hazard ratios of 1.7 and 4.4, respectively. This means that the 6p+12p tensor GSVD is independent of stage, the best predictor of OV survival to date. The 61 months KM median survival time difference is about 85% and more than two years greater than the 33 month difference between the patients classified by stage alone. This means that the tensor GSVD and stage combined make a better predictor than stage alone. (*e*) The 249 patients classified by both the 7p tensor GSVD and stage. (*f*) The 249 patients classified by both the Xq tensor GSVD and stage. (*g*) KM curves of the validation set of 148 stage III-IV patients classified by the 6p+12p arraylet correlation, show a median survival time difference of 22 months, with the corresponding log-rank test *P*-value < 10^−2^, and the univariate Cox proportional hazard ratio 1.9. This validates the survival analyses of the discovery set of 249 patients. (*h*) Survival analyses of the 148 patients classified by the 7p arraylet correlation. (*i*) The 148 patients classified by the Xq arraylet correlation.

We, first, require the subtensor to be tumor-exclusive and platform-consistent: include the tumor arraylet *u*_1,*a*_ that is the most exclusive to the tumor dataset, i.e., *u*_1,1_, as well as a *y*-probelet $v_{y,c}^{T}$ of consistent, i.e., approximately equal copy numbers in both platforms. Second, we require the subtensor to be correlated with an OV patient’s prognosis in the discovery set of patients, i.e., include an *x*-probelet $v_{x,b}^{T}$ that classifies the discovery set of patients into two groups of high (> 0.5 standardized median absolute deviation, i.e., sMAD, from the median) and low coefficients, of significantly (log-rank test *P*-value < 0.05) and robustly (throughout the range of ±0.1 sMAD around the cutoff) different prognoses (Fig. 2). Third, we require the subtensor to be correlated with prognosis in the validation set of patients, i.e., include an arraylet that classifies the validation set of patients into two groups of high and low Spearman’s rank correlation coefficients of significantly different prognoses, consistent with the *x*-probelet’s classification of the discovery set of patients (Fig. 3, and Sec. 1.3 in S1 Appendix). Note that the validation set includes 148 TCGA patients, mutually exclusive of the discovery set, with primary OV tumor profiles measured by at least one of the two DNA microarray platforms that were used to measure the discovery datasets (S2 Dataset).

We find that each of the tensor GSVDs of only the chromosome arms 7p and Xq, and only the combination of the two chromosome arms 6p+12p (but not 6p nor 12p separately), uncovers a pattern of tumor-exclusive and platform-consistent co-occurring CNAs that is correlated with an OV patient’s prognosis in the discovery and, separately, validation set of patients.

## Biological Results

### Independent Chromosome Arm-Wide Predictors of OV Survival and Response to Platinum-Based Chemotherapy

To date, the best predictor of OV survival has remained the tumor’s stage at diagnosis [31] (Sec. 2.1, and Figs. D and E in S1 Appendix). Additional indicators, such as the residual disease after surgery, the outcome of subsequent therapy, and the neoplasm status, which is the last known status of the disease, are determined during treatment. No diagnostic exists that distinguishes between platinum-based chemotherapy-resistant and -sensitive tumors before the treatment [32, 33].

We find and validate, by using survival analyses of the discovery and, separately, validation set of patients, as well as only the 88% and 95% platinum-based chemotherapy patients in the discovery and validation sets, respectively (Fig. F in S1 Appendix), that each of the patterns, across 6p+12, 7p, and Xq, is correlated with an OV patient’s prognosis and response to platinum-based chemotherapy, is independent of stage, and together with stage makes a better predictor than stage alone.

We also find and validate that each of these three tensor GSVDs is independent of each of the additional standard indicators (Tables A and B in S1 Appendix). For example, survival analyses of the discovery set classified by the 6p+12p tensor GSVD into high and low *x*-probelet coefficients, and by pathology at diagnosis into tumor stages I-II and III-IV, give the bivariate Cox hazard ratios of 1.5 and 4.0, which are similar to the corresponding univariate ratios of 1.7 and 4.4, respectively [18]. Similarly, survival analyses of the validation set classified by the 6p+12p tensor GSVD into high and low arraylet correlation coefficients, and by pathology at diagnosis into tumor stages III and IV, give the bivariate Cox hazard ratios of 1.9 and 1.8, which are the same as the corresponding univariate ratios (Fig. G in S1 Appendix). This means that the 6p+12p tensor GSVD and stage are independent predictors of survival. Therefore, combined with any one of the standard indicators, each of the three tensor GSVDs makes a better predictor than the standard indicator alone (Figs. H and I in S1 Appendix). For example, the Kaplan-Meier (KM) median survival time difference of 61 months among the discovery set of patients classified by both the 6p+12p tensor GSVD and stage, is about 85% and more than two years greater than the 33 month difference between the patients classified by stage alone [19]. The KM median survival difference of 34 months among the validation set of patients classified by both the 6p+12p tensor GSVD and stage, is about 62% and more than one year greater than the 21 month difference between the patients classified by stage alone.

Note that while the discovery set of patients reflects the general OV patient population, with approximately 5%, 7%, 76%, and 12% of the patients diagnosed at stages I, II, III, and IV, respectively, the validation set reflects the high-stage OV patient population, with approximately 20% and 80% of the patients diagnosed at stages III and IV, respectively. The 6p+12p, 7p, and Xq tensor GSVDs, therefore, predict survival both in the general as well as in the high-stage OV patient population. Note also that the discovery and validation sets each include mostly, i.e., > 95% high-grade, i.e., grades 2 and higher tumors. Tumor grade does not correlate with survival in either the discovery or the validation set of patients. Survival analyses of only the > 95% patients with high-grade tumors in the discovery and, separately, validation set give qualitatively the same and quantitatively similar results to those of the analyses of 100% of the patients in each set, respectively. The 6p+12p, 7p, and Xq tensor GSVDs, therefore, predict survival in the high-grade OV patient population, and are independent of the OV tumor’s grade as well as the molecular distinctions between high- and low-grade OV tumors [30].

**Fig 4:**
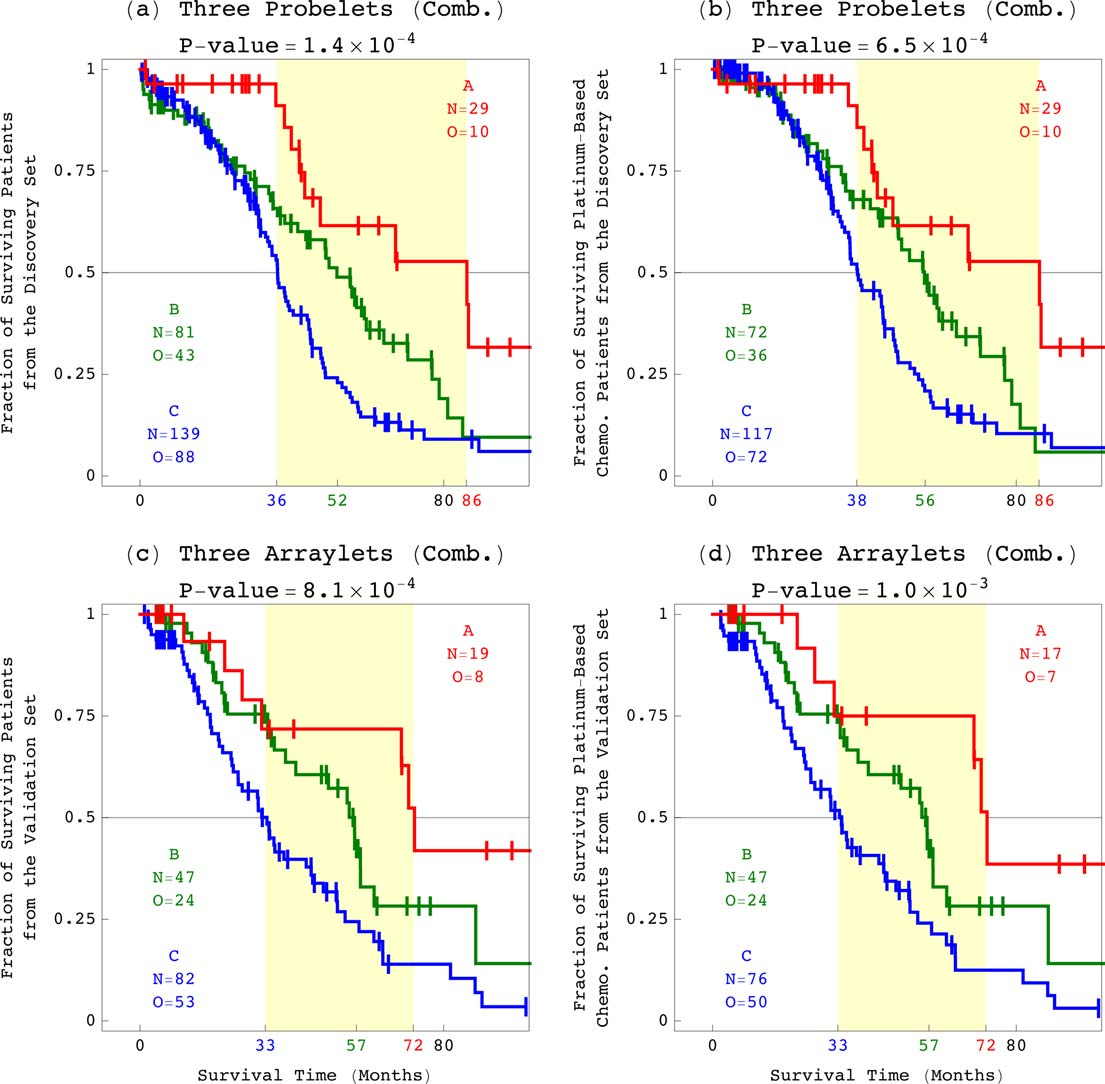
Survival analyses of the discovery and validation sets of patients, as well as only the platinum-based chemotherapy patients in the discovery and validation sets, classified by the 6p+12p, 7p, and Xq tensor GSVD combined. (*a*) KM curves of the discovery set of 249 patients classified by combination of the 6p+12p, 7p, and Xq *x*-probelet coefficients, show median survival times of 86, 52, and 36 months for the groups A, B, and C, respectively, with the corresponding log-rank test *P*-value < 10^−3^. (*b*) KM survival analysis of only the 218, i.e., ∼ 88% platinum-based chemotherapy patients in the discovery set, classified by combination of the three tensor GSVDs, gives qualitatively the same and quantitatively similar results to those of the analyses of 100% of the patients. This means that the combination of the three tensor GSVDs predicts survival in the platinum-based chemotherapy patient population. (*c*) KM curves of the validation set of 148 stage III-IV patients classified by combination of the 6p+12p, 7p, and Xq arraylet correlation coefficients, show median survival times of 72, 57, and 33 months for the groups A, B, and C, respectively, with the corresponding log-rank test *P*-value < 10^−3^. This validates the survival analyses of the discovery set of 249 patients. (*d*) KM survival analysis of only the 140, i.e., ∼ 95% platinum-based chemotherapy patients in the validation set, classified by combination of the three tensor GSVDs.

We observe three groups of significantly different prognoses among the discovery and, separately, validation set of patients, as well as only the platinum-based chemotherapy patients, classified by a combination of the three, i.e., 6p+12p, 7p, and Xq, tensor GSVD classifications, each of which is binomial (Fig. 4). In group A, a combination of a low 6p+12p *x*-probelet coefficient or arraylet correlation, and high 7p and Xq *x*-probelet coefficients or arraylet correlations is indicative of a patient’s significantly longer survival time and better response to platinum-based chemotherapy. In group B, the three combinations where just one of the three binomial classifications differs from that of group A, indicate shorter survival time and worse response to chemotherapy than those of group A. In group C, the four combinations where at least two of the three binomial classifications differ from that of group A, indicate shorter survival time and worse response to chemotherapy than those of group B as well as group A. For example, the KM median survival times of the discovery set of patients classified into groups A, B, and C are 86, 52, and 36 months, such that the median survival time of group A is more than four years greater than, and more than twice that of group C.

This suggests a possible implementation of the 6p+12p, 7p, and Xq patterns in a pathology laboratory test, where a patient’s survival and response to platinum-based chemotherapy is predicted based upon the combination of the correlations of the OV tumor’s DNA copy-number profile with the 6p+12p, 7p, and Xq patterns.

### Novel Frequent Focal CNAs Indicating Survival

OV tumors exhibit significant CNA variation among them, much more so than, e.g., GBM brain tumors [2, 13]. Very few frequently occurring OV CNAs have been identified to date.

We find, by using segmentation [20, 21], that the three tensor GSVD arraylets include most known OV-associated CNAs that map to the corresponding chromosome arms, and several previously unreported yet frequent CNAs in > 23% of the patients. For example, the 6p+12p arraylet includes two segments corresponding to the only known OV focal CNAs that map to 6p+12p, 7p, or Xq (Sec. 2.2 in S1 Appendix). One, a deletion (6p11.2), overlaps the 3’ end unique to isoform a of the DNA primase polypeptide 2-encoding *PRIM2* [2]. The other, an amplification (12p12.1-p11.23), contains several genes, including the Kirsten rat sarcoma viral oncogene homolog *KRAS*, one of three human Ras genes, and the 5’ ends of isoforms b and d of the *SRY* (sex determining region Y)-box 5-encoding *SOX5* [34], and is significantly (log-rank test *P*-value < 0.05, and KM median survival time difference ≥ 12 months) correlated with OV survival (S3 Dataset).

We also find that the three arraylet patterns include novel frequent focal CNAs (segments < 125 probes). Among these, four amplifications and two deletions are significantly correlated with OV survival (Fig. J in S1 Appendix). The amplifications flank the segment that contains *KRAS*. Two consecutive segments (12p12.1) contain the 5’ ends of isoforms a and e of *SOX5*, and exons 5 and 6, the first exons that are common to isoforms a, b, d, and e of *SOX5* [35]. Two other consecutive segments (12p11.23) contain the inositol 1,4,5-trisphosphate receptor type 2-encoding *ITPR2*, and the asunder spermatogenesis regulator-encoding *ASUN*. *ASUN* was discovered in a screen of expressed sequence tags on 12p11-p12, which DNA amplification correlated with mRNA overexpression in four human testicular seminomas and one ovarian papillary serous adenocarcinoma cell line, exemplifying human germ cell tumors [36]. *ASUN* and its homologs are essential for nuclear division after DNA replication in the HeLa human cervical cancer cell line, the frog, and the fly [37]. One deletion (7p22.1-p21.3) contains the replication protein A3-encoding *RPA3*. The other (Xq21.31) contains the cytoplasmic poly(A)-binding protein 5-encoding *PABPC5*, and the sequence tag site DX214 adjacent to translocation breakpoints observed in premature ovarian failure [38].

### Possible Roles in OV Pathogenesis

We find, by using gene ontology enrichment analyses of the OV tumor mRNA expression profiles of the patients [39, 40], that differential mRNA expression between the patients, classified by any one of the three tensor GSVDs, is enriched in ontologies corresponding to one of three hallmarks of cancer [41]: cell immortality in 6p+12p, DNA instability in 7p, and cellular immune response suppression in Xq.

The differential mRNA expression of genes from these enriched ontologies that are located on any one of the chromosome arms is consistent with the CNAs across that arm (Fig. K in S1 Appendix, and S4 Dataset). Genes that map to amplifications or deletions on any one arraylet pattern, are overexpressed or underexpressed, respectively, in the patients which tumor profiles are classified, by the corresponding tensor GSVD, as highly similar to that pattern, i.e., patients of high *x*-probelet coefficients or arraylet correlations. The differential expression of all microRNAs and proteins that map to any one of the chromosome arms is also consistent with the CNAs across that arm (Sec. 2.3, and Figs. L and M in S1 Appendix, and S5 and S6 Datasets). A coherent picture emerges for each pattern, suggesting roles for the CNAs in OV pathogenesis in addition to personalized diagnosis, prognosis, and treatment.

#### 6p+12p. A cell’s transformation and immortality are correlated with a patient’s shorter survival

The genes, which are significantly (Mann-Whitney-Wilcoxon *P*-values < 0.05) differentially expressed between the 6p+12p tensor GSVD classes, i.e., in the patient group of high 6p+12p *x*-probelet coefficient or arraylet correlation, relative to the patient group of low coefficient or correlation, are enriched (hypergeometric *P*-values < 10^−3^) in the ontologies of cellular response to ionizing radiation (GO:0071479), and major histocompatibility (MHC) protein complex (GO:0042611). Most of the GO:0071479 genes are underexpressed, including the p21 cyclin-dependent kinase inhibitor-encoding *CDKN1A*, and the p38 mitogen-activated protein kinase-encoding *MAPK14*, which map to a deletion > 45 Mbp on the telomeric part of 6p (6p25.3-p21.1). Also underexpressed is p38, the protein encoded by *MAPK14*. All GO:0042611 genes, including the tumor necrosis factor-encoding *TNF*, are underexpressed, and map to the same deletion. The one microRNA that is significantly differentially expressed between the 6p+12p tensor GSVD classes, and maps to the same deletion, is the splicing-dependent microRNA miR-877*, which is encoded by the 13th intron of the ATP-binding cassette subfamily F member 1-encoding gene *ABCF1* [44]. Both miR-877* and *ABCF1* are consistently underexpressed.

One of only two GO:0071479 overexpressed genes is the *RAD51*-associated protein 1-encoding *RAD51AP1*, which maps to an amplification > 9 Mbp on the telomeric part of 12p (12p13.33-p13.31) that is significantly correlated with OV survival. All four microRNAs that are differentially expressed between the 6p+12p tensor GSVD classes, and map to the same amplification, miR-200c, miR-200c*, miR-141, and miR-141*, are consistently overexpressed. The second protein that is significantly differentially expressed between the 6p+12p tensor GSVD classes is p27. Consistently, the cyclin-dependent kinase inhibitor *CDKN1B*, which encodes p27, maps to a 4.5 Mbp amplification (12p13.2-p12.3) that is significantly correlated with OV survival, and its mRNA is overexpressed. The mRNA encoded by *KRAS* is also overexpressed.

Note that while the 6p+12p pattern of CNAs is correlated with survival in the discovery and, separately, validation sets, neither the 6p nor the 12p pattern alone are correlated with survival. Indeed, experiments studying the conditions for the transformation of human normal to tumor cells indicate that cells, where both p21 and p38 are inactive, are susceptible to Ras-mediated transformation [42, 43]. However, the activation of Ras alone induces tumor-suppressing cellular senescence via the activities of either p21 or p38. The 6p+12p pattern, therefore, which includes the loss of the p21-encoding *CDKN1A* and the p38-encoding *MAPK14* on 6p, and the gain of *KRAS* on 12p, encodes for cellular conditions that combined but not separately can lead to transformation.

In addition, p21 and p38 are necessary for p53-mediated cell cycle arrest [45] and apoptosis [46], respectively, in response to DNA damage. Overexpression of the p21-encoding *CDKN1A* is correlated with a low malignant potential of an ovarian tumor [47]. *RAD51AP1* overexpression disrupts cell cycle arrest and apoptosis, can lead to cellular resistance to DNA-damaging cancer therapies, such as platinum-based chemotherapy, and may increase DNA instability [48]. *TNF*-induced apoptosis is correlated with downregulation of *ITPR2* [49]. Overexpression of miR-200c, and miR-141, both of which putatively target the *BRCA1* associated protein-1 oncosuppressor-encoding *BAP1*, is correlated with OV tumor growth, dedifferentiation, and invasiveness [50, 51]. Overexpression of the *CDKN1B*-encoded p27, which can promote cellular migration [52] and even proliferation [53], is correlated with a poor OV patient’s prognosis [54, 55].

Taken together, previously unrecognized co-occurring deletion of *CDKN1A* and *MAPK14* on 6p and amplification of *KRAS* on 12p, which encode for human cell transformation, together with deletion of *TNF* on 6p, and amplification of *RAD51AP1* and *ITPR2* on 12p, are correlated with a suppression of cell cycle arrest, senescence, and apoptosis, i.e., a tumor cell’s immortality, and a patient’s shorter survival time. Note that there already exist drugs that interact with *CDKN1A*, *MAPK14*, and *RAD51AP1*, even though these genes were not recognized previously as targets for OV drug therapy [56].

#### 7p. A cell’s DNA stability is correlated with a longer survival

The genes that are significantly differentially expressed between the 7p tensor GSVD classes are enriched (hypergeometric *P*-value < 10^−10^) in the ontology of DNA strand elongation involved in DNA replication (GO:0006271). Most of these genes are overexpressed, including the DNA polymerase delta subunit 2-encoding *POLD2* that is essential for DNA replication and repair, which maps to an amplification > 17 Mbp on the centromeric part of 7p (7p14.1-p11.2). Only two genes are underexpressed: *RPA3* on 7p and the DNA ligase IV-encoding *LIG4* on 13q. The interaction of p53 with the *RPA3*-encoded protein mediates suppression of homologous recombination (HR), the preferred cellular mechanism for DNA double-strand break (DSB) repair during replication [57]. *LIG4* is essential for DSB repair via the more error-prone nonhomologous end joining pathway [58]. HR defects are thought to facilitate the significant CNA heterogeneity among OV tumors [2].

Taken together, previously unrecognized co-occurring deletion and underexpression of *RPA3*, and amplification and overexpression of *POLD2* on 7p are correlated with DNA DSB repair via HR during replication, i.e., DNA stability, and a longer survival time.

#### Xq. Cellular immune response is correlated with a longer survival

The genes that are differentially expressed between the Xq tensor GSVD classes are enriched (hypergeometric *P*-value < 10^−6^) in the ontology of antigen processing and presentation of peptide antigen (GO:0048002). Most of these genes are overexpressed, including the B-cell receptor-associated protein 31-encoding *BCAP31*, which maps to an amplification > 11 Mbp on the telomeric part of Xq (Xq27.3-q28). All three microRNAs that are differentially expressed between the Xq tensor GSVD classes, and map to the same amplification, miR-888, miR-224, and miR-452, together with the gamma-aminobutyric acid (GABA) A receptor epsilon-encoding *GABRE*, which hosts mir-224 and mir-452 in its introns, are consistently overexpressed. Underexpression of miR-224 was implicated in OV pathogenesis [50]. *PABPC5*, which maps to a focal deletion on Xq, is suppressed upon viral infection [59].

Taken together, previously unrecognized co-occurring deletion of *PABPC5*, and amplification and overexpression of *BCAP31* on Xq are correlated with a cellular immune response, and a longer survival time.

## Discussion

We defined a novel tensor GSVD, an exact simultaneous decomposition of two datasets, arranged in two higher-than-second-order tensors of matched column dimensions but independent row dimensions. We showed that the mathematical properties of the tensor GSVD allow interpreting its variables and operations in terms of the similar as well as dissimilar, e.g., biomedical reality between the datasets. We demonstrated the tensor GSVD in comparative modeling of patient- and platform-matched but probe-independent OV tumor and normal DNA copy-number profiles from TCGA. The modeling resulted in new insights into the poorly understood relations between an OV tumor’s genome and a patient’s survival phenotype. Three previously unrecognized chromosome arm-wide patterns of tumor-exclusive and platform-consistent co-occurring alterations were uncovered, across 6p+12p, 7p, and Xq, that are correlated with an OV patient’s survival and response to platinum-based chemotherapy, and are of possible roles in OV pathogenesis, and of a possible implementation in a pathology laboratory test for personalized OV diagnosis, prognosis, and treatment.

Note that unlike previous analyses of the TCGA OV DNA copy-number data, notably by TCGA [2], our analyses were not limited to the 22 human autosomal chromosomes, and include the X chromosome. This is because the tensor GSVD, like the GSVD, comparatively—based upon the structure of the data—separates the matched datasets into uncorrelated, i.e., orthogonal patterns across the tumor and normal probes. Patterns of copy-number variation across the tumor probes that occur in the normal human genome, and are common to the tumor and normal datasets, such as the female-specific X chromosome amplification, are orthogonal to, and, therefore, are separated from the patterns that are exclusive to the tumor dataset. For example, the GSVD comparative modeling of patient-matched GBM tumor and normal copy-number profiles separated the prognosis-correlated GBM tumor-exclusive pattern from the female-specific X chromosome amplification as well as from experimental artifacts (or batch effects) due to experimental variations in, e.g., tissue batch, genomic center, hybridization date, and scanner, without a-priori knowledge of these variations.

Unlike recent approaches to the integrative modeling of different types of large-scale molecular biological profiles from the same set of patients, notably clustering [60, 61], our comparative modeling was not limited to tumor profiles, and included also patient- and platform-matched normal DNA copy-number profiles. This is because the tensor GSVD, like the GSVD, finds not just the similarities but, at the same time also the dissimilarities among the profiles without making any assumptions, except for the structure of the data: two third-order tensors, of matched columns that correspond to the same sets of patients and platforms, and independent rows that correspond to the probes in either the tumor or the normal dataset. The patients, platforms, tumor and normal probes as well as the tissue types, each represent a degree of freedom. Unfolded into two matrices or appended into a single tensor (or even unfolded and appended into a single matrix), some of the degrees of freedom are lost and much of the information in the datasets might also be lost. For example, SVD of the GBM tumor and normal profiles appended into a single matrix, while it is related to the GSVD of the data, would not separate the tumor dataset into patterns across the tumor probes that are orthogonal.

Additional possible applications of the tensor GSVD in personalized medicine include comparative modeling of two patient- and tissue-matched datasets, each corresponding to (*i*) a set of large-scale molecular biological profiles, e.g., DNA copy numbers, acquired by a high-throughput technology, e.g., DNA microarrays; (*ii*) a set of biomedical images or signals; or (*iii*) a set of cellular pathological observations, e.g., a tumor’s stage. Such tensor GSVD comparative models can uncover variations across the patients and tissues that are common to, possibly causally coordinated between the two aspects of the disease. In clinical settings, such tensor GSVD comparative models can determine an individual patient’s medical status in relation to all the other patients in a set, and inform the patient’s diagnosis, prognosis and treatment.

## Supporting Information

S1 Appendix A PDF format file, readable by Adobe Acrobat Reader. (PDF)

S1 Mathematica Notebook Tensor GSVD of patient- and platform-matched tumor and normal genomic profiles. A PDF format file, readable by Adobe Acrobat Reader. The corresponding Mathematica 9.0.1 code file, executable by Mathematica and readable by Mathematica Player, is available at http://www.alterlab.org/OV_prognosis/.
(PDF)

S1 Dataset Discovery Set of Patients. A tab-delimited text format file, readable by both Mathematica and Microsoft Excel, reproducing TCGA annotations of the discovery set of 249 patients. The tumor and normal profiles of the discovery set of patients measured by each of the two DNA microarray platforms, tabulating relative copy-number variation across the 6p+12p, 7p, and Xq tumor and normal probes, are available in tab-delimited text format files at http://www.alterlab.org/OV_prognosis/.
(TXT)

S2 Dataset Validation Set of Patients. A tab-delimited text format file reproducing TCGA annotations of the validation set of 148 patients. The tumor profiles of the validation set of patients, tabulating relative copy-number variation across the 6p+12p, 7p, and Xq tumor probes, are available in tab-delimited text format files at http://www.alterlab.org/OV_prognosis/.
(TXT)

S3 Dataset First, Most Tumor-Exclusive Tumor Arraylets. A tab-delimited text format file tabulating the segments of the first, most tumor-exclusive tumor arraylets computed by tensor GSVD of the discovery set of patients across 6p+12p, 7p, or Xq.
(TXT)

S4 Dataset Differential mRNA Expression. A tab-delimited text format file tabulating differential expression of 11,457 autosomal and X chromosome mRNAs in the 6p+12p, 7p, and Xq tensor GSVD classes. The mRNA expression profiles of 394 of the 397 patients in the discovery and validation sets are available in tab-delimited text format files at http://www.alterlab.org/OV_prognosis/.
(TXT)

S5 Dataset Differential microRNA Expression. A tab-delimited text format file tabulating differential expression of 639 autosomal and X chromosome microRNAs in the 6p+12p, 7p, and Xq tensor GSVD classes. The microRNA expression profiles of 395 patients are available in tab-delimited text format files at http://www.alterlab.org/OV_prognosis/.
(TXT)

S6 Dataset Differential Protein Expression. A tab-delimited text format file tabulating differential expression of 175 antibodies that probe for 136 autosomal and X chromosome proteins in the 6p+12p, 7p, and Xq tensor GSVD classes. The protein expression profiles of 282 patients are available in tab-delimited text format files at http://www.alterlab.org/OV_prognosis/.
(TXT)
