# Supplementary material for: Tensor GSVD of Patient- and Platform-Matched Tumor and Normal DNA Copy-Number Profiles Uncovers Chromosome Arm-Wide Patterns of Tumor-Exclusive Platform-Consistent Alterations Encoding for Cell Transformation and Predicting Ovarian Cancer Survival
Source: PLoS One. 2015 Apr 15;10(4):e0121396. doi: 10.1371/journal.pone.0121396 (PMC4398562; doi:10.1371/journal.pone.0121396)
Supplement: S1 Appendix — (PDF) [file pone.0121396.s001.pdf]

1. Mathematical Method: Tensor GSVD

1.1. Discovery Datasets are Pairs of Column-Matched but Row-Independent Tensors. The discovery set of patients reflects the general primary, high-grade OV patient population, with approximately 5%, 7%, 76%, and 12% of the patients diagnosed at stages I, II, III, and IV, and 218, i.e., ∼88%, treated with platinum-based chemotherapy, i.e., cisplatin, carboplatin, or oxaliplatin, and 240 of the 249, i.e., >95% of the tumors at grades 2 and higher.

Each profile in the discovery datasets lists $\log_2$ of TCGA level 1 background-subtracted intensity in the sample relative to the male Promega DNA reference, with signal to background ≥2.5 for both the sample and reference in ≥90% of the 391,190 autosomal probes and ≥65% of the 10,911 X chromosome probes that match between the two Agilent Human array CGH (aCGH) DNA microarray platforms, G4447A and G4124A. Tumor and normal probes were selected with valid data in ≥99% of the tumor or normal arrays of each platform, respectively. For each chromosome arm or combination of two chromosome arms, and for each platform, the <0.5% missing data entries in the tumor and normal profiles were estimated by using the SVD, as previously described [12]. Each profile was then centered at its copy-number median, and normalized by its copy-number sMAD.

1.2. The Tensor GSVD.

Existence, uniqueness and special cases.

Lemma A. The tensor GSVD exists for any two, e.g., third-order tensors $\mathcal{D}_i \in \mathbb{R}^{K_i \times L \times M}$ of the same column dimensions L and M but different row dimensions K_i, where $K_i \geq LM$ for $i = 1, 2$, if the tensors unfold into full column-rank matrices, $D_i \in \mathbb{R}^{K_i \times LM}$, $D_{ix} \in \mathbb{R}^{K_i M \times L}$, and $D_{iy} \in \mathbb{R}^{K_i L \times M}$, each preserving the K_i-row dimension, L-x-, or M-y- column dimension, respectively.

Proof. The tensor GSVD of Eq. (1), of the pair of third-order tensors $\mathcal{D}_i$, is constructed from the GSVDs of Eqs. (2) and (3), of the pairs of full column-rank matrices D_i, D_{ix}, and D_{iy}, where $i = 1, 2$. From the existence of the GSVDs of Eqs. (2) and (3) [5, 6], the orthonormal column bases vectors of U_i, as well as the normalized x- and y-row bases vectors of the invertible V_x^T or V_y^T, exist, and, therefore, the tensor GSVD of Eq. (1) also exists. Note that the proof holds for tensors of higher-than-third order. □

Lemma B. The tensor GSVD has the same uniqueness properties as the GSVD.

Proof. From the uniqueness properties of the GSVDs of Eqs. (2) and (3), the orthonormal column bases vectors $u_{i,a}$, and the normalized row bases vectors $v_{x,b}^T$, and $v_{y,c}^T$ of the tensor GSVD of Eq. (1) are unique, except in degenerate subspaces, defined by subsets of equal generalized singular values σ_i, σ_{ix}, and σ_{iy}, respectively, and up to phase factors of ±1. The tensor GSVD, therefore, has the same uniqueness properties as the GSVD. Note that the proof holds for tensors of higher-than-third order. □

Corollary A. For two second-order tensors, the tensor GSVD reduces to the GSVD of the corresponding matrices.

Proof. For two second-order tensors, e.g., the matrices $D_i \in \mathbb{R}^{K_i \times L}$, the tensor GSVD of Eq. (1) is

$$\begin{aligned} D_i &= R_i \times_a U_i \times_b V_x \\ &= U_i R_i V_x^T, \qquad i = 1, 2. \end{aligned} \tag{A1}$$

The row- and x-column mode GSVDs of Eqs. (2) and (3) are identical, because unfolding each matrix D_i while preserving either its K_i-row dimension, or L-x-column dimension results in D_i, up to permutations of either its columns or rows, respectively,

$$D_i = U_i \Sigma_i V_x^T = D_{ix}, \quad i = 1, 2. \tag{A2}$$

From the uniqueness properties of the tensor GSVD of Eq. (A1), and the GSVDs of Eq. (A2) it follows that $R_i = \Sigma_i$, and that for two second-order tensors, i.e., matrices, the tensor GSVD is equivalent to the GSVD. □

Theorem A. The tensor GSVD of the tensor $\mathcal{D}_1 \in \mathbb{R}^{LM \times L \times M}$, which row mode unfolding gives the identity matrix $D_1 = I \in \mathbb{R}^{LM \times LM}$, and a tensor $\mathcal{D}_2$ of the same column dimensions reduces to the HOSVD of $\mathcal{D}_2$.

Proof. Consider the GSVD of Eq. (2), of the matrices $D_1 = I$ and D_2, as computed by using the QR decomposition of the appended D_1 and D_2, and the SVD of the block of the resulting column-wise orthonormal Q that corresponds to D_2, i.e., $Q_2 = U_{Q_2} \Sigma_{Q_2} V_{Q_2}^T$ [5],

$$\begin{bmatrix} D_1 \\ D_2 \end{bmatrix} = \begin{bmatrix} I \\ D_2 \end{bmatrix} = QR = \begin{bmatrix} Q_1 \\ Q_2 \end{bmatrix} R = \begin{bmatrix} R^{-1} \\ U_{Q_2} \Sigma_{Q_2} V_{Q_2}^T \end{bmatrix} R, \tag{A3}$$

where R is upper triangular and, therefore, invertible. Since Q is column-wise orthonormal, $V_{Q_2}^T$ is orthonormal, and Σ_{Q_2} is positive diagonal, it follows that

$$\begin{aligned} I &= Q_1^T Q_1 + Q_2^T Q_2 \\ &= R^{-T} R^{-1} + V_{Q_2} \Sigma_{Q_2}^2 V_{Q_2}^T \\ &= (V_{Q_2}^T R)^{-T} (V_{Q_2}^T R)^{-1} + \Sigma_{Q_2}^2, \\ (I - \Sigma_{Q_2}^2)^{-1} &= (V_{Q_2}^T R)(V_{Q_2}^T R)^T, \end{aligned} \tag{A4}$$

and that $(I - \Sigma_{Q_2}^2)^{\frac{1}{2}} V_{Q_2}^T R$ is orthonormal. The GSVD of Eq. (2) factors the matrix D_2 into a column-wise orthonormal U_{Q_2}, a positive diagonal $\Sigma_{Q_2}(I - \Sigma_{Q_2}^2)^{-\frac{1}{2}}$ and an orthonormal $(I - \Sigma_{Q_2}^2)^{\frac{1}{2}} V_{Q_2}^T R$, and is, therefore, reduced to the SVD of D_2.

Note that this proof holds for the GSVDs of Eq. (3). This is because the x- and y-column unfoldings of the tensor $\mathcal{D}_1 \in \mathbb{R}^{LM \times L \times M}$, which row mode unfolding gives the identity matrix $D_1 = I \in \mathbb{R}^{LM \times LM}$, give

$$D_{1x} = \begin{bmatrix} I \\ \vdots \\ I \\ 0 \\ \vdots \\ 0 \end{bmatrix} \begin{matrix} \left.\vphantom{\begin{matrix} I \\ \vdots \\ I \end{matrix}}\right\} M \\ \left.\vphantom{\begin{matrix} 0 \\ \vdots \\ 0 \end{matrix}}\right\} M(M-1) \end{matrix},$$

$$D_{1y} = \begin{bmatrix} I \\ \vdots \\ I \\ 0 \\ \vdots \\ 0 \end{bmatrix} \begin{matrix} \left.\vphantom{\begin{matrix} I \\ \vdots \\ I \end{matrix}}\right\} L \\ \left.\vphantom{\begin{matrix} 0 \\ \vdots \\ 0 \end{matrix}}\right\} L(L-1) \end{matrix}. \quad \text{(A5)}$$

The GSVDs of Eqs. (2) and (3), of any one of the matrices D_1, D_{1x}, or D_{1y} with the corresponding full column-rank matrices D_2, D_{2x}, or D_{2y}, are, therefore, reduced to the SVDs of D_2, D_{2x}, or D_{2y}, respectively.

The tensor GSVD of Eq. (1), where the orthonormal column bases vectors $u_{2,a}$, and the normalized row bases vectors $v^T_{x,b}$, and $v^T_{y,c}$ in the factorization of the tensor $\mathcal{D}_2$ are computed via the SVDs of the unfolded tensor is, therefore, reduced to the HOSVD of $\mathcal{D}_2$ [25–27]. Note that the proof holds for tensors of higher-than-third order. □

Interpretation. The "tensor generalized Shannon entropy" of each dataset,

$$0 \le d_i = -(2\log LM)^{-1} \sum_{a=1}^{LM} \sum_{b=1}^{L} \sum_{c=1}^{M} \mathcal{P}_{i,abc} \log \mathcal{P}_{i,abc} \le 1, \quad i = 1, 2, \quad \text{(A6)}$$

measures the complexity of each dataset from the distribution of the overall information among the different subtensors. An entropy of zero corresponds to an ordered and redundant dataset in which all the information is captured by a single subtensor. An entropy of one corresponds to a disordered and random dataset in which all subtensors are of equal significance.

Fig. A (on p. A-3). The tensor GSVD of the patient- and platform-matched DNA copy-number profiles of the 7p chromosome arm. The tensor GSVD is depicted in a raster display, with relative copy-number gain (red), no change (black), and loss (green), explicitly showing the first through the 5th, and the 245th through the 249th 7p x-probelets, both 7p y-probelets, and the first through the 10th, and the 489th through the 498th 7p tumor and normal arraylets. We prove that the significance of a subtensor in the tumor dataset relative to that of the corresponding subtensor in the normal dataset, i.e., the tensor GSVD angular distance, equals the row mode GSVD angular distance, i.e., the significance of the corresponding tumor arraylet in the tumor dataset relative to that of the normal arraylet in the normal dataset. The tensor GSVD angular distances for the 498 pairs of 7p arraylets are depicted in a bar chart display, where the angular distance corresponding to the first pair of arraylets is $\sim\pi/4$. For the 7p chromosome arm, we find that the most significant subtensor in the tumor dataset is a combination of (i) the first y-probelet, which is approximately invariant across the platforms, (ii) the first x-probelet, which classifies the discovery set of patients into two groups of high and low coefficients, of significantly and robustly different prognoses, and (iii) the first, most tumor-exclusive tumor arraylet, which classifies the validation set of patients into two groups of high and low correlations of significantly different prognoses consistent with the x-probelet's classification of the discovery set.

Fig. B (on p. A-4). The tensor GSVD of the patient- and platform-matched DNA copy-number profiles of the Xq chromosome arm. The tensor GSVD is depicted in a raster display, with relative copy-number gain (red), no change (black), and loss (green), explicitly showing the first through the 5th, and the 245th through the 249th Xq x-probelets, both Xq y-probelets, and the first through the 10th, and the 489th through the 498th Xq tumor and normal arraylets. The tensor GSVD angular distances for the 498 pairs of Xq arraylets are depicted in a bar chart display, where the angular distance corresponding to the first pair of arraylets is $\sim\pi/4$.

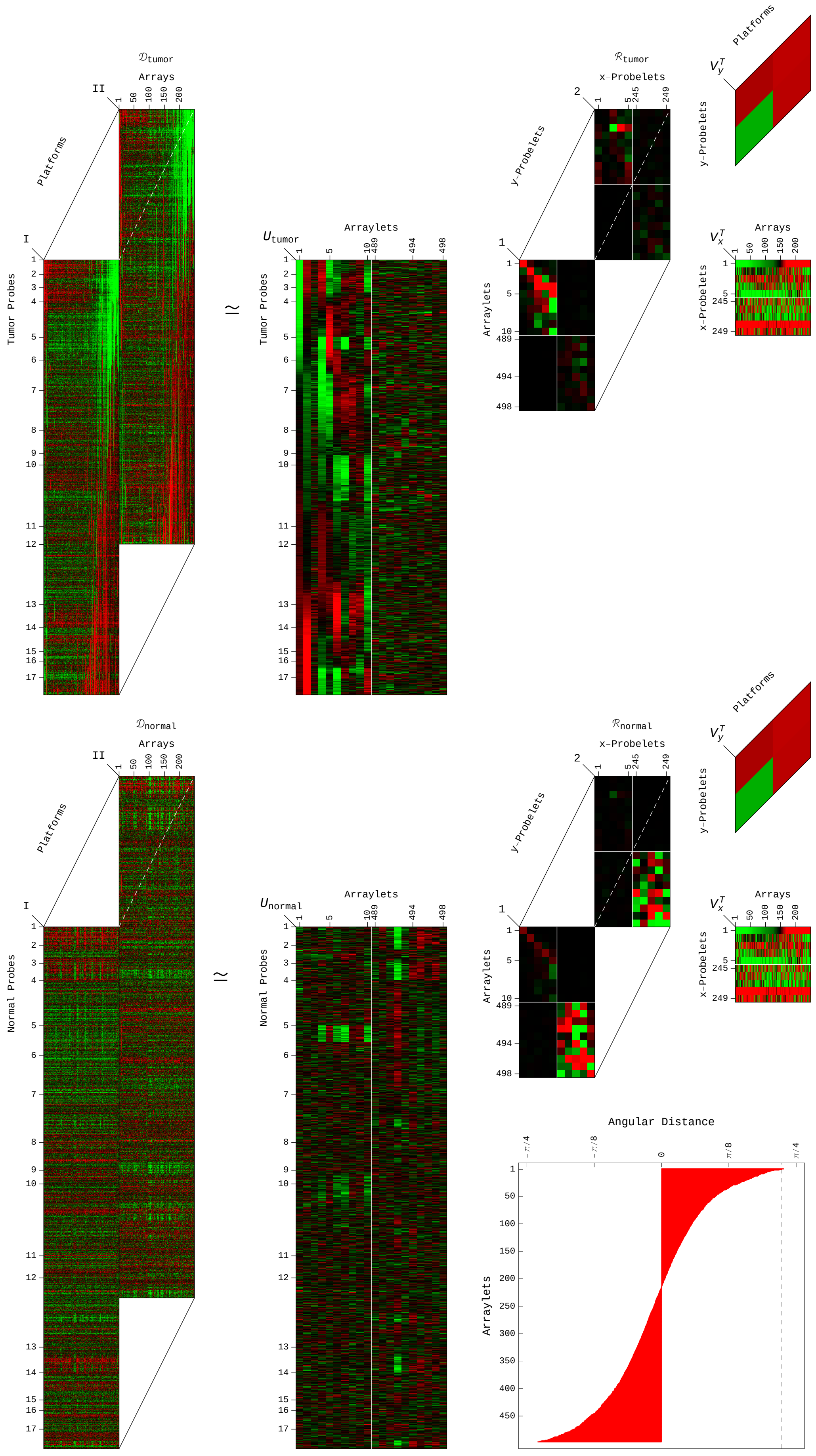

Fig. A (captions on p. A-2).

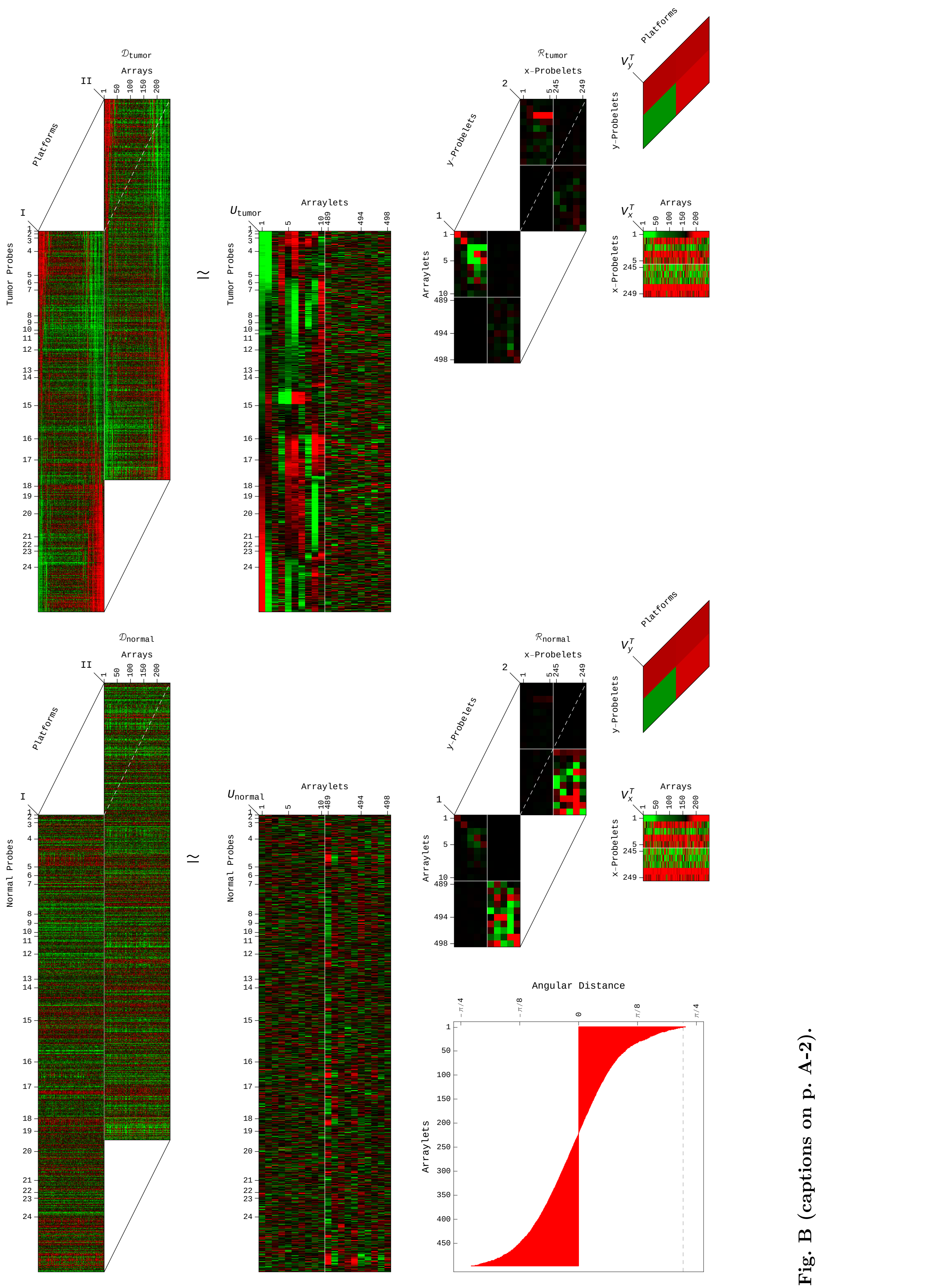

Fig. B (captions on p. A-2).

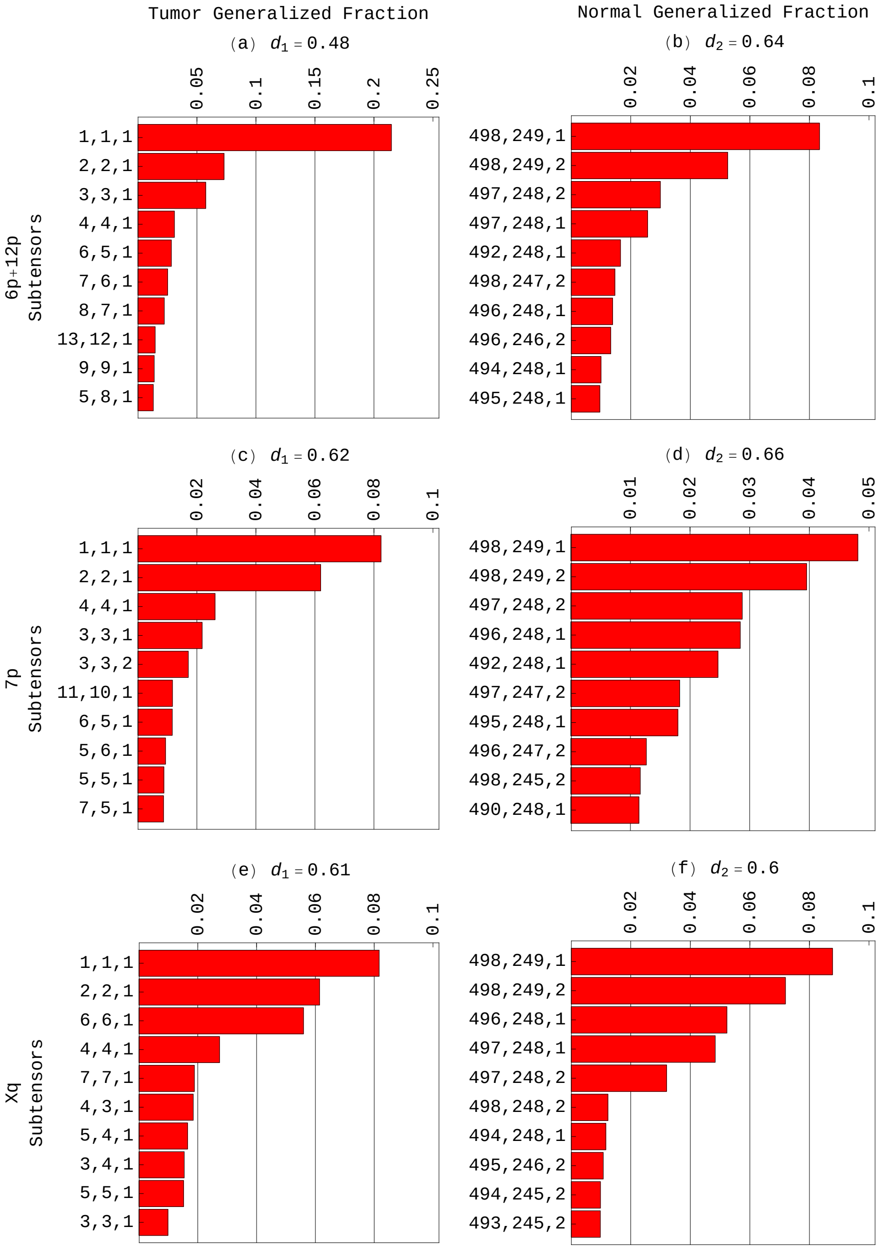

Fig. C. Most significant subtensors in the tumor and normal discovery datasets. Bar charts of the ten subtensors $\mathcal{S}_i(a,b,c)$ of Eq. (1) that are most significant in the 6p+12p (a) tumor, and (b) normal, 7p (c) tumor, and (d) normal, and Xq (e) tumor, and (f) normal datasets, in terms of the fractions $\mathcal{P}_{i,abc}$ of Eq. (4), i.e., the subtensors which correspond to the coefficients $\mathcal{R}_{i,abc}$ of largest magnitudes. The most significant subtensor in each of the tumor datasets, e.g., is $\mathcal{S}_1(1,1,1)$, which is a combination or an outer product of the first, most tumor-exclusive tumor arraylet, and the first x- and y-probelets. The most significant subtensor in each of the normal datasets is $\mathcal{S}_2(498,249,1)$, which is a combination or an outer product of the 498th, most normal-exclusive normal arraylet, the 249th x-probelet and the first y-probelet. The tensor generalized Shannon entropy d_i of Eq. (A6) of each dataset is also noted.

1.3. Discovery and Validation of CNAs Predicting OV Survival. For the validation dataset, we selected 131 and 41 stage III-IV OV aCGH profiles measured by the Agilent Human aCGH G4447A and G4124A microarray platforms, respectively, corresponding to 148 primary OV tumors. Of the 148 patients, 140, i.e., ∼95%, were treated with platinum-based chemotherapy, and 144, i.e., >95% of the tumors are high-grade, i.e., grades 2 and higher tumors. Each profile lists $\log_2$ of TCGA level 1 background-subtracted intensity in the sample relative to the male Promega DNA reference, with signal to background ≥2.5 for both the sample and reference in ≥99.5% of the 391,190 autosomal probes and ≥96.5% of the 10,911 X chromosome probes that match between the platforms. Medians of the profiles of samples from the same patient were then taken.

The arraylet correlation cutoff is the x-probelet coefficient cutoff scaled by the norm/$\sqrt{2}$ of the Spearman's rank correlation coefficients of the 498 tumor profiles of the discovery set of patients, as previously described [13].

2. Biological Results

2.1. Independent Chromosome Arm-Wide Predictors of OV Survival and Response to Platinum-Based Chemotherapy.

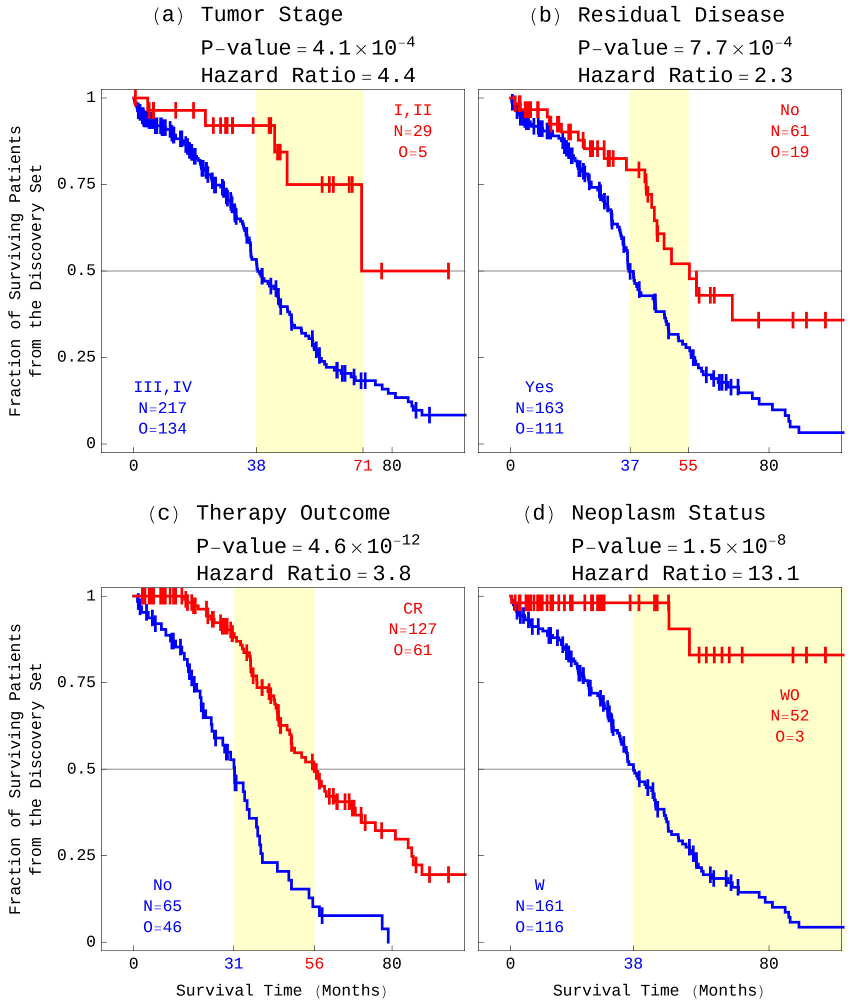

Fig. D. Survival analyses of the discovery set of patients classified by the standard OV indicators. KM curves of the discovery set of 249 patients classified by (a) tumor stage at diagnosis, the best predictor of OV survival to date, (b) residual disease after surgery, i.e., no (No) or some (Yes) macroscopic disease, (c) outcome of subsequent therapy, i.e., complete remission (CR) or not (No). (d) neoplasm status, i.e., with (W) tumor or without (WO).

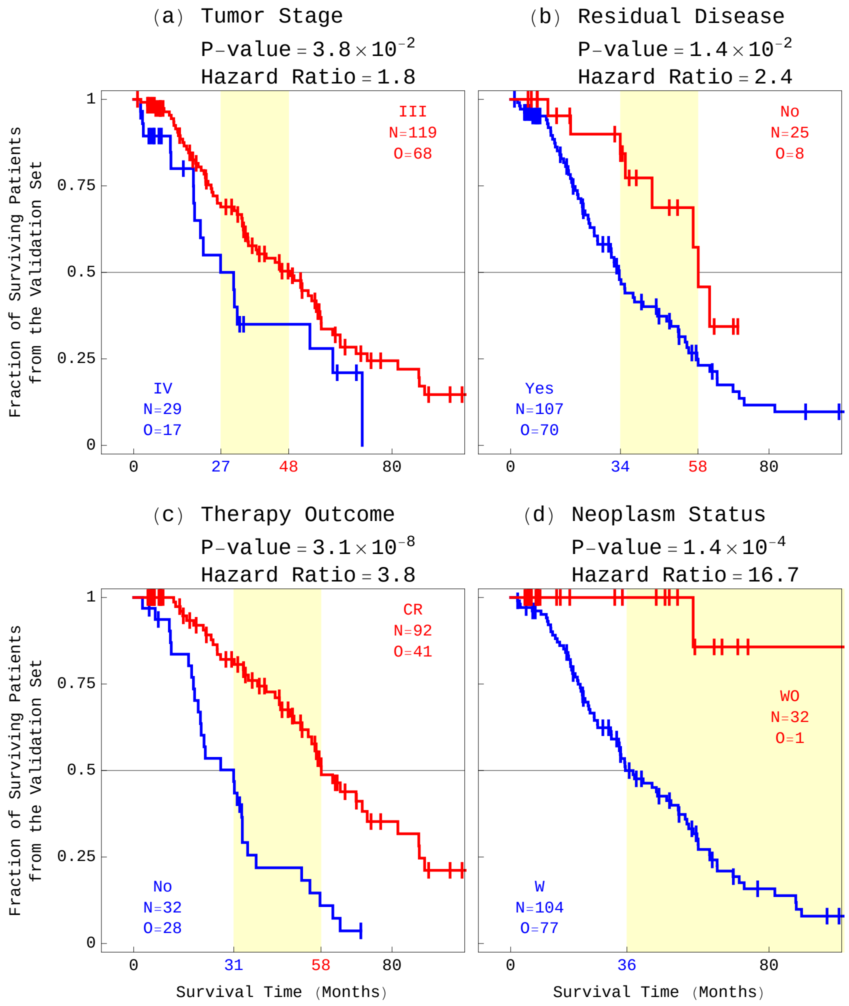

Fig. E. Survival analyses of the validation set of patients classified by the standard OV indicators. KM curves of the validation set of 148 stage III-IV patients classified by (*a*) tumor stage at diagnosis, (*b*) residual disease after surgery, i.e., no (No) or some (Yes) macroscopic disease, (*c*) outcome of subsequent therapy, i.e., complete remission (CR) or not (No). (*d*) neoplasm status, i.e., with (W) tumor or without (WO).

Fig. F (on p. A-8). Survival analyses of the platinum-based chemotherapy patients in the discovery and validation sets classified by tensor GSVD, or tensor GSVD and tumor stage at diagnosis. (*a*) Kaplan-Meier (KM) curves of only the 218, i.e., ∼88% platinum-based chemotherapy patients in the discovery set, classified by the 6p+12p x-probelet coefficient, show a median survival time difference of 14 months, with the corresponding log-rank test P-value $< 10^{-3}$. The univariate Cox proportional hazard ratio is 2.0. (*b*) Survival analyses of the 218 patients classified by the 7p x-probelet coefficient. (*c*) The 218 patients classified by the Xq x-probelet coefficient. (*d*) The 218 patients classified by both the 6p+12p tensor GSVD and tumor stage at diagnosis, show the bivariate Cox hazard ratios of 1.8 and 4.1, which do not differ significantly from the corresponding univariate hazard ratios of 2.0 and 4.4, respectively. This means that the 6p+12p tensor GSVD is independent of stage, the best predictor of OV survival to date. (*e*) The 218 patients classified by both the 7p tensor GSVD and stage. (*f*) The 218 patients classified by both the Xq tensor GSVD and stage. (*g*) KM curves of only the 140, i.e., ∼95% platinum-based chemotherapy patients in the validation set, classified by the 6p+12p arraylet correlation, show a median survival time difference of 18 months, with the univariate Cox proportional hazard ratio 1.8. This validates the survival analyses of the 218 chemotherapy patients in the discovery set. (*h*) Survival analyses of the 148 patients classified by the 7p arraylet correlation. (*i*) The 148 patients classified by the Xq arraylet correlation.

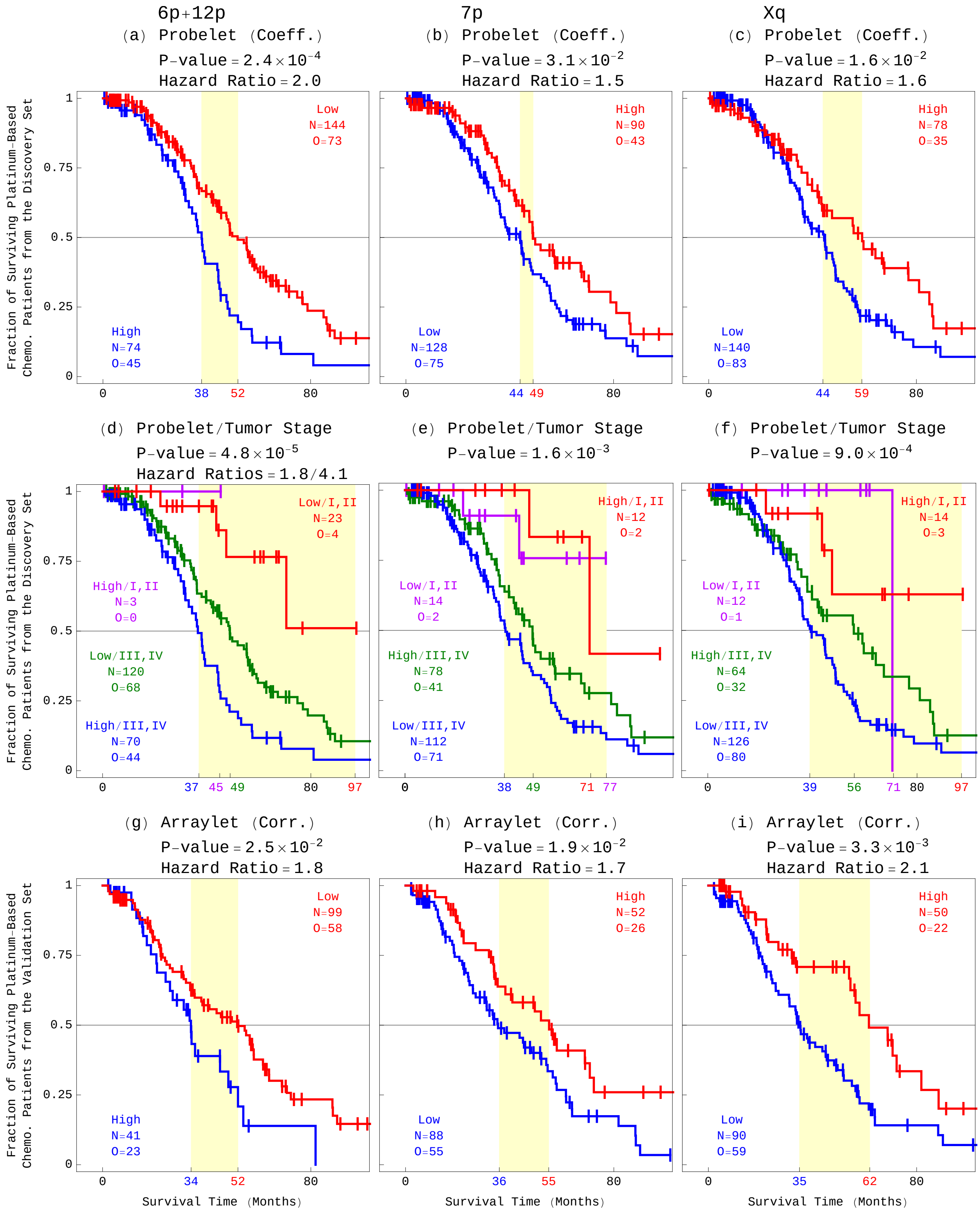

Fig. F (captions on p. A-7).

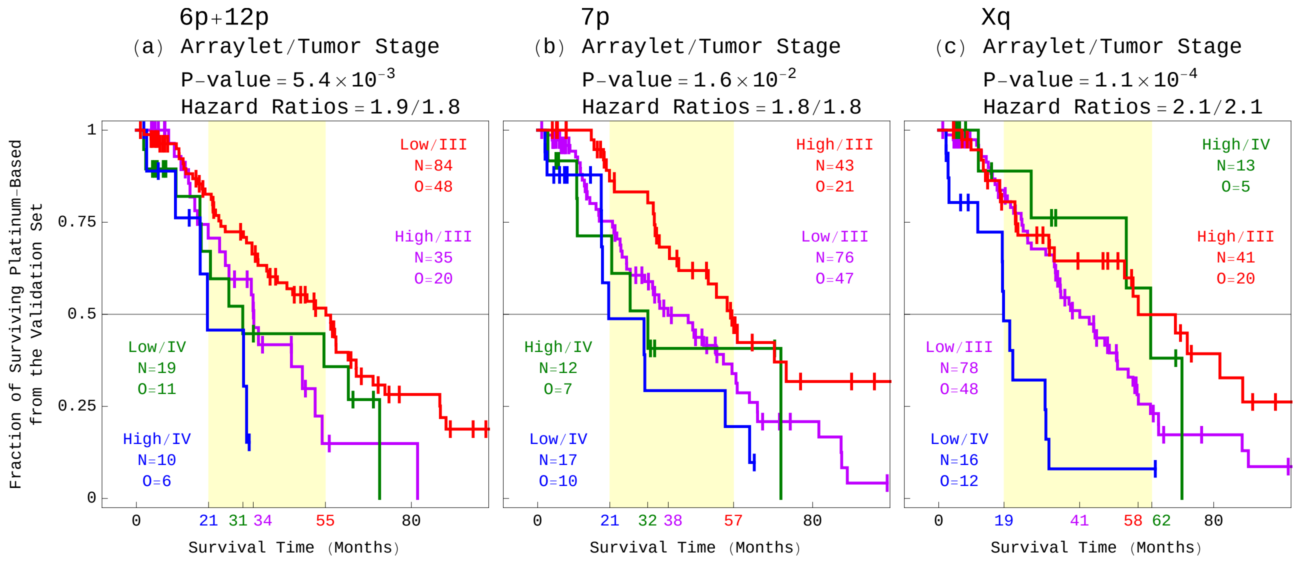

Fig. G. Survival analyses of the validation set of patients classified by tensor GSVD and tumor stage at diagnosis. (*a*) KM curves of the validation set of 148 stage III-IV patients classified by both the 6p+12p tensor GSVD and tumor stage at diagnosis, show the bivariate Cox hazard ratios of 1.9 and 1.8, which are the same as the corresponding univariate ratios. This means that the 6p+12p tensor GSVD is independent of stage, the best predictor of OV survival to date. The 34 months KM median survival time difference is about 62% and more than one year greater than the 21 month difference between the patients classified by stage alone. This means that the tensor GSVD and stage combined make a better predictor than stage alone. (*b*) The 148 patients classified by both the 7p tensor GSVD and stage. (*c*) The 148 patients classified by both the Xq tensor GSVD and stage.

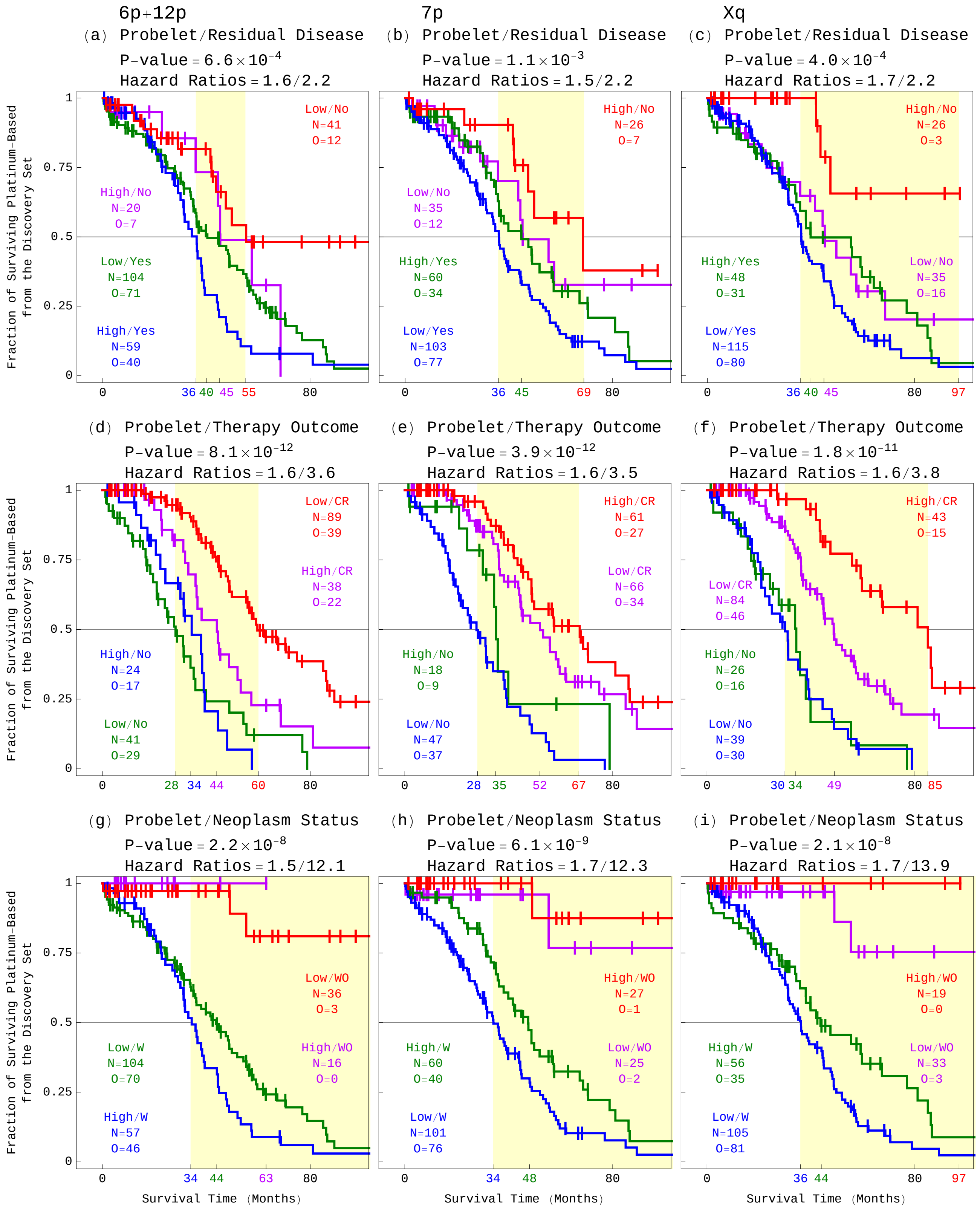

Fig. H. Survival analyses of the discovery set of patients classified by tensor GSVD and standard OV indicators other than stage. KM curves of the discovery set of 249 patients classified by both the (*a*) 6p+12p, (*b*) 7p, or (*c*) Xq tensor GSVD, and residual disease after surgery, the (*d*) 6p+12p, (*e*) 7p, or (*f*) Xq tensor GSVD, and outcome of subsequent therapy, and (*g*) 6p+12p, (*h*) 7p, or (*i*) Xq tensor GSVD, and neoplasm status.

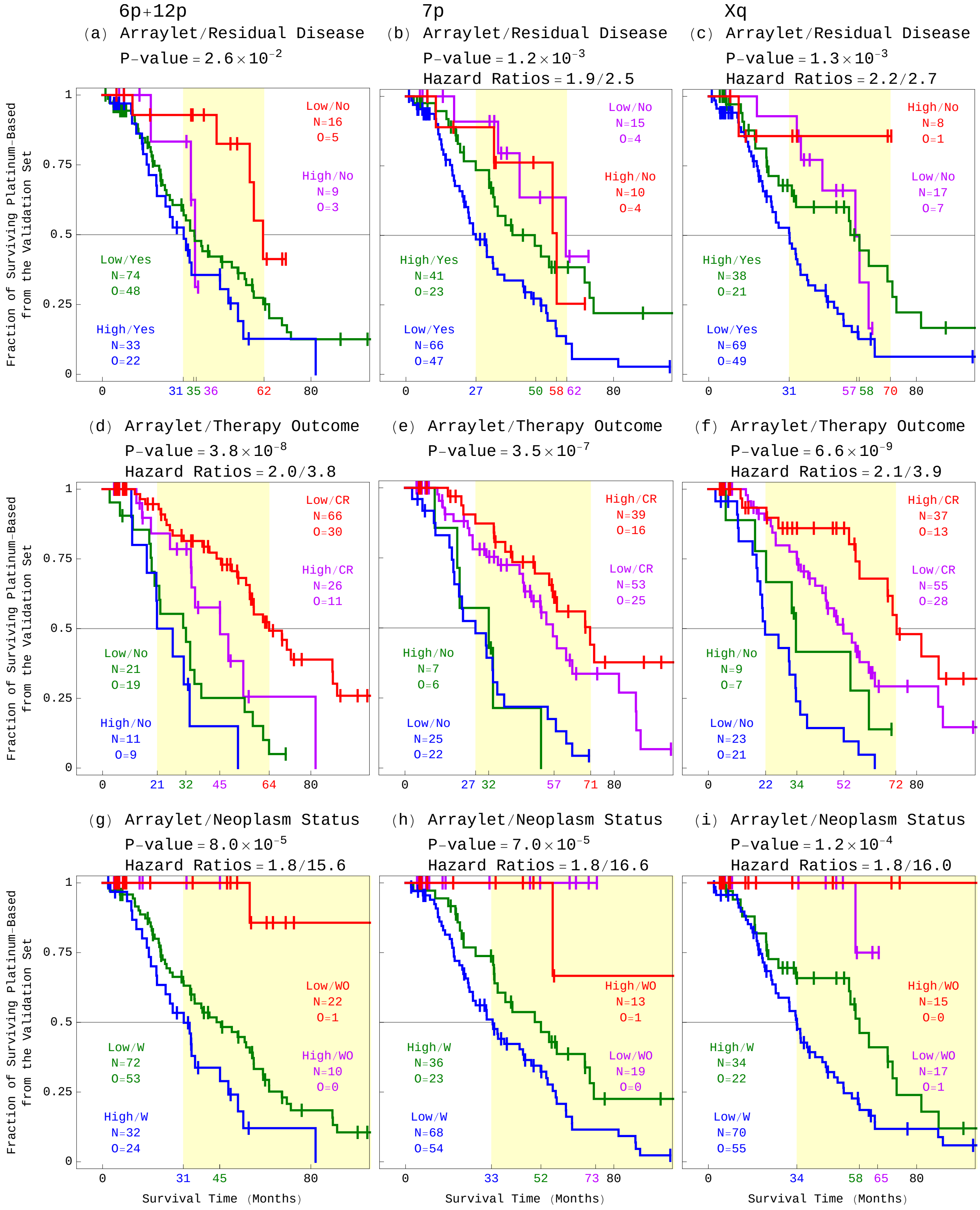

Fig. I. Survival analyses of the validation set of patients classified by tensor GSVD and standard OV indicators other than stage. KM curves of the validation set of 148 stage III-IV patients classified by both the (*a*) 6p+12p, (*b*) 7p, or (*c*) Xq tensor GSVD, and residual disease after surgery, the (*d*) 6p+12p, (*e*) 7p, or (*f*) Xq tensor GSVD, and outcome of subsequent therapy, and (*g*) 6p+12p, (*h*) 7p, or (*i*) Xq tensor GSVD, and neoplasm status.

Predictor		Discovery and Validation Sets Hazard Ratio	P-value
Tensor GSVD	6p+12p	1.8	1.0×10^{-4}
	7p	1.7	1.7×10^{-4}
	Xq	1.7	4.8×10^{-4}
Tumor Stage		4.1	1.8×10^{-3}
Residual Disease		2.3	8.4×10^{-5}
Therapy Outcome		3.8	8.3×10^{-17}
Neoplasm Status		14.0	1.8×10^{-7}

Table A. Cox univariate proportional hazard models of the discovery and validation sets of patients classified by any one of the tensor GSVDs or the standard OV indicators.

Chromosome Arm	Predictor	Discovery and Validation Sets Hazard Ratio	P-value
6p+12p	Tensor GSVD	1.7	4.4×10^{-4}
	Tumor Stage	3.7	3.9×10^{-3}
	Tensor GSVD	1.6	2.5×10^{-3}
	Residual Disease	2.2	1.2×10^{-4}
	Tensor GSVD	1.7	1.2×10^{-3}
	Therapy Outcome	3.7	1.9×10^{-15}
	Tensor GSVD	1.6	1.2×10^{-3}
	Neoplasm Status	13.0	3.9×10^{-7}
7p	Tensor GSVD	1.7	4.2×10^{-4}
	Tumor Stage	3.9	2.4×10^{-3}
	Tensor GSVD	1.6	1.3×10^{-3}
	Residual Disease	2.2	1.1×10^{-4}
	Tensor GSVD	1.5	1.6×10^{-2}
	Therapy Outcome	3.5	2.4×10^{-14}
	Tensor GSVD	1.7	6.0×10^{-4}
	Neoplasm Status	13.3	3.0×10^{-7}
Xq	Tensor GSVD	1.6	1.7×10^{-3}
	Tumor Stage	3.8	3.2×10^{-3}
	Tensor GSVD	1.9	1.1×10^{-4}
	Residual Disease	2.2	9.3×10^{-5}
	Tensor GSVD	1.8	8.5×10^{-4}
	Therapy Outcome	3.8	1.1×10^{-16}
	Tensor GSVD	1.7	6.7×10^{-4}
	Neoplasm Status	14.5	1.3×10^{-7}

Table B. Cox bivariate proportional hazard models of the patients in the discovery and validation sets classified by both tensor GSVD and the standard OV indicators.

2.2. Novel Frequent Focal CNAs Indicating Survival. To interpret the 6p+12p, 7p, and Xq tumor arraylets, we mapped the tumor probes onto the National Center for Biotechnology Information (NCBI) human genome sequence build 37, by using the Agilent Technologies probe annotations posted at the University of California at Santa Cruz (UCSC) human genome browser [20]. We segmented each of the arraylets and assigned each segment a P-value by using the circular binary segmentation (CBS) algorithm, as previously described [21]. To assign a CNA in a segment, we calculated the segment's median copy number, and sMAD from the median in the corresponding arraylet. If the segment's median is at least one sMAD greater (or lesser) than the arraylet's median, then the arraylet is assigned a gain (or a loss) in the segment. Similarly, we calculated the segment's median copy number, and sMAD from the median in each tumor profile. If the segment's median is at least one sMAD greater (or lesser) than the profile's median, then the patient is assigned a gain (or a loss) in the segment.

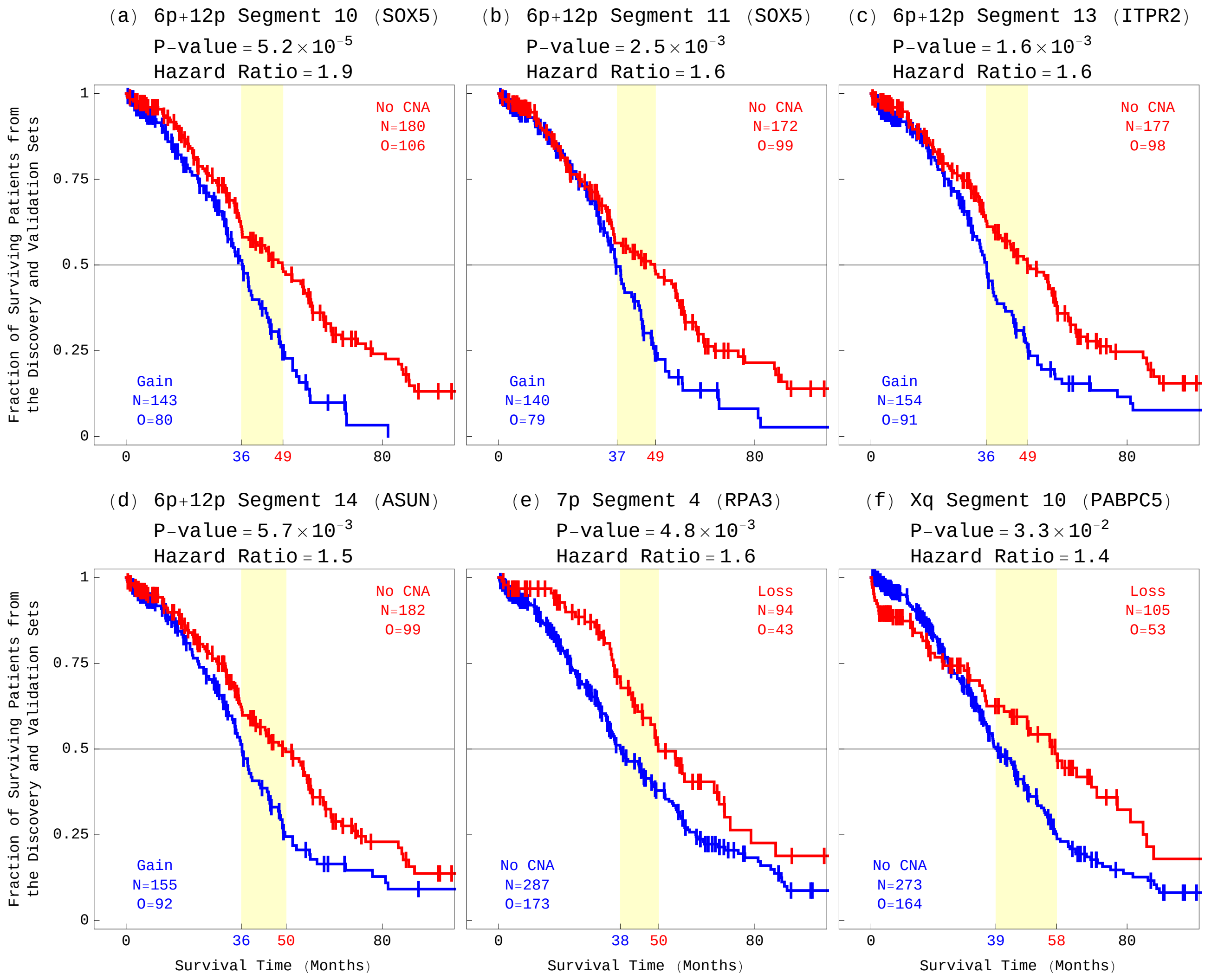

Fig. J. Survival analyses of the discovery and validation sets of patients classified by the novel frequent focal CNAs included in the tensor GSVD arraylets. Six novel frequent focal CNAs that are included in the tensor GSVD arraylets are significantly correlated with OV survival. Two amplified consecutive segments (12p12.1) contain (*a*) the 5' ends of isoforms a and e of *SOX5*, and (*b*) exons 5 and 6, the first exons that are common to isoforms a, b, d, and e of *SOX5*. Two other amplified consecutive segments (12p11.23) contain (*c*) *ITPR2* and (*d*) *ASUN*. One deletion (7p22.1-p21.3) contains (*e*) *RPA3*. Another deletion (Xq21.31) contains (*f*) *PABPC5*, and the sequence tag site DXS241 adjacent to translocation breakpoints observed in premature ovarian failure.

2.3. Possible Roles in OV Pathogenesis. To compare the variation in DNA copy numbers with that in gene expression, we used mRNA expression profiles that were available for 394 of the 397 TCGA patients in the discovery and validation sets. Each profile lists TCGA level 3 mRNA expression for 11,457 autosomal and X chromosome genes on the Affymetrix Human Genome U133A Array platform with UCSC coordinates [20] and GO annotations [39]. Medians of the profiles of samples from the same patient were taken. To examine the possible relations between a tensor GSVD class and the OV pathogenesis, we assessed the enrichment of the subsets of genes that are differentially expressed between the tensor GSVD classes in any one of the multiple GO annotations [40]. The P-value of a given enrichment was calculated assuming hypergeometric probability distribution of the annotations among the genes in the global set, and of the subset of annotations among the subset of genes, as previously described [12].

To compare with the variation in microRNA expression, we used microRNA expression profiles that were available for 395 of the 397 patients. Each profile lists TCGA level 3 microRNA expression for 639 autosomal and X chromosome microRNAs on the Agilent Human microRNA Array 8x15K platform with UCSC coordinates. Medians of the profiles of samples from the same patient were taken.

To compare with the variation in protein expression, we used protein expression profiles that were available for 282 of the 397 patients. Each profile lists TCGA level 3 protein expression for the 175 antibodies on the MD Anderson Reverse Phase Protein Array (RPPA), which probe for the abundance levels of 136 proteins encoded by autosomal and X chromosome genes.

We find that the CNAs are consistent with differential mRNA, microRNA, and protein expression between the tensor GSVD classes (Figs. K–M). The mRNA and protein encoded by, e.g., *MAPK14*, which is deleted in the 6p+12p arraylet, are both significantly (Mann-Whitney-Wilcoxon P-values $<10^{-5}$) underexpressed in the tensor GSVD class of a high 6p+12p x-probelet coefficient, or arraylet correlation relative to the tensor GSVD class of a low 6p+12p x-probelet coefficient, or arraylet correlation. The microRNA mir-877* that maps to the same deletion as *MAPK14* is also significantly (Mann-Whitney-Wilcoxon P-value <0.05) underexpressed.

Fig. K (on p. A-15). Differential mRNA expression between the tensor GSVD classes is consistent with the CNAs. (a) *TNF*, (b) *MAPK14*, and (c) *CDKN1A*, which are deleted in the 6p+12p arraylet, are significantly (Mann-Whitney-Wilcoxon P-value <0.05) underexpressed in the tensor GSVD class of a high 6p+12p x-probelet coefficient, or arraylet correlation relative to the tensor GSVD class of a low 6p+12p x-probelet coefficient, or arraylet correlation. (d) *RAD51AP1*, (e) *ITPR2*, and (f) *ASUN*, which are amplified in the 6p+12p arraylet, are significantly overexpressed in the tensor GSVD class of a high 6p+12p x-probelet coefficient, or arraylet correlation. (g) *RPA3*, which is deleted, and (h) *POLD2*, which is amplified, in the 7p arraylet, are significantly underexpressed and overexpressed, respectively, in the tensor GSVD class of a high 7p x-probelet coefficient, or arraylet correlation. (i) *BCAP31*, which is amplified in the Xq arraylet, is significantly overexpressed in the tensor GSVD class of a high Xq x-probelet coefficient, or arraylet correlation.

Fig. L (on p. A-16). Differential microRNA expression between the tensor GSVD classes is consistent with the CNAs. (a) mir-877*, which is deleted, and (b) mir-200c, (c) mir-200c*, (d) mir-141, and (e) mir-141*, which are amplified in the 6p+12p arraylet, are significantly (Mann-Whitney-Wilcoxon P-value <0.05) underexpressed and overexpressed, respectively, in the tensor GSVD class of a high 6p+12p x-probelet coefficient, or arraylet correlation relative to the tensor GSVD class of a low 6p+12p x-probelet coefficient, or arraylet correlation. (f) mir-888, (g) mir-224, and (h) mir-452, which are amplified in the Xq arraylet, are significantly overexpressed in the tensor GSVD class of a high Xq x-probelet coefficient, or arraylet correlation.

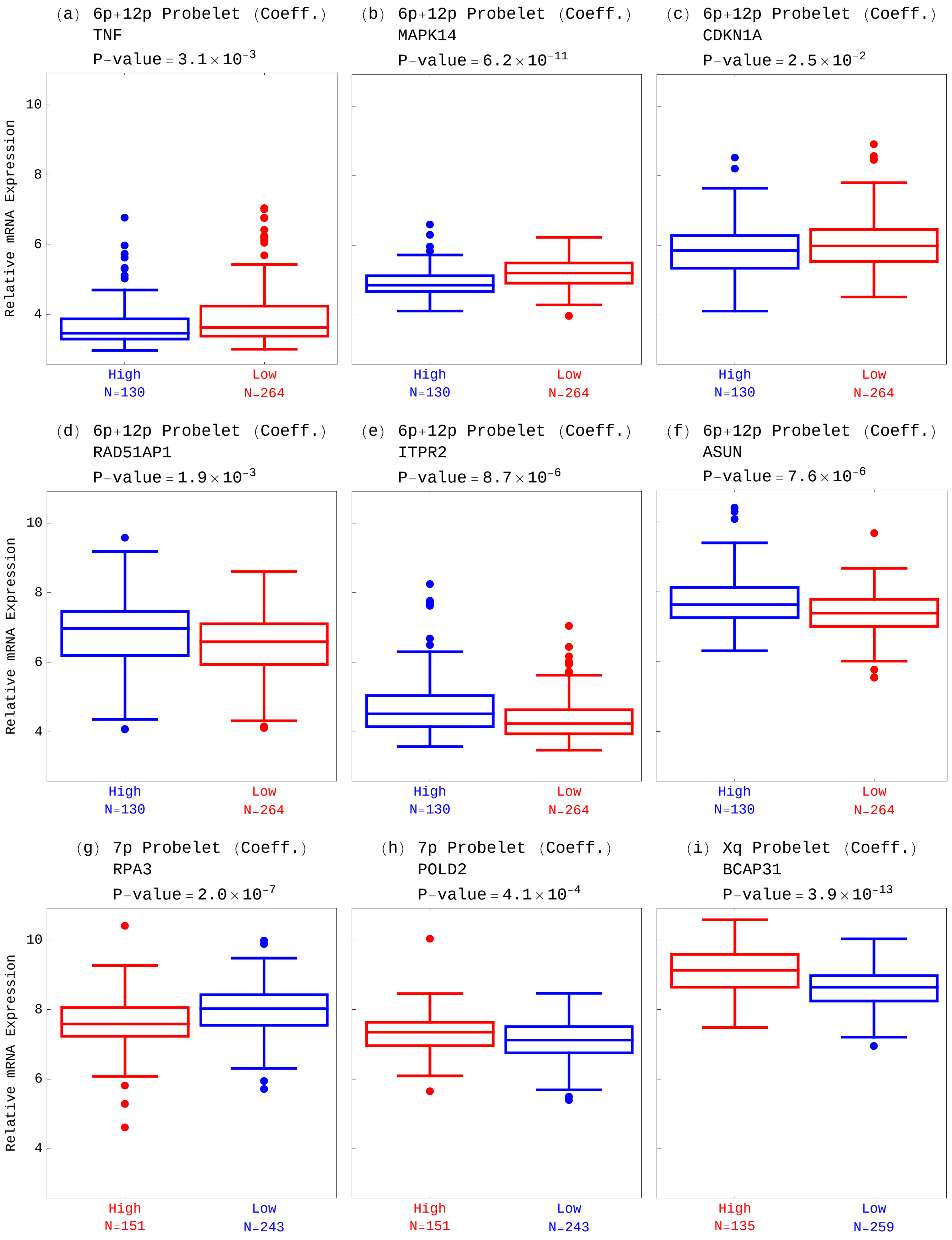

Fig. K (captions on p. A-14).

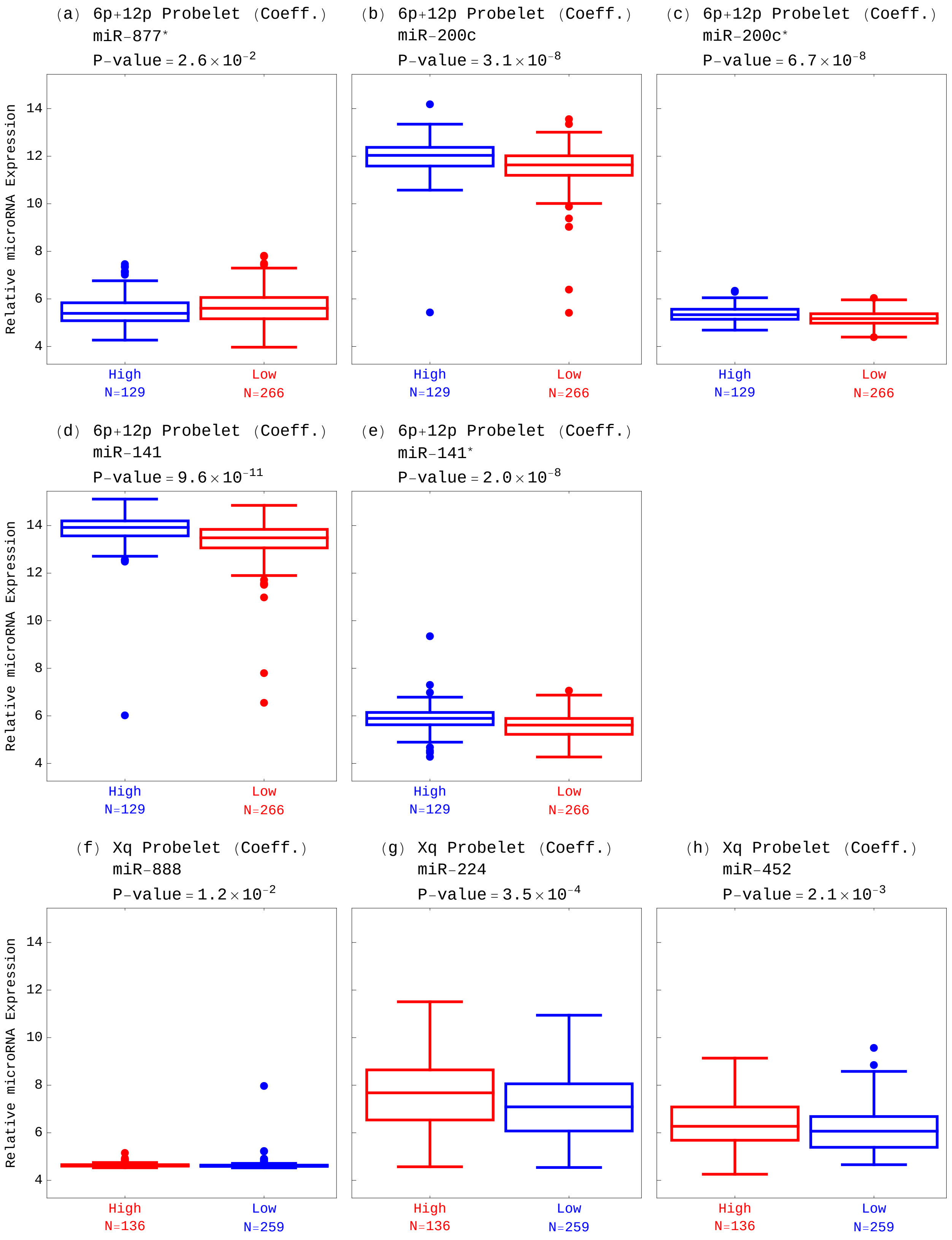

Fig. L (captions on p. A-14).

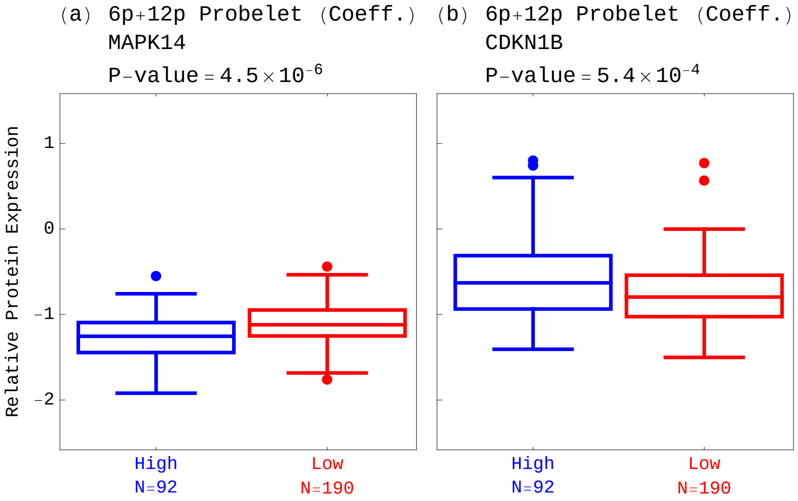

Fig. M. Differential protein expression between the tensor GSVD classes is consistent with the CNAs. (*a*) *MAPK14*, which is deleted, and (*b*) *CDKN1B*, which is amplified in the 6p+12p arraylet, are significantly (Mann-Whitney-Wilcoxon P-value <0.05) underexpressed and overexpressed, respectively, in the tensor GSVD class of a high 6p+12p x-probelet coefficient, or arraylet correlation relative to the tensor GSVD class of a low 6p+12p x-probelet coefficient, or arraylet correlation.
