# Supplementary material for: Tensor GSVD of Patient- and Platform-Matched Tumor and Normal DNA Copy-Number Profiles Uncovers Chromosome Arm-Wide Patterns of Tumor-Exclusive Platform-Consistent Alterations Encoding for Cell Transformation and Predicting Ovarian Cancer Survival
Source: PLoS One. 2015 Apr 15;10(4):e0121396. doi: 10.1371/journal.pone.0121396 (PMC4398562; doi:10.1371/journal.pone.0121396)
Supplement: S1 Mathematica Notebook — A PDF format file, readable by Adobe Acrobat Reader. The corresponding Mathematica 9.0.1 code file, executable by Mathematica and readable by Mathematica Player, is available at http://www.alterlab.org/OV_prognosis/. (PDF) [file pone.0121396.s002.pdf]

(* © Preethi Sankaranarayanan, Theodore E. Schomay, Katherine A. Aiello, and Orly Alter 2015, All Rights Reserved *)

(* Tensor GSVD of Patient- and Platform-Matched Tumor and Normal aCGH Datasets *)

```
(* Initialize *)

Clear["Global`*"]
SetOptions[Graphics, BaseStyle → {FontFamily → "Courier"}];
resolution = 3600;

(* Define Path to Datasets *)

path = "Desktop/OV/Data/";

(* Define the Tensor GSVD Function *)

tGSVD[tumor1_, tumor2_, normal1_, normal2_] :=
 Module[{zTumorArraylets, zNormalArraylets, tumorCore, normalCore,
   xProbelets, yProbelets, tumorArraylets, normalArraylets, d1, d2,
   probelets, tumorData1, tumorData2, normalData1, normalData2}, (
   (* Compute the Mode-x Tensor GSVD *)
   {xProbelets, tumorArraylets, normalArraylets, d1, d2} =
    gsvd[Join[tumor1, tumor2], Join[normal1, normal2]];
   (* Compute the Mode-z Tensor GSVD *)
   {probelets, zTumorArraylets, zNormalArraylets, d1, d2} =
    gsvd[Join[tumor1, tumor2, 2], Join[normal1, normal2, 2]];
   (* Compute the Mode-y Tensor GSVD *)
   {yProbelets, tumorArraylets, normalArraylets, d1, d2} =
    gsvd[Transpose[Join[{Flatten[tumor1]}, {Flatten[tumor2]}]],
     Transpose[Join[{Flatten[normal1]}, {Flatten[normal2]}]]];
   (* Order the First Probelet and the Corresponding Tumor and Normal Arraylets *)
   tumorData1 = tumor1[[All, Ordering[xProbelets[[1]]]]];
   tumorData2 = tumor2[[All, Ordering[xProbelets[[1]]]]];
   normalData1 = normal1[[All, Ordering[xProbelets[[1]]]]];
   normalData2 = normal2[[All, Ordering[xProbelets[[1]]]]];
   xProbelets = xProbelets[[All, Ordering[xProbelets[[1]]]]];
   yProbelets[[1]] = yProbelets[[1]] * (-1);
   (* Compute the Tensor GSVD Core *)
   {tumorCore, normalCore} = coreTensor[tumorData1, tumorData2, normalData1,
     normalData2, xProbelets, yProbelets, zTumorArraylets, zNormalArraylets];
   {tumorData1, tumorData2, normalData1, normalData2, zTumorArraylets,
    zNormalArraylets, tumorCore, normalCore, xProbelets, yProbelets}
  )]
```

```
(* Define the GSVD Function *)

gsvd[tumorData_, normalData_] :=
 Module[{q, q1, q2, r, u1, u2, w1, w2, vt1, vt2, d1, d2, signs, probelets,
   tumorArraylets, normalArraylets, tumorProbes, normalProbes, arrays}, (
   {tumorProbes, arrays} = Dimensions[tumorData];
   {normalProbes, arrays} = Dimensions[normalData];
   (* Compute the QR Decomposition of the Appended Tumor and Normal Datasets *)
   {q, r} = QRDecomposition[Join[tumorData, normalData]];
   q = Transpose[q];
   (* Compute the SVD of the Tumor Block of Q *)
   q1 = q[[1 ;; tumorProbes]];
   {u1, w1, vt1} = SingularValueDecomposition[q1, arrays];
   vt1 = Transpose[vt1].r;
   d1 = DiagonalMatrix[Diagonal[Sqrt[vt1.Transpose[vt1]]]];
   Do[vt1[[a]] = vt1[[a]] / d1[[a, a]], {a, 1, arrays}];
   d1 = w1.d1;
   probelets = vt1;
   tumorArraylets = u1;
   (* Compute the SVD of the Normal Block of Q *)
   q2 = q[[tumorProbes + 1 ;; normalProbes + tumorProbes]];
   {u2, w2, vt2} = SingularValueDecomposition[q2, arrays];
   vt2 = Transpose[vt2].r;
   d2 = DiagonalMatrix[Diagonal[Sqrt[vt2.Transpose[vt2]]]];
   Do[vt2[[a]] = vt2[[a]] / d2[[a, a]], {a, 1, arrays}];
   (* Order the SVD of the Normal Block of Q by Ascending Singular Values *)
   vt2 = Transpose[Take[
          Transpose[Sort[
            Transpose[
             Join[{Diagonal[w2]}, Transpose[vt2]]
             ]]][[2 ;; arrays + 1]]]];
   d2 = DiagonalMatrix[
         Sort[Diagonal[w2]] *
          Transpose[Sort[
            Transpose[
             Join[{Diagonal[w2]}, {Diagonal[d2]}]
             ]]][[2]]];
   normalArraylets =
        Take[Transpose[
          Sort[Transpose[
            Join[{Diagonal[w2]}, u2]
            ]]][[2 ;; normalProbes + 1]]];
   signs = DiagonalMatrix[Diagonal[vt2.Transpose[vt1]]];
   vt2 = signs.vt2;
   normalArraylets = normalArraylets.signs;
   {probelets, tumorArraylets, normalArraylets, d1, d2}
  )]
```

```
(* Define the Core Tensor Function *)

coreTensor[tumor1_, tumor2_, normal1_, normal2_,
  xProbelets_, yProbelets_, zTumorArraylets_, zNormalArraylets_] :=
 Module[{tumorCore, normalCore}, (
   tumorCore = Transpose[
     {Dot[tumor1, Inverse[xProbelets]], Dot[tumor2, Inverse[xProbelets]]}, {3, 1, 2}];
   normalCore = Transpose[{Dot[normal1, Inverse[xProbelets]],
      Dot[normal2, Inverse[xProbelets]]}, {3, 1, 2}];
   tumorCore = Dot[Transpose[zTumorArraylets], tumorCore];
   normalCore = Dot[Transpose[zNormalArraylets], normalCore];
   tumorCore = Dot[tumorCore, Inverse[yProbelets]];
   normalCore = Dot[normalCore, Inverse[yProbelets]];
   {tumorCore, normalCore}
  )]

(* Define the Arraylet Standardization *)

centeredArraylet[arraylet_] :=
 Module[{arrayletMedian, medianAbsoluteDeviations, arrayletCentered},
  (arrayletMedian = Median[arraylet];
   medianAbsoluteDeviations = 1.4826 * MedianDeviation[arraylet];
   arrayletCentered = (arraylet - arrayletMedian) / medianAbsoluteDeviations;
   {arrayletCentered, arrayletMedian, medianAbsoluteDeviations}
  )]

(* Define the Chromosome Band Coordinates *)

arrayletVariables[armBandCoordinates_, arm_] :=
 Module[{armBands, xcoordinates, list, bands, indices, num, subLists, minVal, maxVal}, (
   armBands = armBandCoordinates;
   armBands[[2 ;;, 2]] = armBands[[2 ;;, 2]] + 1;
   armBands[[2 ;;, 3]] = armBands[[2 ;;, 3]] + 1;
   xcoordinates = Range[Length[arm]];
   list = Transpose[Join[{xcoordinates}, {arm[[All, -1]]}]];
   bands = Range[Length[armBands]];
   indices = Flatten[Map[(num = #;
        Position[arm[[All, 2]], _?(# ≤ armBands[[num, 3]] &)][[-1]]) &,
      Range[2, Length[armBands]]]];
   indices = DeleteDuplicates[indices];
   subLists =
    Map[If[# ≠ 1, list[[indices[[# - 1]] + 1 ;; indices[[#]]]], list[[1 ;; indices[[#]]]]] &,
     Range[Length[indices]]];
   minVal = Round[Min[arm[[All, -1]]]];
   maxVal = Ceiling[Max[arm[[All, -1]]]];
   {subLists, minVal, maxVal, indices}
  )]
```

```
(* Define the Segments *)

armSegments[arm_, arrayletMedian_, medianAbsoluteDeviations_] :=
 Module[{armsegments, segmentMean, scaledSegmentMean},
  (armsegments = Import[path <> "Tumor_Arraylets_1.txt", "Table"];
   If[arm ≠ "6p+12p", armsegments = armsegments[[Position[armsegments[[All, 2]], arm][[1]][[
          1]] ;; Position[armsegments[[All, 2]], arm][[-1]][[1]]]];,
    armsegments = Join[armsegments[[Position[armsegments[[All, 2]], 6][[1]][[1]]
          ;; Position[armsegments[[All, 2]], 6][[-1]][[1]]]],
       armsegments[[Position[armsegments[[All, 2]], 12][[1]][[1]] ;;
          Position[armsegments[[All, 2]], 12][[-1]][[1]]]]];];
   segmentMean = armsegments[[All, 10]];
   scaledSegmentMean = (segmentMean - arrayletMedian) / medianAbsoluteDeviations;
   armsegments[[All, 10]] = scaledSegmentMean;
   armsegments[[All, 8]] = armsegments[[All, 10]];
   armsegments
  )]

(* Define the Arraylet Plot *)

arrayletPlotDisplay[arm_, subLists_, xticks_, minVal_, maxVal_, indices_, segments_,
  genes_, armName_, letter_, colorDefinitions_, probes_: 0] := Module[{bandarraylet}, (
   bandarraylet = Show[ListPlot[subLists, AspectRatio → 0.15, FrameLabel → {None,
         Framed[Column[{Style["Relative DNA", FontFamily → "Courier", FontSize → labelsize],
            Style["Copy Number", FontFamily → "Courier", FontSize → labelsize]}, Center],
          FrameStyle → None, FrameMargins → {{0, 0}, {2, 0}}]},
      AxesStyle → {Directive[Dashed, AbsoluteThickness[1.5]], Directive[Black]},
      Frame → True, FrameStyle → Directive[AbsoluteThickness[1.5]], FrameTicks →
        {{}, {{minVal, Style[ToString[minVal], FontFamily → "Courier", FontSize → ticksize],
            {0, 0.005}}, {0, Style[ToString[0], FontFamily → "Courier", FontSize → ticksize],
            {0, 0.005}}, {maxVal, Style[ToString[maxVal], FontFamily → "Courier",
             FontSize → ticksize], {0, 0.005}}}, xticks, {}},
      PlotRange → {{1, Length[arm]}, {Round[minVal, 10^-2], maxVal}},
      PlotRangePadding → None, PlotStyle →
        Map[{colorDefinitions[[#]], PointSize[0.002]} &, Range[Length[indices]]], PlotLabel →
        Framed[Style[Column[{Style["    " <> armName, FontSize → 16, FontFamily → "Courier"],
             letter <> "Tumor Arraylet"}, Spacings → 0.5], FontFamily → "Courier",
          FontSize → labelsize], FrameStyle → None, FrameMargins → {{0, 0}, {4, 0}}]],
     Graphics[{Black, Thickness[0.001], Map[Line[{{#[[1]], #[[3]]}, {#[[2]], #[[3]]}}] &,
         segments[[All, 6 ;; 8]]]}], If[armName == "6p+12p", Graphics[
        {Black, Dashed, Line[{{probes + 1, N[Round[minVal, 10^-2]]}, {probes + 1, maxVal}}]},
        Directive[AbsoluteThickness[1.5]]], {}], Graphics[{Arrowheads[0.006],
        Map[Arrow[{{#[[3]], #[[4]]}, {#[[1]], #[[2]]}}] &, genes[[2 ;;]]]}],
     Graphics[Map[Style[Text[#[[6]], {#[[3]] + #[[8]], #[[4]] + #[[5]]}], Symbol[#[[7]]],
          FontFamily → "courier", FontSize → labelsize] &, genes[[2 ;;]]]],
     ImageSize → {1014, 204}, ImagePadding → {{76, 50}, {5, 20}}];
   bandarraylet
  )]

(* Define the Tick Marks in the Arraylet Plot *)

xTicks[indices_] := Module[{xticks}, (
   xticks = Join[{{1, Style[Framed[ToString[1], FrameStyle → None,
          FrameMargins → {{Automatic, Automatic}, {0, 0.25}}], FontFamily → "Courier",
         FontSize → ticksize], {0, 0.005}}},
     Map[{indices[[# - 1]], Style[Framed[ToString[#], FrameStyle → None,
           FrameMargins → {{Automatic, Automatic}, {0, 2}}], FontFamily → "Courier",
          FontSize → ticksize], {0, 0.005}} &, Range[2, Length[indices]]]];
   xticks
  )]
```

```
(* Define the Tick Marks in the Tumor Raster *)

xTicksRaster[indices_, armBands_] := Module[{tickPosition, xticks}, (tickPosition =
     Map[If[# ≠ 1, Floor[(indices[[# - 1]] + 1) / step], 0] &, Range[Length[indices]]];
   xticks = Transpose[Join[{tickPosition}, {Map[Style[Framed[ToString[#], FrameStyle → None,
           FrameMargins → {{Automatic, Automatic}, {0, -1}}], FontFamily → "Courier",
          FontSize → ticksize] &, Range[Length[armBands] - 1]]},
      {Table[{0, 0.005}, {Length[armBands] - 1}]}]];
   xticks
  )]

(* Define the Tumor Raster *)

tumorRaster[tumor1_, tumor2_] :=
 Module[{tumorData, tumorProbes, binnedTumorData, bineedRows, arrays, raster,
   adjustedValue, binnedRows}, (tumorData = Median[Join[{tumor1}, {tumor2}]];
   tumorProbes = Dimensions[tumor1][[1]];
   binnedTumorData =
    Table[Mean[tumorData[[a ;; a + step - 1]]], {a, 1, Floor[tumorProbes, step], step}];
   {binnedRows, arrays} = Dimensions[binnedTumorData];
   raster = Table[adjustedValue = binnedTumorData[[a, b]];
     If[adjustedValue > 0, {adjustedValue, 0, 0}, {0, adjustedValue, 0}],
     {a, 1, binnedRows}, {b, 1, arrays}];
   raster[[All, All, 2]] = -raster[[All, All, 2]];
   raster = Transpose[raster * rastercontrast];
   raster
  )]

(* Define the Raster Display *)

rasterPlotDisplay[sortedRaster_, xticks_, letter_] := Module[{rasterFig}, (
   rasterFig = Graphics[Raster[sortedRaster, ColorFunction → RGBColor],
     ImageSize → {940, 320}, FrameLabel → {Framed[
         Column[{Style["Cytogenetic Bands", FontFamily → "Courier", FontSize → labelsize,
            FontTracking → "Plain"], Style[letter <> "Tumor Relative DNA Copy Number",
            FontFamily → "Courier", FontSize → labelsize, FontTracking → "Plain"]}, Center],
         FrameStyle → None, FrameMargins → {{0, 0}, {0, -5}}], None, None,
        Framed[Style["Patients", FontFamily → "Courier", FontSize → labelsize],
         FrameStyle → None, FrameMargins → {{0, 0}, {0, 1}}]},
     PlotRangePadding → None, ImagePadding → {{2, 49.5}, {52.5, 0}},
     AspectRatio → 0.3, Frame → True,
     FrameStyle → Directive[AbsoluteThickness[1.5]],
     FrameTicks → {xticks, None, None,
        Map[{#, Style[ToString[#], FontFamily → "Courier", FontSize → ticksize], {0, 0.005}} &,
         {1, 50, 100, 150, 200}]}];
   rasterFig
  )]
```

```
(* Define the Probelet Coefficient Classes and the Probelet Cutoff *)

probeletVariables[probelet_, probeletcutoff_] :=
 Module[{probeletCentered, scaledProbeletCutoff, indx, lowGroup, highGroup, minVal, maxVal},
  (probeletCentered = (probelet - Median[probelet]) / (MedianDeviation[probelet] * 1.4826);
   scaledProbeletCutoff = (probeletcutoff - Median[probelet]) /
     (MedianDeviation[probelet] * 1.4826);
   indx = Position[probeletCentered, _?(# ≤ scaledProbeletCutoff &)][[-1]][[1]];
   lowGroup = Transpose[Join[{probeletCentered[[1 ;; indx]]}, {Range[indx]}]];
   highGroup = Transpose[
     Join[{probeletCentered[[indx + 1 ;;]]}, {Range[indx + 1, Length[probeletCentered]]}]];
   minVal = Min[probeletCentered];
   maxVal = Max[probeletCentered];
   {lowGroup, highGroup, minVal, maxVal, scaledProbeletCutoff}
  )]

(* Define the Probelet Plot *)

probeletPlotDisplay[lowGroup_, highGroup_, xticks_, probeletCutoff_, letter_, armName_: ""] :=
 Module[{probeletFig}, (
   probeletFig = Show[ListPlot[{lowGroup, highGroup},
       Frame → True, FrameStyle → Directive[AbsoluteThickness[1.5]],
       FrameLabel → {Framed[Column[{Style["Relative DNA Copy Number in",
              FontFamily → "Courier", FontSize → labelsize, FontTracking → "Plain"],
             Style["Median Absolute Deviations", FontFamily → "Courier",
              FontSize → labelsize, FontTracking → "Plain"]}, Center],
          FrameStyle → None, FrameMargins → {{0, 0}, {0, -3}}],
         Framed[Style[letter <> "Probelet", FontFamily → "Courier", FontSize → labelsize],
          FrameStyle → None, FrameMargins → {{0, 0}, {1, 0}}], None, None, None},
       AspectRatio → 1.02, PlotStyle → {{If[armName ≠ "6p+12p", Blue, Red], PointSize[0.02]},
         {If[armName ≠ "6p+12p", Red, Blue], PointSize[0.02]}}, FrameTicks → {xticks,
         {{1, Style[ToString[1], FontFamily → "Courier", FontSize → ticksize], {0, 0.02}},
          {50, Style[ToString[50], FontFamily → "Courier", FontSize → ticksize], {0, 0.02}},
          {100, Style[ToString[100], FontFamily → "Courier", FontSize → ticksize], {0, 0.02}},
          {150, Style[ToString[150], FontFamily → "Courier", FontSize → ticksize], {0, 0.02}},
          {200, Style[ToString[200], FontFamily → "Courier", FontSize → ticksize], {0, 0.02}}},
         None, None}, AxesOrigin → {1, 0}, Axes → False,
       ImageSize → {316, 323}, ImagePadding → {{50, 0}, {54.5, 2}},
       PlotRange → {{Automatic, Automatic}, {1, 250}}],
      Graphics[{Dashed, AbsoluteThickness[1.5], Black,
        Line[{{probeletCutoff, 1}, {probeletCutoff, 249}}]}]];
   probeletFig
  )]
```

```
(* Define the Generalized Fractions *)

generalizedFractions[tumorCore_, normalCore_] := Module[{tumorFractions,
    normalFractions, totaltumorFractions, totalnormalFractions, entropy1, entropy2}, (
tumorFractions = tumorCore^2;
totaltumorFractions = Total[Flatten[tumorFractions]];
tumorFractions = tumorFractions / totaltumorFractions;
normalFractions = normalCore^2;
totalnormalFractions = Total[Flatten[normalFractions]];
normalFractions = normalFractions / totalnormalFractions;
    entropy1 = -N[Sum[tumorFractions[[p, q, r]] * Log[tumorFractions[[p, q, r]]],
          {p, 1, 498}, {q, 1, 249}, {r, 1, 2}] / Log[498 * 249 * 2]];
entropy1 = Round[entropy1, 0.01];
    entropy2 = -N[Sum[normalFractions[[p, q, r]] * Log[normalFractions[[p, q, r]]],
          {p, 1, 498}, {q, 1, 249}, {r, 1, 2}] / Log[498 * 249 * 2]];
entropy2 = Round[entropy2, 0.01];
    {tumorFractions, normalFractions, entropy1, entropy2}
   )]

(* Define the Fractions Display *)

barChartDisplay[fractions_, labelx_, labely_, xgrid_, xframe_, plotrange_] :=
 Module[{pos, vals, arrayNum, ylabels, barChart, framey, gridx, framex}, (
    gridx = xgrid;
    framex = xframe;
    pos = Map[Position[fractions, RankedMax[Flatten[fractions], #]] &, Range[1, 10]];
    pos = Flatten[pos];
    pos = Map[ToString[pos[[#]]] &, Range[Length[pos]]];
    pos = Map[StringJoin[Riffle[#, ","]] &, Partition[pos, 3]];
    vals = Map[RankedMax[Flatten[fractions], #] &, Range[1, 10]];
    arrayNum = 10;
    ylabels = Range[arrayNum];
    framex = Table[{gridx[[a]], Style[Rotate[framex[[a]], Pi / 2]]}, {a, 1, 6}];
    framey = Map[{11 - #, pos[[#]]} &, Reverse[Range[Length[pos]]]];
    framey = ReplaceAll[framey, "1,1,1" -> "  1,1,1"];
    gridx = Table[{gridx[[a]], RGBColor[0, 0, 0]}, {a, 1, 6}];
    barChart = Show[BarChart[Reverse[vals], BarOrigin → Left, ChartStyle → Red,
       ChartBaseStyle → EdgeForm[Thin], AspectRatio → 1, Ticks → None, Axes → None,
       ImageSize → 250], PlotRange → {plotrange, {0.5, arrayNum + 0.5}}, Frame → True,
     FrameTicks → {None, framey, framex, None}, FrameLabel → {None, labely, labelx, ""},
     GridLines → {Drop[Drop[gridx, {6}], {1}], None},
     BaseStyle → {FontFamily → "Courier", FontSize → 10}];
    barChart
   )]
```

```
(* Compute the 6p+12p Tensor GSVD *)

(* Read the 6p+12p Datasets *)

tumor1 = Import[path <> "D_Tumor_1_6p+12p.txt", "Table"];
tumor2 = Import[path <> "D_Tumor_2_6p+12p.txt", "Table"];
normal1 = Import[path <> "D_Normal_1_6p+12p.txt", "Table"];
normal2 = Import[path <> "D_Normal_2_6p+12p.txt", "Table"];
ArrayNames = tumor1[[1, 5 ;;]];
tumorProbeID = tumor1[[2 ;;, 1]];
tumorProbeLoc = tumor1[[2 ;;, 2 ;; 4]];
normalProbeID = normal1[[2 ;;, 1]];
normalProbeLoc = normal1[[2 ;;, 2 ;; 4]];
tumor1 = Drop[tumor1, 1, 4];
tumor2 = Drop[tumor2, 1, 4];
normal1 = Drop[normal1, 1, 4];
normal2 = Drop[normal2, 1, 4];

(* Compute the 6p+12p Tensor GSVD *)

{tumor1, tumor2, normal1, normal2, zTumorArraylets, zNormalArraylets, tumorCore,
   normalCore, xProbelets, yProbelets} = tGSVD[tumor1, tumor2, normal1, normal2];

labelsize = 14;
ticksize = 10;
colorDefinitions = Chop[Drop[Flatten[Table[{ColorConvert[Hue[a / 24], RGBColor],
       ColorConvert[Hue[(a + 12) / 24], RGBColor]}, {a, 10, -1, -1}]], {24}]];
colorDefinitions = Join[colorDefinitions, colorDefinitions];

(* Create the 6p+12p Generalized Fractions Display *)

{tumorFractions, normalFractions, entropy1, entropy2} =
  generalizedFractions[tumorCore, normalCore];
labelx = Column[{"Tumor Generalized Fraction",
     "(a) \!\(\*SubscriptBox[\(d\), \(1\)]\) = " <> ToString[entropy1]}, Center, 0.75];
labely = ColumnForm[{"6p+12p", "Subtensors"}, Center];
gridx = {0, 0.05, 0.1, 0.15, 0.2, 0.25};
framex = {"0", "0.05", "0.1", "0.15", "0.2", "0.25"};
plotrange = {0.0052, 0.25};
sixp12pTumorBarChart =
  barChartDisplay[tumorFractions, labelx, labely, gridx, framex, plotrange];
labelx = Column[{"Normal Generalized Fraction",
     "(b) \!\(\*SubscriptBox[\(d\), \(2\)]\) = " <> ToString[entropy2]}, Center, 0.75];
labely = "  ";
gridx = {0, 0.02, 0.04, 0.06, 0.08, 0.1};
framex = {"0", "0.02", "0.04", "0.06", "0.08", "0.1"};
plotrange = {0.0021, 0.1};
sixp12pNormalBarChart =
  barChartDisplay[normalFractions, labelx, labely, gridx, framex, plotrange];
```

```
(* Input the 6p+12p Chromosome Band Coordinates *)

{arrayletCentered, arrayletMedian, medianAbsoluteDeviations} =
  centeredArraylet[zTumorArraylets[[All, 1]]];
arm = Transpose[Join[Transpose[tumorProbeLoc], {arrayletCentered}]];
probes = Position[arm[[All, 1]], 6][[-1]][[1]];
arm = arm[[1 ;; probes]];
armBands = {{"#chrom", "chromStart", "chromEnd", "name"}, {"chr6", 0, 2 300 000, "p25.3"},
    {"chr6", 2 300 000, 4 200 000, "p25.2"}, {"chr6", 4 200 000, 7 100 000, "p25.1"},
    {"chr6", 7 100 000, 10 600 000, "p24.3"}, {"chr6", 10 600 000, 11 600 000, "p24.2"},
    {"chr6", 11 600 000, 13 400 000, "p24.1"}, {"chr6", 13 400 000, 15 200 000, "p23"},
    {"chr6", 15 200 000, 25 200 000, "p22.3"}, {"chr6", 25 200 000, 27 000 000, "p22.2"},
    {"chr6", 27 000 000, 30 400 000, "p22.1"}, {"chr6", 30 400 000, 32 100 000, "p21.33"},
    {"chr6", 32 100 000, 33 500 000, "p21.32"}, {"chr6", 33 500 000, 36 600 000, "p21.31"},
    {"chr6", 36 600 000, 40 500 000, "p21.2"}, {"chr6", 40 500 000, 46 200 000, "p21.1"},
    {"chr6", 46 200 000, 51 800 000, "p12.3"}, {"chr6", 51 800 000, 52 900 000, "p12.2"},
    {"chr6", 52 900 000, 57 000 000, "p12.1"}, {"chr6", 57 000 000, 58 700 000, "p11.2"},
    {"chr12", 0, 3 300 000, "p13.33"}, {"chr12", 3 300 000, 5 400 000, "p13.32"},
    {"chr12", 5 400 000, 10 100 000, "p13.31"}, {"chr12", 10 100 000, 12 800 000, "p13.2"},
    {"chr12", 12 800 000, 14 800 000, "p13.1"}, {"chr12", 14 800 000, 20 000 000, "p12.3"},
    {"chr12", 20 000 000, 21 300 000, "p12.2"}, {"chr12", 21 300 000, 26 500 000, "p12.1"},
    {"chr12", 26 500 000, 27 800 000, "p11.23"}, {"chr12", 27 800 000, 30 700 000, "p11.22"},
    {"chr12", 30 700 000, 33 300 000, "p11.21"}, {"chr12", 33 300 000, 35 800 000, "p11.1"}};

{subLists, minVal, maxVal, indices} = arrayletVariables[armBands[[1 ;; 20]], arm];

arm = Transpose[Join[Transpose[tumorProbeLoc], {arrayletCentered}]];
arm = arm[[Position[arm[[All, 1]], 12][[1]][[1]] ;;]];

{subLists1, minVal, maxVal, indices1} =
   arrayletVariables[Join[{armBands[[1]]}, armBands[[21 ;;]]], arm];

Map[(subLists1[[#]][[All, 1]] = subLists1[[#]][[All, 1]] + probes) &,
   Range[Length[subLists1]]];

subLists = Join[subLists, subLists1];
indices1 = indices1 + probes;
indices = Join[indices, indices1];
arm = Transpose[Join[Transpose[tumorProbeLoc], {arrayletCentered}]];
minVal = Round[Min[arm[[All, -1]]] - 3];
maxVal = Round[Max[arm[[All, -1]]]];

(* Input the 6p+12p Segments′ and Genes′ Coordinates *)

segments = armSegments["6p+12p", arrayletMedian, medianAbsoluteDeviations];
genes = {{"xcoord", "ycoord", "xcoord1", "ycoord1", "Displacement", "gene-name", "color",
    "xDisplacement"}, {3986.5, -0.0043, 3986.5, -0.01, -0.0015, "MIR877", "Blue", 0},
   {4137, -0.0043, 4137, 0.008, 0.0012, "TNF", "Red", 0},
   {4904, -0.0043, 4904, 0.008, 0.0012, "MAPK14", "Red", 0},
   {5023.5, -0.0043, 5023.5, -0.01, -0.0015, "CDKN1A", "Red", 0},
   {8216.5, -0.0003, 8476.5, -0.0003, 0, "PRIM2", "Black", 350},
   {9097.5, 0.0118, 9097.5, 0.019, 0.0012, "RAD51AP1", "Red", 0},
   {9521.2, 0.0118, 9521.2, -0.01, -0.0015, "MIR200C/141", "Blue", 0},
   {12 074.5, 0.0216, 12 614.5, 0.0216, 0, "KRAS", "Black", 300},
   {10 320.5, 0.0105, 10 320.5, 0.0025, -0.0015, "CDKN1B", "Red", 0},
   {11 777, 0.0188, 11 377, 0.0188, 0, "SOX5", "Red", -300},
   {12 268.5, 0.0175, 12 268.5, 0.003, -0.002, "ITPR2", "Red", 0},
   {12 345, 0.016, 12 645, 0.016, 0, "ASUN", "Red", 280}};
genes[[2 ;;, 2]] = (genes[[2 ;;, 2]] - arrayletMedian) / medianAbsoluteDeviations;
genes[[2 ;;, 4]] = (genes[[2 ;;, 4]] - arrayletMedian) / medianAbsoluteDeviations;
scaledDisplacement = genes[[2 ;;, 5]] * 500;
genes[[2 ;;, 5]] = scaledDisplacement;
```

```
(* Create the 6p+12p Arraylet Plot Display *)

xticks = Join[{{1, Style[Framed[ToString[1], FrameStyle → None,
       FrameMargins → {{Automatic, Automatic}, {0, 0.25}}],
      FontFamily → "Courier", FontSize → ticksize], {0, 0.005}}},
   Map[{indices[[# - 1]], Style[Framed[ToString[#], FrameStyle → None,
        FrameMargins → {{Automatic, Automatic}, {0, 2}}],
       FontFamily → "Courier", FontSize → ticksize], {0, 0.005}} &, Range[2, 19]],
   {{probes + 1, Style[Framed[ToString[1], FrameStyle → None,
       FrameMargins → {{Automatic, Automatic}, {0, 0.25}}],
      FontFamily → "Courier", FontSize → ticksize], {0, 0.005}}},
   Map[{indices1[[# - 1]], Style[Framed[ToString[#], FrameStyle → None,
        FrameMargins → {{Automatic, Automatic}, {0, 2}}], FontFamily → "Courier",
       FontSize → ticksize], {0, 0.005}} &, Range[2, Length[indices1]]]];
xticks[[17]][[2]] = Style[Framed["17  ", FrameStyle → None,
     FrameMargins → {{Automatic, Automatic}, {0, 2}}],
   FontFamily → "Courier", FontSize → ticksize];
xticks[[18]][[2]] =
  Style[Framed["  18", FrameStyle → None, FrameMargins → {{Automatic, Automatic}, {0, 2}}],
   FontFamily → "Courier", FontSize → ticksize];
xticks[[19]][[2]] =
  Style[Framed["19  ", FrameStyle → None, FrameMargins → {{Automatic, Automatic}, {0, 2}}],
   FontFamily → "Courier", FontSize → ticksize];
xticks[[20]][[2]] =
  Style[Framed["  1", FrameStyle → None, FrameMargins → {{Automatic, Automatic}, {0, 2}}],
   FontFamily → "Courier", FontSize → ticksize];

gArraylet = arrayletPlotDisplay[arm, subLists, xticks, minVal, maxVal,
   indices, segments, genes, "6p+12p", "(a)  ", colorDefinitions, probes];

(* Create the 6p+12p Tumor Datasets Raster Display *)

step = 5;
rastercontrast = 1;
sortedRaster = tumorRaster[tumor1, tumor2];

xticks = xTicksRaster[indices, armBands];
xticks[[17]][[2]] =
  Style[Framed["17  ", FrameStyle → None, FrameMargins → {{Automatic, Automatic}, {0, -1}}],
   FontFamily → "Courier", FontSize → ticksize];
xticks[[18]][[2]] =
  Style[Framed["  18", FrameStyle → None, FrameMargins → {{Automatic, Automatic}, {0, -1}}],
   FontFamily → "Courier", FontSize → ticksize];
xticks[[19]][[2]] =
  Style[Framed["19  ", FrameStyle → None, FrameMargins → {{Automatic, Automatic}, {0, -1}}],
   FontFamily → "Courier", FontSize → ticksize];
xticks[[20]][[2]] =
  Style[Framed["  1", FrameStyle → None, FrameMargins → {{Automatic, Automatic}, {0, -1}}],
   FontFamily → "Courier", FontSize → ticksize];
Map[(xticks[[#]][[2]] = Style[Framed[ToString[# - 19], FrameStyle → None,
        FrameMargins → {{Automatic, Automatic}, {0, -1}}],
       FontFamily → "Courier", FontSize → ticksize];) &, Range[21, 31]];

gRaster = rasterPlotDisplay[sortedRaster, xticks, "(c)  "];
```

```
(* Classify the Patients into High and Low 6p+12p Probelet Coefficients *)

probeletCutoff = 0.050337629778934495;
{lowGroup, highGroup, minVal, maxVal, probeletCutoff} =
  probeletVariables[xProbelets[[1]], probeletCutoff];

xticks = {{-8.5, Style[Framed["-8.5", FrameStyle → None, FrameMargins → {{0, 0}, {0, -4.5}}],
      FontFamily → "Courier", FontSize → ticksize], {0, 0.02}},
    {-6.5, Style[Framed["-6.5", FrameStyle → None, FrameMargins → {{0, 0}, {0, -4.5}}],
      FontFamily → "Courier", FontSize → ticksize], {0, 0.02}},
    {-1.5, Style[Framed["-1.5", FrameStyle → None, FrameMargins → {{0, 0}, {0, -4.5}}],
      FontFamily → "Courier", FontSize → ticksize], {0, 0.02}},
    {-1, Style[Framed["-1", FrameStyle → None, FrameMargins → {{0, 0}, {0, -4.5}}],
      FontFamily → "Courier", FontSize → ticksize], {0, 0.02}},
    {-0.5, Style[Framed["-0.5  ", FrameStyle → None, FrameMargins → {{0, 0}, {0, -4.5}}],
      FontFamily → "Courier", FontSize → ticksize, FontTracking → "Narrow"], {0, 0.02}},
    {probeletCutoff, Style[Framed[" " <> ToString[N[Round[probeletCutoff, 10^-1]]],
       FrameStyle → None, FrameMargins → {{0, 0}, {0, -4.5}}], FontFamily → "Courier",
      FontSize → ticksize, FontTracking → "Narrow"], {0, 0.02}}, {2.5, Style[Framed[
       ToString[2.5], FrameStyle → None, FrameMargins → {{Automatic, Automatic}, {0, -4.5}}],
      FontFamily → "Courier", FontSize → ticksize], {0, 0.02}},
    {2, Style[Framed["2", FrameStyle → None, FrameMargins → {{0, 0}, {0, -4.5}}],
      FontFamily → "Courier", FontSize → ticksize], {0, 0.02}},
    {1.5, Style[Framed["1.5", FrameStyle → None, FrameMargins → {{0, 0}, {0, -4.5}}],
      FontFamily → "Courier", FontSize → ticksize], {0, 0.02}},
    {0, Style[Framed["0", FrameStyle → None, FrameMargins → {{0, 0}, {0, -4.5}}],
      FontFamily → "Courier", FontSize → ticksize], {0, 0.02}},
    {3, Style[Framed["3", FrameStyle → None, FrameMargins → {{0, 0}, {0, -4.5}}],
      FontFamily → "Courier", FontSize → ticksize], {0, 0.02}},
    {1, Style[Framed["1", FrameStyle → None, FrameMargins → {{0, 0}, {0, -4.5}}],
      FontFamily → "Courier", FontSize → ticksize], {0, 0.02}}};

(* Create the 6p+12p Probelet Display *)

gProbelet = probeletPlotDisplay[lowGroup, highGroup, xticks, probeletCutoff, "(b)  ", "6p+12p"];

(* Combine the 6p+12p Probelet, Tumor Arraylet, and Tumor Datasets Displays *)

figure1 = Grid[{{gArraylet, SpanFromLeft}, {gProbelet, gRaster}},
   Alignment → {{Right}, {Left, Right}}, Spacings → {2 → 0.2, 2 → 0}];
```

```
(* Compute the 7p Tensor GSVD *)

(* Read the 7p Datasets *)

tumor1 = Import[path <> "D_Tumor_1_7p.txt", "Table"];
tumor2 = Import[path <> "D_Tumor_2_7p.txt", "Table"];
normal1 = Import[path <> "D_Normal_1_7p.txt", "Table"];
normal2 = Import[path <> "D_Normal_2_7p.txt", "Table"];
ArrayNames = tumor1[[1, 5 ;;]];
tumorProbeID = tumor1[[2 ;;, 1]];
tumorProbeLoc = tumor1[[2 ;;, 2 ;; 4]];
normalProbeID = normal1[[2 ;;, 1]];
normalProbeLoc = normal1[[2 ;;, 2 ;; 4]];
tumor1 = Drop[tumor1, 1, 4];
tumor2 = Drop[tumor2, 1, 4];
normal1 = Drop[normal1, 1, 4];
normal2 = Drop[normal2, 1, 4];

(* Compute the 7p Tensor GSVD *)

{tumor1, tumor2, normal1, normal2, zTumorArraylets, zNormalArraylets, tumorCore,
   normalCore, xProbelets, yProbelets} = tGSVD[tumor1, tumor2, normal1, normal2];

labelsize = 14;
ticksize = 10;
colorDefinitions = Chop[Drop[Flatten[Table[{ColorConvert[Hue[a / 24], RGBColor],
       ColorConvert[Hue[(a + 12) / 24], RGBColor]}, {a, 10, -1, -1}]], {24}]];
colorDefinitions = Join[colorDefinitions, colorDefinitions];

(* Create the 7p Generalized Fractions Display *)

{tumorFractions, normalFractions, entropy1, entropy2} =
  generalizedFractions[tumorCore, normalCore];
gridx = {0, 0.02, 0.04, 0.06, 0.08, 0.1};
framex = {"0", "0.02", "0.04", "0.06", "0.08", "0.1"};
plotrange = {0.0021, 0.1};
labelx =
  ColumnForm[{"(c) \!\(\*SubscriptBox[\(d\), \(1\)]\) = " <> ToString[entropy1]}, Center];
labely = ColumnForm[{"7p", "Subtensors"}, Center];
sevenpTumorBarChart =
  barChartDisplay[tumorFractions, labelx, labely, gridx, framex, plotrange];
gridx = {0.0001, 0.01, 0.02, 0.03, 0.04, 0.05};
framex = {"0", "0.01", "0.02", "0.03", "0.04", "0.05"};
labelx =
  ColumnForm[{"(d) \!\(\*SubscriptBox[\(d\), \(2\)]\) = " <> ToString[entropy2]}, Center];
labely = " ";
plotrange = {0.00112, 0.05};
sevenpNormalBarChart =
  barChartDisplay[normalFractions, labelx, labely, gridx, framex, plotrange];
```

```
(* Input the 7p Chromosome Band Coordinates *)

{arrayletCentered, arrayletMedian, medianAbsoluteDeviations} =
 centeredArraylet[zTumorArraylets[[All, 1]]];
arm = Transpose[Join[Transpose[tumorProbeLoc], {arrayletCentered}]];
armBands = {{"#chrom", "chromStart", "chromEnd", "name"}, {"chr7", 0, 2 800 000, "p22.3"},
   {"chr7", 2 800 000, 4 500 000, "p22.2"}, {"chr7", 4 500 000, 7 300 000, "p22.1"},
   {"chr7", 7 300 000, 13 800 000, "p21.3"}, {"chr7", 13 800 000, 16 500 000, "p21.2"},
   {"chr7", 16 500 000, 20 900 000, "p21.1"}, {"chr7", 20 900 000, 25 500 000, "p15.3"},
   {"chr7", 25 500 000, 28 000 000, "p15.2"}, {"chr7", 28 000 000, 28 800 000, "p15.1"},
   {"chr7", 28 800 000, 35 000 000, "p14.3"}, {"chr7", 35 000 000, 37 200 000, "p14.2"},
   {"chr7", 37 200 000, 43 300 000, "p14.1"}, {"chr7", 43 300 000, 45 400 000, "p13"},
   {"chr7", 45 400 000, 49 000 000, "p12.3"}, {"chr7", 49 000 000, 50 500 000, "p12.2"},
   {"chr7", 50 500 000, 54 000 000, "p12.1"}, {"chr7", 54 000 000, 58 000 000, "p11.2"}};

{subLists, minVal, maxVal, indices} = arrayletVariables[armBands, arm];

(* Input the 7p Segments' and Genes' Coordinates *)

segments = armSegments[7, arrayletMedian, medianAbsoluteDeviations];
genes = {{"xcoord", "ycoord", "xcoord1", "ycoord1", "Displacement", "gene-name", "color",
   "xDisplacement"}, {698.5, -0.0211, 698.5, 0.008, 0.001, "RPA3", "Red", 30},
  {5472, 0.0066, 5472, -0.01, -0.0015, "POLD2", "Red", 30}};
genes[[2 ;;, 2]] = (genes[[2 ;;, 2]] - arrayletMedian) / medianAbsoluteDeviations;
genes[[2 ;;, 4]] = (genes[[2 ;;, 4]] - arrayletMedian) / medianAbsoluteDeviations;
scaledDisplacement = genes[[2 ;;, 5]] * 500;
genes[[2 ;;, 5]] = scaledDisplacement;

(* Create the 7p Arraylet Plot Display *)

xticks = xTicks[indices];

gArraylet = arrayletPlotDisplay[arm, subLists, xticks,
   minVal, maxVal, indices, segments, genes, "7p", "(d)  ", colorDefinitions];

(* Create the 7p Tumor Datasets Raster Display *)

step = 5;
rastercontrast = 1;
sortedRaster = tumorRaster[tumor1, tumor2];

xticks = xTicksRaster[indices, armBands];

gRaster = rasterPlotDisplay[sortedRaster, xticks, "(f)  "];
```

```
(* Classify the Patients into High and Low 7p Probelet Coefficients *)

probeletCutoff = 0.003947059214898124;
{lowGroup, highGroup, minVal, maxVal, probeletCutoff} =
  probeletVariables[xProbelets[[1]], probeletCutoff];

(* Create the 7p Probelet Display *)

xticks = {{-2.5, Style[Framed["-2.5", FrameStyle → None, FrameMargins → {{0, 0}, {0, -4.5}}],
      FontFamily → "Courier", FontSize → ticksize], {0, 0.02}},
    {-0.5, Style[Framed["-0.5   ", FrameStyle → None, FrameMargins → {{0, 0}, {0, -4.5}}],
      FontFamily → "Courier", FontSize → ticksize,
      FontTracking → "Narrow"], {0, 0.02}}, {probeletCutoff,
     Style[Framed["  " <> ToString[N[Round[probeletCutoff, 10^-1]]], FrameStyle → None,
       FrameMargins → {{0, 0}, {0, -4.5}}], FontFamily → "Courier",
      FontSize → ticksize, FontTracking → "Narrow"], {0, 0.02}},
    {2.5, Style[Framed[ToString[2.5], FrameStyle → None,
       FrameMargins → {{Automatic, Automatic}, {0, -4.5}}],
      FontFamily → "Courier", FontSize → ticksize], {0, 0.02}},
    {4.5, Style[Framed["4.5", FrameStyle → None, FrameMargins → {{0, 0}, {0, -4.5}}],
      FontFamily → "Courier", FontSize → ticksize], {0, 0.02}},
    {6.5, Style[Framed["6.5", FrameStyle → None, FrameMargins → {{0, 0}, {0, -4.5}}],
      FontFamily → "Courier", FontSize → ticksize], {0, 0.02}}};

gProbelet = probeletPlotDisplay[lowGroup, highGroup, xticks, probeletCutoff, "(e)  "];

(* Combine the 7p Probelet, Tumor Arraylet, and Tumor Datasets Displays *)

figure2 = Grid[{{gArraylet, SpanFromLeft}, {gProbelet, gRaster}},
   Alignment → {{Right}, {Left, Right}}, Spacings → {2 → 0.2, 2 → 0}];
```

```
(* Compute the Xq Tensor GSVD *)
```

```
(* Read the Xq Datasets *)

tumor1 = Import[path <> "D_Tumor_1_Xq.txt", "Table"];
tumor2 = Import[path <> "D_Tumor_2_Xq.txt", "Table"];
normal1 = Import[path <> "D_Normal_1_Xq.txt", "Table"];
normal2 = Import[path <> "D_Normal_2_Xq.txt", "Table"];
ArrayNames = tumor1[[1, 5 ;;]];
tumorProbeID = tumor1[[2 ;;, 1]];
tumorProbeLoc = tumor1[[2 ;;, 2 ;; 4]];
normalProbeID = normal1[[2 ;;, 1]];
normalProbeLoc = normal1[[2 ;;, 2 ;; 4]];
tumor1 = Drop[tumor1, 1, 4];
tumor2 = Drop[tumor2, 1, 4];
normal1 = Drop[normal1, 1, 4];
normal2 = Drop[normal2, 1, 4];

(* Compute the Xq Tensor GSVD *)

{tumor1, tumor2, normal1, normal2, zTumorArraylets, zNormalArraylets, tumorCore,
   normalCore, xProbelets, yProbelets} = tGSVD[tumor1, tumor2, normal1, normal2];

labelsize = 14;
ticksize = 10;
colorDefinitions = Chop[Drop[Flatten[Table[{ColorConvert[Hue[a / 24], RGBColor],
       ColorConvert[Hue[(a + 12) / 24], RGBColor]}, {a, 10, -1, -1}]], {24}]];
colorDefinitions = Join[colorDefinitions, colorDefinitions];

(* Create the Xq Generalized Fractions Display *)

{tumorFractions, normalFractions, entropy1, entropy2} =
  generalizedFractions[tumorCore, normalCore];
gridx = {0, 0.02, 0.04, 0.06, 0.08, 0.1};
framex = {"0", "0.02", "0.04", "0.06", "0.08", "0.1"};
plotrange = {0.0021, 0.1};
labelx =
  ColumnForm[{"(e) \!\(\*SubscriptBox[\(d\), \(1\)]\) = " <> ToString[entropy1]}, Center];
labely = ColumnForm[{"Xq", "Subtensors"}, Center];
xqTumorBarChart = barChartDisplay[tumorFractions, labelx, labely, gridx, framex, plotrange];
labelx =
  ColumnForm[{"(f) \!\(\*SubscriptBox[\(d\), \(2\)]\) = " <> ToString[entropy2]}, Center];
labely = " ";
xqNormalBarChart =
  barChartDisplay[normalFractions, labelx, labely, gridx, framex, plotrange];
```

```
(* Input the Xq Chromosome Band Coordinates *)

{arrayletCentered, arrayletMedian, medianAbsoluteDeviations} =
 centeredArraylet[zTumorArraylets[[All, 1]]];
arm = Transpose[Join[Transpose[tumorProbeLoc], {arrayletCentered}]];
armBands = {{"#chrom", "chromStart", "chromEnd", "name"},
   {"chrX", 60 600 000, 63 000 000, "q11.1"}, {"chrX", 63 000 000, 64 600 000, "q11.2"},
   {"chrX", 64 600 000, 67 800 000, "q12"}, {"chrX", 67 800 000, 71 800 000, "q13.1"},
   {"chrX", 71 800 000, 73 900 000, "q13.2"}, {"chrX", 73 900 000, 76 000 000, "q13.3"},
   {"chrX", 76 000 000, 84 600 000, "q21.1"}, {"chrX", 84 600 000, 86 200 000, "q21.2"},
   {"chrX", 86 200 000, 91 800 000, "q21.31"}, {"chrX", 91 800 000, 93 500 000, "q21.32"},
   {"chrX", 93 500 000, 98 300 000, "q21.33"}, {"chrX", 98 300 000, 102 600 000, "q22.1"},
   {"chrX", 102 600 000, 103 700 000, "q22.2"}, {"chrX", 103 700 000, 108 700 000, "q22.3"},
   {"chrX", 108 700 000, 116 500 000, "q23"}, {"chrX", 116 500 000, 120 900 000, "q24"},
   {"chrX", 120 900 000, 128 700 000, "q25"}, {"chrX", 128 700 000, 130 400 000, "q26.1"},
   {"chrX", 130 400 000, 133 600 000, "q26.2"}, {"chrX", 133 600 000, 138 000 000, "q26.3"},
   {"chrX", 138 000 000, 140 300 000, "q27.1"}, {"chrX", 140 300 000, 142 100 000, "q27.2"},
   {"chrX", 142 100 000, 147 100 000, "q27.3"}, {"chrX", 147 100 000, 155 270 560, "q28"}};

{subLists, minVal, maxVal, indices} = arrayletVariables[armBands, arm];

(* Input the Xq Segments' and Genes' Coordinates *)

segments = armSegments["Xq", arrayletMedian, medianAbsoluteDeviations];
genes = {{"xcoord", "ycoord", "xcoord1", "ycoord1", "Displacement", "gene-name", "color",
   "xDisplacement"}, {1063.5, -0.0064, 1063.5, 0.012, 0.0007, "PABPC5", "Red", 0},
  {3569.5, 0.027, 3569.5, 0.01, -0.001, "MIR888", "Blue", 0},
  {3882.6, 0.027, 3882.5, 0.036, 0.00035, "MIR224/452", "Blue", 0},
  {3996.5, 0.027, 3996.5, 0.01, -0.001, "BCAP31", "Red", 0}};
genes[[2 ;;, 2]] = (genes[[2 ;;, 2]] - arrayletMedian) / medianAbsoluteDeviations;
genes[[2 ;;, 4]] = (genes[[2 ;;, 4]] - arrayletMedian) / medianAbsoluteDeviations;
scaledDisplacement = genes[[2 ;;, 5]] * 500;
genes[[2 ;;, 5]] = scaledDisplacement;

(* Create the Xq Arraylet Plot *)

xticks = xTicks[indices];
xticks[[10]][[2]] =
  Style[Framed["10 ", FrameStyle → None, FrameMargins → {{Automatic, Automatic}, {0, 2}}],
   FontFamily → "Courier", FontSize → ticksize];
xticks[[11]][[2]] = Style[Framed[" 11", FrameStyle → None, FrameMargins →
     {{Automatic, Automatic}, {0, 2}}], FontFamily → "Courier", FontSize → ticksize];
xticks[[22]][[2]] = Style[Framed["22 ", FrameStyle → None, FrameMargins →
     {{Automatic, Automatic}, {0, 2}}], FontFamily → "Courier", FontSize → ticksize];
xticks[[23]][[2]] = Style[Framed[" 23", FrameStyle → None, FrameMargins →
     {{Automatic, Automatic}, {0, 2}}], FontFamily → "Courier", FontSize → ticksize];

gArraylet = arrayletPlotDisplay[arm, subLists, xticks,
   minVal, maxVal, indices, segments, genes, "Xq", "(g) ", colorDefinitions];
```

```
(* Create the Xq Tumor Datasets Raster Display *)

step = 5;
rastercontrast = 1;
sortedRaster = tumorRaster[tumor1, tumor2];

xticks = xTicksRaster[indices, armBands];
xticks[[10]][[2]] =
  Style[Framed["10 ", FrameStyle → None, FrameMargins → {{Automatic, Automatic}, {0, -1}}],
   FontFamily → "Courier", FontSize → ticksize];
xticks[[11]][[2]] = Style[Framed[" 11", FrameStyle → None, FrameMargins →
      {{Automatic, Automatic}, {0, -1}}], FontFamily → "Courier", FontSize → ticksize];
xticks[[22]][[2]] = Style[Framed["22 ", FrameStyle → None, FrameMargins →
      {{Automatic, Automatic}, {0, -1}}], FontFamily → "Courier", FontSize → ticksize];
xticks[[23]][[2]] = Style[Framed[" 23", FrameStyle → None, FrameMargins →
      {{Automatic, Automatic}, {0, -1}}], FontFamily → "Courier", FontSize → ticksize];

gRaster = rasterPlotDisplay[sortedRaster, xticks, "(i)  "];

(* Classify the Patients into High and Low Xq Probelet Coefficients *)

probeletCutoff = 0.005459355777945107;
{lowGroup, highGroup, minVal, maxVal, probeletCutoff} =
  probeletVariables[xProbelets[[1]], probeletCutoff];

xticks = {{-8.5, Style[Framed["-8.5", FrameStyle → None, FrameMargins → {{0, 0}, {0, -4.5}}],
      FontFamily → "Courier", FontSize → ticksize], {0, 0.02}},
    {-6.5, Style[Framed["-6.5", FrameStyle → None, FrameMargins → {{0, 0}, {0, -4.5}}],
      FontFamily → "Courier", FontSize → ticksize], {0, 0.02}},
    {-4.5, Style[Framed["-4.5", FrameStyle → None, FrameMargins → {{0, 0}, {0, -4.5}}],
      FontFamily → "Courier", FontSize → ticksize], {0, 0.02}},
    {-2.5, Style[Framed["-2.5", FrameStyle → None, FrameMargins → {{0, 0}, {0, -4.5}}],
      FontFamily → "Courier", FontSize → ticksize], {0, 0.02}},
    {-0.5, Style[Framed["-0.5   ", FrameStyle → None, FrameMargins → {{0, 0}, {0, -4.5}}],
      FontFamily → "Courier", FontSize → ticksize, FontTracking → "Narrow"], {0, 0.02}},
    {probeletCutoff, Style[Framed["  " <> ToString[N[Round[probeletCutoff, 10^-1]]],
       FrameStyle → None, FrameMargins → {{0, 0}, {0, -4.5}}], FontFamily → "Courier",
      FontSize → ticksize, FontTracking → "Narrow"], {0, 0.02}}, {2.5, Style[Framed[
       ToString[2.5], FrameStyle → None, FrameMargins → {{Automatic, Automatic}, {0, -4.5}}],
      FontFamily → "Courier", FontSize → ticksize], {0, 0.02}},
    {4.5, Style[Framed["4.5", FrameStyle → None, FrameMargins → {{0, 0}, {0, -4.5}}],
      FontFamily → "Courier", FontSize → ticksize], {0, 0.02}},
    {6.5, Style[Framed["6.5", FrameStyle → None, FrameMargins → {{0, 0}, {0, -4.5}}],
      FontFamily → "Courier", FontSize → ticksize], {0, 0.02}}};

(* Create the Xq Probelet Display *)

gProbelet = probeletPlotDisplay[lowGroup, highGroup, xticks, probeletCutoff, "(h)  "];

(* Combine the Xq Probelet, Tumor Arraylet, and Tumor Datasets Displays *)

figure3 = Grid[{{gArraylet, SpanFromLeft}, {gProbelet, gRaster}},
   Alignment → {{Right}, {Left, Right}}, Spacings → {2 → 0.2, 2 → 0}];
```

```
(* Display Probelets, Tumor Arraylets, and Tumor Datasets *)
```

```
fig2 = Column[{figure1, figure2, figure3}, Spacings → 1]
```

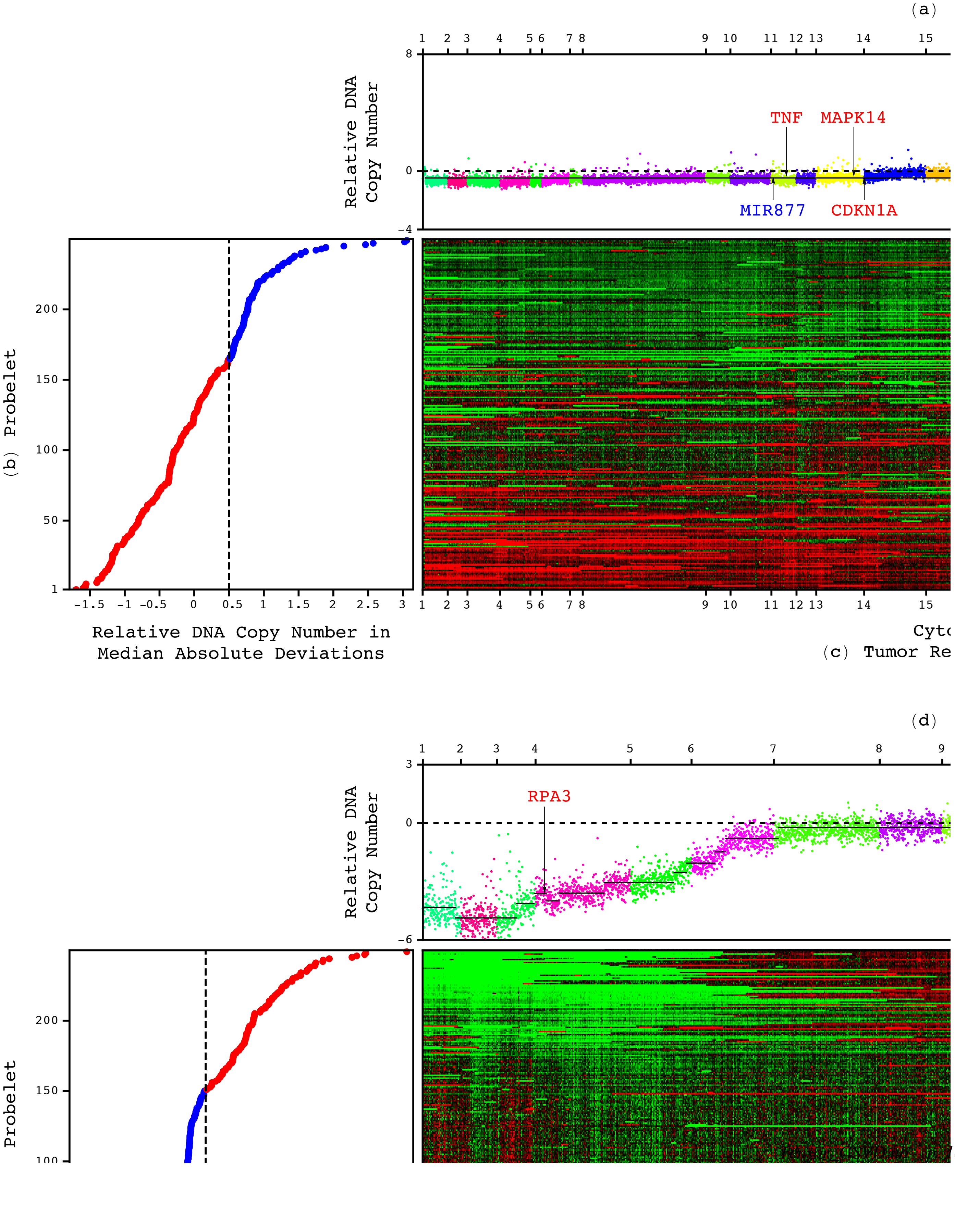

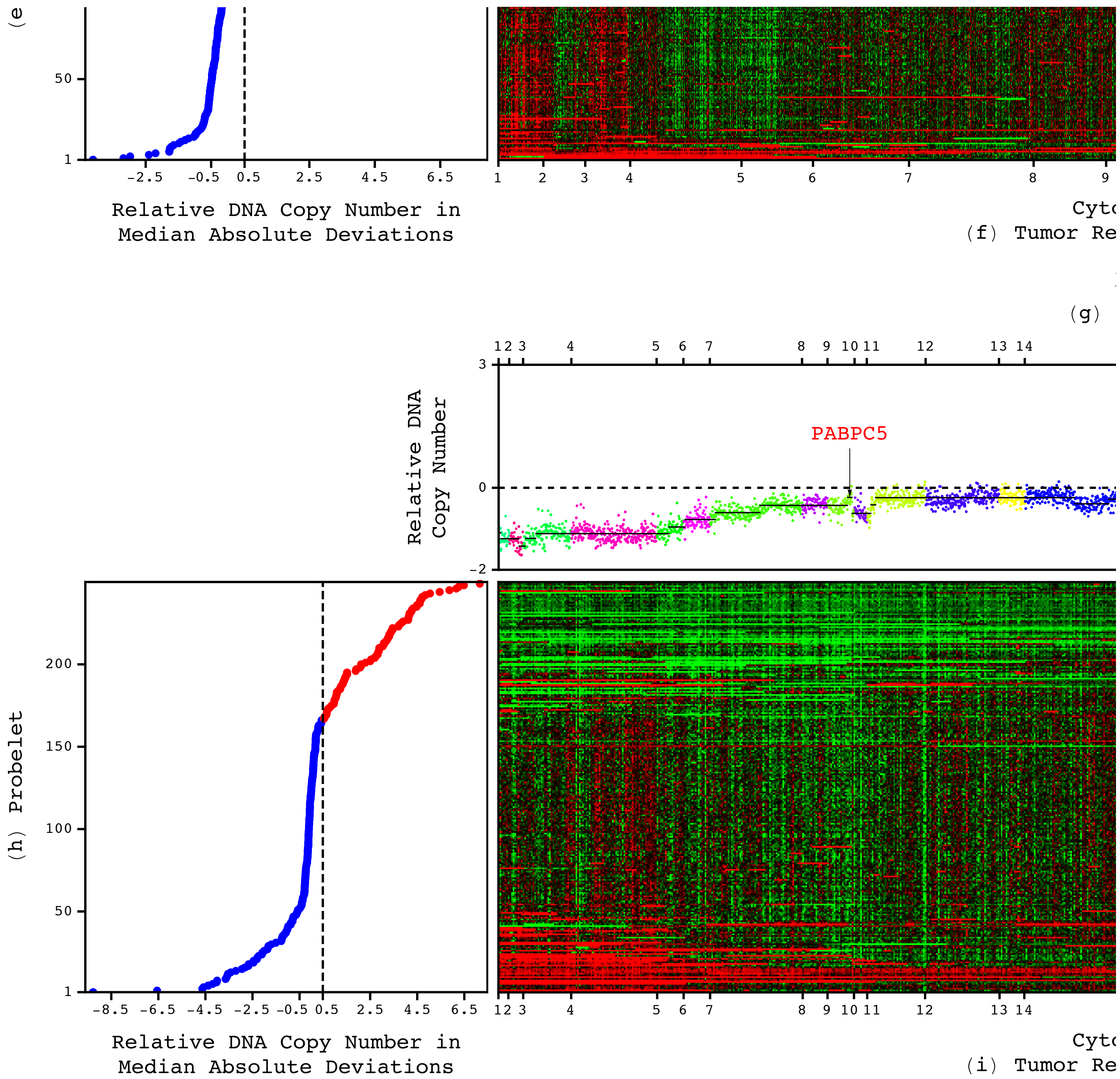


```
Export[path <> "Figure_2.pdf", fig2, "PDF"];
```

```
(* Display the Tumor and Normal Generalized Fractions Bar Chart Display *)
```

```
figC = GraphicsGrid[{{sixp12pTumorBarChart, sixp12pNormalBarChart},
   {sevenpTumorBarChart, sevenpNormalBarChart}, {xqTumorBarChart, xqNormalBarChart}},
  ImageSize → 500, Spacings → {-15, {0, -20, -27, 0}}]
```

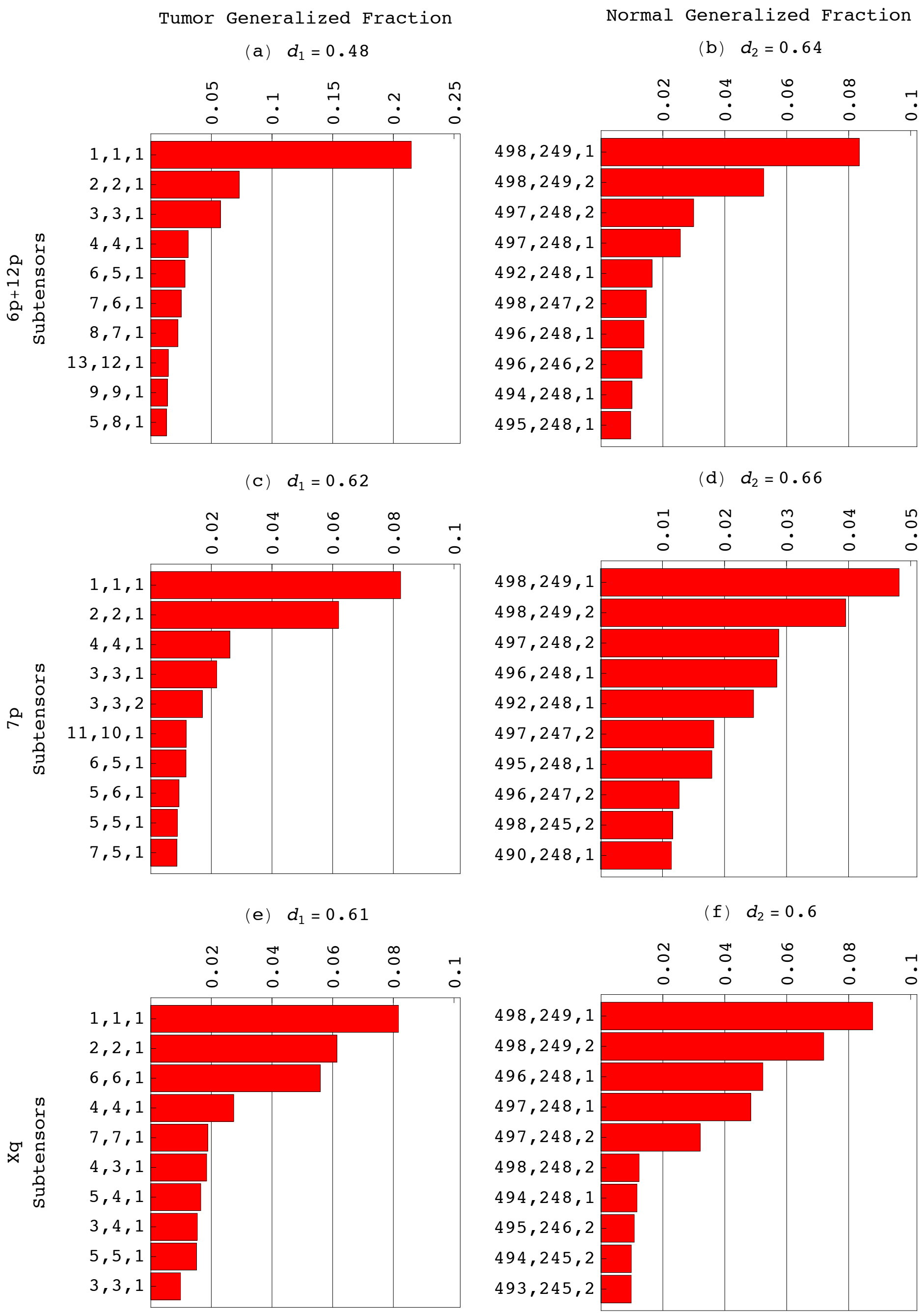


```
Export[path <> "Figure_C.pdf", figC, "PDF", ImageSize → 500];
```

```
(* Compute and Display Kaplan-Meier Survival Curves and Log-Rank Test P-Values *)

monthConversion = (365.25 / 12);
Clear[framex, framey, labelx, labely, xlabel, ylabel];

(* Read Survival and Classification Data *)

annotationColumn[annotation_] :=
  Transpose[annotations][[Position[annotationNames, annotation][[1, 1]]]];
options[annotation_] := Intersection[annotationColumn[annotation]];
optionNumbers[annotation_] := Dimensions[options[annotation]][[1]];
optionCounts[annotation_] :=
  Table[Count[annotationColumn[annotation], options[annotation][[a]]],
   {a, 1, optionNumbers[annotation]}];

(* Identify the Classification Groups *)

order[annotation_] := Sort[optionCounts[annotation], Greater];
first[annotation_] := Position[options[annotation], firstAnnotation][[1]][[1]];
second[annotation_] := Position[options[annotation], secondAnnotation][[1]][[1]];
third[annotation_] := Position[options[annotation], thirdAnnotation][[1]][[1]];
fourth[annotation_] := Position[options[annotation], fourthAnnotation][[1]][[1]];
groups[annotation_] :=
  Sort[Transpose[Join[{annotationColumn[annotation]}, {times}, { status}]]];
optionCountsPosition[annotation_] :=
  Join[{0}, Table[Sum[optionCounts[annotation][[a]], {a, 1, b}],
    {b, 1, Dimensions[optionCounts[annotation]][[1]]}]];
firstGroup[annotation_] := Select[groups[annotation], #[[1]] == firstAnnotation &];
secondGroup[annotation_] := Select[groups[annotation], #[[1]] == secondAnnotation &];
thirdGroup[annotation_] := Select[groups[annotation], #[[1]] == thirdAnnotation &];
fourthGroup[annotation_] := Select[groups[annotation], #[[1]] == fourthAnnotation &];
```

```
(* Evaluate the Kaplan-Meier Survival Curves of the Classification Groups *)

evaluateFirst[annotation_] := {
  nFirstGroup =
   optionCounts[annotation][[Position[options[annotation], firstAnnotation][[1]][[1]]]];
  oFirstGroup = nFirstGroup;
  group = Transpose[firstGroup[annotation]][[2]];
  Do[If[firstGroup[annotation][[a, 3]] == 0, group = Drop[group, {a}]],
   {a, nFirstGroup, 1, -1}];
  firstMedian = 0;
  firstLine = {{0, 1}};
  firstLines = {RGBColor[0, 0, 1], Thickness[0.0075]};
  y = 1;
  Do[
   If[firstGroup[annotation][[a, 3]] == 0,
    {oFirstGroup = oFirstGroup - 1;
     firstLines = Join[firstLines, {Line[{{firstGroup[annotation][[a, 2]], y + 0.02},
          {firstGroup[annotation][[a, 2]], y - 0.02}}]}];
     If[a == optionCounts[annotation][[first[annotation]]],
      firstLine = Join[firstLine, {{firstGroup[annotation][[a, 2]], y}}]]},
    {firstLine = Join[firstLine, {{firstGroup[annotation][[a, 2]], y}}];
     y = y * (nFirstGroup - a) / (nFirstGroup - a + 1);
     If[firstMedian == 0, If[y ≤ 0.5, firstMedian = Round[firstGroup[annotation][[a, 2]]]]];
     firstLine = Join[firstLine, {{firstGroup[annotation][[a, 2]], y}}]}],
   {a, 1, optionCounts[annotation][[first[annotation]]]}];
  If[firstMedian == 0, firstMedian =
    Round[firstGroup[annotation][[optionCounts[annotation][[first[annotation]]], 2]]]];
  firstLine = Graphics[{RGBColor[0, 0, 1], Thickness[0.0075], Line[firstLine]}];
  firstLines = Graphics[firstLines];
  textFirstGroup = Graphics[Text[
     Style[ColumnForm[{
        StringReplace[options[annotation][[first[annotation]]], "_" → " "],
        StringJoin["N=", ToString[nFirstGroup]],
        StringJoin["O=", ToString[oFirstGroup]]},
       Center], {RGBColor[0, 0, 1], FontFamily → "Courier"}],
     textCoordinates[[1]]]]}
```

```
evaluateSecond[annotation_] := {
  nSecondGroup =
   optionCounts[annotation][[Position[options[annotation], secondAnnotation][[1]][[1]]]];
  oSecondGroup = nSecondGroup;
  group = Transpose[secondGroup[annotation]][[2]];
  Do[If[secondGroup[annotation][[a, 3]] == 0, group = Drop[group, {a}]],
   {a, nSecondGroup, 1, -1}];
  secondMedian = 0;
  secondLine = {{0, 1}};
  secondLines = {RGBColor[1, 0, 0], Thickness[0.0075]};
  y = 1;
  Do[
   If[secondGroup[annotation][[a, 3]] == 0,
    {oSecondGroup = oSecondGroup - 1;
     secondLines = Join[secondLines, {Line[{{secondGroup[annotation][[a, 2]], y + 0.02},
          {secondGroup[annotation][[a, 2]], y - 0.02}}]}];
     If[a == optionCounts[annotation][[second[annotation]]],
      secondLine = Join[secondLine, {{secondGroup[annotation][[a, 2]], y}}]]},
    {secondLine = Join[secondLine, {{secondGroup[annotation][[a, 2]], y}}];
     y = y * (nSecondGroup - a) / (nSecondGroup - a + 1);
     If[secondMedian == 0,
      If[y ≤ 0.5, secondMedian = Round[secondGroup[annotation][[a, 2]]]]];
     secondLine = Join[secondLine, {{secondGroup[annotation][[a, 2]], y}}]}],
   {a, 1, optionCounts[annotation][[second[annotation]]]}];
  If[secondMedian == 0, secondMedian =
    Round[secondGroup[annotation][[optionCounts[annotation][[second[annotation]]], 2]]]];
  secondLine = Graphics[{RGBColor[1, 0, 0], Thickness[0.0075], Line[secondLine]}];
  secondLines = Graphics[secondLines];
  textSecondGroup = Graphics[
    Text[Style[ColumnForm[{
        StringReplace[options[annotation][[second[annotation]]], "_" → " "],
        StringJoin["N=", ToString[nSecondGroup]],
        StringJoin["O=", ToString[oSecondGroup]]},
       Center], {RGBColor[1, 0, 0], FontFamily → "Courier"}],
     textCoordinates[[2]]]]}
```

```
evaluateThird[annotation_, displayNumber_] :=
 If[displayNumber ≥ 3, {
   nThirdGroup =
    optionCounts[annotation][[Position[options[annotation], thirdAnnotation][[1]][[1]]]];
   oThirdGroup = nThirdGroup;
   group = Transpose[thirdGroup[annotation]][[2]];
   Do[If[thirdGroup[annotation][[a, 3]] == 0, group = Drop[group, {a}]],
    {a, nThirdGroup, 1, -1}];
   thirdMedian = 0;
   thirdLine = {{0, 1}};
   thirdLines = {RGBColor[0, 0.5, 0], Thickness[0.0075]};
   y = 1;
   Do[
    If[thirdGroup[annotation][[a, 3]] == 0,
     {oThirdGroup = oThirdGroup - 1;
      thirdLines = Join[thirdLines, {Line[{{thirdGroup[annotation][[a, 2]], y + 0.02},
           {thirdGroup[annotation][[a, 2]], y - 0.02}}]}];
      If[a == optionCounts[annotation][[third[annotation]]],
       thirdLine = Join[thirdLine, {{thirdGroup[annotation][[a, 2]], y}}]]},
     {thirdLine = Join[thirdLine, {{thirdGroup[annotation][[a, 2]], y}}];
      y = y * (nThirdGroup - a) / (nThirdGroup - a + 1);
      If[thirdMedian == 0, If[y ≤ 0.5, thirdMedian = Round[thirdGroup[annotation][[a, 2]]]]];
      thirdLine = Join[thirdLine, {{thirdGroup[annotation][[a, 2]], y}}]}],
    {a, 1, optionCounts[annotation][[third[annotation]]]}];
   If[thirdMedian == 0, thirdMedian =
     Round[thirdGroup[annotation][[optionCounts[annotation][[third[annotation]]], 2]]]];
   thirdLine = Graphics[{RGBColor[0, 0.5, 0], Thickness[0.0075], Line[thirdLine]}];
   thirdLines = Graphics[thirdLines];
   textThirdGroup = Graphics[
     Text[Style[ColumnForm[{
          StringReplace[options[annotation][[third[annotation]]], "_" → " "],
          StringJoin["N=", ToString[nThirdGroup]],
          StringJoin["O=", ToString[oThirdGroup]]},
         Center], {RGBColor[0, 0.5, 0], FontFamily → "Courier"}],
      textCoordinates[[3]]]]},
  {thirdLine = Graphics[];
   thirdLines = Graphics[];
   textThirdGroup = Graphics[]}]
```

```
evaluateFourth[annotation_, displayNumber_] :=
 If[displayNumber ≥ 4, {
   nFourthGroup =
    optionCounts[annotation][[Position[options[annotation], fourthAnnotation][[1]][[1]]]];
   oFourthGroup = nFourthGroup;
   group = Transpose[fourthGroup[annotation]][[2]];
   Do[If[fourthGroup[annotation][[a, 3]] == 0, group = Drop[group, {a}]],
    {a, nFourthGroup, 1, -1}];
   fourthMedian = 0;
   fourthLine = {{0, 1}};
   fourthLines = {RGBColor[0.75, 0, 1], Thickness[0.0075]};
   y = 1;
   Do[
    If[fourthGroup[annotation][[a, 3]] == 0,
     {oFourthGroup = oFourthGroup - 1;
      fourthLines = Join[fourthLines, {Line[{{fourthGroup[annotation][[a, 2]], y + 0.02},
           {fourthGroup[annotation][[a, 2]], y - 0.02}}]}];
      If[a == optionCounts[annotation][[fourth[annotation]]],
       fourthLine = Join[fourthLine, {{fourthGroup[annotation][[a, 2]], y}}]]},
     {fourthLine = Join[fourthLine, {{fourthGroup[annotation][[a, 2]], y}}];
      y = y * (nFourthGroup - a) / (nFourthGroup - a + 1);
      If[fourthMedian == 0,
       If[y ≤ 0.5, fourthMedian = Round[fourthGroup[annotation][[a, 2]]]]];
      fourthLine = Join[fourthLine, {{fourthGroup[annotation][[a, 2]], y}}]}],
    {a, 1, optionCounts[annotation][[fourth[annotation]]]}];
   If[fourthMedian == 0, fourthMedian =
     Round[fourthGroup[annotation][[optionCounts[annotation][[fourth[annotation]]], 2]]]];
   fourthLine = Graphics[{RGBColor[0.75, 0, 1], Thickness[0.0075], Line[fourthLine]}];
   fourthLines = Graphics[fourthLines];
   textFourthGroup = Graphics[Text[
      Style[ColumnForm[{
         StringReplace[options[annotation][[fourth[annotation]]], "_" → " "],
         StringJoin["N=", ToString[nFourthGroup]],
         StringJoin["O=", ToString[oFourthGroup]]},
        Center], {RGBColor[0.75, 0, 1], FontFamily → "Courier"}],
      textCoordinates[[4]]]]},
  {fourthLine = Graphics[];
   fourthLines = Graphics[];
   textFourthGroup = Graphics[]}]
```

```
(* Evaluate the Log-Rank Test P-value *)

evaluatePValue[annotation_] := {
   statistics = Sort[Transpose[Join[{times}, {annotationColumn[annotation]}, {status}]]];
   firstObservations = Table[If[
      statistics[[a, 2]] == options[annotation][[first[annotation]]], statistics[[a, 3]], 0],
     {a, 1, patients}];
   firstEvents = Table[If[statistics[[a, 2]] == options[annotation][[first[annotation]]],
      ReplaceAll[statistics[[a, 3]], 0 → 1], 0],
     {a, 1, patients}];
   secondObservations = Table[If[statistics[[a, 2]] ==
       options[annotation][[second[annotation]]], statistics[[a, 3]], 0],
     {a, 1, patients}];
   secondEvents = Table[If[statistics[[a, 2]] == options[annotation][[second[annotation]]],
      ReplaceAll[statistics[[a, 3]], 0 → 1], 0],
     {a, 1, patients}];
   Do[If[statistics[[a, 1]] == statistics[[a - 1, 1]], {
      firstObservations[[a - 1]] = firstObservations[[a - 1]] + firstObservations[[a]];
      firstObservations = Drop[firstObservations, {a}];
      firstEvents[[a - 1]] = firstEvents[[a - 1]] + firstEvents[[a]];
      firstEvents = Drop[firstEvents, {a}];
      secondObservations[[a - 1]] = secondObservations[[a - 1]] + secondObservations[[a]];
      secondObservations = Drop[secondObservations, {a}];
      secondEvents[[a - 1]] = secondEvents[[a - 1]] + secondEvents[[a]];
      secondEvents = Drop[secondEvents, {a}]
     }], {a, patients, 2, -1}];
   timesNumbers = Dimensions[firstObservations][[1]];
   firstNumbers =
    Table[Total[firstEvents] - Total[Take[firstEvents, 1 ;; a - 1]], {a, 1, timesNumbers}];
   secondNumbers = Table[Total[secondEvents] - Total[Take[secondEvents, 1 ;; a - 1]],
     {a, 1, timesNumbers}];
   observations = firstObservations + secondObservations;
   numbers = firstNumbers + secondNumbers;
   firstExpectations = Table[If[numbers[[a]] == 0, 0,
      N[observations[[a]] * firstNumbers[[a]] / numbers[[a]]]],
     {a, 1, timesNumbers}];
   secondExpectations = Table[If[numbers[[a]] == 0, 0,
      N[observations[[a]] * secondNumbers[[a]] / numbers[[a]]]],
     {a, 1, timesNumbers}];
   variances = Table[If[numbers[[a]] ≤ 1, 0,
      N[firstNumbers[[a]] * secondNumbers[[a]] * observations[[a]] *
        (numbers[[a]] - observations[[a]]) / numbers[[a]] ^2 / (numbers[[a]] - 1)]],
     {a, 1, timesNumbers}];
   z = (N[Total[firstObservations - firstExpectations]]) ^2 / N[Total[variances]];
   pValue = 1 - CDF[ChiSquareDistribution[1], {z}][[1]]};
```

```
(* Create the Kaplan-Meier Survival Curves *)

w = 0.0075;
months = 100;
framey[ylabel_] := ReplaceAll[ReplaceAll[
    Table[{a, Style[If[ylabel == "False", "     ", ToString[a]], FontFamily → "Courier"]},
     {a, 0, 1, 0.25}], "1." → "1"], "0." → "0"];
labelx[xlabel_] := If[xlabel == True, Style["Survival Time (Months)",
    FontFamily → "Courier"], ""];
plotlabelx[annotation_, xlabel_, columnLabel_] :=
  Style[ColumnForm[
    Which[hazardRatio == "blank", {Style[StringJoin["    ", columnLabel], FontSize → 14],
      StringJoin[xlabel, "  ", StringReplace[StringReplace[StringReplace[
          StringReplace[annotation, "_6p+12p" → ""], "_7p" → ""], "_Xq" → ""], "_" → "  "]],
      StringJoin["     P-value = ", ToString[TraditionalForm[
         ScientificForm[pValue, 2, NumberPoint →
           If[Dimensions[Characters[ToString[NumberForm[pValue, 2, NumberFormat → (#1 &),
                  ExponentFunction → (# &)]]]]][[1]] < 3, ".0", "."]]
        ]]], "\n"},
     hazardRatio == "", {Style[StringJoin["    ", columnLabel], FontSize → 14],
      StringJoin[xlabel, "  ", StringReplace[StringReplace[StringReplace[
          StringReplace[annotation, "_6p+12p" → ""], "_7p" → ""], "_Xq" → ""], "_" → "  "]],
      StringJoin["     P-value = ", ToString[TraditionalForm[
         ScientificForm[pValue, 2, NumberPoint →
           If[Dimensions[Characters[ToString[NumberForm[pValue, 2, NumberFormat → (#1 &),
                  ExponentFunction → (# &)]]]]][[1]] < 3, ".0", "."]]
        ]]]},
     True, {Style[StringJoin["    ", columnLabel], FontSize → 14],
      StringJoin[xlabel, "  ", StringReplace[StringReplace[StringReplace[
          StringReplace[annotation, "_6p+12p" → ""], "_7p" → ""], "_Xq" → ""], "_" → "  "]],
      StringJoin["     P-value = ", ToString[TraditionalForm[
         ScientificForm[pValue, 2, NumberPoint →
           If[Dimensions[Characters[ToString[NumberForm[pValue, 2, NumberFormat → (#1 &),
                  ExponentFunction → (# &)]]]]][[1]] < 3, ".0", "."]]
        ]]], StringJoin["     Hazard Ratio", hazardRatio]}], Left],
   FontFamily → "Courier", FontSize → 12];
```

```
labely[ylabel_] :=
  If[ylabel == "False", Style[ColumnForm[{" ", " "}, Center], FontFamily → "Courier"],
   Style[ColumnForm[{title, ylabel}, Center], FontFamily → "Courier"]];
display[annotation_, displayNumber_, xplotlabel_,
   xlabel_, ylabel_, medianTicks_, highlight_, columnLabel_] := {
   {firstMedian, secondMedian, thirdMedian, fourthMedian} = Table[0, {4}];
   evaluateFirst[annotation];
   evaluateSecond[annotation];
   evaluateThird[annotation, displayNumber];
   evaluateFourth[annotation, displayNumber];
   framex = If[displayNumber == 2,
     {{0, Style["0", FontFamily → "Courier"]}, {80, Style["80", FontFamily → "Courier"]},
      {firstMedian, If[firstMedian == secondMedian, "",
        Style[StringJoin[medianTicks[[1]], ToString[firstMedian], medianTicks[[2]]],
         {RGBColor[0, 0, 1], FontFamily → "Courier"}]]},
      {secondMedian, Style[StringJoin[medianTicks[[3]], ToString[secondMedian],
         medianTicks[[4]]], {RGBColor[1, 0, 0], FontFamily → "Courier"}]}},
     {{0, Style["0", FontFamily → "Courier"]},
      If[76 < firstMedian < 84 || 76 < secondMedian < 84 || 76 < thirdMedian < 84 ||
        76 < fourthMedian < 84, {0, Style["0", FontFamily → "Courier"]},
       {80, Style["80", FontFamily → "Courier"]}], If[fourthMedian ≠ 0,
       {fourthMedian, Style[StringJoin[medianTicks[[7]], If[fourthMedian == firstMedian,
           "", If[fourthMedian == thirdMedian, "", ToString[fourthMedian]]],
          medianTicks[[8]]], {RGBColor[0.75, 0, 1], FontFamily → "Courier"}]},
       {fourthMedian, Style[StringJoin[medianTicks[[7]], If[fourthMedian == firstMedian,
           "", If[fourthMedian == thirdMedian, "", ""]], medianTicks[[8]]],
         {RGBColor[0.75, 0, 1], FontFamily → "Courier"}]}],
      If[thirdMedian ≠ 0, {thirdMedian, Style[StringJoin[medianTicks[[5]], ToString[
           thirdMedian], medianTicks[[6]]], {RGBColor[0, 0.5, 0], FontFamily → "Courier"}]},
       {thirdMedian, Style[StringJoin[medianTicks[[5]], "", medianTicks[[6]]],
         {RGBColor[0, 0.5, 0], FontFamily → "Courier"}]}],
      If[secondMedian ≠ 0, {secondMedian, Style[StringJoin[medianTicks[[3]], ToString[
           secondMedian], medianTicks[[4]]], {RGBColor[1, 0, 0], FontFamily → "Courier"}]},
       {secondMedian, Style[StringJoin[medianTicks[[3]], "", medianTicks[[4]]],
         {RGBColor[1, 0, 0], FontFamily → "Courier"}]}],
      If[firstMedian ≠ 0, {firstMedian, Style[StringJoin[medianTicks[[1]], ToString[
           firstMedian], medianTicks[[2]]], {RGBColor[0, 0, 1], FontFamily → "Courier"}]},
       {firstMedian, Style[StringJoin[medianTicks[[1]], "", medianTicks[[2]]],
         {RGBColor[0, 0, 1], FontFamily → "Courier"}]}]
     }];
   If[displayNumber == 2, evaluatePValue[annotation]];
   Show[{Graphics[If[highlight == True, {RGBColor[1, 1, 0.8], Rectangle[{
          Min[Select[{firstMedian, secondMedian, thirdMedian, fourthMedian}, # ≠ 0 &]], -0.025},
          Max[
           Select[{firstMedian, secondMedian, thirdMedian, fourthMedian}, # ≠ 0 &]], 1.025}]},
       {}]],
     fourthLine, fourthLines, textFourthGroup,
     thirdLine, thirdLines, textThirdGroup,
     firstLine, firstLines, textFirstGroup,
     secondLine, secondLines, textSecondGroup},
    GridLines → {None, {{0.5, Thickness[0.0025]}}},
    Frame → True,
    FrameTicks → {framex, framey[ylabel], None, None},
    FrameLabel → {labelx[xlabel], labely[ylabel], None, None},
    PlotLabel → plotlabelx[annotation, xplotlabel, columnLabel],
    AspectRatio → 1,
    PlotRange → {{-0.1 * months, 1.025 * months}, {-0.025, 1.025}},
    ImageSize → 250]}
```

```
(* Display the Survival Analyses of the Discovery and Validation Sets of Patients Classified by the
Tensor GSVD *)

title = "Fraction of Surviving Patients";

stream = path <> "Discovery_Patients.txt";
annotations = Import[stream, "Table"];
{patients, annotationsNumber} = Dimensions[annotations] - {1, 1};
annotationNames = Take[annotations, 1, {2, annotationsNumber + 1}][[1]];
annotations = Drop[Import[stream, "Table"], 1, 1];
Clear[stream];

times = Table[
   If[annotationColumn["Days_Death"][[a]] == "Null",
    If[annotationColumn["Days_Followup"][[a]] == "Null",
     "Null",
     annotationColumn["Days_Followup"][[a]] / monthConversion],
    annotationColumn["Days_Death"][[a]] / monthConversion],
   {a, 1, patients}];
status = Table[If[annotationColumn["Days_Death"][[a]] == "Null",
    If[annotationColumn["Days_Followup"][[a]] == "Null", 2, 0], 1],
   {a, 1, patients}];
positionNull = Position[status, 2];
Do[{
  annotations = Drop[annotations, positionNull[[a]]],
  times = Drop[times, positionNull[[a]]],
  status = Drop[status, positionNull[[a]]]},
 {a, Dimensions[positionNull][[1]], 1, -1}]
patients = patients - Dimensions[positionNull][[1]];

displayNumber = 2;
annotation = "Probelet_6p+12p_(Coeff.)";
firstAnnotation = "High";
secondAnnotation = "Low";
highlight = True;
xplotlabel = "(a)";
xlabel = False;
ylabel = "from the Discovery Set";
medianTicks = Table["", {a, 1, 8}];
hazardRatio = " = 1.7";
columnLabel = "6p+12p";
textCoordinates = {{0.09 * months, 0.1}, {0.865 * months, 0.9}};
g1 = Show[display[annotation, displayNumber, xplotlabel,
    xlabel, ylabel, medianTicks, highlight, columnLabel], ImageSize → 250];

displayNumber = 2;
annotation = "Probelet_7p_(Coeff.)";
firstAnnotation = "Low";
secondAnnotation = "High";
highlight = True;
xplotlabel = "(b)";
xlabel = False;
ylabel = "False";
medianTicks = Table["", {a, 1, 8}];
hazardRatio = " = 1.7";
columnLabel = "7p";
textCoordinates = {{0.09 * months, 0.1}, {0.865 * months, 0.9}};
g2 = Show[display[annotation, displayNumber, xplotlabel,
    xlabel, ylabel, medianTicks, highlight, columnLabel], ImageSize → 250];
```

```
displayNumber = 2;
annotation = "Probelet_Xq_(Coeff.)";
firstAnnotation = "Low";
secondAnnotation = "High";
highlight = True;
xplotlabel = "(c)";
xlabel = False;
ylabel = "False";
medianTicks = Table["", {a, 1, 8}];
hazardRatio = " = 1.6";
columnLabel = "Xq";
textCoordinates = {{0.09 * months, 0.1}, {0.865 * months, 0.9}};
g3 = Show[display[annotation, displayNumber, xplotlabel,
     xlabel, ylabel, medianTicks, highlight, columnLabel], ImageSize → 250];

displayNumber = 4;
annotation = "Probelet_6p+12p/Tumor_Stage";
firstAnnotation = "High/III,IV";
secondAnnotation = "Low/I,II";
thirdAnnotation = "Low/III,IV";
fourthAnnotation = "High/I,II";
highlight = True;
xplotlabel = "(d)";
xlabel = False;
ylabel = "from the Discovery Set";
medianTicks = Table["", {a, 1, 8}];
medianTicks[[2]] = "  ";
medianTicks[[6]] = "  ";
medianTicks[[7]] = "   ";
hazardRatio = "s = 1.5/4.0";
pValue = 4.2 * 10^-4;
columnLabel = "";
textCoordinates =
  {{0.09 * months, 0.1}, {0.865 * months, 0.9}, {0.09 * months, 0.35}, {0.09 * months, 0.6}};
g4 = Show[display[annotation, displayNumber, xplotlabel, xlabel,
     ylabel, medianTicks, highlight, columnLabel], ImageSize → 250];

displayNumber = 4;
annotation = "Probelet_7p/Tumor_Stage";
firstAnnotation = "Low/III,IV";
secondAnnotation = "High/I,II";
thirdAnnotation = "High/III,IV";
fourthAnnotation = "Low/I,II";
highlight = True;
xplotlabel = "(e)";
xlabel = False;
ylabel = "False";
medianTicks = Table["", {a, 1, 8}];
medianTicks[[4]] = "  ";
medianTicks[[7]] = "  ";
hazardRatio = "s = 1.6/4.2";
pValue = 1.1 * 10^-4;
columnLabel = "";
textCoordinates =
  {{0.09 * months, 0.1}, {0.865 * months, 0.9}, {0.09 * months, 0.35}, {0.09 * months, 0.6}};
g5 = Show[display[annotation, displayNumber, xplotlabel, xlabel,
     ylabel, medianTicks, highlight, columnLabel], ImageSize → 250];
```

```
displayNumber = 4;
annotation = "Probelet_Xq/Tumor_Stage";
firstAnnotation = "Low/III,IV";
secondAnnotation = "High/I,II";
thirdAnnotation = "High/III,IV";
fourthAnnotation = "Low/I,II";
highlight = True;
xplotlabel = "(f)";
xlabel = False;
ylabel = "False";
medianTicks = Table["", {a, 1, 8}];
hazardRatio = "blank";
pValue = 8.3 * 10^-4;
columnLabel = "";
textCoordinates =
  {{0.09 * months, 0.1}, {0.865 * months, 0.9}, {0.09 * months, 0.35}, {0.09 * months, 0.6}};
g6 = Show[display[annotation, displayNumber, xplotlabel, xlabel,
     ylabel, medianTicks, highlight, columnLabel], ImageSize → 250];

stream = path <> "Validation_Patients.txt";
annotations = Import[stream, "Table"];
{patients, annotationsNumber} = Dimensions[annotations] - {1, 1};
annotationNames = Take[annotations, 1, {2, annotationsNumber + 1}][[1]];
annotations = Drop[Import[stream, "Table"], 1, 1];
Clear[stream];

times = Table[
    If[annotationColumn["Days_Death"][[a]] == "Null",
     If[annotationColumn["Days_Followup"][[a]] == "Null",
      "Null",
      annotationColumn["Days_Followup"][[a]] / monthConversion],
     annotationColumn["Days_Death"][[a]] / monthConversion],
    {a, 1, patients}];
status = Table[If[annotationColumn["Days_Death"][[a]] == "Null",
     If[annotationColumn["Days_Followup"][[a]] == "Null", 2, 0], 1],
    {a, 1, patients}];
positionNull = Position[status, 2];
Do[{
   annotations = Drop[annotations, positionNull[[a]]],
   times = Drop[times, positionNull[[a]]],
   status = Drop[status, positionNull[[a]]]},
  {a, Dimensions[positionNull][[1]], 1, -1}]
patients = patients - Dimensions[positionNull][[1]];

displayNumber = 2;
annotation = "Arraylet_6p+12p_(Corr.)";
firstAnnotation = "High";
secondAnnotation = "Low";
highlight = True;
xplotlabel = "(g)";
xlabel = True;
ylabel = "from the Validation Set";
medianTicks = Table["", {a, 1, 8}];
hazardRatio = "=1.9";
columnLabel = "";
textCoordinates = {{0.09 * months, 0.1}, {0.865 * months, 0.9}};
g7 = Show[display[annotation, displayNumber, xplotlabel,
     xlabel, ylabel, medianTicks, highlight, columnLabel], ImageSize → 250];
```

```
displayNumber = 2;
annotation = "Arraylet_7p_(Corr.)";
firstAnnotation = "Low";
secondAnnotation = "High";
highlight = True;
xplotlabel = "(h)";
xlabel = True;
ylabel = "False";
medianTicks = Table["", {a, 1, 8}];
hazardRatio = "=1.7";
columnLabel = "";
textCoordinates = {{0.09 * months, 0.1}, {0.865 * months, 0.9}};
g8 = Show[display[annotation, displayNumber, xplotlabel,
     xlabel, ylabel, medianTicks, highlight, columnLabel], ImageSize → 250];

displayNumber = 2;
annotation = "Arraylet_Xq_(Corr.)";
firstAnnotation = "Low";
secondAnnotation = "High";
highlight = True;
xplotlabel = "(i)";
xlabel = True;
ylabel = "False";
medianTicks = Table["", {a, 1, 8}];
hazardRatio = "=1.9";
columnLabel = "";
textCoordinates = {{0.09 * months, 0.1}, {0.865 * months, 0.9}};
g9 = Show[display[annotation, displayNumber, xplotlabel,
     xlabel, ylabel, medianTicks, highlight, columnLabel], ImageSize → 250];

fig3 =
 GraphicsGrid[{{g1, g2, g3}, {g4, g5, g6}, {g7, g8, g9}}, ImageSize → 750, Spacings → {-67, -12}]
```

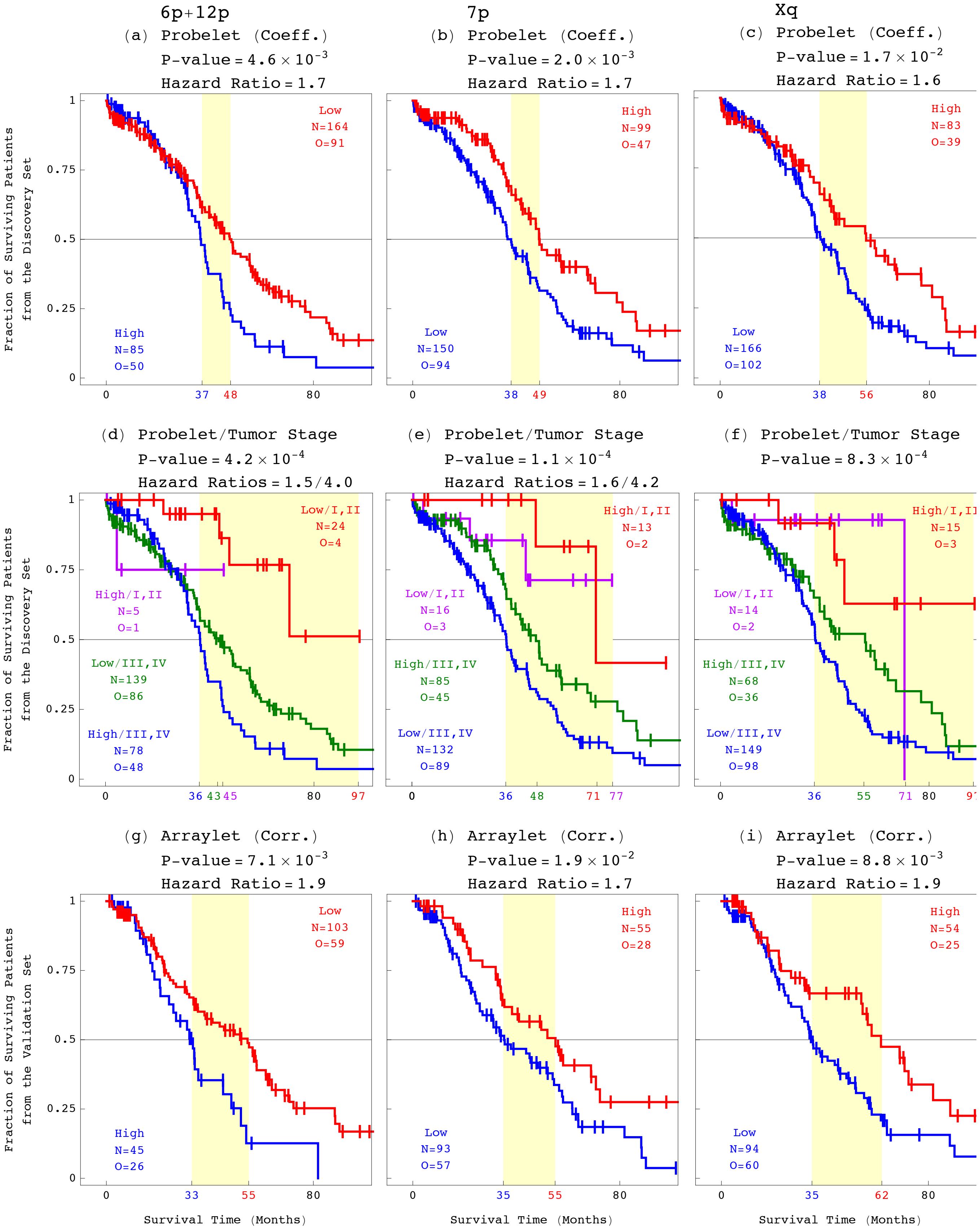


```
Export[path <> "Figure_3.pdf", fig3, "PDF", ImageSize → 750, ImageResolution → resolution];
```

```
(* Display the Survival Analyses of the Discovery Set Classified by the Standard Indicators *)

title = "Fraction of Surviving Patients";

stream = path <> "Discovery_Patients.txt";
annotations = Import[stream, "Table"];
{patients, annotationsNumber} = Dimensions[annotations] - {1, 1};
annotationNames = Take[annotations, 1, {2, annotationsNumber + 1}][[1]];
annotations = Drop[Import[stream, "Table"], 1, 1];
Clear[stream];

times = Table[
   If[annotationColumn["Days_Death"][[a]] == "Null",
    If[annotationColumn["Days_Followup"][[a]] == "Null",
     "Null",
     annotationColumn["Days_Followup"][[a]] / monthConversion],
    annotationColumn["Days_Death"][[a]] / monthConversion],
   {a, 1, patients}];
status = Table[If[annotationColumn["Days_Death"][[a]] == "Null",
    If[annotationColumn["Days_Followup"][[a]] == "Null", 2, 0], 1],
   {a, 1, patients}];
positionNull = Position[status, 2];
Do[{
  annotations = Drop[annotations, positionNull[[a]]],
  times = Drop[times, positionNull[[a]]],
  status = Drop[status, positionNull[[a]]]},
 {a, Dimensions[positionNull][[1]], 1, -1}]
patients = patients - Dimensions[positionNull][[1]];

displayNumber = 2;
annotation = "Tumor_Stage";
firstAnnotation = "III,IV";
secondAnnotation = "I,II";
highlight = True;
xplotlabel = "(a)";
xlabel = False;
ylabel = "from the Discovery Set";
medianTicks = Table["", {a, 1, 8}];
hazardRatio = " = 4.4";
columnLabel = "";
textCoordinates = {{0.09 * months, 0.1}, {0.865 * months, 0.9}};
g1 = Show[display[annotation, displayNumber, xplotlabel,
    xlabel, ylabel, medianTicks, highlight, columnLabel], ImageSize → 250];

displayNumber = 2;
annotation = "Residual_Disease";
firstAnnotation = "Yes";
secondAnnotation = "No";
highlight = True;
xplotlabel = "(b)";
xlabel = False;
ylabel = "False";
medianTicks = Table["", {a, 1, 8}];
hazardRatio = " = 2.3";
columnLabel = "";
textCoordinates = {{0.09 * months, 0.1}, {0.865 * months, 0.9}};
g2 = Show[display[annotation, displayNumber, xplotlabel,
    xlabel, ylabel, medianTicks, highlight, columnLabel], ImageSize → 250];
```

```
displayNumber = 2;
annotation = "Therapy_Outcome";
firstAnnotation = "No";
secondAnnotation = "CR";
highlight = True;
xplotlabel = "(c)";
xlabel = True;
ylabel = "from the Discovery Set";
medianTicks = Table["", {a, 1, 8}];
hazardRatio = "=3.8";
columnLabel = "";
textCoordinates = {{0.09 * months, 0.1}, {0.865 * months, 0.9}};
g3 = Show[display[annotation, displayNumber, xplotlabel,
    xlabel, ylabel, medianTicks, highlight, columnLabel], ImageSize → 250];
```

```
displayNumber = 2;
annotation = "Neoplasm_Status";
firstAnnotation = "W";
secondAnnotation = "WO";
highlight = True;
xplotlabel = "(d)";
xlabel = True;
ylabel = "False";
medianTicks = Table["", {a, 1, 8}];
hazardRatio = "=13.1";
columnLabel = "";
textCoordinates = {{0.09 * months, 0.1}, {0.865 * months, 0.9}};
textCoordinates[[2]] = {0.865 * months, 0.65};
g4 = Show[display[annotation, displayNumber, xplotlabel,
    xlabel, ylabel, medianTicks, highlight, columnLabel], ImageSize → 250];
```

```
figD = GraphicsGrid[{{g1, g2}, {g3, g4}}, ImageSize → 500, Spacings → {-70, -12}]
```

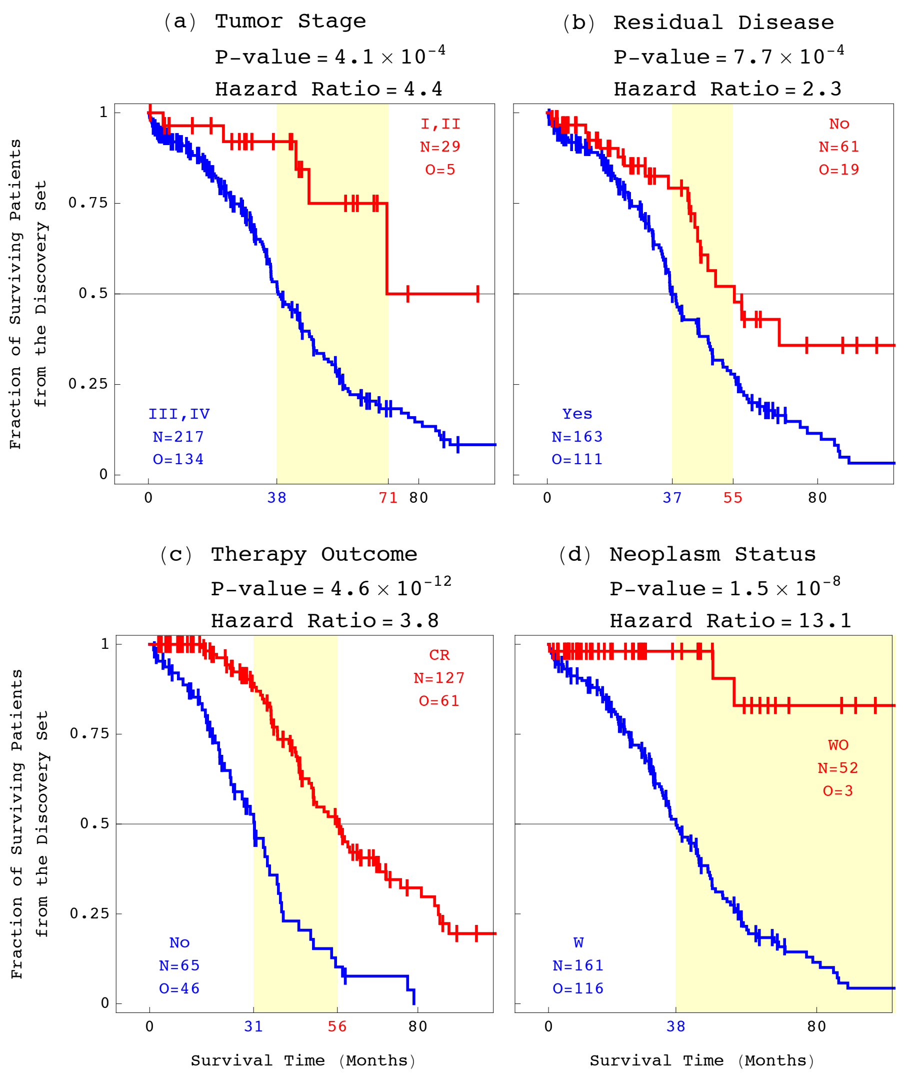


```
Export[path <> "Figure_D.pdf", figD, "PDF", ImageSize → 500, ImageResolution → resolution];
```

```
(* Display the Survival Analyses of the Validation Set Classified by the Standard Indicators *)

stream = path <> "Validation_Patients.txt";
annotations = Import[stream, "Table"];
{patients, annotationsNumber} = Dimensions[annotations] - {1, 1};
annotationNames = Take[annotations, 1, {2, annotationsNumber + 1}][[1]];
annotations = Drop[Import[stream, "Table"], 1, 1];
Clear[stream];

times = Table[
   If[annotationColumn["Days_Death"][[a]] == "Null",
    If[annotationColumn["Days_Followup"][[a]] == "Null",
     "Null",
     annotationColumn["Days_Followup"][[a]] / monthConversion],
    annotationColumn["Days_Death"][[a]] / monthConversion],
   {a, 1, patients}];
status = Table[If[annotationColumn["Days_Death"][[a]] == "Null",
    If[annotationColumn["Days_Followup"][[a]] == "Null", 2, 0], 1],
   {a, 1, patients}];
positionNull = Position[status, 2];
Do[{
  annotations = Drop[annotations, positionNull[[a]]],
  times = Drop[times, positionNull[[a]]],
  status = Drop[status, positionNull[[a]]]},
 {a, Dimensions[positionNull][[1]], 1, -1}]
patients = patients - Dimensions[positionNull][[1]];

displayNumber = 2;
annotation = "Tumor_Stage";
firstAnnotation = "IV";
secondAnnotation = "III";
highlight = True;
xplotlabel = "(a)";
xlabel = False;
ylabel = "from the Validation Set";
medianTicks = Table["", {a, 1, 8}];
hazardRatio = " = 1.8";
columnLabel = "";
textCoordinates = {{0.09 * months, 0.1}, {0.865 * months, 0.9}};
g1 = Show[display[annotation, displayNumber, xplotlabel,
    xlabel, ylabel, medianTicks, highlight, columnLabel], ImageSize → 250];

displayNumber = 2;
annotation = "Residual_Disease";
firstAnnotation = "Yes";
secondAnnotation = "No";
highlight = True;
xplotlabel = "(b)";
xlabel = False;
ylabel = "False";
medianTicks = Table["", {a, 1, 8}];
hazardRatio = " = 2.4";
columnLabel = "";
textCoordinates = {{0.09 * months, 0.1}, {0.865 * months, 0.9}};
g2 = Show[display[annotation, displayNumber, xplotlabel,
    xlabel, ylabel, medianTicks, highlight, columnLabel], ImageSize → 250];
```

```
displayNumber = 2;
annotation = "Therapy_Outcome";
firstAnnotation = "No";
secondAnnotation = "CR";
highlight = True;
xplotlabel = "(c)";
xlabel = True;
ylabel = "from the Validation Set";
medianTicks = Table["", {a, 1, 8}];
hazardRatio = "=3.8";
columnLabel = "";
textCoordinates = {{0.09 * months, 0.1}, {0.865 * months, 0.9}};
g3 = Show[display[annotation, displayNumber, xplotlabel,
    xlabel, ylabel, medianTicks, highlight, columnLabel], ImageSize → 250];
```

```
displayNumber = 2;
annotation = "Neoplasm_Status";
firstAnnotation = "W";
secondAnnotation = "WO";
highlight = True;
xplotlabel = "(d)";
xlabel = True;
ylabel = "False";
medianTicks = Table["", {a, 1, 8}];
hazardRatio = "=16.7";
columnLabel = "";
textCoordinates = {{0.09 * months, 0.1}, {0.865 * months, 0.9}};
textCoordinates[[2]] = {0.865 * months, 0.65};
g4 = Show[display[annotation, displayNumber, xplotlabel,
    xlabel, ylabel, medianTicks, highlight, columnLabel], ImageSize → 250];
```

```
figE = GraphicsGrid[{{g1, g2}, {g3, g4}}, ImageSize → 500, Spacings → {-70, -12}]
```

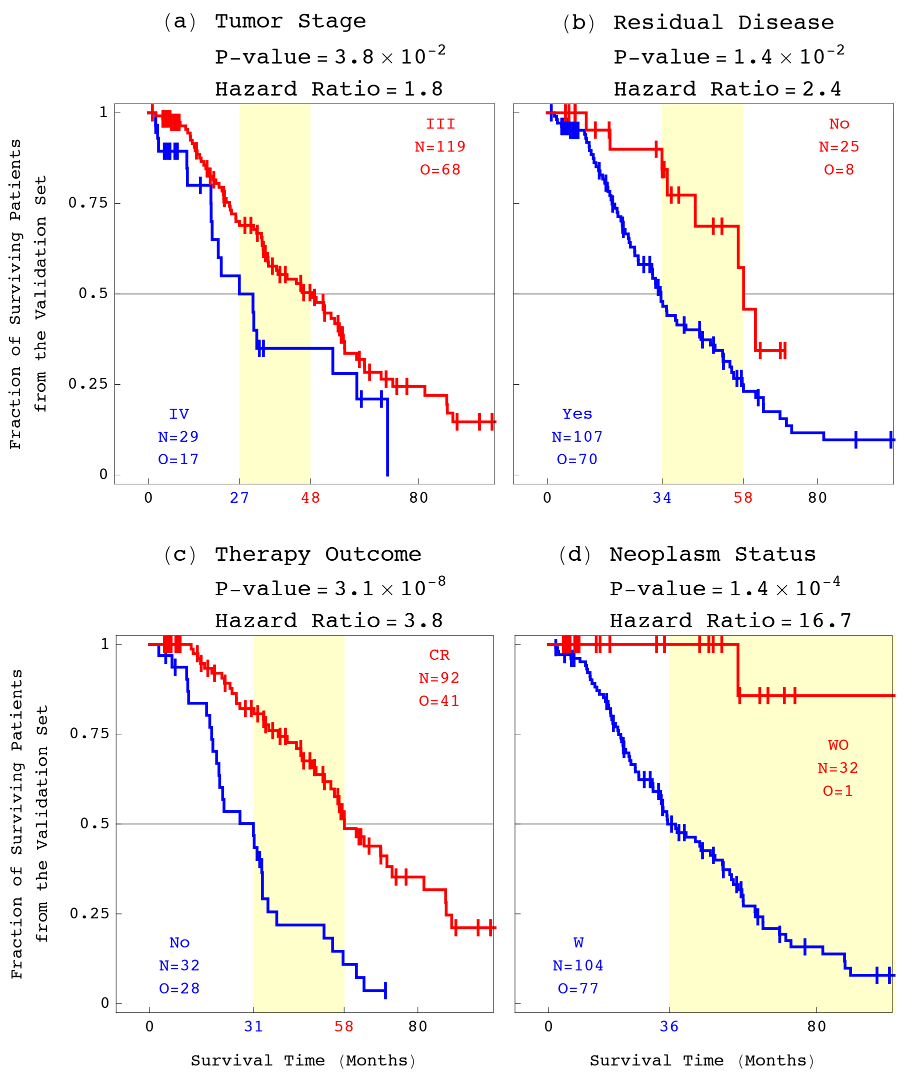


```
Export[path <> "Figure_E.pdf", figE, "PDF", ImageSize → 500, ImageResolution → resolution];
```

```
(* Display the Survival Analyses of the Discovery and Validation Platinum-Based Chemotherapy
Patients Classified by the Tensor GSVD and Tumor Stage *)

title = "Fraction of Surviving Platinum-Based";

stream = path <> "Discovery_Patients.txt";
annotations = Import[stream, "Table"];
{patients, annotationsNumber} = Dimensions[annotations] - {1, 1};
annotationNames = Take[annotations, 1, {2, annotationsNumber + 1}][[1]];
annotations = Drop[Import[stream, "Table"], 1, 1];
Clear[stream];

times = Table[
   If[annotationColumn["Days_Death"][[a]] == "Null",
    If[annotationColumn["Days_Followup"][[a]] == "Null",
     "Null",
     annotationColumn["Days_Followup"][[a]] / monthConversion],
    annotationColumn["Days_Death"][[a]] / monthConversion],
   {a, 1, patients}];
status = Table[
   If[annotationColumn["Platinum-Based_Chemotherapy"][[a]] == "Null", 2,
    If[annotationColumn["Platinum-Based_Chemotherapy"][[a]] == "No", 2,
     If[annotationColumn["Days_Death"][[a]] == "Null",
      If[annotationColumn["Days_Followup"][[a]] == "Null", 2, 0], 1]]],
   {a, 1, patients}];
positionNull = Position[status, 2];
Do[{
  annotations = Drop[annotations, positionNull[[a]]],
  times = Drop[times, positionNull[[a]]],
  status = Drop[status, positionNull[[a]]]},
 {a, Dimensions[positionNull][[1]], 1, -1}]
patients = patients - Dimensions[positionNull][[1]];

displayNumber = 2;
annotation = "Probelet_6p+12p_(Coeff.)";
firstAnnotation = "High";
secondAnnotation = "Low";
highlight = True;
xplotlabel = "(a)";
xlabel = False;
ylabel = "Chemo. Patients from the Discovery Set";
medianTicks = Table["", {a, 1, 8}];
hazardRatio = " = 2.0";
columnLabel = "6p+12p";
textCoordinates = {{0.09 * months, 0.1}, {0.865 * months, 0.9}};
g1 = Show[display[annotation, displayNumber, xplotlabel,
    xlabel, ylabel, medianTicks, highlight, columnLabel], ImageSize → 250];
```

```
displayNumber = 2;
annotation = "Probelet_7p_(Coeff.)";
firstAnnotation = "Low";
secondAnnotation = "High";
highlight = True;
xplotlabel = "(b)";
xlabel = False;
ylabel = "False";
medianTicks = Table["", {a, 1, 8}];
medianTicks[[2]] = " ";
medianTicks[[3]] = " ";
hazardRatio = " = 1.5";
columnLabel = "7p";
textCoordinates = {{0.09 * months, 0.1}, {0.865 * months, 0.9}};
g2 = Show[display[annotation, displayNumber, xplotlabel,
    xlabel, ylabel, medianTicks, highlight, columnLabel], ImageSize → 250];

displayNumber = 2;
annotation = "Probelet_Xq_(Coeff.)";
firstAnnotation = "Low";
secondAnnotation = "High";
highlight = True;
xplotlabel = "(c)";
xlabel = False;
ylabel = "False";
medianTicks = Table["", {a, 1, 8}];
hazardRatio = " = 1.6";
columnLabel = "Xq";
textCoordinates = {{0.09 * months, 0.1}, {0.865 * months, 0.9}};
g3 = Show[display[annotation, displayNumber, xplotlabel,
    xlabel, ylabel, medianTicks, highlight, columnLabel], ImageSize → 250];

displayNumber = 4;
annotation = "Probelet_6p+12p/Tumor_Stage";
firstAnnotation = "High/III,IV";
secondAnnotation = "Low/I,II";
thirdAnnotation = "Low/III,IV";
fourthAnnotation = "High/I,II";
highlight = True;
xplotlabel = "(d)";
xlabel = False;
ylabel = "Chemo. Patients from the Discovery Set";
medianTicks = Table["", {a, 1, 8}];
medianTicks[[2]] = "  ";
medianTicks[[8]] = " ";
medianTicks[[5]] = "  ";
hazardRatio = "s = 1.8/4.1";
pValue = 4.8 * 10^-5;
columnLabel = "";
textCoordinates =
  {{0.09 * months, 0.1}, {0.865 * months, 0.9}, {0.09 * months, 0.35}, {0.09 * months, 0.6}};
g4 = Show[display[annotation, displayNumber, xplotlabel, xlabel,
    ylabel, medianTicks, highlight, columnLabel], ImageSize → 250];
```

```
displayNumber = 4;
annotation = "Probelet_7p/Tumor_Stage";
firstAnnotation = "Low/III,IV";
secondAnnotation = "High/I,II";
thirdAnnotation = "High/III,IV";
fourthAnnotation = "Low/I,II";
highlight = True;
xplotlabel = "(e)";
xlabel = False;
ylabel = "False";
medianTicks = Table["", {a, 1, 8}];
medianTicks[[7]] = " ";
medianTicks[[4]] = " ";
hazardRatio = "blank";
pValue = 1.6 * 10^-3;
columnLabel = "";
textCoordinates =
  {{0.09 * months, 0.1}, {0.865 * months, 0.9}, {0.09 * months, 0.35}, {0.09 * months, 0.6}};
g5 = Show[display[annotation, displayNumber, xplotlabel, xlabel,
    ylabel, medianTicks, highlight, columnLabel], ImageSize → 250];

displayNumber = 4;
annotation = "Probelet_Xq/Tumor_Stage";
firstAnnotation = "Low/III,IV";
secondAnnotation = "High/I,II";
thirdAnnotation = "High/III,IV";
fourthAnnotation = "Low/I,II";
highlight = True;
xplotlabel = "(f)";
xlabel = False;
ylabel = "False";
medianTicks = Table["", {a, 1, 8}];
hazardRatio = "blank";
pValue = 9.0 * 10^-4;
columnLabel = "";
textCoordinates =
  {{0.09 * months, 0.1}, {0.865 * months, 0.9}, {0.09 * months, 0.35}, {0.09 * months, 0.6}};
g6 = Show[display[annotation, displayNumber, xplotlabel, xlabel,
    ylabel, medianTicks, highlight, columnLabel], ImageSize → 250];

stream = path <> "Validation_Patients.txt";
annotations = Import[stream, "Table"];
{patients, annotationsNumber} = Dimensions[annotations] - {1, 1};
annotationNames = Take[annotations, 1, {2, annotationsNumber + 1}][[1]];
annotations = Drop[Import[stream, "Table"], 1, 1];
Clear[stream];
```

```
times = Table[
   If[annotationColumn["Days_Death"][[a]] == "Null",
    If[annotationColumn["Days_Followup"][[a]] == "Null",
     "Null",
     annotationColumn["Days_Followup"][[a]] / monthConversion],
    annotationColumn["Days_Death"][[a]] / monthConversion],
   {a, 1, patients}];
status = Table[
   If[annotationColumn["Platinum-Based_Chemotherapy"][[a]] == "Null", 2,
    If[annotationColumn["Platinum-Based_Chemotherapy"][[a]] == "No", 2,
     If[annotationColumn["Days_Death"][[a]] == "Null",
      If[annotationColumn["Days_Followup"][[a]] == "Null", 2, 0], 1]]],
   {a, 1, patients}];
positionNull = Position[status, 2];
Do[{
  annotations = Drop[annotations, positionNull[[a]]],
  times = Drop[times, positionNull[[a]]],
  status = Drop[status, positionNull[[a]]]},
 {a, Dimensions[positionNull][[1]], 1, -1}]
patients = patients - Dimensions[positionNull][[1]];

displayNumber = 2;
annotation = "Arraylet_6p+12p_(Corr.)";
firstAnnotation = "High";
secondAnnotation = "Low";
highlight = True;
xplotlabel = "(g)";
xlabel = True;
ylabel = "Chemo. Patients from the Validation Set";
medianTicks = Table["", {a, 1, 8}];
hazardRatio = " = 1.8";
columnLabel = "";
textCoordinates = {{0.09 * months, 0.1}, {0.865 * months, 0.9}};
g7 = Show[display[annotation, displayNumber, xplotlabel,
    xlabel, ylabel, medianTicks, highlight, columnLabel], ImageSize → 250];

displayNumber = 2;
annotation = "Arraylet_7p_(Corr.)";
firstAnnotation = "Low";
secondAnnotation = "High";
highlight = True;
xplotlabel = "(h)";
xlabel = True;
ylabel = "False";
medianTicks = Table["", {a, 1, 8}];
hazardRatio = " = 1.7";
columnLabel = "";
textCoordinates = {{0.09 * months, 0.1}, {0.865 * months, 0.9}};
g8 = Show[display[annotation, displayNumber, xplotlabel,
    xlabel, ylabel, medianTicks, highlight, columnLabel], ImageSize → 250];
```

```
displayNumber = 2;
annotation = "Arraylet_Xq_(Corr.)";
firstAnnotation = "Low";
secondAnnotation = "High";
highlight = True;
xplotlabel = "(i)";
xlabel = True;
ylabel = "False";
medianTicks = Table["", {a, 1, 8}];
hazardRatio = " = 2.1";
columnLabel = "";
textCoordinates = {{0.09 * months, 0.1}, {0.865 * months, 0.9}};
g9 = Show[display[annotation, displayNumber, xplotlabel,
     xlabel, ylabel, medianTicks, highlight, columnLabel], ImageSize → 250];

figF =
 GraphicsGrid[{{g1, g2, g3}, {g4, g5, g6}, {g7, g8, g9}}, ImageSize → 750, Spacings → {-67, -12}]
```

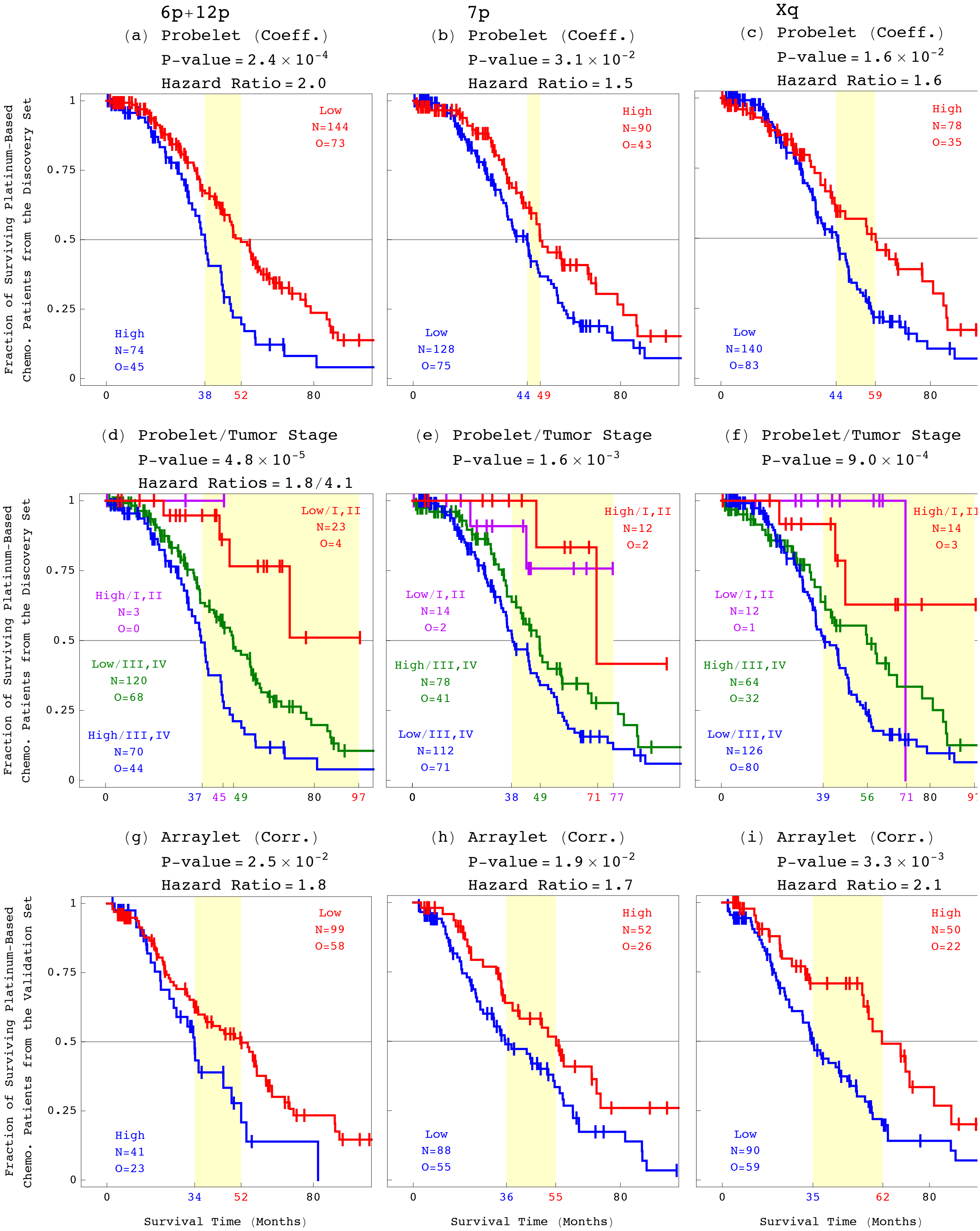


```
Export[path <> "Figure_F.pdf", figF, "PDF", ImageSize → 750, ImageResolution → resolution];
```

```
(* Display the Survival Analyses of the Validation Set Classified by the Tensor GSVD and Stage *)

stream = path <> "Validation_Patients.txt";
annotations = Import[stream, "Table"];
{patients, annotationsNumber} = Dimensions[annotations] - {1, 1};
annotationNames = Take[annotations, 1, {2, annotationsNumber + 1}][[1]];
annotations = Drop[Import[stream, "Table"], 1, 1];
Clear[stream];

times = Table[
   If[annotationColumn["Days_Death"][[a]] == "Null",
    If[annotationColumn["Days_Followup"][[a]] == "Null",
     "Null",
     annotationColumn["Days_Followup"][[a]] / monthConversion],
    annotationColumn["Days_Death"][[a]] / monthConversion],
   {a, 1, patients}];
status = Table[If[annotationColumn["Days_Death"][[a]] == "Null",
    If[annotationColumn["Days_Followup"][[a]] == "Null", 2, 0], 1],
   {a, 1, patients}];
positionNull = Position[status, 2];
Do[{
  annotations = Drop[annotations, positionNull[[a]]],
  times = Drop[times, positionNull[[a]]],
  status = Drop[status, positionNull[[a]]]},
 {a, Dimensions[positionNull][[1]], 1, -1}]
patients = patients - Dimensions[positionNull][[1]];

displayNumber = 4;
annotation = "Arraylet_6p+12p/Tumor_Stage";
firstAnnotation = "High/IV";
secondAnnotation = "Low/III";
thirdAnnotation = "Low/IV";
fourthAnnotation = "High/III";
highlight = True;
xplotlabel = "(a)";
xlabel = True;
ylabel = "from the Validation Set";
medianTicks = Table["", {a, 1, 8}];
hazardRatio = "s = 1.9/1.8";
medianTicks[[7]] = "   ";
medianTicks[[6]] = "  ";
pValue = 5.4 * 10^-3;
columnLabel = "6p+12p";
textCoordinates =
  {{0.09 * months, 0.1}, {0.865 * months, 0.9}, {0.09 * months, 0.35}, {0.09 * months, 0.6}};
textCoordinates[[1]] = {0.09 * months - 3, 0.1};
textCoordinates[[3]] = {0.09 * months - 3, 0.35};
textCoordinates[[4]] = {0.865 * months, 0.65};
g1 = Show[display[annotation, displayNumber, xplotlabel,
    xlabel, ylabel, medianTicks, highlight, columnLabel], ImageSize → 250];
```

```
displayNumber = 4;
annotation = "Arraylet_7p/Tumor_Stage";
firstAnnotation = "Low/IV";
secondAnnotation = "High/III";
thirdAnnotation = "High/IV";
fourthAnnotation = "Low/III";
highlight = True;
xplotlabel = "(b)";
xlabel = True;
ylabel = "False";
medianTicks = Table["", {a, 1, 8}];
medianTicks[[7]] = " ";
hazardRatio = "s = 1.8/1.8";
pValue = 1.6 * 10^-2;
columnLabel = "7p";
textCoordinates =
  {{0.09 * months, 0.1}, {0.865 * months, 0.9}, {0.09 * months, 0.35}, {0.09 * months, 0.6}};
textCoordinates[[1]] = {0.09 * months - 3, 0.1};
textCoordinates[[3]] = {0.09 * months - 3, 0.35};
textCoordinates[[4]] = {0.865 * months, 0.65};
g2 = Show[display[annotation, displayNumber, xplotlabel,
    xlabel, ylabel, medianTicks, highlight, columnLabel], ImageSize → 250];

displayNumber = 4;
annotation = "Arraylet_Xq/Tumor_Stage";
firstAnnotation = "Low/IV";
secondAnnotation = "High/III";
thirdAnnotation = "High/IV";
fourthAnnotation = "Low/III";
highlight = True;
xplotlabel = "(c)";
xlabel = True;
ylabel = "False";
medianTicks = Table["", {a, 1, 8}];
medianTicks[[4]] = " ";
medianTicks[[5]] = "  ";
hazardRatio = "s = 2.1/2.1";
pValue = 1.1 * 10^-4;
columnLabel = "Xq";
textCoordinates =
  {{0.09 * months, 0.1}, {0.865 * months, 0.9}, {0.09 * months, 0.35}, {0.09 * months, 0.6}};
textCoordinates = {{0.09 * months - 3, 0.1}, {0.865 * months, 0.65},
   {0.865 * months, 0.9}, {0.09 * months - 3, 0.35}};
g3 = Show[display[annotation, displayNumber, xplotlabel, xlabel,
    ylabel, medianTicks, highlight, columnLabel], ImageSize → 250];
```

```
figG = GraphicsGrid[{{g1, g2, g3}}, ImageSize → 750, Spacings → {-67, -12}]
```

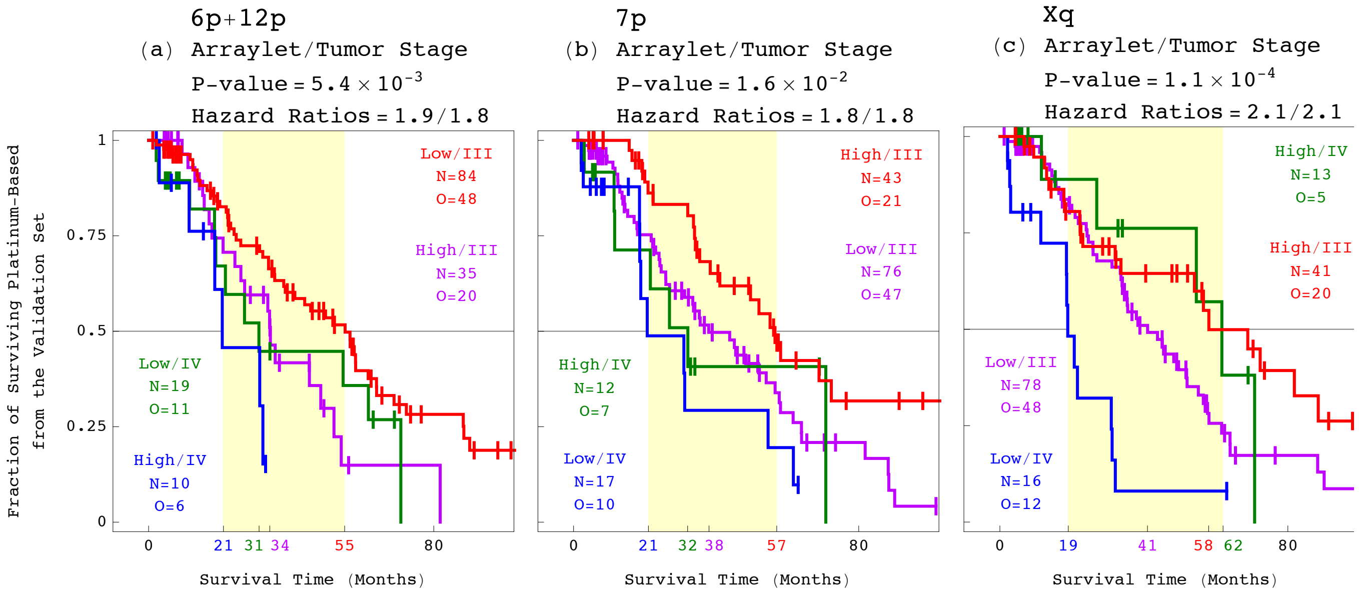


```
Export[path <> "Figure_G.pdf", figG, "PDF", ImageSize → 750, ImageResolution → resolution];
```

```
(* Display the Survival Analyses of the Discovery Set Classified by the Tensor GSVD and Standard
Indicators other than Tumor Stage *)

stream = path <> "Discovery_Patients.txt";
annotations = Import[stream, "Table"];
{patients, annotationsNumber} = Dimensions[annotations] - {1, 1};
annotationNames = Take[annotations, 1, {2, annotationsNumber + 1}][[1]];
annotations = Drop[Import[stream, "Table"], 1, 1];
Clear[stream];

times = Table[
   If[annotationColumn["Days_Death"][[a]] == "Null",
    If[annotationColumn["Days_Followup"][[a]] == "Null",
     "Null",
     annotationColumn["Days_Followup"][[a]] / monthConversion],
    annotationColumn["Days_Death"][[a]] / monthConversion],
   {a, 1, patients}];
status = Table[If[annotationColumn["Days_Death"][[a]] == "Null",
    If[annotationColumn["Days_Followup"][[a]] == "Null", 2, 0], 1],
   {a, 1, patients}];
positionNull = Position[status, 2];
Do[{
  annotations = Drop[annotations, positionNull[[a]]],
  times = Drop[times, positionNull[[a]]],
  status = Drop[status, positionNull[[a]]]},
 {a, Dimensions[positionNull][[1]], 1, -1}]
patients = patients - Dimensions[positionNull][[1]];

displayNumber = 4;
annotation = "Probelet_6p+12p/Residual_Disease";
firstAnnotation = "High/Yes";
secondAnnotation = "Low/No";
thirdAnnotation = "Low/Yes";
fourthAnnotation = "High/No";
highlight = True;
xplotlabel = "(a)";
xlabel = False;
ylabel = "from the Discovery Set";
medianTicks = Table["", {a, 1, 8}];
medianTicks[[2]] = "   ";
medianTicks[[7]] = "   ";
medianTicks[[3]] = "  ";
hazardRatio = "s = 1.6/2.2";
pValue = 6.6 * 10^-4;
columnLabel = "6p+12p";
textCoordinates =
  {{0.09 * months, 0.1}, {0.865 * months, 0.9}, {0.09 * months, 0.35}, {0.09 * months, 0.6}};
g1 = Show[display[annotation, displayNumber, xplotlabel, xlabel,
    ylabel, medianTicks, highlight, columnLabel], ImageSize → 250];
```

```
displayNumber = 4;
annotation = "Probelet_7p/Residual_Disease";
firstAnnotation = "Low/Yes";
secondAnnotation = "High/No";
thirdAnnotation = "High/Yes";
fourthAnnotation = "Low/No";
highlight = True;
xplotlabel = "(b)";
xlabel = False;
ylabel = "False";
medianTicks = Table["", {a, 1, 8}];
hazardRatio = "s = 1.5/2.2";
pValue = 1.1 * 10^-3;
columnLabel = "7p";
textCoordinates =
  {{0.09 * months, 0.1}, {0.865 * months, 0.9}, {0.09 * months, 0.35}, {0.09 * months, 0.6}};
g2 = Show[display[annotation, displayNumber, xplotlabel, xlabel,
     ylabel, medianTicks, highlight, columnLabel], ImageSize → 250];

displayNumber = 4;
annotation = "Probelet_Xq/Residual_Disease";
firstAnnotation = "Low/Yes";
secondAnnotation = "High/No";
thirdAnnotation = "High/Yes";
fourthAnnotation = "Low/No";
highlight = True;
xplotlabel = "(c)";
xlabel = False;
ylabel = "False";
medianTicks = Table["", {a, 1, 8}];
medianTicks[[2]] = "   ";
medianTicks[[7]] = "   ";
hazardRatio = "s = 1.7/2.2";
pValue = 4.0 * 10^-4;
columnLabel = "Xq";
textCoordinates =
  {{0.09 * months, 0.1}, {0.865 * months, 0.9}, {0.09 * months, 0.35}, {0.09 * months, 0.6}};
textCoordinates[[4]] = {0.865 * months, 0.35};
g3 = Show[display[annotation, displayNumber, xplotlabel,
     xlabel, ylabel, medianTicks, highlight, columnLabel], ImageSize → 250];
```

```
displayNumber = 4;
annotation = "Probelet_6p+12p/Therapy_Outcome";
firstAnnotation = "High/No";
secondAnnotation = "Low/CR";
thirdAnnotation = "Low/No";
fourthAnnotation = "High/CR";
highlight = True;
xplotlabel = "(d)";
xlabel = False;
ylabel = "from the Discovery Set";
medianTicks = Table["", {a, 1, 8}];
medianTicks[[1]] = " ";
medianTicks[[6]] = " ";
hazardRatio = "s = 1.6/3.6";
pValue = 8.1 * 10^-12;
columnLabel = "";
textCoordinates =
  {{0.09 * months, 0.1}, {0.865 * months, 0.9}, {0.09 * months, 0.35}, {0.09 * months, 0.6}};
textCoordinates[[1]] = {0.09 * months, 0.35};
textCoordinates[[3]] = {0.09 * months, 0.1};
textCoordinates[[4]] = {0.865 * months, 0.65};
g4 = Show[display[annotation, displayNumber, xplotlabel,
    xlabel, ylabel, medianTicks, highlight, columnLabel], ImageSize → 250];

displayNumber = 4;
annotation = "Probelet_7p/Therapy_Outcome";
firstAnnotation = "Low/No";
secondAnnotation = "High/CR";
thirdAnnotation = "High/No";
fourthAnnotation = "Low/CR";
highlight = True;
xplotlabel = "(e)";
xlabel = False;
ylabel = "False";
medianTicks = Table["", {a, 1, 8}];
medianTicks[[2]] = " ";
medianTicks[[5]] = " ";
hazardRatio = "s = 1.6/3.5";
pValue = 3.9 * 10^-12;
columnLabel = "";
textCoordinates =
  {{0.09 * months, 0.1}, {0.865 * months, 0.9}, {0.09 * months, 0.35}, {0.09 * months, 0.6}};
textCoordinates[[4]] = {0.865 * months, 0.65};
g5 = Show[display[annotation, displayNumber, xplotlabel,
    xlabel, ylabel, medianTicks, highlight, columnLabel], ImageSize → 250];
```

```
displayNumber = 4;
annotation = "Probelet_Xq/Therapy_Outcome";
firstAnnotation = "Low/No";
secondAnnotation = "High/CR";
thirdAnnotation = "High/No";
fourthAnnotation = "Low/CR";
highlight = True;
xplotlabel = "(f)";
xlabel = False;
ylabel = "False";
medianTicks = Table["", {a, 1, 8}];
medianTicks[[2]] = "  ";
medianTicks[[3]] = "  ";
hazardRatio = "s = 1.6/3.8";
pValue = 1.8 * 10^-11;
columnLabel = "";
textCoordinates =
  {{0.09 * months, 0.1}, {0.865 * months, 0.9}, {0.09 * months, 0.35}, {0.09 * months, 0.6}};
g6 = Show[display[annotation, displayNumber, xplotlabel, xlabel,
    ylabel, medianTicks, highlight, columnLabel], ImageSize → 250];

displayNumber = 4;
annotation = "Probelet_6p+12p/Neoplasm_Status";
firstAnnotation = "High/W";
secondAnnotation = "Low/WO";
thirdAnnotation = "Low/W";
fourthAnnotation = "High/WO";
highlight = True;
xplotlabel = "(g)";
xlabel = True;
ylabel = "from the Discovery Set";
medianTicks = Table["", {a, 1, 8}];
hazardRatio = "s = 1.5/12.1";
pValue = 2.2 * 10^-8;
columnLabel = "";
textCoordinates =
  {{0.09 * months, 0.1}, {0.865 * months, 0.9}, {0.09 * months, 0.35}, {0.09 * months, 0.6}};
textCoordinates[[2]] = {0.865 * months, 0.6};
textCoordinates[[4]] = {0.865 * months, 0.35};
g7 = Show[display[annotation, displayNumber, xplotlabel,
    xlabel, ylabel, medianTicks, highlight, columnLabel], ImageSize → 250];
```

```
displayNumber = 4;
annotation = "Probelet_7p/Neoplasm_Status";
firstAnnotation = "Low/W";
secondAnnotation = "High/WO";
thirdAnnotation = "High/W";
fourthAnnotation = "Low/WO";
highlight = True;
xplotlabel = "(h)";
xlabel = True;
ylabel = "False";
medianTicks = Table["", {a, 1, 8}];
hazardRatio = "s = 1.7/12.3";
pValue = 6.1 * 10^-9;
columnLabel = "";
textCoordinates =
  {{0.09 * months, 0.1}, {0.865 * months, 0.9}, {0.09 * months, 0.35}, {0.09 * months, 0.6}};
textCoordinates[[2]] = {0.865 * months, 0.6};
textCoordinates[[4]] = {0.865 * months, 0.35};
g8 = Show[display[annotation, displayNumber, xplotlabel,
    xlabel, ylabel, medianTicks, highlight, columnLabel], ImageSize → 250];

displayNumber = 4;
annotation = "Probelet_Xq/Neoplasm_Status";
firstAnnotation = "Low/W";
secondAnnotation = "High/WO";
thirdAnnotation = "High/W";
fourthAnnotation = "Low/WO";
highlight = True;
xplotlabel = "(i)";
xlabel = True;
ylabel = "False";
medianTicks = Table["", {a, 1, 8}];
hazardRatio = "s = 1.7/13.9";
pValue = 2.1 * 10^-8;
columnLabel = "";
textCoordinates =
  {{0.09 * months, 0.1}, {0.865 * months, 0.9}, {0.09 * months, 0.35}, {0.09 * months, 0.6}};
textCoordinates[[2]] = {0.865 * months, 0.6};
textCoordinates[[4]] = {0.865 * months, 0.35};
g9 = Show[display[annotation, displayNumber, xplotlabel,
    xlabel, ylabel, medianTicks, highlight, columnLabel], ImageSize → 250];

figH =
 GraphicsGrid[{{g1, g2, g3}, {g4, g5, g6}, {g7, g8, g9}}, ImageSize → 750, Spacings → {-67, -12}]
```

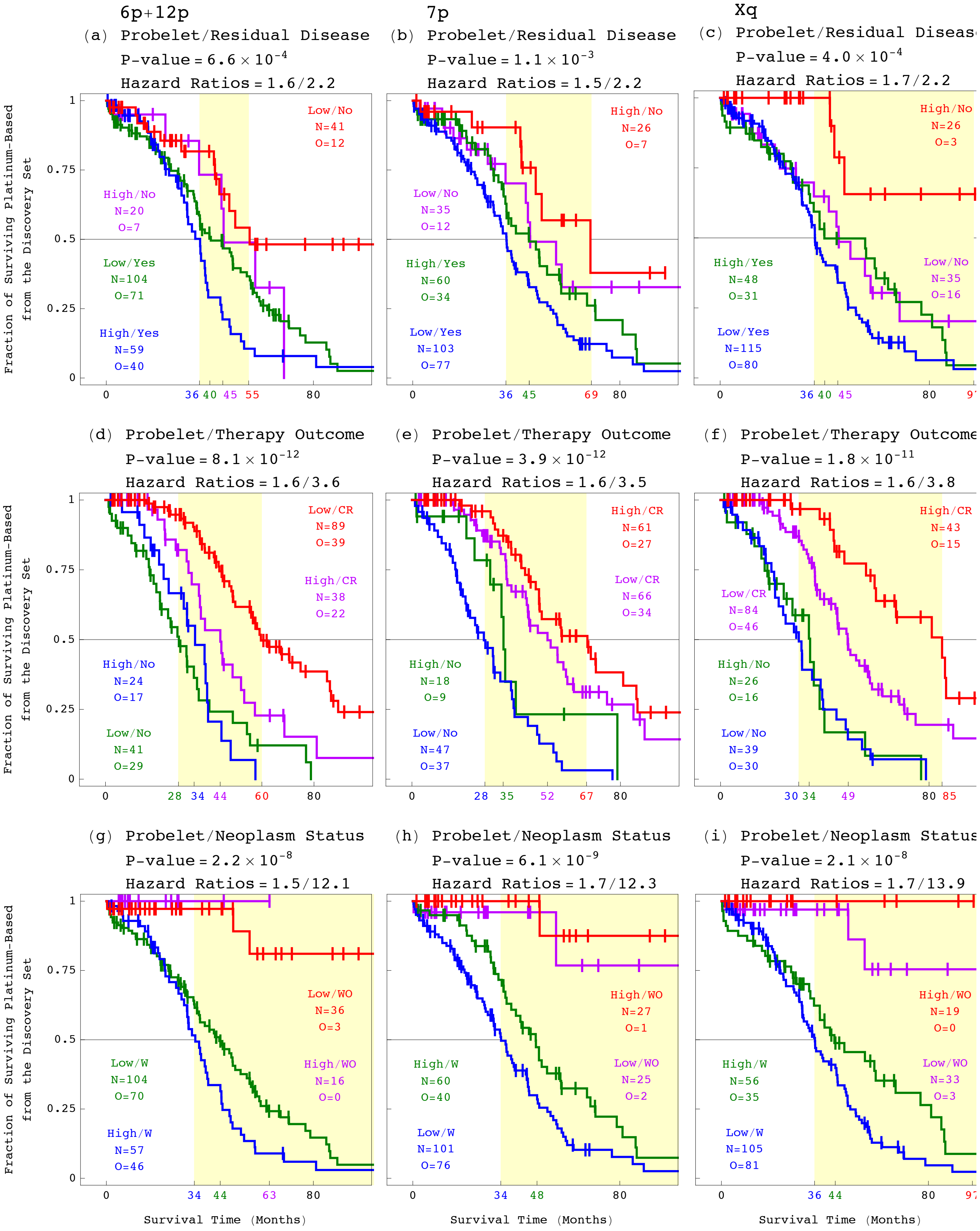


```
Export[path <> "Figure_H.pdf", figH, "PDF", ImageSize → 750, ImageResolution → resolution];
```

```
(* Display the Survival Analyses of the Validation Set Classified by the Tensor GSVD and Standard
Indicators other than Tumor Stage *)

stream = path <> "Validation_Patients.txt";
annotations = Import[stream, "Table"];
{patients, annotationsNumber} = Dimensions[annotations] - {1, 1};
annotationNames = Take[annotations, 1, {2, annotationsNumber + 1}][[1]];
annotations = Drop[Import[stream, "Table"], 1, 1];
Clear[stream];

times = Table[
   If[annotationColumn["Days_Death"][[a]] == "Null",
    If[annotationColumn["Days_Followup"][[a]] == "Null",
     "Null",
     annotationColumn["Days_Followup"][[a]] / monthConversion],
    annotationColumn["Days_Death"][[a]] / monthConversion],
   {a, 1, patients}];
status = Table[If[annotationColumn["Days_Death"][[a]] == "Null",
    If[annotationColumn["Days_Followup"][[a]] == "Null", 2, 0], 1],
   {a, 1, patients}];
positionNull = Position[status, 2];
Do[{
  annotations = Drop[annotations, positionNull[[a]]],
  times = Drop[times, positionNull[[a]]],
  status = Drop[status, positionNull[[a]]]},
 {a, Dimensions[positionNull][[1]], 1, -1}]
patients = patients - Dimensions[positionNull][[1]];

displayNumber = 4;
annotation = "Arraylet_6p+12p/Residual_Disease";
firstAnnotation = "High/Yes";
secondAnnotation = "Low/No";
thirdAnnotation = "Low/Yes";
fourthAnnotation = "High/No";
highlight = True;
xplotlabel = "(a)";
xlabel = False;
ylabel = "from the Validation Set";
medianTicks = Table["", {a, 1, 8}];
medianTicks[[2]] = "  ";
medianTicks[[7]] = "     ";
hazardRatio = "blank";
pValue = 2.6 * 10^-2;
columnLabel = "6p+12p";
textCoordinates =
  {{0.09 * months, 0.1}, {0.865 * months, 0.9}, {0.09 * months, 0.35}, {0.09 * months, 0.6}};
textCoordinates[[4]] = {0.865 * months, 0.65};
g1 = Show[display[annotation, displayNumber, xplotlabel,
    xlabel, ylabel, medianTicks, highlight, columnLabel], ImageSize → 250];
```

```
displayNumber = 4;
annotation = "Arraylet_7p/Residual_Disease";
firstAnnotation = "Low/Yes";
secondAnnotation = "High/No";
thirdAnnotation = "High/Yes";
fourthAnnotation = "Low/No";
highlight = True;
xplotlabel = "(b)";
xlabel = False;
ylabel = "False";
medianTicks = Table["", {a, 1, 8}];
medianTicks[[7]] = "  ";
hazardRatio = "s = 1.9/2.5";
pValue = 1.2 * 10^-3;
columnLabel = "7p";
textCoordinates =
  {{0.09 * months, 0.1}, {0.865 * months, 0.9}, {0.09 * months, 0.35}, {0.09 * months, 0.6}};
textCoordinates[[2]] = {0.865 * months, 0.65};
textCoordinates[[4]] = {0.865 * months, 0.9};
g2 = Show[display[annotation, displayNumber, xplotlabel,
    xlabel, ylabel, medianTicks, highlight, columnLabel], ImageSize → 250];

displayNumber = 4;
annotation = "Arraylet_Xq/Residual_Disease";
firstAnnotation = "Low/Yes";
secondAnnotation = "High/No";
thirdAnnotation = "High/Yes";
fourthAnnotation = "Low/No";
highlight = True;
xplotlabel = "(c)";
xlabel = False;
ylabel = "False";
medianTicks = Table["", {a, 1, 8}];
medianTicks[[5]] = "  ";
medianTicks[[8]] = "  "
hazardRatio = "s = 2.2/2.7";
pValue = 1.3 * 10^-3;
columnLabel = "Xq";
textCoordinates =
  {{0.09 * months, 0.1}, {0.865 * months, 0.9}, {0.09 * months, 0.35}, {0.09 * months, 0.6}};
textCoordinates[[4]] = {0.865 * months, 0.65};
g3 = Show[display[annotation, displayNumber, xplotlabel,
    xlabel, ylabel, medianTicks, highlight, columnLabel], ImageSize → 250];
```

```
displayNumber = 4;
annotation = "Arraylet_6p+12p/Therapy_Outcome";
firstAnnotation = "High/No";
secondAnnotation = "Low/CR";
thirdAnnotation = "Low/No";
fourthAnnotation = "High/CR";
highlight = True;
xplotlabel = "(d)";
xlabel = False;
ylabel = "from the Validation Set";
medianTicks = Table["", {a, 1, 8}];
hazardRatio = "s = 2.0/3.8";
pValue = 3.8 * 10^-8;
columnLabel = "";
textCoordinates =
  {{0.09 * months, 0.1}, {0.865 * months, 0.9}, {0.09 * months, 0.35}, {0.09 * months, 0.6}};
textCoordinates[[4]] = {0.865 * months, 0.65};
g4 = Show[display[annotation, displayNumber, xplotlabel,
     xlabel, ylabel, medianTicks, highlight, columnLabel], ImageSize → 250];

displayNumber = 4;
annotation = "Arraylet_7p/Therapy_Outcome";
firstAnnotation = "Low/No";
secondAnnotation = "High/CR";
thirdAnnotation = "High/No";
fourthAnnotation = "Low/CR";
highlight = True;
xplotlabel = "(e)";
xlabel = False;
ylabel = "False";
medianTicks = Table["", {a, 1, 8}];
medianTicks[[2]] = "   ";
hazardRatio = "blank";
pValue = 3.5 * 10^-7;
columnLabel = "";
textCoordinates =
  {{0.09 * months, 0.1}, {0.865 * months, 0.9}, {0.09 * months, 0.35}, {0.09 * months, 0.6}};
textCoordinates[[4]] = {0.865 * months, 0.65};
g5 = Show[display[annotation, displayNumber, xplotlabel,
     xlabel, ylabel, medianTicks, highlight, columnLabel], ImageSize → 250];

displayNumber = 4;
annotation = "Arraylet_Xq/Therapy_Outcome";
firstAnnotation = "Low/No";
secondAnnotation = "High/CR";
thirdAnnotation = "High/No";
fourthAnnotation = "Low/CR";
highlight = True;
xplotlabel = "(f)";
xlabel = False;
ylabel = "False";
medianTicks = Table["", {a, 1, 8}];
hazardRatio = "s = 2.1/3.9";
pValue = 6.6 * 10^-9;
columnLabel = "";
textCoordinates =
  {{0.09 * months, 0.1}, {0.865 * months, 0.9}, {0.09 * months, 0.35}, {0.09 * months, 0.6}};
textCoordinates[[4]] = {0.865 * months, 0.65};
g6 = Show[display[annotation, displayNumber, xplotlabel,
     xlabel, ylabel, medianTicks, highlight, columnLabel], ImageSize → 250];
```

```
displayNumber = 4;
annotation = "Arraylet_6p+12p/Neoplasm_Status";
firstAnnotation = "High/W";
secondAnnotation = "Low/WO";
thirdAnnotation = "Low/W";
fourthAnnotation = "High/WO";
highlight = True;
xplotlabel = "(g)";
xlabel = True;
ylabel = "from the Validation Set";
medianTicks = Table["", {a, 1, 8}];
hazardRatio = "s = 1.8/15.6";
pValue = N[8 * 10^-5];
columnLabel = "";
textCoordinates =
  {{0.09 * months, 0.1}, {0.865 * months, 0.9}, {0.09 * months, 0.35}, {0.09 * months, 0.6}};
textCoordinates[[2]] = {0.865 * months, 0.6 - 0.03};
textCoordinates[[4]] = {0.865 * months, 0.35 - 0.03};
g7 = Show[display[annotation, displayNumber, xplotlabel,
    xlabel, ylabel, medianTicks, highlight, columnLabel], ImageSize → 250];

displayNumber = 4;
annotation = "Arraylet_7p/Neoplasm_Status";
firstAnnotation = "Low/W";
secondAnnotation = "High/WO";
thirdAnnotation = "High/W";
fourthAnnotation = "Low/WO";
highlight = True;
xplotlabel = "(h)";
xlabel = True;
ylabel = "False";
medianTicks = Table["", {a, 1, 8}];
medianTicks[[8]] = " ";
hazardRatio = "s = 1.8/16.6";
pValue = 7.0 * 10^-5;
columnLabel = "";
textCoordinates =
  {{0.09 * months, 0.1}, {0.865 * months, 0.9}, {0.09 * months, 0.35}, {0.09 * months, 0.6}};
textCoordinates[[2]] = {0.865 * months, 0.6 - 0.03};
textCoordinates[[4]] = {0.865 * months, 0.35 - 0.03};
g8 = Show[display[annotation, displayNumber, xplotlabel,
    xlabel, ylabel, medianTicks, highlight, columnLabel], ImageSize → 250];
```

```
displayNumber = 4;
annotation = "Arraylet_Xq/Neoplasm_Status";
firstAnnotation = "Low/W";
secondAnnotation = "High/WO";
thirdAnnotation = "High/W";
fourthAnnotation = "Low/WO";
highlight = True;
xplotlabel = "(i)";
xlabel = True;
ylabel = "False";
medianTicks = Table["", {a, 1, 8}];
medianTicks[[6]] = " ";
medianTicks[[7]] = " ";
hazardRatio = "s = 1.8/16.0";
pValue = 1.2 * 10^-4;
columnLabel = "";
textCoordinates =
  {{0.09 * months, 0.1}, {0.865 * months, 0.9}, {0.09 * months, 0.35}, {0.09 * months, 0.6}};
textCoordinates[[2]] = {0.865 * months, 0.6 - 0.03};
textCoordinates[[4]] = {0.865 * months, 0.35 - 0.03};
g9 = Show[display[annotation, displayNumber, xplotlabel,
    xlabel, ylabel, medianTicks, highlight, columnLabel], ImageSize → 250];

figI =
 GraphicsGrid[{{g1, g2, g3}, {g4, g5, g6}, {g7, g8, g9}}, ImageSize → 750, Spacings → {-67, -12}]
```

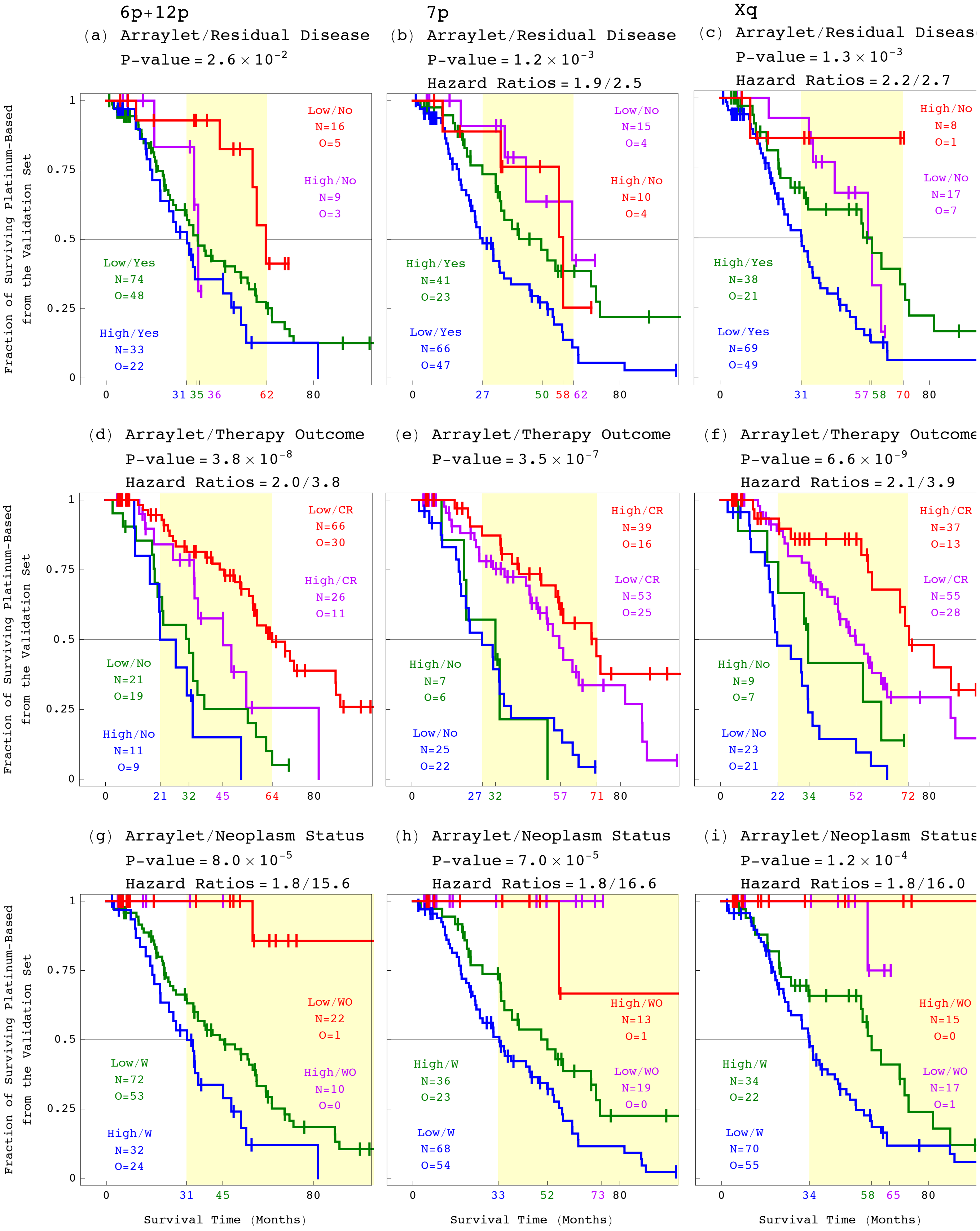


```
Export[path <> "Figure_I.pdf", figI, "PDF", ImageSize → 750, ImageResolution → resolution];
```

```
(* Display the Survival Analyses of the Discovery and Validation Patients Classified by the Three
Probelets and Arraylets *)

title = "Fraction of Surviving Patients";

stream = path <> "Discovery_Patients.txt";
annotations = Import[stream, "Table"];
{patients, annotationsNumber} = Dimensions[annotations] - {1, 1};
annotationNames = Take[annotations, 1, {2, annotationsNumber + 1}][[1]];
annotations = Drop[Import[stream, "Table"], 1, 1];
Clear[stream];

times = Table[
   If[annotationColumn["Days_Death"][[a]] == "Null",
    If[annotationColumn["Days_Followup"][[a]] == "Null",
     "Null",
     annotationColumn["Days_Followup"][[a]] / monthConversion],
    annotationColumn["Days_Death"][[a]] / monthConversion],
   {a, 1, patients}];
status = Table[If[annotationColumn["Days_Death"][[a]] == "Null",
    If[annotationColumn["Days_Followup"][[a]] == "Null", 2, 0], 1],
   {a, 1, patients}];
positionNull = Position[status, 2];
Do[{
  annotations = Drop[annotations, positionNull[[a]]],
  times = Drop[times, positionNull[[a]]],
  status = Drop[status, positionNull[[a]]]},
 {a, Dimensions[positionNull][[1]], 1, -1}]
patients = patients - Dimensions[positionNull][[1]];

displayNumber = 3;
annotation = "Three_Probelets_(Comb.)";
firstAnnotation = "C";
secondAnnotation = "A";
thirdAnnotation = "B";
highlight = True;
xplotlabel = "(a)";
xlabel = False;
ylabel = "from the Discovery Set";
medianTicks = Table["", {a, 1, 8}];
medianTicks[[3]] = "  ";
hazardRatio = "";
columnLabel = "";
pValue = 1.4 * 10^-4;
textCoordinates = {{0.09 * months, 0.1}, {0.865 * months, 0.9}, {0.09 * months, 0.35}};
g1 = Show[display[annotation, displayNumber, xplotlabel,
    xlabel, ylabel, medianTicks, highlight, columnLabel], ImageSize → 287.5];

title = "Fraction of Surviving Platinum-Based";

stream = path <> "Discovery_Patients.txt";
annotations = Import[stream, "Table"];
{patients, annotationsNumber} = Dimensions[annotations] - {1, 1};
annotationNames = Take[annotations, 1, {2, annotationsNumber + 1}][[1]];
annotations = Drop[Import[stream, "Table"], 1, 1];
Clear[stream];
```

```
times = Table[
   If[annotationColumn["Days_Death"][[a]] == "Null",
    If[annotationColumn["Days_Followup"][[a]] == "Null",
     "Null",
     annotationColumn["Days_Followup"][[a]] / monthConversion],
    annotationColumn["Days_Death"][[a]] / monthConversion],
   {a, 1, patients}];
status = Table[
   If[annotationColumn["Platinum-Based_Chemotherapy"][[a]] == "Null", 2,
    If[annotationColumn["Platinum-Based_Chemotherapy"][[a]] == "No", 2,
     If[annotationColumn["Days_Death"][[a]] == "Null",
      If[annotationColumn["Days_Followup"][[a]] == "Null", 2, 0], 1]]],
   {a, 1, patients}];
positionNull = Position[status, 2];
Do[{
  annotations = Drop[annotations, positionNull[[a]]],
  times = Drop[times, positionNull[[a]]],
  status = Drop[status, positionNull[[a]]]},
 {a, Dimensions[positionNull][[1]], 1, -1}]
patients = patients - Dimensions[positionNull][[1]];

displayNumber = 3;
annotation = "Three_Probelets_(Comb.)";
firstAnnotation = "C";
secondAnnotation = "A";
thirdAnnotation = "B";
highlight = True;
xplotlabel = "(b)";
xlabel = False;
ylabel = "Chemo. Patients from the Discovery Set";
medianTicks = Table["", {a, 1, 8}];
medianTicks[[3]] = " ";
hazardRatio = "";
pValue = 6.5 * 10^-4;
columnLabel = "";
textCoordinates = {{0.09 * months, 0.1}, {0.865 * months, 0.9}, {0.09 * months, 0.35}};
g2 = Show[display[annotation, displayNumber, xplotlabel,
    xlabel, ylabel, medianTicks, highlight, columnLabel], ImageSize → 287.5];

title = "Fraction of Surviving Patients";

stream = path <> "Validation_Patients.txt";
annotations = Import[stream, "Table"];
{patients, annotationsNumber} = Dimensions[annotations] - {1, 1};
annotationNames = Take[annotations, 1, {2, annotationsNumber + 1}][[1]];
annotations = Drop[Import[stream, "Table"], 1, 1];
Clear[stream];
```

```
times = Table[
   If[annotationColumn["Days_Death"][[a]] == "Null",
    If[annotationColumn["Days_Followup"][[a]] == "Null",
     "Null",
     annotationColumn["Days_Followup"][[a]] / monthConversion],
    annotationColumn["Days_Death"][[a]] / monthConversion],
   {a, 1, patients}];
status = Table[If[annotationColumn["Days_Death"][[a]] == "Null",
    If[annotationColumn["Days_Followup"][[a]] == "Null", 2, 0], 1],
   {a, 1, patients}];
positionNull = Position[status, 2];
Do[{
  annotations = Drop[annotations, positionNull[[a]]],
  times = Drop[times, positionNull[[a]]],
  status = Drop[status, positionNull[[a]]]},
 {a, Dimensions[positionNull][[1]], 1, -1}]
patients = patients - Dimensions[positionNull][[1]];

displayNumber = 3;
annotation = "Three_Arraylets_(Comb.)";
firstAnnotation = "C";
secondAnnotation = "A";
thirdAnnotation = "B";
highlight = True;
xplotlabel = "(c)";
xlabel = True;
ylabel = "from the Validation Set";
medianTicks = Table["", {a, 1, 8}];
hazardRatio = "";
pValue = 8.1 * 10^-4;
columnLabel = "";
textCoordinates = {{0.09 * months, 0.1}, {0.865 * months, 0.9}, {0.09 * months, 0.35}};
g3 = Show[display[annotation, displayNumber, xplotlabel,
    xlabel, ylabel, medianTicks, highlight, columnLabel], ImageSize → 287.5];

title = "Fraction of Surviving Platinum-Based";

stream = path <> "Validation_Patients.txt";
annotations = Import[stream, "Table"];
{patients, annotationsNumber} = Dimensions[annotations] - {1, 1};
annotationNames = Take[annotations, 1, {2, annotationsNumber + 1}][[1]];
annotations = Drop[Import[stream, "Table"], 1, 1];
Clear[stream];
```

```
times = Table[
   If[annotationColumn["Days_Death"][[a]] == "Null",
    If[annotationColumn["Days_Followup"][[a]] == "Null",
     "Null",
     annotationColumn["Days_Followup"][[a]] / monthConversion],
    annotationColumn["Days_Death"][[a]] / monthConversion],
   {a, 1, patients}];
status = Table[
   If[annotationColumn["Platinum-Based_Chemotherapy"][[a]] == "Null", 2,
    If[annotationColumn["Platinum-Based_Chemotherapy"][[a]] == "No", 2,
     If[annotationColumn["Days_Death"][[a]] == "Null",
      If[annotationColumn["Days_Followup"][[a]] == "Null", 2, 0], 1]]],
   {a, 1, patients}];
positionNull = Position[status, 2];
Do[{
  annotations = Drop[annotations, positionNull[[a]]],
  times = Drop[times, positionNull[[a]]],
  status = Drop[status, positionNull[[a]]]},
 {a, Dimensions[positionNull][[1]], 1, -1}]
patients = patients - Dimensions[positionNull][[1]];

displayNumber = 3;
annotation = "Three_Arraylets_(Comb.)";
firstAnnotation = "C";
secondAnnotation = "A";
thirdAnnotation = "B";
highlight = True;
xplotlabel = "(d)";
xlabel = True;
ylabel = "Chemo. Patients from the Validation Set";
medianTicks = Table["", {a, 1, 8}];
hazardRatio = "";
pValue = 1.0 * 10^-3;
columnLabel = "";
textCoordinates = {{0.09 * months, 0.1}, {0.865 * months, 0.9}, {0.09 * months, 0.35}};
g4 = Show[display[annotation, displayNumber, xplotlabel,
    xlabel, ylabel, medianTicks, highlight, columnLabel], ImageSize → 287.5];
```

```
fig4 = GraphicsGrid[{{g1, g2}, {g3, g4}}, ImageSize → 575, Spacings → {0, -14}]
```

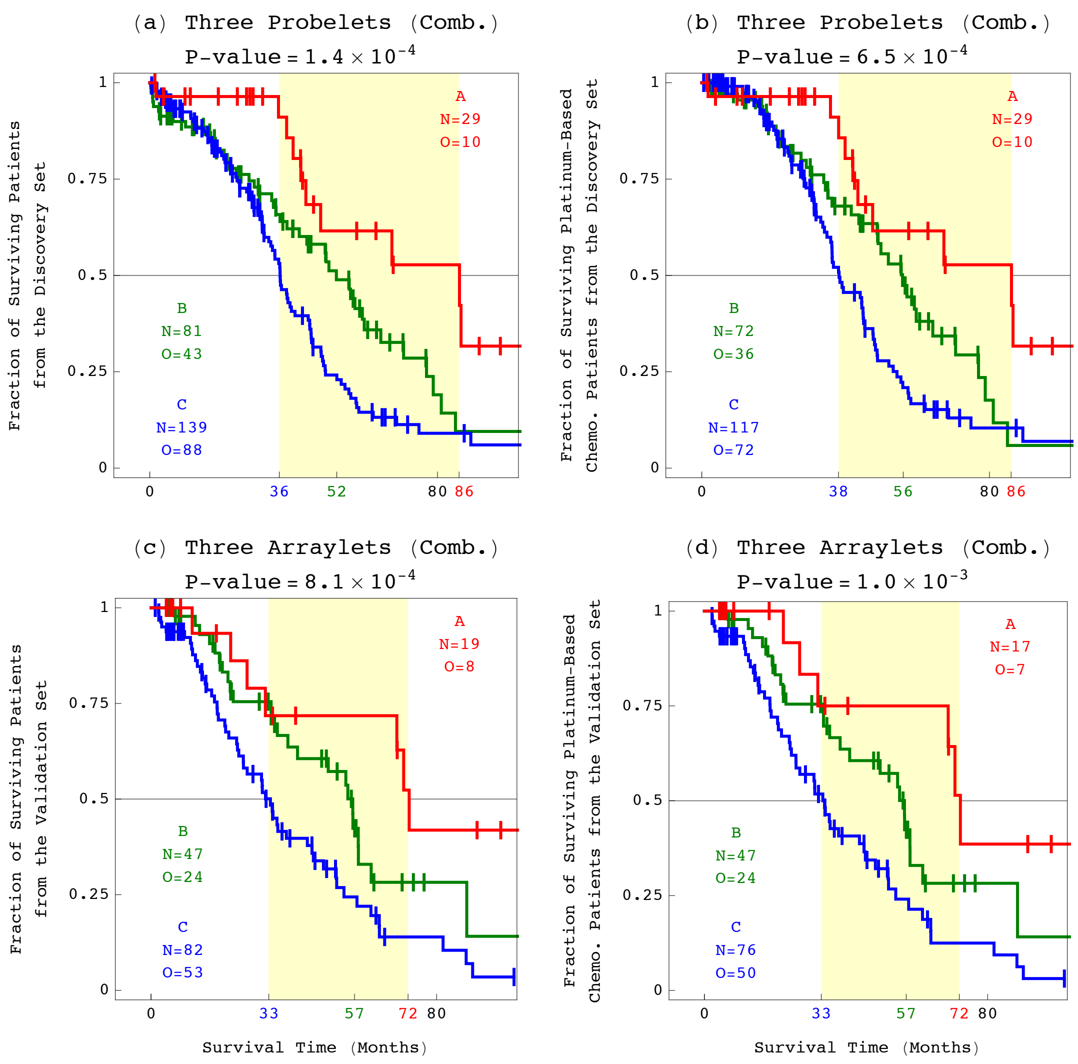


```
Export[path <> "Figure_4.pdf", fig4, "PDF", ImageSize → 575, ImageResolution → resolution];
```

```
(* Display the Survival Analyses of the Novel Focal Copy-Number Alterations *)

(* Read TCGA Annotations of the Discovery Patients *)

title = "Fraction of Surviving Patients from";

stream = path <> "Discovery_Patients.txt";
annotations = Import[stream, "Table"];
{patients, annotationsNumber} = Dimensions[annotations] - {1, 1};
annotationNames = Take[annotations, 1, {2, annotationsNumber + 1}][[1]];
annotations = Drop[Import[stream, "Table"], 1, 1];
Clear[stream];

colIdx = {Position[annotationNames, "Days_Death"][[1]][[1]],
   Position[annotationNames, "Days_Followup"][[1]][[1]],
   Position[annotationNames, "6p+12p_Segment_10_(SOX5)"][[1]][[1]],
   Position[annotationNames, "6p+12p_Segment_11_(SOX5)"][[1]][[1]],
   Position[annotationNames, "6p+12p_Segment_13_(ITPR2)"][[1]][[1]],
   Position[annotationNames, "6p+12p_Segment_14_(ASUN)"][[1]][[1]],
   Position[annotationNames, "7p_Segment_4_(RPA3)"][[1]][[1]],
   Position[annotationNames, "Xq_Segment_10_(PABPC5)"][[1]][[1]]};

annotationNames = annotationNames[[colIdx]];
annotations = annotations[[All, colIdx]];

times = Table[
   If[annotationColumn["Days_Death"][[a]] == "Null",
    If[annotationColumn["Days_Followup"][[a]] == "Null",
     "Null",
     annotationColumn["Days_Followup"][[a]] / monthConversion],
    annotationColumn["Days_Death"][[a]] / monthConversion],
   {a, 1, patients}];
status = Table[If[annotationColumn["Days_Death"][[a]] == "Null",
    If[annotationColumn["Days_Followup"][[a]] == "Null", 2, 0], 1],
   {a, 1, patients}];
positionNull = Position[status, 2];
Do[{
  annotations = Drop[annotations, positionNull[[a]]],
  times = Drop[times, positionNull[[a]]],
  status = Drop[status, positionNull[[a]]]},
 {a, Dimensions[positionNull][[1]], 1, -1}]
patients = patients - Dimensions[positionNull][[1]];

annotationsDisc = annotations;
timesDisc = times;
statusDisc = status;
patientsDisc = patients;

(* Read TCGA Annotations of the Validation Patients *)

stream = path <> "Validation_Patients.txt";
annotations = Import[stream, "Table"];
{patients, annotationsNumber} = Dimensions[annotations] - {1, 1};
annotationNames = Take[annotations, 1, {2, annotationsNumber + 1}][[1]];
annotations = Drop[Import[stream, "Table"], 1, 1];
Clear[stream];
```

```
colIdx = {Position[annotationNames, "Days_Death"][[1]][[1]],
   Position[annotationNames, "Days_Followup"][[1]][[1]],
   Position[annotationNames, "6p+12p_Segment_10_(SOX5)"][[1]][[1]],
   Position[annotationNames, "6p+12p_Segment_11_(SOX5)"][[1]][[1]],
   Position[annotationNames, "6p+12p_Segment_13_(ITPR2)"][[1]][[1]],
   Position[annotationNames, "6p+12p_Segment_14_(ASUN)"][[1]][[1]],
   Position[annotationNames, "7p_Segment_4_(RPA3)"][[1]][[1]],
   Position[annotationNames, "Xq_Segment_10_(PABPC5)"][[1]][[1]]};

annotationNames = annotationNames[[colIdx]];
annotations = annotations[[All, colIdx]];

times = Table[
   If[annotationColumn["Days_Death"][[a]] == "Null",
    If[annotationColumn["Days_Followup"][[a]] == "Null",
     "Null",
     annotationColumn["Days_Followup"][[a]] / monthConversion],
    annotationColumn["Days_Death"][[a]] / monthConversion],
   {a, 1, patients}];
status = Table[If[annotationColumn["Days_Death"][[a]] == "Null",
    If[annotationColumn["Days_Followup"][[a]] == "Null", 2, 0], 1],
   {a, 1, patients}];
positionNull = Position[status, 2];
Do[{
  annotations = Drop[annotations, positionNull[[a]]],
  times = Drop[times, positionNull[[a]]],
  status = Drop[status, positionNull[[a]]]},
 {a, Dimensions[positionNull][[1]], 1, -1}]
patients = patients - Dimensions[positionNull][[1]];

annotationsVal = annotations;
timesVal = times;
statusVal = status;
patientsVal = patients;

(* Join TCGA Annotations of the Discovery and Validation Patients *)

annotations = Join[annotationsDisc, annotationsVal];
times = Join[timesDisc, timesVal];
status = Join[statusDisc, statusVal];
patients = patientsDisc + patientsVal;

(* Create KM Curves for Patients Classified by mRNA Expression Levels *)

displayNumber = 2;
fourthMedian = 0;
annotation = "6p+12p_Segment_10_(SOX5)";
firstAnnotation = "Gain";
secondAnnotation = "No_CNA";
highlight = True;
xplotlabel = "(a)";
xlabel = False;
ylabel = "the Discovery and Validation Sets";
columnLabel = "";
medianTicks = Table["", {a, 1, 8}];
pValue = 5.2 * 10^-5;
hazardRatio = " = 1.9";
textCoordinates = {{0.09 * months, 0.1}, {0.865 * months, 0.9}};
g1 = Show[display[annotation, displayNumber, xplotlabel,
    xlabel, ylabel, medianTicks, highlight, columnLabel], ImageSize → 250];
```

```
displayNumber = 2;
fourthMedian = 0;
annotation = "6p+12p_Segment_11_(SOX5)";
firstAnnotation = "Gain";
secondAnnotation = "No_CNA";
highlight = True;
xplotlabel = "(b)";
xlabel = False;
ylabel = "False";
columnLabel = "";
medianTicks = Table["", {a, 1, 8}];
pValue = 2.5 * 10^-3;
hazardRatio = " = 1.6";
textCoordinates = {{0.09 * months, 0.1}, {0.865 * months, 0.9}};
g2 = Show[display[annotation, displayNumber, xplotlabel,
    xlabel, ylabel, medianTicks, highlight, columnLabel], ImageSize → 250];

displayNumber = 2;
fourthMedian = 0;
annotation = "6p+12p_Segment_13_(ITPR2)";
firstAnnotation = "Gain";
secondAnnotation = "No_CNA";
highlight = True;
xplotlabel = "(c)";
xlabel = False;
ylabel = "False";
columnLabel = "";
medianTicks = Table["", {a, 1, 8}];
pValue = 1.6 * 10^-3;
hazardRatio = " = 1.6";
textCoordinates = {{0.09 * months, 0.1}, {0.865 * months, 0.9}};
g3 = Show[display[annotation, displayNumber, xplotlabel,
    xlabel, ylabel, medianTicks, highlight, columnLabel], ImageSize → 250];

displayNumber = 2;
fourthMedian = 0;
annotation = "6p+12p_Segment_14_(ASUN)";
firstAnnotation = "Gain";
secondAnnotation = "No_CNA";
highlight = True;
xplotlabel = "(d)";
xlabel = True;
ylabel = "the Discovery and Validation Sets";
columnLabel = "";
medianTicks = Table["", {a, 1, 8}];
pValue = 5.7 * 10^-3;
hazardRatio = " = 1.5";
textCoordinates = {{0.09 * months, 0.1}, {0.865 * months, 0.9}};
g4 = Show[display[annotation, displayNumber, xplotlabel,
    xlabel, ylabel, medianTicks, highlight, columnLabel], ImageSize → 250];
```

```
displayNumber = 2;
fourthMedian = 0;
annotation = "7p_Segment_4_(RPA3)";
firstAnnotation = "No_CNA";
secondAnnotation = "Loss";
highlight = True;
xplotlabel = "(e)";
xlabel = True;
ylabel = "False";
columnLabel = "";
medianTicks = Table["", {a, 1, 8}];
pValue = 4.8 * 10^-3;
hazardRatio = " = 1.6";
textCoordinates = {{0.09 * months, 0.1}, {0.865 * months, 0.9}};
g5 = Show[display[annotation, displayNumber, xplotlabel,
    xlabel, ylabel, medianTicks, highlight, columnLabel], ImageSize → 250];

displayNumber = 2;
fourthMedian = 0;
annotation = "Xq_Segment_10_(PABPC5)";
firstAnnotation = "No_CNA";
secondAnnotation = "Loss";
highlight = True;
xplotlabel = "(f)";
xlabel = True;
ylabel = "False";
columnLabel = "";
medianTicks = Table["", {a, 1, 8}];
pValue = 3.3 * 10^-2;
hazardRatio = " = 1.4";
textCoordinates = {{0.09 * months, 0.1}, {0.865 * months, 0.9}};
g6 = Show[display[annotation, displayNumber, xplotlabel,
    xlabel, ylabel, medianTicks, highlight, columnLabel], ImageSize → 250];
```

```
figJ = GraphicsGrid[{{g1, g2, g3}, {g4, g5, g6}}, ImageSize → 750, Spacings → {-67, -12}]
```

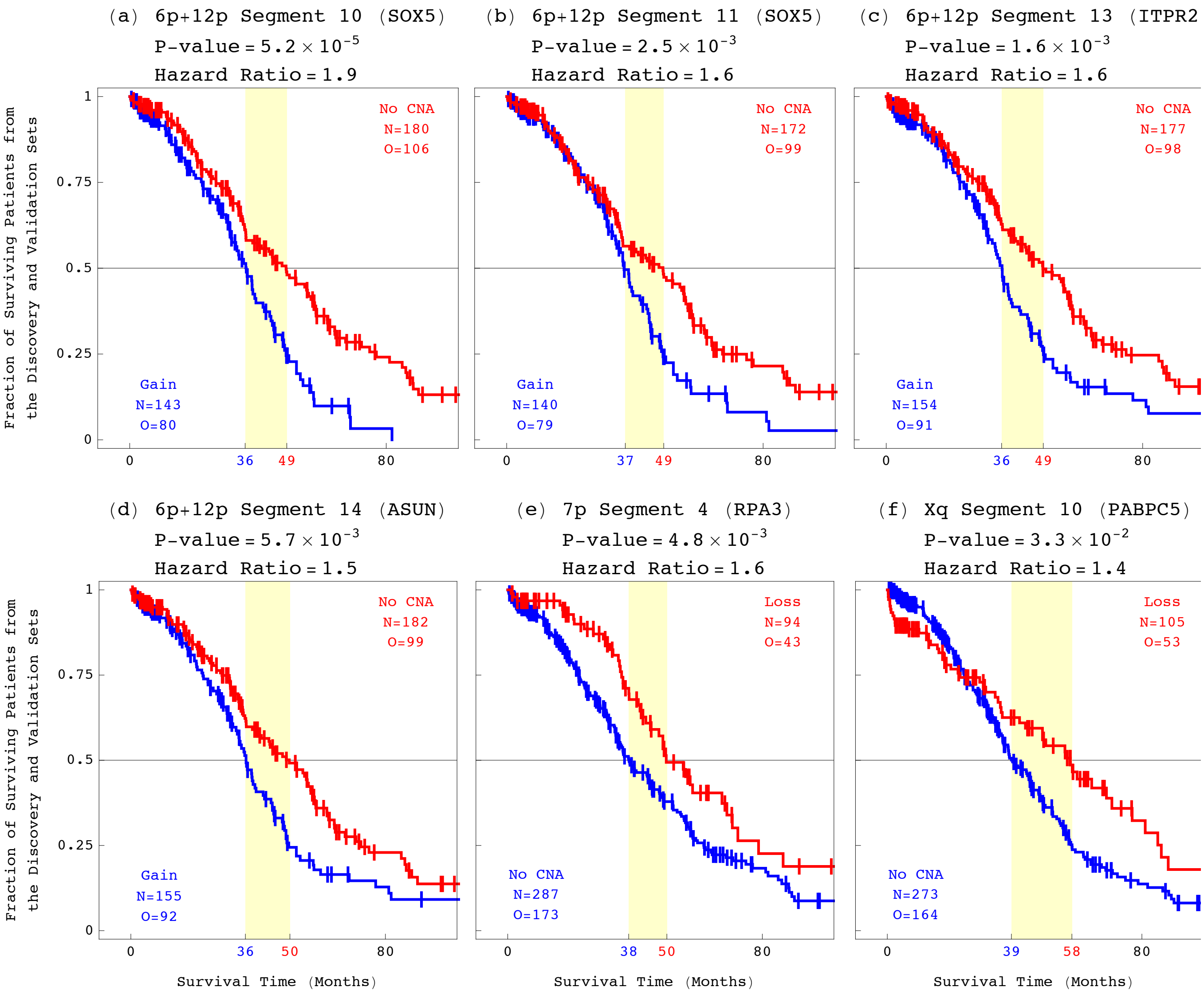


```
Export[path <> "Figure_J.pdf", figJ, "PDF", ImageSize → 750, ImageResolution → resolution];
```

```
(* Display Differential mRNA, microRNA, and Protein Expression between the Tensor GSVD Classes *)
```

```
(* Define the Boxplot Display *)

boxPlot[data_, dataLabels_, frameLabels_, frameTicks_, plotLabel_,
  plotRange_, text_, alignmnt_, text1_, colors_] := {nData = Dimensions[data][[1]];
  pValue = MannWhitneyTest[{data[[1]], data[[2]]}];
  g = Table[{}, {a, 1, nData}];
  bins = Flatten[Table[
     Labeled[{}, Style[ColumnForm[{dataLabels[[a]], "N=" <> ToString[Length[data[[a]]]]},
        Center], colors[[a]]]], {a, 2, nData}]];
  Do[g[[a]] = BoxWhiskerChart[Flatten[If[a == 1, {Labeled[data[[a]],
          Style[ColumnForm[{dataLabels[[a]], "N=" <> ToString[Length[data[[a]]]]}, Center],
           colors[[a]]]], If[nData == 3, {bins[[1]], bins[[2]]}, bins[[1]]]},
        {Table[If[b == a, Labeled[data[[b]], ColumnForm[{dataLabels[[b]],
               "N=" <> ToString[Length[data[[b]]]]}, Center]], {}], {b, 1, nData}]}], 1],
     {"Median", {"Outliers", "●", colors[[a]]}, {"MedianMarker", colors[[a]]},
      {"Whiskers", colors[[a]]}, {"Fences", colors[[a]]}},
     PlotLabel → Style[Column[{plotLabel[[1]], text1 <> plotLabel[[2]],
          StringJoin[text <> "P-value = ", ToString[TraditionalForm[ScientificForm[pValue, 2,
              NumberPoint →
               If[Dimensions[Characters[ToString[NumberForm[pValue, 2, NumberFormat → (#1 &),
                        ExponentFunction → (# &)]]]][[1]] < 3, ".0", "."]]]]]}, alignmnt],
        SingleLetterItalics → False], If[Length[plotRange] > 0, PlotRange → plotRange,
      PlotRange → {{0.48, Automatic}, {-0.22, 0.22}}],
     FrameTicks → {{frameTicks, None}, {None, None}},
     FrameLabel → frameLabels,
     ImageSize → 250, BarSpacing → Tiny,
     ChartBaseStyle → Directive[EdgeForm[{Thick, colors[[a]]}], White, Thick],
     AspectRatio → 1,
     BaseStyle → {FontFamily → "Courier", FontSize → 10}], {a, 1, nData}];
  Show[Table[g[[a]], {a, 1, nData}]]}

(* Determine the Tensor GSVD Classes and Display the Boxplots *)

plotBarChart[minval_, maxval_, segtext_, grp1text_, grp2text_, exptext_, xlabel_, ylabel_,
  yticks_, figtitle_, patientFile_, text_, alignmnt_: Left, text1_: "", colors_] :=
 Module[{genepos, ncnapos, expressionpos, nocna, cna, nullPosition},
  (genepos = Position[patientFile[[1]], segtext][[1, 1]];
   ncnapos = Flatten[Position[patientFile[[All, genepos]], grp1text]];
   expressionpos = Position[patientFile[[1]], exptext][[1, 1]];
   nocna = Flatten[patientFile[[ncnapos, expressionpos]]];
   nullPosition = Position[nocna, "Null"];
   If[nullPosition ≠ {}, nocna = Delete[nocna, nullPosition];];
   ncnapos = Flatten[Position[patientFile[[All, genepos]], grp2text]];
   cna = Flatten[patientFile[[ncnapos, expressionpos]]];
   nullPosition = Position[cna, "Null"];
   If[nullPosition ≠ {}, cna = Delete[cna, nullPosition];];
   boxPlot[{cna, nocna}, {StringReplace[grp2text, "_" → " "],
      StringReplace[grp1text, "_" → " "]}, {xlabel, ylabel}, yticks, figtitle,
     {{0.48, Automatic}, {minval, maxval}}, text, alignmnt, text1, colors][[1]])]
```

```
(* Read TCGA Annotations of the Patients *)

patientFile = Join[Import[path <> "Discovery_Patients.txt", "TSV"],
   Import[path <> "Validation_Patients.txt", "TSV"][[2 ;;]]];

(* Display Differential mRNA Expression *)

minval = 2.75;
maxval = 10.75;
colors = {RGBColor[0, 0, 1], RGBColor[1, 0, 0], RGBColor[0, 0, 0], RGBColor[0, 1, 0]};
g1 = plotBarChart[minval, maxval, "Probelet_6p+12p_(Coeff.)", "Low", "High", "mRNA_(TNF)",
   "  ", "Relative mRNA Expression", Table[{i, ToString[i]}, {i, 4, 10, 2}],
   {"(a) 6p+12p Probelet (Coeff.)", "TNF"}, patientFile, "    ", Left, "    ", colors];
g2 = plotBarChart[minval, maxval, "Probelet_6p+12p_(Coeff.)", "Low",
   "High", "mRNA_(MAPK14)", "  ", "  ", Table[{i, "  "}, {i, 4, 10, 2}],
   {"(b) 6p+12p Probelet (Coeff.)", "MAPK14"}, patientFile, "    ", Left, "    ", colors];
g3 = plotBarChart[minval, maxval, "Probelet_6p+12p_(Coeff.)", "Low",
   "High", "mRNA_(CDKN1A)", "  ", "  ", Table[{i, "  "}, {i, 4, 10, 2}],
   {"(c) 6p+12p Probelet (Coeff.)", "CDKN1A"}, patientFile, "    ", Left, "    ", colors];

g4 = plotBarChart[minval, maxval, "Probelet_6p+12p_(Coeff.)", "Low", "High",
   "mRNA_(RAD51AP1)", "  ", "Relative mRNA Expression", Table[{i, ToString[i]}, {i, 4, 10, 2}],
   {"(d) 6p+12p Probelet (Coeff.)", "RAD51AP1"}, patientFile, "    ", Left, "    ", colors];
g5 = plotBarChart[minval, maxval, "Probelet_6p+12p_(Coeff.)", "Low",
   "High", "mRNA_(ITPR2)", "  ", "  ", Table[{i, "  "}, {i, 4, 10, 2}],
   {"(e) 6p+12p Probelet (Coeff.)", "ITPR2"}, patientFile, "    ", Left, "    ", colors];
g6 = plotBarChart[minval, maxval, "Probelet_6p+12p_(Coeff.)", "Low",
   "High", "mRNA_(ASUN)", "  ", "  ", Table[{i, "  "}, {i, 4, 10, 2}],
   {"(f) 6p+12p Probelet (Coeff.)", "ASUN"}, patientFile, "    ", Left, "    ", colors];

colors = {RGBColor[1, 0, 0], RGBColor[0, 0, 1], RGBColor[0, 0, 0], RGBColor[0, 1, 0]};
g7 = plotBarChart[minval, maxval, "Probelet_7p_(Coeff.)", "Low", "High", "mRNA_(RPA3)",
  "  ", "Relative mRNA Expression", Table[{i, ToString[i]}, {i, 4, 10, 2}],
  {"(g) 7p Probelet (Coeff.)", "RPA3"}, patientFile, "    ", Left, "    ", colors];
g8 = plotBarChart[minval, maxval, "Probelet_7p_(Coeff.)", "Low",
   "High", "mRNA_(POLD2)", "  ", "  ", Table[{i, "  "}, {i, 4, 10, 2}],
   {"(h) 7p Probelet (Coeff.)", "POLD2"}, patientFile, "    ", Left, "    ", colors];
g9 = plotBarChart[minval, maxval, "Probelet_Xq_(Coeff.)", "Low", "High",
   "mRNA_(BCAP31)", "  ", "  ", Table[{i, "  "}, {i, 4, 10, 2}],
   {"(i) Xq Probelet (Coeff.)", "BCAP31"}, patientFile, "    ", Left, "    ", colors];

figK =
 GraphicsGrid[{{g1, g2, g3}, {g4, g5, g6}, {g7, g8, g9}}, ImageSize → 750, Spacings → {-67, 0}]
```

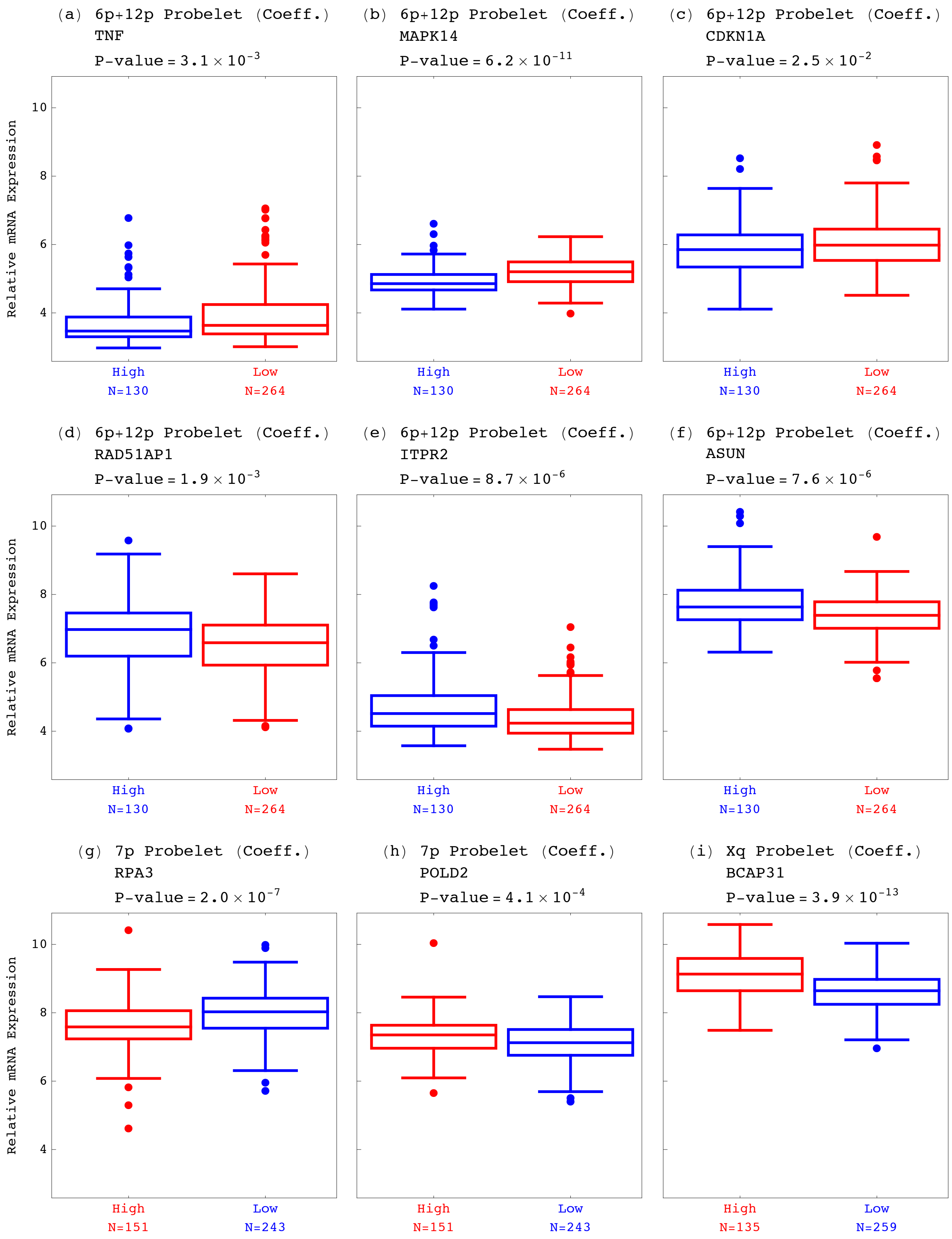


```
Export[path <> "Figure_K.pdf", figK, "PDF", ImageSize → 750];
```

```
(* Display Differential microRNA Expression *)

minval = 3.5;
maxval = 15.2;
colors = {RGBColor[0, 0, 1], RGBColor[1, 0, 0], RGBColor[0, 0, 0], RGBColor[0, 1, 0]};
g1 = plotBarChart[minval, maxval,
   "Probelet_6p+12p_(Coeff.)", "Low", "High", "microRNA_(hsa-miR-877*)",
   "  ", "Relative microRNA Expression", Table[{i, ToString[i]}, {i, 4, 14, 2}],
   {"(a) 6p+12p Probelet (Coeff.)", "miR-877*"}, patientFile, "    ", Left, "    ", colors];

g2 = plotBarChart[minval, maxval, "Probelet_6p+12p_(Coeff.)", "Low",
   "High", "microRNA_(hsa-miR-200c)", "  ", "  ", Table[{i, "  "}, {i, 4, 14, 2}],
   {"(b) 6p+12p Probelet (Coeff.)", "miR-200c"}, patientFile, "    ", Left, "    ", colors];
g3 = plotBarChart[minval, maxval, "Probelet_6p+12p_(Coeff.)", "Low", "High",
   "microRNA_(hsa-miR-200c*)", "  ", "  ", Table[{i, "  "}, {i, 4, 14, 2}],
   {"(c) 6p+12p Probelet (Coeff.)", "miR-200c*"}, patientFile, "    ", Left, "    ", colors];

g4 = plotBarChart[minval, maxval,
   "Probelet_6p+12p_(Coeff.)", "Low", "High", "microRNA_(hsa-miR-141)", "  ",
   "Relative microRNA Expression", Table[{i, ToString[i]}, {i, 4, 14, 2}],
   {"(d) 6p+12p Probelet (Coeff.)", "miR-141"}, patientFile, "    ", Left, "    ", colors];
g5 = plotBarChart[minval, maxval, "Probelet_6p+12p_(Coeff.)", "Low", "High",
   "microRNA_(hsa-miR-141*)", "  ", "  ", Table[{i, "  "}, {i, 4, 14, 2}],
   {"(e) 6p+12p Probelet (Coeff.)", "miR-141*"}, patientFile, "    ", Left, "    ", colors];

colors = {RGBColor[1, 0, 0], RGBColor[0, 0, 1], RGBColor[0, 0, 0], RGBColor[0, 1, 0]};
g6 = plotBarChart[minval, maxval,
  "Probelet_Xq_(Coeff.)", "Low", "High", "microRNA_(hsa-miR-888)", "  ",
  "Relative microRNA Expression", Table[{i, ToString[i]}, {i, 4, 14, 2}],
  {"(f) Xq Probelet (Coeff.)", "miR-888"}, patientFile, "    ", Left, "    ", colors];
g7 = plotBarChart[minval, maxval, "Probelet_Xq_(Coeff.)", "Low", "High",
  "microRNA_(hsa-miR-224)", "  ", "  ", Table[{i, "  "}, {i, 4, 14, 2}],
  {"(g) Xq Probelet (Coeff.)", "miR-224"}, patientFile, "    ", Left, "    ", colors];
g8 = plotBarChart[minval, maxval, "Probelet_Xq_(Coeff.)", "Low", "High",
  "microRNA_(hsa-miR-452)", "  ", "  ", Table[{i, "  "}, {i, 4, 14, 2}],
  {"(h) Xq Probelet (Coeff.)", "miR-452"}, patientFile, "    ", Left, "    ", colors];

figL = GraphicsGrid[{{g1, g2, g3}, {g4, g5}, {g6, g7, g8}}, ImageSize → 750, Spacings → {-67, 0}]
```

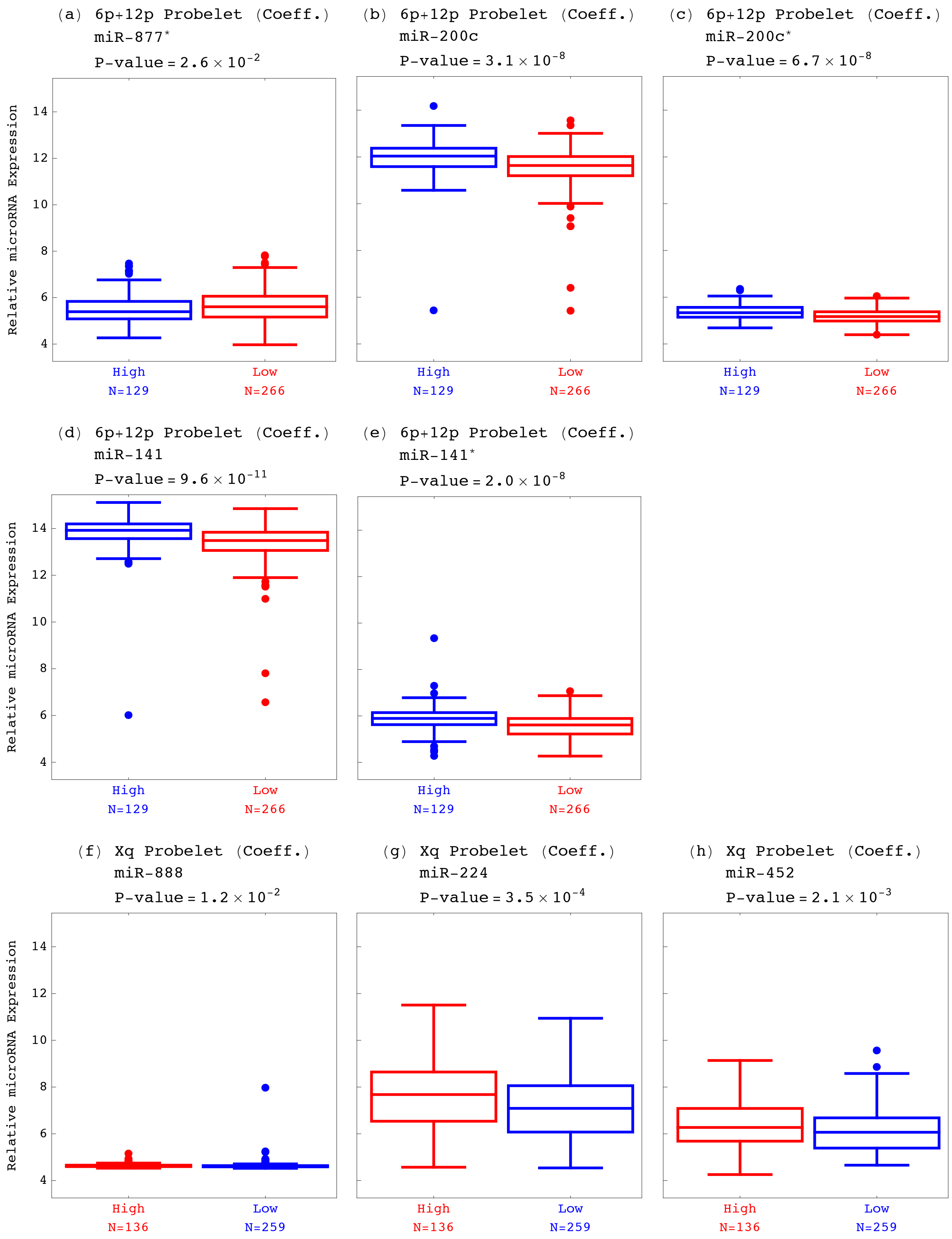


```
Export[path <> "Figure_L.pdf", figL, "PDF", ImageSize → 750];
```

```
(* Display Differential Protein Expression *)

minval = -2.5;
maxval = 1.5;
colors = {RGBColor[0, 0, 1], RGBColor[1, 0, 0], RGBColor[0, 0, 0], RGBColor[0, 1, 0]};
g1 = plotBarChart[minval, maxval,
   "Probelet_6p+12p_(Coeff.)", "Low", "High", "Protein_(MAPK14)", "   ",
   "Relative Protein Expression", Table[{i, ToString[i]}, {i, -2, 1, 1}],
   {"(a) 6p+12p Probelet (Coeff.)", "MAPK14"}, patientFile, "    ", Left, "    ", colors];

g2 = plotBarChart[minval, maxval, "Probelet_6p+12p_(Coeff.)", "Low",
   "High", "Protein_(CDKN1B)", "   ", "   ", Table[{i, "   "}, {i, -2, 1, 1}],
   {"(b) 6p+12p Probelet (Coeff.)", "CDKN1B"}, patientFile, "    ", Left, "    ", colors];

figM = GraphicsGrid[{{g1, g2}}, ImageSize → 500, Spacings → {-70, 0}]
```

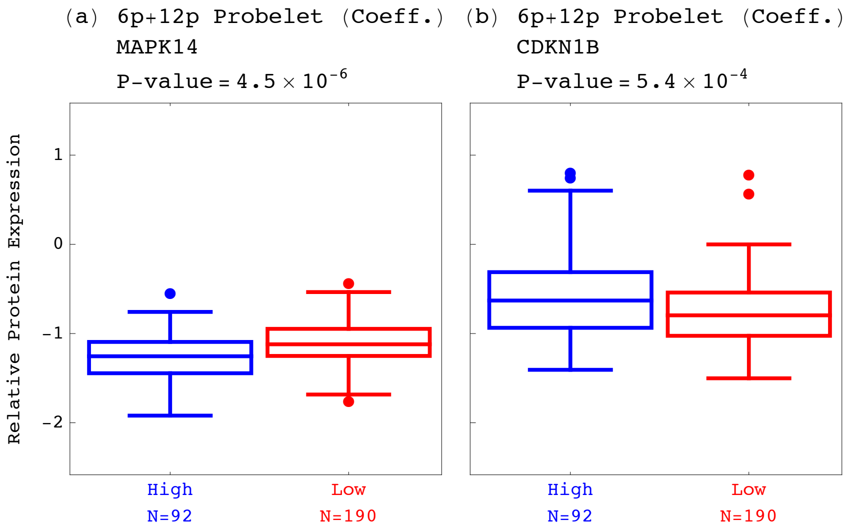


```
Export[path <> "Figure_M.pdf", figM, "PDF", ImageSize → 500];
```

```
(* Display Tensor GSVD of Patient- and Platform-Matched Tumor and Normal aCGH Datasets *)
```

```
(* Create a Raster *)

rasterFileGeneration[Data_, rastercontrast_] := Module[{probes, meanData, arrays, raster}, (
   probes = Dimensions[Data][[1]];
   meanData = Table[Mean[Data[[a ;; a + step - 1]]], {a, 1, Floor[probes, step], step}];
   {probes, arrays} = Dimensions[meanData];
   raster = Table[If[meanData[[a, b]] > 0, {meanData[[a, b]], 0, 0}, {0, -meanData[[a, b]], 0}],
      {a, probes, 1, -1}, {b, 1, arrays}] * rastercontrast;
   raster
  )]
```

```
(* Fit the Raster in a Display *)

coordinateChange[a1_, b1_, a2_, b2_, list_] :=
 Module[{z}, (z = N[(list - a1) * (b2 - a2) / (b1 - a1) + a2];
   z
  )]
```

```
(* Define the Datasets' Label Coordinates *)

figureVariables[armBandCoordinates_, arm_, probes_, sample_, currentarm_: ""] :=
 Module[{armBands, armValue, xcoordinates, list, bands, indices, num, t, tickpos, xticks,
   patients, patientsString, z, patientLabels, bandLabels, yPosition}, (armValue = arm;
   armBands = armBandCoordinates;
   armBands[[2 ;;, 2]] = armBands[[2 ;;, 2]] + 1;
   armBands[[2 ;;, 3]] = armBands[[2 ;;, 3]] + 1;
   If[currentarm == "6p+12p", (armBands = armBandCoordinates[[1 ;; 20]];
     armValue = arm[[1 ;; Position[arm[[All, 1]], 6][[-1]][[1]]]];
     xcoordinates = Range[Length[armValue]];
     list = Transpose[Join[{xcoordinates}, {armValue[[All, -1]]}]];
     bands = Range[Length[armBands]];
     indices = Flatten[Map[(num = #;
          Position[armValue[[All, 3]], _?(# ≤ armBands[[num, 3]] &)][[-1]]) &,
        Range[2, Length[armBands]]]];
     indices = DeleteDuplicates[indices];
     tickpos =
      Map[If[# ≠ 1, Floor[(indices[[# - 1]] + 1) / step], 0] &, Range[Length[indices]]];
     xticks = Transpose[Join[{tickpos}, {Map["" &, Range[Length[tickpos]]]},
        {Table[{0, 0.05}, {Length[tickpos]}]}]];
     armBands = Join[{armBandCoordinates[[1]]}, armBandCoordinates[[21 ;;]]];
     armValue = arm[[Position[arm[[All, 1]], 12][[1]][[1]] ;;]];
     xcoordinates = Range[Length[armValue]];
     list = Transpose[Join[{xcoordinates}, {armValue[[All, -1]]}]];
     bands = Range[Length[armBands]]; indices = Flatten[Map[(num = #;
          Position[armValue[[All, 3]], _?(# ≤ armBands[[num, 3]] &)][[-1]]) &,
        Range[2, Length[armBands]]]]; indices = DeleteDuplicates[indices];
     tickpos = Map[If[# ≠ 1, Floor[(indices[[# - 1]] + 1) / step], 0] &, Range[Length[indices]]];
     xticks = Join[xticks, Transpose[Join[{tickpos},
         {Map["" &, Range[Length[tickpos]]]}, {Table[{0, 0.05}, {Length[tickpos]}]}]]];
     xticks[[20 ;;, 1]] = xticks[[20 ;;, 1]] + (Position[arm[[All, 1]], 6][[-1]][[1]]) / step;),
    (xcoordinates = Range[Length[arm]];
     list = Transpose[Join[{xcoordinates}, {arm[[All, -1]]}]];
     bands = Range[Length[armBands]]; indices = Flatten[Map[(num = #;
          Position[arm[[All, 2]], _?(# ≤ armBands[[num, 3]] &)][[-1]]) &,
        Range[2, Length[armBands]]]]; indices = DeleteDuplicates[indices];
     tickpos = Map[If[# ≠ 1, Floor[(indices[[# - 1]] + 1) / step], 0] &, Range[Length[indices]]];
     xticks = Transpose[Join[{tickpos}, {Map["" &, Range[Length[armBands] - 1]]},
        {Table[{0, 0.05}, {Length[armBands] - 1}]}]];)];
   patients = {1, 50, 100, 150, 200};
   patientsString = {"1  ", "50 ", "100", "150", "200"};
   z = coordinateChange[1, 249, 13, 26, patients];
   If[sample == "Tumor", patientLabels =
      Map[Text[Style[patientsString[[#]], FontFamily → "Courier", FontSize → ticksize],
         {z[[#]], 40.5 + 13}, {0, 0}, {0, 1}] &, Range[Length[patients]]];, patientLabels =
      Map[Text[Style[patientsString[[#]], FontFamily → "Courier", FontSize → ticksize],
         {z[[#]], 38 + 13 - yspace + 2.5}, {0, 0}, {0, 1}] &, Range[Length[patients]]];];
   If[sample == "Tumor", z = (-1) * coordinateChange[0, probes, -25, 50, xticks[[All, 1]]];,
    z = coordinateChange[0, probes, 25 - yspace, -65 - yspace, xticks[[All, 1]]];];
   xticks[[All, 1]] = z;
   If[currentarm == "6p+12p", (t = Join[Range[19], Range[12]];
     bandLabels = Map[Text[Style[ToString[t[[#]]], FontFamily → "Courier",
          FontSize → ticksize], {-1.25, z[[#]]}, {1, 0}] &, Range[Length[t]]];),
    bandLabels = Map[Text[Style[ToString[#], FontFamily → "Courier", FontSize → ticksize],
         {-1.25, z[[#]]}, {1, 0}] &,
       Range[Length[xticks]]];]; yPosition = bandLabels[[All, 2]];
   {bandLabels, patientLabels, xticks, yPosition, z}
  )]
```

```
(* Define the Datasets Display *)

tensorDisplay[raster1_, raster2_, bandLabels_, yPosition_, xticks_,
  patientLabels_, sample_] := Module[{gtensor}, (If[sample == "Tumor", (
     (* Display the Tumor Datasets *)
     gtensor =
       {raster1, raster2, Graphics[Line[{{0, 25}, {0, -50}, {13, -50}, {13, 25}, {0, 25}}]],
        Graphics[Line[{{13, 38 + 13}, {13, -37 + 13}, {26, -37 + 13}, {26, 38 + 13},
            {13, 38 + 13}}]], Graphics[Line[{{0, 25}, {13, 38 + 13}}]],
        Graphics[{White, Dashed, Line[{{13, 25}, {26, 38 + 13}}]}],
        Graphics[{White, Thin, Line[{{13, -50 + 26}, {13, 25}}]}],
        Graphics[Line[{{13, -50}, {26, -37 + 13}}]], Graphics[Line[{{0, 25}, {-2, 25 + 2}}]],
        Graphics[Text[StyleForm["I", FontSize → 10], {-3, 25 + 3.5}, {0, 0}]],
        Graphics[Text[StyleForm["\!\(\*TagBox[\(II\),
          DisplayForm]\)", FontSize → 10], {-3.5 + 13, 38 + 13 + 3.5}, {0, 0}]], Graphics[
         Text[Subscript[StyleForm["𝒟", FontSize → 12], StyleForm["tumor", FontSize → 8]],
          {19.5, 43 + 13 + 4}, {0, 0}]], Graphics[Line[{{13, 38 + 13}, {13 - 2, 38 + 13 + 2}}]],
        Graphics[Text["Platforms", {(6.5 / 2) + (6.5 / 2) - 5, 39 + 3}, {0, 0}, {1, 2}]],
        Graphics[Text["Arrays", {19.5, 43 + 13.5}]], Graphics[
         Text["Tumor Probes", {-5.5, 25 - 13 + 6.5 - 2}, {0, 0}, {0, 1}]], Graphics[bandLabels],
        Map[Graphics[{Line[{{0, yPosition[[#, 2]]}, {-0.75, yPosition[[#, 2]]}}]}] &,
         Range[Length[xticks]]], Graphics[patientLabels],
        Map[Graphics[{Line[{{patientLabels[[#, 2]][[1]], 38 + 13}, {patientLabels[[#, 2]][[
                1]], 38.75 + 13}}]}] &, Range[Length[patientLabels]]]};), (
     (* Display the Normal Datasets *)
     gtensor = {raster1, raster2,
        Graphics[Line[{{0, 25 - yspace}, {0, -65 - yspace}, {13, -65 - yspace}, {13, 25 - yspace},
            {0, 25 - yspace}}]], Graphics[Line[{{13, 38 + 13 - yspace}, {13, -52 + 13 - yspace},
            {26, -52 + 13 - yspace}, {26, 38 + 13 - yspace}, {13, 38 + 13 - yspace}}]],
        Graphics[{White, Thin, Line[{{13, 25 - yspace}, {13, -52 + 13 - yspace}}]}],
        Graphics[Line[{{0, 25 - yspace}, {13, 38 + 13 - yspace}}]],
        Graphics[{White, Dashed, Line[{{13, 25 - yspace}, {26, 38 + 13 - yspace}}]}],
        Graphics[Line[{{13, -65 - yspace}, {26, -52 + 13 - yspace}}]],
        Graphics[Line[{{0, 25 - yspace}, {-2, 25 + 2 - yspace}}]],
        Graphics[Text[StyleForm["I", FontSize → 10], {-3, 25 + 3.5 - yspace}, {0, 0}]],
        Graphics[Text[StyleForm["\!\(\*TagBox[\(II\),
          DisplayForm]\)", FontSize → 10], {-3.5 + 13, 38 + 13 + 3.5 - yspace}, {0, 0}]],
        Graphics[Text[Subscript[StyleForm["𝒟", FontSize → 12],
            StyleForm["normal", FontSize → 8]], {19.5, 43 + 13 + 4 - yspace}, {0, 0}]],
        Graphics[Line[{{13, 38 + 13 - yspace}, {13 - 2, 38 + 13 + 2 - yspace}}]],
        Graphics[Text["Platforms", {(6.5 / 2) + (6.5 / 2) - 5, 39 + 3 - yspace}, {0, 0}, {1, 2}]],
        Graphics[Text["Arrays", {19.5, 43 + 13.5 - yspace}]], Graphics[Text["Normal Probes",
          {-5.5, 25 - 13 + 6.5 - 5 - yspace}, {0, 0}, {0, 1}]], Graphics[patientLabels],
        Map[Graphics[{Line[{{patientLabels[[#, 2]][[1]], 38 + 13 - yspace},
              {patientLabels[[#, 2]][[1]], 38.75 + 13 - yspace}}]}] &,
         Range[Length[patientLabels]]], Graphics[bandLabels],
        Map[Graphics[{Line[{{0, yPosition[[#, 2]]}, {-0.75, yPosition[[#, 2]]}}]}] &,
         Range[Length[xticks]]]};)];
   gtensor
  )]
```

```
(* Define the Arraylets' Label Coordinates *)

arrayletVariables[zArraylets_, bandLabels_, sample_] :=
 Module[{probes, arrays, arrayletRaster, arrayletDisplay, rasterArraylets,
   bandLabelCoordinates, bandLabelsArraylets, arrayletylblpos, arrayletylbls,
   ylist, yliststring}, ({probes, arrays} = Dimensions[zArraylets];
   arrayletDisplay = zArraylets[[All, Join[Range[1, 10], Range[arrays - 9, arrays]]]];
   arrayletRaster = rasterFileGeneration[arrayletDisplay, rastercontrast];
   If[sample == "Tumor",
    rasterArraylets = Graphics[Raster[arrayletRaster, {{space, -50}, {space + (13 * 2), 25}},
        ColorFunction → RGBColor]];, rasterArraylets = Graphics[Raster[arrayletRaster,
        {{space, -65 - yspace}, {space + (13 * 2), 25 - yspace}}, ColorFunction → RGBColor]];];
   bandLabelsArraylets = bandLabels;
   bandLabelCoordinates = bandLabelsArraylets[[All, 2]];
   bandLabelCoordinates[[All, 1]] = bandLabelCoordinates[[All, 1]] + space;
   bandLabelsArraylets[[All, 2]] = bandLabelCoordinates;
   arrayletylblpos = {{space + 0.65, 25}, {space + (4 * 1.3) + 0.65, 25}, {space + 13 - 0.65, 25},
     {space + 13 + 0.65, 25}, {(space + 26) - (4 * 1.3) - 0.65, 25}, {space + 26 - 0.65, 25}};
   If[sample ≠ "Tumor", arrayletylblpos[[All, 2]] = arrayletylblpos[[All, 2]] - yspace;];
   ylist = {1, 5, 10, 240, 245, 249};
   yliststring = {"1  ", "5  ", "10 ", "489", "494", "498"};
   arrayletylbls =
    Map[Text[Style[yliststring[[#]], FontFamily → "Courier", FontSize → ticksize],
       {arrayletylblpos[[#]][[1]], arrayletylblpos[[#]][[2]] + 2.5},
       {0, 0}, {0, 1}] &, Range[Length[ylist]]];
   {rasterArraylets, bandLabelsArraylets, arrayletylbls, arrayletylblpos}
  )]

(* Define the Arraylets Display *)

arrayletsDisplay[rasterArraylets_, bandLabelsArraylets_, arrayletylbls_, arrayletylblpos_,
  xticks_, yPosition_, sample_] := Module[{gzarraylets}, (If[sample == "Tumor", (
     (* Display the Tumor Arraylets *)
     gzarraylets = {rasterArraylets, Graphics[Line[{{space, 25},
           {space, -50}, {space + (13 * 2), -50}, {space + (13 * 2), 25}, {space, 25}}]],
        Graphics[{White, Line[{{space + 13, 25}, {space + 13, -50}}]}],
        Graphics[Line[{{space, 25}, {space - 2, 25 + 2}}]],
        Graphics[Text[StyleForm["\!\(\*TagBox[SubscriptBox[\(U\), \(tumor\)],
           DisplayForm]\)", FontSize → 12], {space - 2.5, 25 + 4}, {0, 0}]],
        Graphics[Text["Arraylets", {space + 13, 30.5}]],
        Graphics[Text["Tumor Probes", {space - 5.5, 25 - 13 + 6.5 - 2}, {0, 0}, {0, 1}]],
        Graphics[bandLabelsArraylets], Map[Graphics[{Line[{{space, yPosition[[#, 2]]},
                {space - 0.75, yPosition[[#, 2]]}}]}] &, Range[Length[xticks]]],
        Graphics[arrayletylbls],
        Map[Graphics[{Line[{#, {#[[1]], #[[2]] + 0.75}}]}] &, arrayletylblpos]};), (
     (* Display the Normal Arraylets *)
     gzarraylets =
       {rasterArraylets, Graphics[Line[{{space, 25 - yspace}, {space, -65 - yspace},
           {space + (13 * 2), -65 - yspace}, {space + (13 * 2), 25 - yspace}, {space, 25 - yspace}}]],
        Graphics[{White, Line[{{space + 13, 25 - yspace}, {space + 13, -65 - yspace}}]}],
        Graphics[Line[{{space, 25 - yspace}, {space - 2, 25 + 2 - yspace}}]],
        Graphics[Text[StyleForm["\!\(\*TagBox[SubscriptBox[\(U\), \(normal\)],
           DisplayForm]\)", FontSize → 12], {space - 2.5, 25 + 4 - yspace}, {0, 0}]],
        Graphics[Text["Arraylets", {space + 13, 30.5 - yspace}]],
        Graphics[Text["Normal Probes", {space - 5.5, 25 - 13 + 6.5 - 4 - yspace}, {0, 0}, {0, 1}]],
        Graphics[bandLabelsArraylets], Map[Graphics[{Line[{{space, yPosition[[#, 2]]},
                {space - 0.75, yPosition[[#, 2]]}}]}] &, Range[Length[xticks]]],
        Graphics[arrayletylbls],
        Map[Graphics[{Line[{#, {#[[1]], #[[2]] + 0.75}}]}] &, arrayletylblpos]};)];
   gzarraylets
  )]
```

```
(* Define the Cores' Label Coordinates *)

coreVariables[core_, rastercontrast_, sample_] :=
 Module[{core1, core2, core1Display, core2Display, probes,
   arrays, coreRaster1, coreRaster2, corexlblpos, corexlbls, coreylblpos,
   coreylbls, xliststring, xlist, ylist}, (core1 = core[[All, All, 1]];
   core2 = core[[All, All, 2]];
   {probes, arrays} = Dimensions[core1];
   core1Display = core1[[Join[Range[1, 10], Range[probes - 9, probes]],
     Join[Range[1, 5], Range[arrays - 4, arrays]]]];
   core2Display = core2[[Join[Range[1, 10], Range[probes - 9, probes]],
     Join[Range[1, 5], Range[arrays - 4, arrays]]]];
   coreRaster1 = rasterFileGeneration[core1Display, rastercontrast];
   coreRaster2 = rasterFileGeneration[core2Display, rastercontrast];
   corexlblpos = {{space + 13 + (0.325 * 2), 38 + 13}, {space + 13 + (4 * 0.65 * 2) + (0.325 * 2),
      38 + 13}, {space + 13 + 6.5 + 0.65, 38 + 13}, {space + 26 - (0.325 * 2), 38 + 13}};
   If[sample ≠ "Tumor", corexlblpos[[All, 2]] = (corexlblpos[[All, 2]] - yspace);];
   xlist = {1, 5, 244, 249};
   xliststring = {"1  ", "5  ", "245", "249"};
   corexlbls = Map[Text[Style[ToString[xliststring[[#]]], FontFamily → "Courier",
        FontSize → ticksize], {corexlblpos[[#]][[1]], corexlblpos[[#]][[2]] + 2.5},
       {0, 0}, {0, 1}] &, Range[Length[xlist]]];
   coreylblpos = {{space, 25 - 0.65}, {space, 25 - (4 * 1.3) - 0.65}, {space, 25 - 13 + 0.65},
     {space, 25 - 13 - 0.65}, {space, 25 - 13 - (5 * 1.3) - 0.65}, {space, -1 + 0.65}};
   If[sample ≠ "Tumor", coreylblpos[[All, 2]] = (coreylblpos[[All, 2]] - yspace);];
   ylist = {"1", "5", "10", "489", "494", "498"};
   coreylbls = Map[Text[Style[ylist[[#]], FontFamily → "Courier", FontSize → ticksize],
       {coreylblpos[[#]][[1]] - 1.25, coreylblpos[[#]][[2]]}, {1, 0}] &,
     Range[Length[ylist]]];
   {coreRaster1, coreRaster2, corexlbls, corexlblpos, coreylbls, coreylblpos, xlist, ylist}
  )]
```

```
(* Define the Cores Display *)

coreDisplay[coreRaster1_, coreRaster2_, corexlbls_, corexlblpos_, coreylbls_,
  coreylblpos_, xlist_, ylist_, sample_] := Module[{gcore}, (If[sample == "Tumor", (
    (* Display the Tumor Core *)
    gcore = {Graphics[Raster[coreRaster1, {{space, 12 - 13}, {space + 13, 25}},
         ColorFunction → RGBColor]], Graphics[Raster[coreRaster2,
         {{space + 13, 25}, {space + 26, 38 + 13}}, ColorFunction → RGBColor]], Graphics[Line[
         {{space, 25}, {space, 12 - 13}, {space + 13, 12 - 13}, {space + 13, 25}, {space, 25}}]],
       Graphics[Line[{{space + 13, 38 + 13}, {space + 13, 25}, {space + 26, 25},
          {space + 26, 38 + 13}, {space + 13, 38 + 13}}]],
       Graphics[Line[{{space, 25}, {space + 13, 38 + 13}}]],
       Graphics[{White, Line[{{space, 25 - 13}, {space + 13, 25 - 13}}]}],
       Graphics[{White, Line[{{space + 13, 25 + 13}, {space + 26, 25 + 13}}]}],
       Graphics[{White, Line[{{space + 6.5, 12 - 13}, {space + 6.5, 25}}]}],
       Graphics[{White, Line[{{space + 13 + 6.5, 25}, {space + 13 + 6.5, 25 + 26}}]}],
       Graphics[{White, Dashed, Line[{{space + 13, 25}, {space + 26, 38 + 13}}]}],
       Graphics[Line[{{space + 13, 12 - 13}, {space + 26, 25}}]],
       Graphics[Line[{{space, 25}, {space - 2, 25 + 2}}]],
       Graphics[Text[StyleForm["1", FontSize → 10], {space - 3, 25 + 3}, {0, 0}]],
       Graphics[Text[StyleForm["2", FontSize → 10], {space + 13 - 3, 38 + 13 + 3}, {0, 0}]],
       Graphics[Line[{{space + 13, 38 + 13}, {space + 13 - 2, 38 + 13 + 2}}]],
       Graphics[Text[Subscript[StyleForm["ℛ", FontSize → 12],
          StyleForm["tumor", FontSize → 8]], {space + 19.5, 43 + 13 + 4}, {0, 0}]],
       Graphics[Text["x-Probelets", {space + 19.5, 43 + 13.5}]],
       Graphics[Text["Arraylets", {space - 5.5, 25 - 13 + 6.5 - 2}, {0, 0}, {0, 1}]],
       Graphics[Text["y-Probelets", {space + (6.5 / 2) + (6.5 / 2) - 5, 39 + 4}, {0, 0}, {1, 2}]],
       Graphics[corexlbls], Map[Graphics[{Line[{corexlblpos[[#]], {corexlblpos[[#]][[1]],
             corexlblpos[[#]][[2]] + 0.75}}]}] &, Range[Length[xlist]]], Graphics[
        coreylbls], Map[Graphics[{Line[{coreylblpos[[#]], {coreylblpos[[#]][[1]] - 0.75,
             coreylblpos[[#]][[2]]}}]}] &, Range[Length[ylist]]]};), (
    (* Display the Normal Core *)
    gcore = {Graphics[Raster[coreRaster1, {{space, 12 - 13 - yspace}, {space + 13, 25 - yspace}},
         ColorFunction → RGBColor]], Graphics[Raster[coreRaster2,
         {{space + 13, 25 - yspace}, {space + 26, 38 + 13 - yspace}}, ColorFunction → RGBColor]],
       Graphics[Line[{{space, 25 - yspace}, {space, 12 - 13 - yspace},
          {space + 13, 12 - 13 - yspace}, {space + 13, 25 - yspace}, {space, 25 - yspace}}]],
       Graphics[Line[{{space + 13, 38 + 13 - yspace}, {space + 13, 25 - yspace}, {space + 26,
           25 - yspace}, {space + 26, 38 + 13 - yspace}, {space + 13, 38 + 13 - yspace}}]],
       Graphics[Line[{{space, 25 - yspace}, {space + 13, 38 + 13 - yspace}}]],
       Graphics[{White, Line[{{space, 25 - 13 - yspace}, {space + 13, 25 - 13 - yspace}}]}],
       Graphics[{White, Line[{{space + 13, 25 + 13 - yspace}, {space + 26, 25 + 13 - yspace}}]}],
       Graphics[{White, Line[{{space + 6.5, 12 - 13 - yspace}, {space + 6.5, 25 - yspace}}]}],
       Graphics[{White,
         Line[{{space + 13 + 6.5, 25 - yspace}, {space + 13 + 6.5, 25 + 26 - yspace}}]}], Graphics[
        {White, Dashed, Line[{{space + 13, 25 - yspace}, {space + 26, 38 + 13 - yspace}}]}],
       Graphics[Line[{{space + 13, 12 - 13 - yspace}, {space + 26, 25 - yspace}}]],
       Graphics[Line[{{space, 25 - yspace}, {space - 2, 25 + 2 - yspace}}]], Graphics[
        Text[StyleForm["1", FontSize → 10], {space - 3, 25 + 3 - yspace}, {0, 0}]], Graphics[
        Text[StyleForm["2", FontSize → 10], {space + 13 - 3, 38 + 13 + 3 - yspace}, {0, 0}]],
       Graphics[Line[{{space + 13, 38 + 13 - yspace}, {space + 13 - 2, 38 + 13 + 2 - yspace}}]],
       Graphics[Text[Subscript[StyleForm["ℛ", FontSize → 12],
          StyleForm["normal", FontSize → 8]], {space + 19.5, 43 + 13 + 4 - yspace}, {0, 0}]],
       Graphics[Text["x-Probelets", {space + 19.5, 43 + 13.5 - yspace}]], Graphics[
        Text["Arraylets", {space - 5.5, 25 - 13 + 6.5 - 2 - yspace}, {0, 0}, {0, 1}]], Graphics[
        Text["y-Probelets", {space + (6.5 / 2) + (6.5 / 2) - 5, 39 + 4 - yspace}, {0, 0}, {1, 2}]],
       Graphics[corexlbls], Map[Graphics[{Line[{corexlblpos[[#]], {corexlblpos[[#]][[1]],
             corexlblpos[[#]][[2]] + 0.75}}]}] &, Range[Length[xlist]]], Graphics[
        coreylbls], Map[Graphics[{Line[{coreylblpos[[#]], {coreylblpos[[#]][[1]] - 0.75,
             coreylblpos[[#]][[2]]}}]}] &, Range[Length[ylist]]]};)]; gcore)]
```

```
(* Define the y-Probelets' Label Coordinates *)

probeletVariables[yProbelets_, rastercontrast_, patientLabels_, sample_] :=
 Module[{xlist, probeletlbl, probeletlabel, probeletxlblpos, probeletxlbls, yProbeletRaster},
  (yProbeletRaster = Reverse[rasterFileGeneration[yProbelets, rastercontrast]];
   probeletlbl = patientLabels;
   probeletlabel = patientLabels[[All, 2]];
   probeletlabel[[All, 1]] = probeletlabel[[All, 1]] + space;
   probeletlabel[[All, 2]] = probeletlabel[[All, 2]] - 26;
   probeletlbl[[All, 2]] = probeletlabel;
   probeletxlblpos = {{space + 13, 25 - 0.65}, {space + 13, 25 - (4 * 1.3) - 0.65},
     {space + 13, 25 - (5 * 1.3) - 0.65}, {space + 13, 25 - 13 + 0.65}};
   If[sample ≠ "Tumor", probeletxlblpos[[All, 2]] = (probeletxlblpos[[All, 2]] - yspace);];
   xlist = {1, 5, 245, 249};
   probeletxlbls =
    Map[Text[Style[ToString[xlist[[#]]], FontFamily → "Courier", FontSize → ticksize],
       {probeletxlblpos[[#]][[1]] - 1.25, probeletxlblpos[[#]][[2]]},
       {1, 0}] &, Range[Length[xlist]]];
   {yProbeletRaster, probeletlbl, probeletxlblpos, probeletxlbls}
  )]

(* Define the x-Probelets Display *)

xProbeletsDisplay[probeletsRaster_, probeletlbl_, probeletxlbls_,
  probeletxlblpos_, sample_] := Module[{gxProbelets}, (If[sample == "Tumor", (
     (* Display the x-Probelets in the Tumor Factorization *)
     gxProbelets =
      {Graphics[Raster[probeletsRaster, {{space + 13, 25 - 13}, {space + 26, 38 - 13}}]],
       Graphics[{RGBColor[0, 0, 0], {Line[{{space + 13, 38 - 13}, {space + 13, 25 - 13},
             {space + 26, 25 - 13}, {space + 26, 38 - 13}, {space + 13, 38 - 13}}]}}],
       Graphics[{White, Line[{{space + 13, (25 - 13) + 6.5}, {space + 26, (25 - 13) + 6.5}}]}],
       Graphics[Line[{{space + 13, 38 - 13}, {space + 13 - 2, 38 + 2 - 13}}]],
       Graphics[Text[StyleForm["\!\(\*SubsuperscriptBox[\(V\), \(x\), \(T\)]\)",
          FontSize → 12], {space + 13 - 3, 38 + 4 - 13}, {0, 0}]],
       Graphics[Text["Arrays", {space + 19.5, 43.5 - 13}]], Graphics[probeletlbl],
       Map[Graphics[{Line[{{probeletlbl[[#, 2]][[1]], 25},
              {probeletlbl[[#, 2]][[1]], 25.75}}]}] &, Range[Length[probeletlbl]]],
       Graphics[probeletxlbls],
       Map[Graphics[{Line[{#, {#[[1]] - 0.75, #[[2]]}}]}] &, probeletxlblpos],
       Graphics[Text["x-Probelets", {space + 13 - 5.5, 25 - 13 + 6.5}, {0, 0}, {0, 1}]]};), (
     (* Display the x-Probelets in the Normal Factorization *)
     gxProbelets = {Graphics[
         Raster[probeletsRaster, {{space + 13, 25 - 13 - yspace}, {space + 26, 38 - 13 - yspace}}]],
       Graphics[{RGBColor[0, 0, 0], {Line[{{space + 13, 38 - 13 - yspace},
             {space + 13, 25 - 13 - yspace}, {space + 26, 25 - 13 - yspace},
             {space + 26, 38 - 13 - yspace}, {space + 13, 38 - 13 - yspace}}]}}], Graphics[{White,
         Line[{{space + 13, (25 - 13) + 6.5 - yspace}, {space + 26, (25 - 13) + 6.5 - yspace}}]}],
       Graphics[Line[{{space + 13, 38 - 13 - yspace}, {space + 13 - 2, 38 + 2 - 13 - yspace}}]],
       Graphics[Text[StyleForm["\!\(\*SubsuperscriptBox[\(V\), \(x\), \(T\)]\)",
          FontSize → 12], {space + 13 - 3, 38 + 4 - 13 - yspace}, {0, 0}]],
       Graphics[Text["Arrays", {space + 19.5, 43.5 - 13 - yspace}]], Graphics[probeletlbl],
       Map[Graphics[{Line[{{probeletlbl[[#, 2]][[1]], 25 - yspace},
              {probeletlbl[[#, 2]][[1]], 25.75 - yspace}}]}] &,
        Range[Length[probeletlbl]]], Graphics[probeletxlbls],
       Map[Graphics[{Line[{#, {#[[1]] - 0.75, #[[2]]}}]}] &, probeletxlblpos], Graphics[
        Text["x-Probelets", {space + 13 - 5.5, 25 - 13 + 6.5 - yspace}, {0, 0}, {0, 1}]]};)];
   gxProbelets
  )]
```

```
(* Define the y-Probelets Display *)

yProbeletsDisplay[yProbeletRaster_, sample_] := Module[{gyProbelets}, (If[sample == "Tumor", (
      (* Display the y-Probelets in the Tumor Factorization *)
      gyProbelets = {Graphics[{RGBColor[yProbeletRaster[[1, 1]]],
           Polygon[{{space + 13, 41.25 + 6.5}, {space + 13 + 6.5, 41.25 + 13},
             {space + 13 + 6.5, 41.25 + 13 + 6.5}, {space + 13, 41.25 + 13}}]}],
         Graphics[{RGBColor[yProbeletRaster[[1, 2]]], Polygon[
            {{space + 13 + 6.5, 41.25 + 13}, {space + 13 + 13, 41.25 + 13 + 6.5},
             {space + 13 + 13, 41.25 + 13 + 13}, {space + 13 + 6.5, 41.25 + 13 + 6.5}}]}],
         Graphics[{RGBColor[yProbeletRaster[[2, 1]]], Polygon[{{space + 13, 41.25},
             {space + 13 + 6.5, 41.25 + 6.5}, {space + 13 + 6.5, 41.25 + 13},
             {space + 13, 41.25 + 6.5}}]}], Graphics[{RGBColor[yProbeletRaster[[2, 2]]],
           Polygon[{{space + 13 + 6.5, 41.25 + 6.5}, {space + 13 + 13, 41.25 + 13},
             {space + 13 + 13, 41.25 + 13 + 6.5}, {space + 13 + 6.5, 41.25 + 13}}]}],
         Graphics[Line[{{space + 13, 41.25 + 13}, {space + 13 - 2, 41.25 + 13 + 2}}]],
         Graphics[{EdgeForm[{RGBColor[0, 0, 0]}], FaceForm[], Polygon[{{space + 13, 41.25},
             {space + 26, 41.25 + 13}, {space + 26, 41.25 + 26}, {space + 13, 41.25 + 13}}]}],
         Graphics[Text[StyleForm["\!\(\*SubsuperscriptBox[\(V\), \(y\), \(T\)]\)",
            FontSize → 12], {space + 13 - 3, 41.25 + 13 + 4}, {0, 0}]], Graphics[
          Text["Platforms", {space + 13 + 6.5 / 2, 41.25 + 1.5 + 26 - 6.5 / 2}, {0, 0}, {1, 1}]],
         Graphics[Text["y-Probelets", {space + 13 - 5.5, 25 - 4.25 + 26}, {0, 0}, {0, 1}]]};), (
      (* Display the y-Probelets in the Normal Factorization *)
      gyProbelets = {Graphics[{RGBColor[yProbeletRaster[[1, 1]]],
           Polygon[{{space + 13, 41.25 + 6.5 - yspace}, {space + 13 + 6.5, 41.25 + 13 - yspace},
             {space + 13 + 6.5, 41.25 + 13 + 6.5 - yspace}, {space + 13, 41.25 + 13 - yspace}}]}],
         Graphics[{RGBColor[yProbeletRaster[[1, 2]]], Polygon[{{space + 13 + 6.5,
              41.25 + 13 - yspace}, {space + 13 + 13, 41.25 + 13 + 6.5 - yspace}, {space + 13 + 13,
              41.25 + 13 + 13 - yspace}, {space + 13 + 6.5, 41.25 + 13 + 6.5 - yspace}}]}],
         Graphics[{RGBColor[yProbeletRaster[[2, 1]]], Polygon[
            {{space + 13, 41.25 - yspace}, {space + 13 + 6.5, 41.25 + 6.5 - yspace},
             {space + 13 + 6.5, 41.25 + 13 - yspace}, {space + 13, 41.25 + 6.5 - yspace}}]}],
         Graphics[{RGBColor[yProbeletRaster[[2, 2]]], Polygon[{{space + 13 + 6.5,
              41.25 + 6.5 - yspace}, {space + 13 + 13, 41.25 + 13 - yspace}, {space + 13 + 13,
              41.25 + 13 + 6.5 - yspace}, {space + 13 + 6.5, 41.25 + 13 - yspace}}]}], Graphics[
          Line[{{space + 13, 41.25 + 13 - yspace}, {space + 13 - 2, 41.25 + 13 + 2 - yspace}}]],
         Graphics[{EdgeForm[{RGBColor[0, 0, 0]}], FaceForm[],
           Polygon[{{space + 13, 41.25 - yspace}, {space + 26, 41.25 + 13 - yspace},
             {space + 26, 41.25 + 26 - yspace}, {space + 13, 41.25 + 13 - yspace}}]}],
         Graphics[Text[StyleForm["\!\(\*SubsuperscriptBox[\(V\), \(y\), \(T\)]\)",
            FontSize → 12], {space + 13 - 3, 41.25 + 13 + 4 - yspace}, {0, 0}]],
         Graphics[Text["Platforms", {space + 13 + 6.5 / 2, 41.25 + 1.5 + 26 - 6.5 / 2 - yspace},
           {0, 0}, {1, 1}]], Graphics[Text["y-Probelets",
           {space + 13 - 5.5, 25 - 4.25 + 26 - yspace}, {0, 0}, {0, 1}]]};)];
   gyProbelets
  )]

(* Define the Equal Sign Display *)

gEqualDisplay[sample_] := Module[{gEqual}, (If[sample == "Tumor",
     (gEqual = Graphics[Text[StyleForm["≃", FontSize → 30], {32.5, 25 - 13 + 6.5 - 2}]];),
     (gEqual =
        Graphics[Text[StyleForm["≃", FontSize → 30], {30.5, 25 - 13 + 6.5 - 2 - yspace}]];)];
   gEqual
  )]
```

```
(* Define the Angular Distances Bar Chart *)

angularDistance[tumorCore_, normalCore_] := Module[
  {tumorCore1, tumorCore2, normalCore1, normalCore2, arrays, a, b, distances, ylabels, framex,
   gridx, framey, gridy, labelx, labely, barsFull, barsInset}, (a = tumorCore / normalCore;
   arrays = 249 * 2;
   distances = Table[N[ArcTan[a[[b, 1]]] - Pi / 4], {b, 1, arrays}][[All, 1]];
   ylabels = {1, 50, 100, 150, 200, 250, 300, 350, 400, 450};
   gridx = {-π / 4, -π / 8, 0, π / 8, π / 4};
   framex = {"-π/4", "-π/8", "0", "π/8", "π/4"};
   framex = Table[{gridx[[a]], Style[Rotate[framex[[a]], Pi / 2]]}, {a, 1, Length[framex]}];
   gridx = Table[{gridx[[a]], RGBColor[0, 0, 0]}, {a, 1, 5}];
   framey = Table[{arrays - ylabels[[a]], ylabels[[a]]}, {a, 1, Dimensions[ylabels][[1]]}];
   labelx = Style["Angular Distance", FontSize → 10];
   labely = Style["Arraylets", FontSize → 10];
   barsFull = Show[BarChart[Reverse[distances], BarOrigin → Left, ChartStyle → Red,
       ChartBaseStyle → EdgeForm[None], Axes → False, PlotRange → {{-0.8, 0.8}, Automatic},
       AspectRatio → 1, ImageSize → 300], Frame → True, FrameTicks → {None, framey, framex, None},
     FrameLabel → {None, labely, labelx, None}, GridLines → {{distances[[2]]}, None},
     GridLinesStyle → Directive[Dashed], BaseStyle → {FontFamily → "Courier", FontSize → 8}];
   barsInset = Graphics[Rectangle[{82 - 6.5, -65 - yspace},
       {space + 26, -65 - yspace + 50.25 + 7}, barsFull]];
   barsInset
  )]
```

```
(* Compute the 6p+12p Tensor GSVD *)
```

```
(* Read the 6p+12p Datasets *)

tumor1 = Import[path <> "D_Tumor_1_6p+12p.txt", "Table"];
tumor2 = Import[path <> "D_Tumor_2_6p+12p.txt", "Table"];
normal1 = Import[path <> "D_Normal_1_6p+12p.txt", "Table"];
normal2 = Import[path <> "D_Normal_2_6p+12p.txt", "Table"];
arrayNames = tumor1[[1, 5 ;;]];
tumorProbeID = tumor1[[2 ;;, 1]];
tumorProbeLoc = tumor1[[2 ;;, 2 ;; 4]];
normalProbeID = normal1[[2 ;;, 1]];
normalProbeLoc = normal1[[2 ;;, 2 ;; 4]];
tumor1 = Drop[tumor1, 1, 4];
tumor2 = Drop[tumor2, 1, 4];
normal1 = Drop[normal1, 1, 4];
normal2 = Drop[normal2, 1, 4];

(* Compute the 6p+12p Tensor GSVD *)

{tumor1, tumor2, normal1, normal2, zTumorArraylets, zNormalArraylets, tumorCore,
   normalCore, xProbelets, yProbelets} = tGSVD[tumor1, tumor2, normal1, normal2];

(* Create the Rasters of the 6p+12p Tumor Datasets *)

labelsize = 12;
yspace = 115;
ticksize = 8;
rastercontrast = 1;
step = 5;

tumor1Raster = rasterFileGeneration[tumor1, rastercontrast];
tumor2Raster = rasterFileGeneration[tumor2, rastercontrast];
probes = Dimensions[tumor1Raster][[1]];
```

```
(* Input the 6p+12p Chromosome Band Coordinates *)

arm = Transpose[Join[Transpose[tumorProbeLoc], {zTumorArraylets[[All, 1]]}]];
armBands = {{"#chrom", "chromStart", "chromEnd", "name"}, {"chr6", 0, 2300000, "p25.3"},
   {"chr6", 2300000, 4200000, "p25.2"}, {"chr6", 4200000, 7100000, "p25.1"},
   {"chr6", 7100000, 10600000, "p24.3"}, {"chr6", 10600000, 11600000, "p24.2"},
   {"chr6", 11600000, 13400000, "p24.1"}, {"chr6", 13400000, 15200000, "p23"},
   {"chr6", 15200000, 25200000, "p22.3"}, {"chr6", 25200000, 27000000, "p22.2"},
   {"chr6", 27000000, 30400000, "p22.1"}, {"chr6", 30400000, 32100000, "p21.33"},
   {"chr6", 32100000, 33500000, "p21.32"}, {"chr6", 33500000, 36600000, "p21.31"},
   {"chr6", 36600000, 40500000, "p21.2"}, {"chr6", 40500000, 46200000, "p21.1"},
   {"chr6", 46200000, 51800000, "p12.3"}, {"chr6", 51800000, 52900000, "p12.2"},
   {"chr6", 52900000, 57000000, "p12.1"}, {"chr6", 57000000, 58700000, "p11.2"},
   {"chr12", 0, 3300000, "p13.33"}, {"chr12", 3300000, 5400000, "p13.32"},
   {"chr12", 5400000, 10100000, "p13.31"}, {"chr12", 10100000, 12800000, "p13.2"},
   {"chr12", 12800000, 14800000, "p13.1"}, {"chr12", 14800000, 20000000, "p12.3"},
   {"chr12", 20000000, 21300000, "p12.2"}, {"chr12", 21300000, 26500000, "p12.1"},
   {"chr12", 26500000, 27800000, "p11.23"}, {"chr12", 27800000, 30700000, "p11.22"},
   {"chr12", 30700000, 33300000, "p11.21"}, {"chr12", 33300000, 35800000, "p11.1"}};

{bandLabels, patientLabels, xticks, yPosition, z} =
  figureVariables[armBands, arm, probes, "Tumor", "6p+12p"];

Map[(bandLabels[[#[[1]]]] =
      Text[Style[ToString[#[[1]]], FontFamily → "Courier", FontSize → ticksize],
       {-1.25, #[[2]]}, {1, 0}];) &,
  {{5, z[[5]] + 0.25}, {6, z[[6]] - 0.25}, {7, z[[7]] + 0.25}, {8, z[[8]] - 0.25}}];
bandLabels[[20]] = Text[Style[ToString[1], FontFamily → "Courier", FontSize → ticksize],
   {-1.25, z[[20]] - 0.5}, {1, 0}];

(* Display the 6p+12p Tumor Datasets *)

tumor1Raster = Graphics[Raster[tumor1Raster, {{0, -50}, {13, 25}}, ColorFunction → RGBColor]];
tumor2Raster =
  Graphics[Raster[tumor2Raster, {{13, -37 + 13}, {26, 38 + 13}}, ColorFunction → RGBColor]];

gTumorTensor = tensorDisplay[tumor1Raster,
   tumor2Raster, bandLabels, yPosition, xticks, patientLabels, "Tumor"];

(* Display the 6p+12p Tumor Arraylets *)

space = 43.5;
rastercontrast = 50;
step = 5;
{rasterArraylets, bandLabelsArraylets, arrayletylbls, arrayletylblpos} =
  arrayletVariables[zTumorArraylets, bandLabels, "Tumor"];

gzTumorArraylets = arrayletsDisplay[rasterArraylets,
   bandLabelsArraylets, arrayletylbls, arrayletylblpos, xticks, yPosition, "Tumor"];

(* Display the 6p+12p Tumor Core *)

rastercontrast = 0.01;
step = 1;
space = 82;
{coreRaster1, coreRaster2, corexlbls, corexlblpos, coreylbls, coreylblpos, xlist, ylist} =
  coreVariables[tumorCore, rastercontrast, "Tumor"];

gTumorCore = coreDisplay[coreRaster1, coreRaster2,
   corexlbls, corexlblpos, coreylbls, coreylblpos, xlist, ylist, "Tumor"];
```

```
(* Display the 6p+12p x- and y-Probelets *)

rastercontrast = 30;
step = 1;
{rows, columns} = Dimensions[xProbelets];
probeletsRaster = rasterFileGeneration[
   xProbelets[[Join[Range[1, 5], Range[rows - 4, rows]], All]], rastercontrast];

rastercontrast = 1;
step = 1;
space = 117 - 10.75;
{yProbeletRaster, probeletlbl, probeletxlblpos, probeletxlbls} =
  probeletVariables[yProbelets, rastercontrast, patientLabels, "Tumor"];

gxTumorProbelets =
  xProbeletsDisplay[probeletsRaster, probeletlbl, probeletxlbls, probeletxlblpos, "Tumor"];

gyTumorProbelets = yProbeletsDisplay[yProbeletRaster, "Tumor"];
gTumorEqual = gEqualDisplay["Tumor"];

(* Create Rasters of the 6p+12p Normal Datasets *)

rastercontrast = 1;
step = 5;

normal1Raster = rasterFileGeneration[normal1, rastercontrast];
normal2Raster = rasterFileGeneration[normal2, rastercontrast];
probes = Dimensions[normal1Raster][[1]];

(* Input the 6p+12p Chromosome Band Coordinates *)

arm = Transpose[Join[Transpose[normalProbeLoc], {zNormalArraylets[[All, 1]]}]];

{bandLabels, patientLabels, xticks, yPosition, z} =
  figureVariables[armBands, arm, probes, "Normal", "6p+12p"];

Map[(bandLabels[[#[[1]]]] =
      Text[Style[ToString[#[[1]]], FontFamily → "Courier", FontSize → ticksize],
       {-1.25, #[[2]]}, {1, 0}];) &,
  {{5, z[[5]] + 0.25}, {6, z[[6]] - 0.25}, {7, z[[7]] + 0.25}, {8, z[[8]] - 0.25}}];
bandLabels[[20]] = Text[Style[ToString[1], FontFamily → "Courier", FontSize → ticksize],
   {-1.25, z[[20]] - 0.5}, {1, 0}];

(* Display the 6p+12p Normal Datasets *)

normal1Raster = Graphics[
   Raster[normal1Raster, {{0, -65 - yspace}, {13, 25 - yspace}}, ColorFunction → RGBColor]];
normal2Raster = Graphics[Raster[normal2Raster,
     {{13, -52 + 13 - yspace}, {26, 38 + 13 - yspace}}, ColorFunction → RGBColor]];

gNormalTensor = tensorDisplay[normal1Raster,
   normal2Raster, bandLabels, yPosition, xticks, patientLabels, "Normal"];
```

```
(* Display the 6p+12p Normal Arraylets *)

space = 43.5;
rastercontrast = 50;
step = 5;
{rasterArraylets, bandLabelsArraylets, arrayletylbls, arrayletylblpos} =
  arrayletVariables[zNormalArraylets, bandLabels, "Normal"];

gzNormalArraylets = arrayletsDisplay[rasterArraylets,
   bandLabelsArraylets, arrayletylbls, arrayletylblpos, xticks, yPosition, "Normal"];

(* Display the 6p+12p Normal Core *)

rastercontrast = 0.01;
step = 1;
space = 82;
{coreRaster1, coreRaster2, corexlbls, corexlblpos, coreylbls, coreylblpos, xlist, ylist} =
  coreVariables[normalCore, rastercontrast, "Normal"];

gNormalCore = coreDisplay[coreRaster1, coreRaster2,
   corexlbls, corexlblpos, coreylbls, coreylblpos, xlist, ylist, "Normal"];

(* Display the 6p+12p x- and y-Probelets *)

rastercontrast = 30;
step = 1;
{rows, columns} = Dimensions[xProbelets];
probeletsRaster = rasterFileGeneration[
   xProbelets[[Join[Range[1, 5], Range[rows - 4, rows]], All]], rastercontrast];

rastercontrast = 1;
step = 1;
space = 117 - 10.75;
{yProbeletRaster, probeletlbl, probeletxlblpos, probeletxlbls} =
  probeletVariables[yProbelets, rastercontrast, patientLabels, "Normal"];

gxNormalProbelets =
  xProbeletsDisplay[probeletsRaster, probeletlbl, probeletxlbls, probeletxlblpos, "Normal"];

gyNormalProbelets = yProbeletsDisplay[yProbeletRaster, "Normal"];
gNormalEqual = gEqualDisplay["Normal"];

(* Display the 6p+12p Angular Distances Bar Chart *)

barsInset = angularDistance[tumorCore, normalCore];

(* Display the 6p+12p Tensor GSVD *)

fig1 = Show[{gTumorTensor, gTumorEqual, gzTumorArraylets,
   gTumorCore, gxTumorProbelets, gyTumorProbelets, gNormalTensor, gNormalEqual,
   gzNormalArraylets, gNormalCore, gxNormalProbelets, gyNormalProbelets, barsInset},
  PlotRange → All, ImageSize → 750, AspectRatio → Automatic]
```

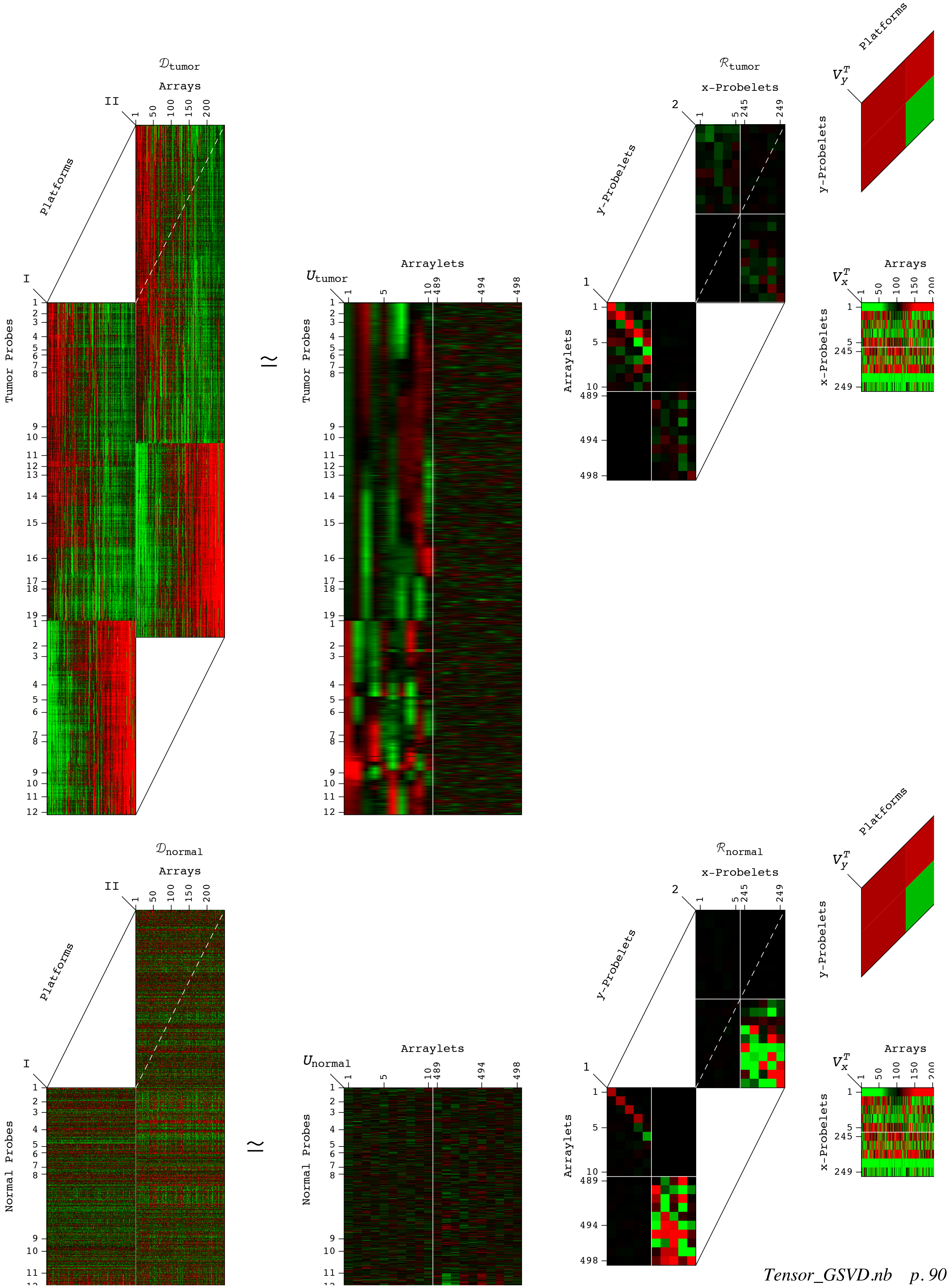

$\mathcal{D}_{tumor}$
Arrays
II
1 50 100 150 200
Platforms
I
Tumor Probes
1 2 3 4 5 6 7 8 9 10 11 12 13 14 15 16 17 18 19
1 2 3 4 5 6 7 8 9 10 11 12
≃
U_{tumor}
Arraylets
1 5 10 489 494 498
Tumor Probes
$\mathcal{R}_{tumor}$
x-Probelets
2
1 5 245 249
y-Probelets
1
Arraylets
1 5 10 489 494 498
V_y^T
Platforms
y-Probelets
V_x^T
Arrays
1 50 100 150 200
x-Probelets
1 5 245 249
$\mathcal{D}_{normal}$
Arrays
II
1 50 100 150 200
Platforms
I
Normal Probes
1 2 3 4 5 6 7 8 9 10 11
≃
U_{normal}
Arraylets
1 5 10 489 494 498
Normal Probes
$\mathcal{R}_{normal}$
x-Probelets
2
1 5 245 249
y-Probelets
1
Arraylets
1 5 10 489 494 498
V_y^T
Platforms
y-Probelets
V_x^T
Arrays
1 50 100 150 200
x-Probelets
1 5 245 249

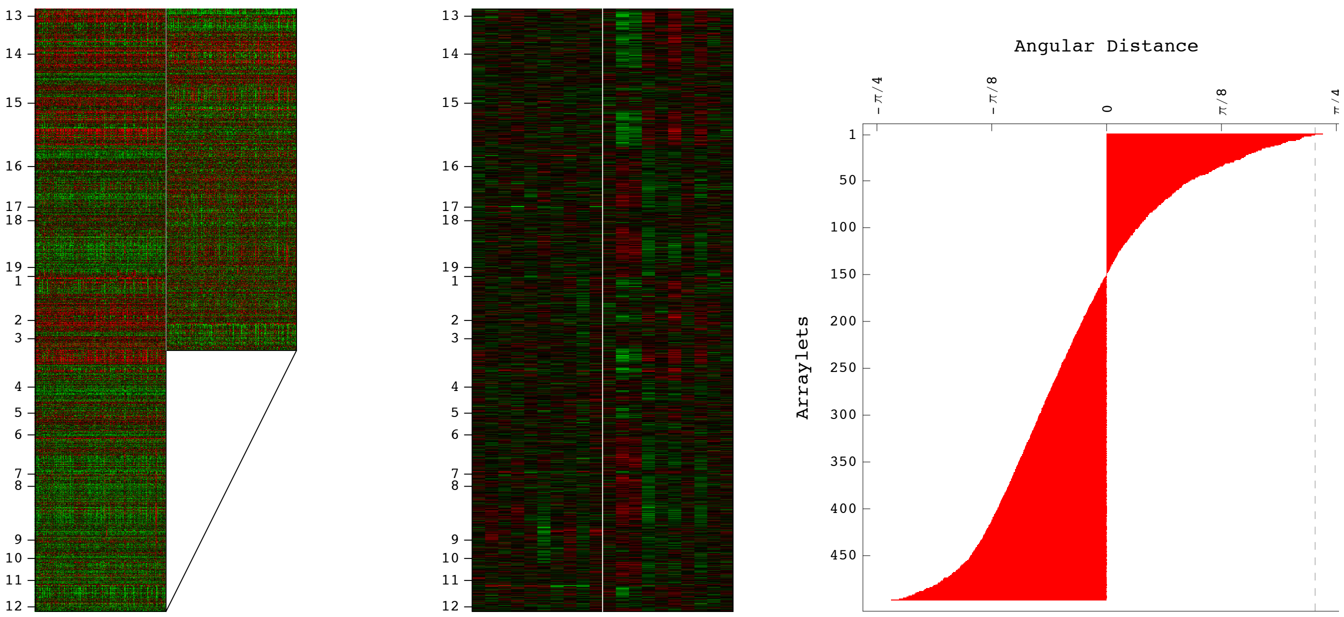


```
Export[path <> "Figure_1.pdf", fig1, "PDF", ImageSize → 750];
```

```
(* Compute the 7p Tensor GSVD *)
```

```
(* Read the 7p Datasets *)

tumor1 = Import[path <> "D_Tumor_1_7p.txt", "Table"];
tumor2 = Import[path <> "D_Tumor_2_7p.txt", "Table"];
normal1 = Import[path <> "D_Normal_1_7p.txt", "Table"];
normal2 = Import[path <> "D_Normal_2_7p.txt", "Table"];
arrayNames = tumor1[[1, 5 ;;]];
tumorProbeID = tumor1[[2 ;;, 1]];
tumorProbeLoc = tumor1[[2 ;;, 2 ;; 4]];
normalProbeID = normal1[[2 ;;, 1]];
normalProbeLoc = normal1[[2 ;;, 2 ;; 4]];
tumor1 = Drop[tumor1, 1, 4];
tumor2 = Drop[tumor2, 1, 4];
normal1 = Drop[normal1, 1, 4];
normal2 = Drop[normal2, 1, 4];

(* Compute the 7p Tensor GSVD *)

{tumor1, tumor2, normal1, normal2, zTumorArraylets, zNormalArraylets, tumorCore,
   normalCore, xProbelets, yProbelets} = tGSVD[tumor1, tumor2, normal1, normal2];

(* Create Rasters of the 7p Tumor Datasets *)

labelsize = 12;
yspace = 115;
ticksize = 8;
rastercontrast = 1;
step = 5;

tumor1Raster = rasterFileGeneration[tumor1, rastercontrast];
tumor2Raster = rasterFileGeneration[tumor2, rastercontrast];
probes = Dimensions[tumor1Raster][[1]];
```

```
(* Input the 7p Chromosome Band Coordinates *)

arm = Transpose[Join[Transpose[tumorProbeLoc], {zTumorArraylets[[All, 1]]}]];
armBands = {{"#chrom", "chromStart", "chromEnd", "name"}, {"chr7", 0, 2800000, "p22.3"},
   {"chr7", 2800000, 4500000, "p22.2"}, {"chr7", 4500000, 7300000, "p22.1"},
   {"chr7", 7300000, 13800000, "p21.3"}, {"chr7", 13800000, 16500000, "p21.2"},
   {"chr7", 16500000, 20900000, "p21.1"}, {"chr7", 20900000, 25500000, "p15.3"},
   {"chr7", 25500000, 28000000, "p15.2"}, {"chr7", 28000000, 28800000, "p15.1"},
   {"chr7", 28800000, 35000000, "p14.3"}, {"chr7", 35000000, 37200000, "p14.2"},
   {"chr7", 37200000, 43300000, "p14.1"}, {"chr7", 43300000, 45400000, "p13"},
   {"chr7", 45400000, 49000000, "p12.3"}, {"chr7", 49000000, 50500000, "p12.2"},
   {"chr7", 50500000, 54000000, "p12.1"}, {"chr7", 54000000, 58000000, "p11.2"}};

{bandLabels, patientLabels, xticks, yPosition, z} =
  figureVariables[armBands, arm, probes, "Tumor"];

(* Display the 7p Tumor Datasets *)

tumor1Raster = Graphics[Raster[tumor1Raster, {{0, -50}, {13, 25}}, ColorFunction → RGBColor]];
tumor2Raster =
  Graphics[Raster[tumor2Raster, {{13, -37 + 13}, {26, 38 + 13}}, ColorFunction → RGBColor]];

gTumorTensor = tensorDisplay[tumor1Raster,
   tumor2Raster, bandLabels, yPosition, xticks, patientLabels, "Tumor"];

(* Display the 7p Tumor Arraylets *)

space = 43.5;
rastercontrast = 50;
step = 5;
{rasterArraylets, bandLabelsArraylets, arrayletylbls, arrayletylblpos} =
  arrayletVariables[zTumorArraylets, bandLabels, "Tumor"];

gzTumorArraylets = arrayletsDisplay[rasterArraylets,
   bandLabelsArraylets, arrayletylbls, arrayletylblpos, xticks, yPosition, "Tumor"];

(* Display the 7p Tumor Core *)

rastercontrast = 0.01;
step = 1;
space = 82;
{coreRaster1, coreRaster2, corexlbls, corexlblpos, coreylbls, coreylblpos, xlist, ylist} =
  coreVariables[tumorCore, rastercontrast, "Tumor"];

gTumorCore = coreDisplay[coreRaster1, coreRaster2,
   corexlbls, corexlblpos, coreylbls, coreylblpos, xlist, ylist, "Tumor"];
```

```
(* Display the 7p x- and y-Probelets *)

rastercontrast = 30;
step = 1;
{rows, columns} = Dimensions[xProbelets];
probeletsRaster = rasterFileGeneration[
   xProbelets[[Join[Range[1, 5], Range[rows - 4, rows]], All]], rastercontrast];

rastercontrast = 1;
step = 1;
space = 117 - 10.75;
{yProbeletRaster, probeletlbl, probeletxlblpos, probeletxlbls} =
  probeletVariables[yProbelets, rastercontrast, patientLabels, "Tumor"];

gxTumorProbelets =
  xProbeletsDisplay[probeletsRaster, probeletlbl, probeletxlbls, probeletxlblpos, "Tumor"];

gyTumorProbelets = yProbeletsDisplay[yProbeletRaster, "Tumor"];
gTumorEqual = gEqualDisplay["Tumor"];

(* Create Rasters of the 7p Normal Datasets *)

rastercontrast = 1;
step = 5;

normal1Raster = rasterFileGeneration[normal1, rastercontrast];
normal2Raster = rasterFileGeneration[normal2, rastercontrast];
probes = Dimensions[normal1Raster][[1]];

(* Input the 7p Chromosome Band Coordinates *)

arm = Transpose[Join[Transpose[normalProbeLoc], {zNormalArraylets[[All, 1]]}]];

{bandLabels, patientLabels, xticks, yPosition, z} =
  figureVariables[armBands, arm, probes, "Normal"];

(* Display the 7p Normal Datasets *)

normal1Raster = Graphics[
   Raster[normal1Raster, {{0, -65 - yspace}, {13, 25 - yspace}}, ColorFunction → RGBColor]];
normal2Raster = Graphics[Raster[normal2Raster,
     {{13, -52 + 13 - yspace}, {26, 38 + 13 - yspace}}, ColorFunction → RGBColor]];

gNormalTensor = tensorDisplay[normal1Raster,
   normal2Raster, bandLabels, yPosition, xticks, patientLabels, "Normal"];
```

```
(* Display the 7p Normal Arraylets *)

space = 43.5;
rastercontrast = 50;
step = 5;
{rasterArraylets, bandLabelsArraylets, arrayletylbls, arrayletylblpos} =
  arrayletVariables[zNormalArraylets, bandLabels, "Normal"];

gzNormalArraylets = arrayletsDisplay[rasterArraylets,
   bandLabelsArraylets, arrayletylbls, arrayletylblpos, xticks, yPosition, "Normal"];

(* Display the 7p Normal Core *)

rastercontrast = 0.01;
step = 1;
space = 82;
{coreRaster1, coreRaster2, corexlbls, corexlblpos, coreylbls, coreylblpos, xlist, ylist} =
  coreVariables[normalCore, rastercontrast, "Normal"];

gNormalCore = coreDisplay[coreRaster1, coreRaster2,
   corexlbls, corexlblpos, coreylbls, coreylblpos, xlist, ylist, "Normal"];

(* Display the 7p x- and y-Probelets *)

rastercontrast = 30;
step = 1;
{rows, columns} = Dimensions[xProbelets];
probeletsRaster = rasterFileGeneration[
   xProbelets[[Join[Range[1, 5], Range[rows - 4, rows]], All]], rastercontrast];

rastercontrast = 1;
step = 1;
space = 117 - 10.75;
{yProbeletRaster, probeletlbl, probeletxlblpos, probeletxlbls} =
  probeletVariables[yProbelets, rastercontrast, patientLabels, "Normal"];

gxNormalProbelets =
  xProbeletsDisplay[probeletsRaster, probeletlbl, probeletxlbls, probeletxlblpos, "Normal"];

gyNormalProbelets = yProbeletsDisplay[yProbeletRaster, "Normal"];
gNormalEqual = gEqualDisplay["Normal"];

(* Display the 7p Angular Distances Bar Chart *)

barsInset = angularDistance[tumorCore, normalCore];

(* Display the 7p Tensor GSVD *)

figA = Show[{gTumorTensor, gTumorEqual, gzTumorArraylets,
   gTumorCore, gxTumorProbelets, gyTumorProbelets, gNormalTensor, gNormalEqual,
   gzNormalArraylets, gNormalCore, gxNormalProbelets, gyNormalProbelets, barsInset},
  PlotRange → All, ImageSize → 750, AspectRatio → Automatic]
```

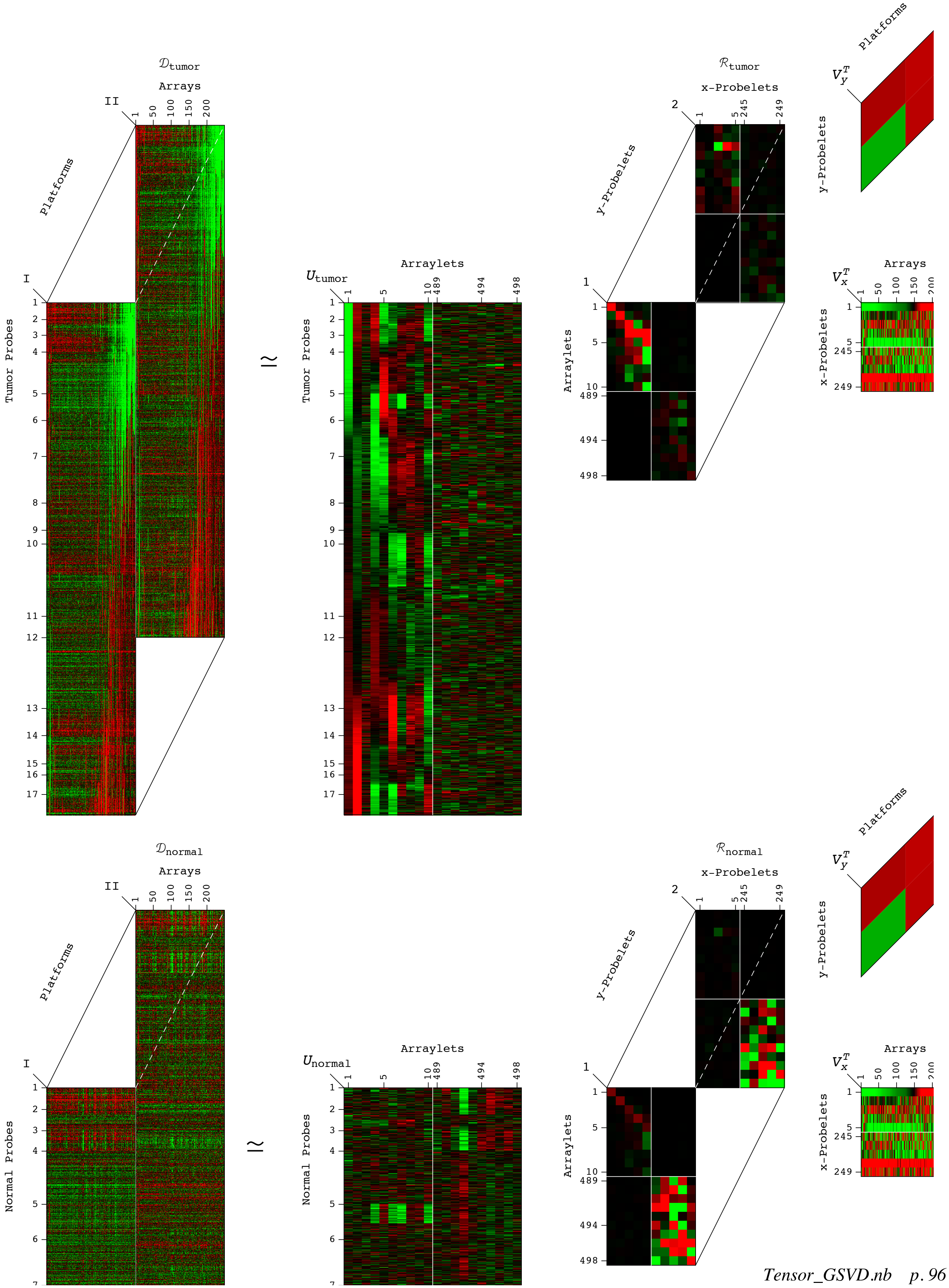
$\mathcal{D}_{\text{tumor}}$
Arrays
Platforms
I
II
Tumor Probes
U_{tumor}
Arraylets
$\mathcal{R}_{\text{tumor}}$
x-Probelets
y-Probelets
Arraylets
V_y^T
V_x^T
$\mathcal{D}_{\text{normal}}$
Normal Probes
U_{normal}
$\mathcal{R}_{\text{normal}}$

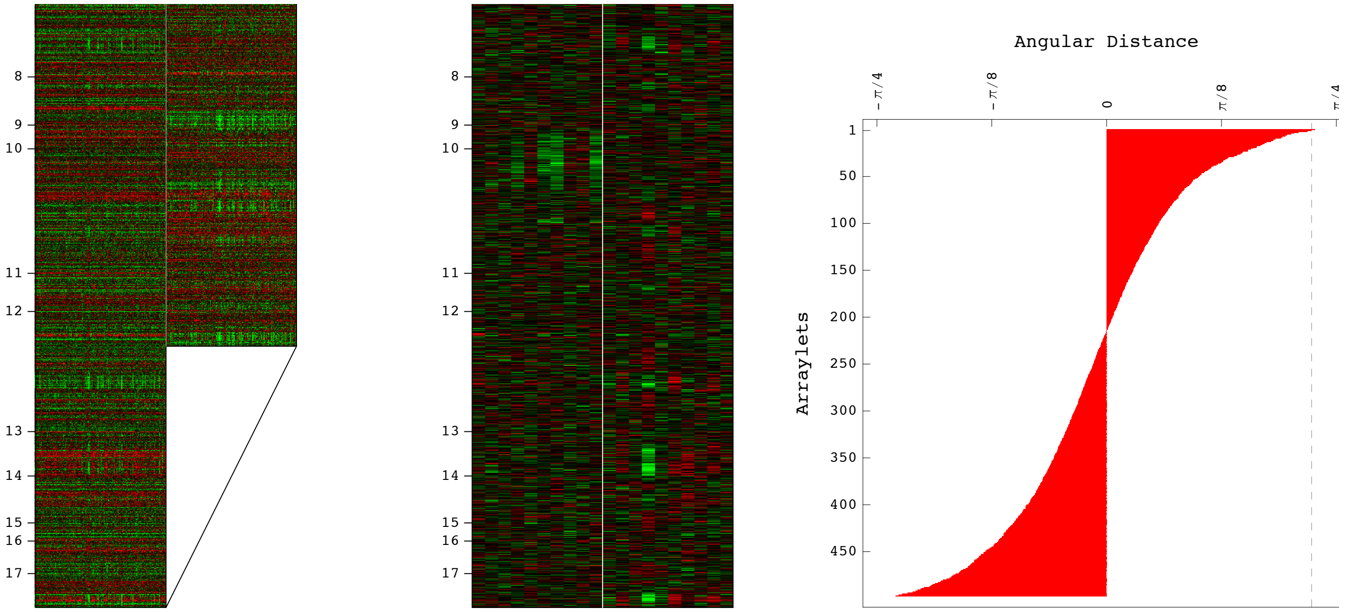


```
Export[path <> "Figure_A.pdf", figA, "PDF", ImageSize → 750];
```

```
(* Compute Xq Tensor GSVD *)

(* Read Xq Datasets *)

tumor1 = Import[path <> "D_Tumor_1_Xq.txt", "Table"];
tumor2 = Import[path <> "D_Tumor_2_Xq.txt", "Table"];
normal1 = Import[path <> "D_Normal_1_Xq.txt", "Table"];
normal2 = Import[path <> "D_Normal_2_Xq.txt", "Table"];
arrayNames = tumor1[[1, 5 ;;]];
tumorProbeID = tumor1[[2 ;;, 1]];
tumorProbeLoc = tumor1[[2 ;;, 2 ;; 4]];
normalProbeID = normal1[[2 ;;, 1]];
normalProbeLoc = normal1[[2 ;;, 2 ;; 4]];
tumor1 = Drop[tumor1, 1, 4];
tumor2 = Drop[tumor2, 1, 4];
normal1 = Drop[normal1, 1, 4];
normal2 = Drop[normal2, 1, 4];

(* Compute the Xq Tensor GSVD *)

{tumor1, tumor2, normal1, normal2, zTumorArraylets, zNormalArraylets, tumorCore,
   normalCore, xProbelets, yProbelets} = tGSVD[tumor1, tumor2, normal1, normal2];

(* Create Rasters of the Xq Tumor Datasets *)

labelsize = 12;
yspace = 115;
ticksize = 8;
rastercontrast = 1;
step = 5;

tumor1Raster = rasterFileGeneration[tumor1, rastercontrast];
tumor2Raster = rasterFileGeneration[tumor2, rastercontrast];
probes = Dimensions[tumor1Raster][[1]];
```

```
(* Input the Xq Chromosome Band Coordinates *)

arm = Transpose[Join[Transpose[tumorProbeLoc], {zTumorArraylets[[All, 1]]}]];
armBands = {{"#chrom", "chromStart", "chromEnd", "name"},
   {"chrX", 60 600 000, 63 000 000, "q11.1"}, {"chrX", 63 000 000, 64 600 000, "q11.2"},
   {"chrX", 64 600 000, 67 800 000, "q12"}, {"chrX", 67 800 000, 71 800 000, "q13.1"},
   {"chrX", 71 800 000, 73 900 000, "q13.2"}, {"chrX", 73 900 000, 76 000 000, "q13.3"},
   {"chrX", 76 000 000, 84 600 000, "q21.1"}, {"chrX", 84 600 000, 86 200 000, "q21.2"},
   {"chrX", 86 200 000, 91 800 000, "q21.31"}, {"chrX", 91 800 000, 93 500 000, "q21.32"},
   {"chrX", 93 500 000, 98 300 000, "q21.33"}, {"chrX", 98 300 000, 102 600 000, "q22.1"},
   {"chrX", 102 600 000, 103 700 000, "q22.2"}, {"chrX", 103 700 000, 108 700 000, "q22.3"},
   {"chrX", 108 700 000, 116 500 000, "q23"}, {"chrX", 116 500 000, 120 900 000, "q24"},
   {"chrX", 120 900 000, 128 700 000, "q25"}, {"chrX", 128 700 000, 130 400 000, "q26.1"},
   {"chrX", 130 400 000, 133 600 000, "q26.2"}, {"chrX", 133 600 000, 138 000 000, "q26.3"},
   {"chrX", 138 000 000, 140 300 000, "q27.1"}, {"chrX", 140 300 000, 142 100 000, "q27.2"},
   {"chrX", 142 100 000, 147 100 000, "q27.3"}, {"chrX", 147 100 000, 155 270 560, "q28"}};

{bandLabels, patientLabels, xticks, yPosition, z} =
  figureVariables[armBands, arm, probes, "Tumor"];

Map[(bandLabels[[#[[1]]]] =
     Text[Style[ToString[#[[1]]], FontFamily → "Courier", FontSize → ticksize],
      {-1.25, #[[2]]}, {1, 0}];) &, {{1, 25.4}, {2, 24.3}, {3, 23.05},
   {10, z[[10]] + 0.1}, {11, z[[11]] - 0.95}, {22, z[[22]] + 0.2}, {23, z[[23]] - 0.2}}];

(* Display the Xq Tumor Datasets *)

tumor1Raster = Graphics[Raster[tumor1Raster, {{0, -50}, {13, 25}}, ColorFunction → RGBColor]];
tumor2Raster =
  Graphics[Raster[tumor2Raster, {{13, -37 + 13}, {26, 38 + 13}}, ColorFunction → RGBColor]];

gTumorTensor = tensorDisplay[tumor1Raster,
   tumor2Raster, bandLabels, yPosition, xticks, patientLabels, "Tumor"];

(* Display the Xq Tumor Arraylets *)

space = 43.5;
rastercontrast = 50;
step = 5;
{rasterArraylets, bandLabelsArraylets, arrayletylbls, arrayletylblpos} =
  arrayletVariables[zTumorArraylets, bandLabels, "Tumor"];

gzTumorArraylets = arrayletsDisplay[rasterArraylets,
   bandLabelsArraylets, arrayletylbls, arrayletylblpos, xticks, yPosition, "Tumor"];

(* Display the Xq Tumor Core *)

rastercontrast = 0.01;
step = 1;
space = 82;
{coreRaster1, coreRaster2, corexlbls, corexlblpos, coreylbls, coreylblpos, xlist, ylist} =
  coreVariables[tumorCore, rastercontrast, "Tumor"];

gTumorCore = coreDisplay[coreRaster1, coreRaster2,
   corexlbls, corexlblpos, coreylbls, coreylblpos, xlist, ylist, "Tumor"];
```

```
(* Display the Xq x- and y-Probelets *)

rastercontrast = 30;
step = 1;
{rows, columns} = Dimensions[xProbelets];
probeletsRaster = rasterFileGeneration[
   xProbelets[[Join[Range[1, 5], Range[rows - 4, rows]], All]], rastercontrast];

rastercontrast = 1;
step = 1;
space = 117 - 10.75;
{yProbeletRaster, probeletlbl, probeletxlblpos, probeletxlbls} =
  probeletVariables[yProbelets, rastercontrast, patientLabels, "Tumor"];

gxTumorProbelets =
  xProbeletsDisplay[probeletsRaster, probeletlbl, probeletxlbls, probeletxlblpos, "Tumor"];

gyTumorProbelets = yProbeletsDisplay[yProbeletRaster, "Tumor"];
gTumorEqual = gEqualDisplay["Tumor"];

(* Create Rasters of the Xq Normal Datasets *)

rastercontrast = 1;
step = 5;

normal1Raster = rasterFileGeneration[normal1, rastercontrast];
normal2Raster = rasterFileGeneration[normal2, rastercontrast];
probes = Dimensions[normal1Raster][[1]];

(* Input the Xq Chromosome Band Coordinates *)

arm = Transpose[Join[Transpose[normalProbeLoc], {zNormalArraylets[[All, 1]]}]];

{bandLabels, patientLabels, xticks, yPosition, z} =
  figureVariables[armBands, arm, probes, "Normal"];

Map[(bandLabels[[#[[1]]]] = Text[Style[ToString[#[[1]]],
         FontFamily → "Courier", FontSize → ticksize], {-1.25, #[[2]]}, {1, 0}];) &,
  {{1, 25 - yspace + 0.5}, {2, 25 - yspace - 0.5}, {3, 25 - yspace - 1.95}, {10, z[[10]] + 0.1},
   {11, z[[11]] - 0.95}, {22, z[[22]] + 0.3}, {23, z[[23]] - 0.2}}];

(* Display the Xq Normal Datasets *)

normal1Raster = Graphics[
   Raster[normal1Raster, {{0, -65 - yspace}, {13, 25 - yspace}}, ColorFunction → RGBColor]];
normal2Raster = Graphics[Raster[normal2Raster,
    {{13, -52 + 13 - yspace}, {26, 38 + 13 - yspace}}, ColorFunction → RGBColor]];

(* Display the Xq Normal Arraylets *)

gNormalTensor = tensorDisplay[normal1Raster,
   normal2Raster, bandLabels, yPosition, xticks, patientLabels, "Normal"];

space = 43.5;
rastercontrast = 50;
step = 5;
{rasterArraylets, bandLabelsArraylets, arrayletylbls, arrayletylblpos} =
  arrayletVariables[zNormalArraylets, bandLabels, "Normal"];

gzNormalArraylets = arrayletsDisplay[rasterArraylets,
   bandLabelsArraylets, arrayletylbls, arrayletylblpos, xticks, yPosition, "Normal"];
```

```
(* Display the Xq Normal Core *)

rastercontrast = 0.01;
step = 1;
space = 82;
{coreRaster1, coreRaster2, corexlbls, corexlblpos, coreylbls, coreylblpos, xlist, ylist} =
  coreVariables[normalCore, rastercontrast, "Normal"];

gNormalCore = coreDisplay[coreRaster1, coreRaster2,
    corexlbls, corexlblpos, coreylbls, coreylblpos, xlist, ylist, "Normal"];

(* Display the Xq x- and y-Probelets *)

rastercontrast = 30;
step = 1;
{rows, columns} = Dimensions[xProbelets];
probeletsRaster = rasterFileGeneration[
    xProbelets[[Join[Range[1, 5], Range[rows - 4, rows]], All]], rastercontrast];

rastercontrast = 1;
step = 1;
space = 117 - 10.75;
{yProbeletRaster, probeletlbl, probeletxlblpos, probeletxlbls} =
  probeletVariables[yProbelets, rastercontrast, patientLabels, "Normal"];

gxNormalProbelets =
  xProbeletsDisplay[probeletsRaster, probeletlbl, probeletxlbls, probeletxlblpos, "Normal"];

gyNormalProbelets = yProbeletsDisplay[yProbeletRaster, "Normal"];
gNormalEqual = gEqualDisplay["Normal"];

(* Display the Xq Angular Distances Bar Chart *)

barsInset = angularDistance[tumorCore, normalCore];

(* Display the Xq Tensor GSVD *)

figB = Show[{gTumorTensor, gTumorEqual, gzTumorArraylets,
    gTumorCore, gxTumorProbelets, gyTumorProbelets, gNormalTensor, gNormalEqual,
    gzNormalArraylets, gNormalCore, gxNormalProbelets, gyNormalProbelets, barsInset},
  PlotRange → All, ImageSize → 750, AspectRatio → Automatic]
```

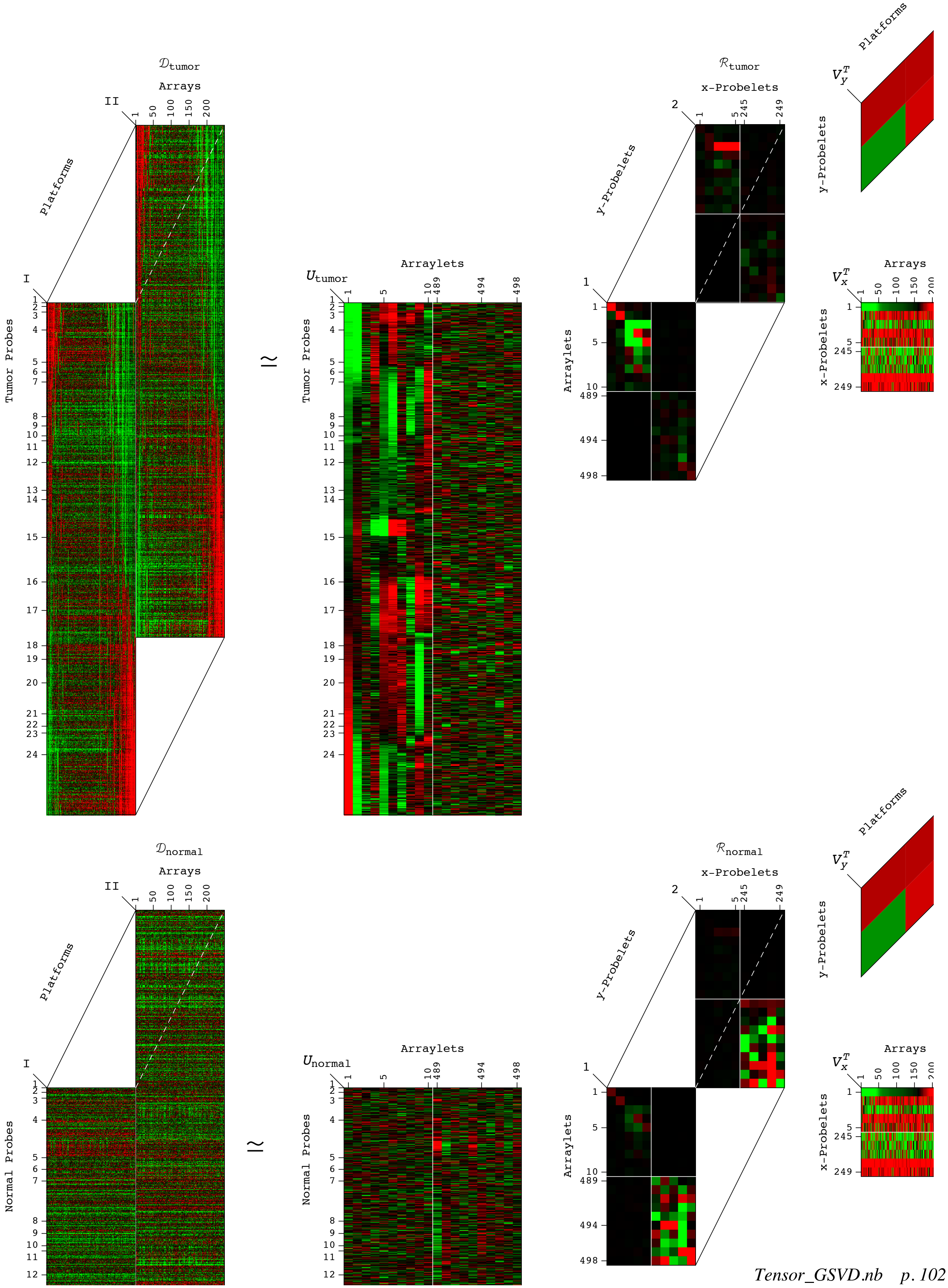

𝒟tumor
Arrays
I
II
Platforms
Tumor Probes
≈
Utumor
Arraylets
ℛtumor
x-Probelets
y-Probelets
Arraylets
Platforms
V_y^T
y-Probelets
V_x^T
Arrays
x-Probelets
𝒟normal
Arrays
Platforms
Normal Probes
Unormal
Arraylets
ℛnormal
x-Probelets
y-Probelets
Arraylets
Platforms
V_y^T
y-Probelets
V_x^T
Arrays
x-Probelets

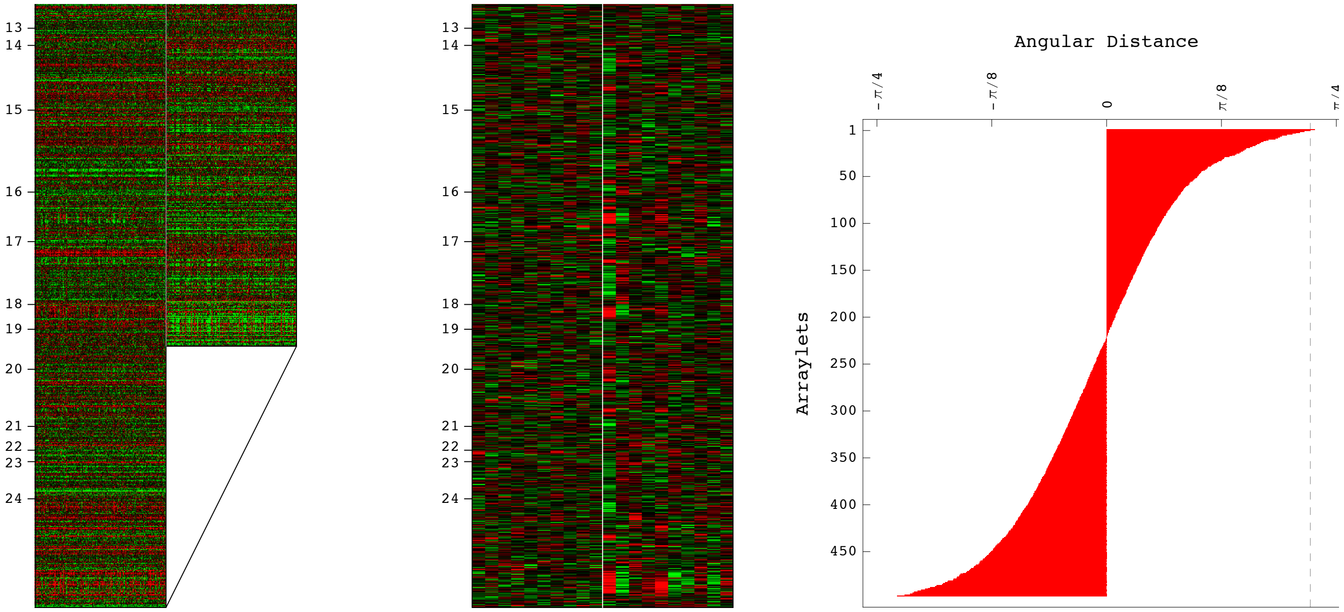


```
Export[path <> "Figure_B.pdf", figB, "PDF", ImageSize → 750];
```
